# In Vivo Models of Cardiovascular Disease: *Drosophila melanogaster* as a Genetic Model of Congenital Heart Disease

**DOI:** 10.3390/biomedicines13102569

**Published:** 2025-10-21

**Authors:** Theodora M Stougiannou, Maria Koutini, Fotios Mitropoulos, Dimos Karangelis

**Affiliations:** 1 Department of Cardiothoracic Surgery, University General Hospital, Democritus University of Thrace, 68100 Alexandroupolis, Greece; 2 Department of Paediatric, Congenital and Adult Cardiac Surgery, Mitera Hospital, Marousi, 15123 Athens, Greece

**Keywords:** drosophila, genetic, evolution, cardiovascular, cardiac, congenital, model, heart

## Abstract

*Drosophila melanogaster* (*D. melanogaster*) has been widely used in biology, including classical genetics, for almost a century. With the entire *D. melanogaster* genome sequenced and the existence of transgenic and mutant individuals, the species offers opportunities for targeted gene expression and manipulation. Genes involved in the regulation of the animal’s cardiac development include genes associated with the ancient regulatory networks that direct the formation of the cardiac form. However, additional loci can also affect cardiac development, including genes associated with cellular metabolism and protein homeostasis; signaling pathways necessary for the establishment of body segmentation and polarity; homeotic genes involved in the establishment of the animal body plan; and finally, genes encoding chromatin modification enzymes. Conservation in the genetic networks governing cardiac development between *D. melanogaster* and mammalian vertebrates, coupled with the absence of genetic redundancy in *D. melanogaster*, allows for the study and evaluation of mutations that could potentially disrupt cardiac development in the former. In this manner, phenotypes in *D. melanogaster* can be compared with phenotypes present in vertebrate animal models and human patients; this, in turn, allows for comparisons of gene function to be made across different species and for identification of candidate genes with a potential effect on cardiac development. These genes can then be further tested in vertebrate models with possible clinical implications. It is thus the purpose of this comprehensive literature review to summarize and categorize studies evaluating the results of genetic mutations on *D. melanogaster* cardiac development, as well as uncover any associations between *D. melanogaster* and similar phenotypes in vertebrates and humans due to effects on the corresponding gene orthologs.

## 1. Introduction

Congenital heart disease comprises defects in cardiac morphogenesis; it is the most common type of birth defect [1], with its incidence globally calculated at 8–9.5 per 1000 live births, though regional variability can exist [2]. Defects are often classified based on the presence/absence of cyanosis due to right-to-left shunting. Cyanotic defects include Tetralogy of Fallot, transposition of the great arteries, and total anomalous pulmonary venous return [3,4,5,6]. Non-cyanotic defects, on the other hand, can include both obstructive disease, including aortic stenosis and coarctation of the aorta [7,8], as well as left-to-right shunts. The latter comprises defects such as atrial and ventricular septal defects, patent ductus arteriosus, and atrioventricular canal defect [9,10,11,12].

Many defects are often diagnosed before or right after birth. Some, including Tetralogy of Fallot [13], may require surgical correction to maintain proper communication between right and left circulations [14]. Regarding causality, different factors can be identified, including inherited or de novo genetic mutations [15,16,17,18] and larger chromosomal aberrations such as aneuploidy and polyploidy syndromes [19,20,21]. For example, aneuploidy syndromes such as Turner syndrome or various Trisomy Syndromes can be associated with cardiac septum and outflow tract defects [22,23]. Aneuploidy syndromes can also often present with mosaicism; in this case, a set of cells exhibits chromosomal aneuploidy, while the remaining cells possess a normal karyotype [24]. Mosaic aneuploidy syndromes often result in longer survival with less severe characteristics [24,25,26] compared to their non-mosaic counterparts, as with, for example, mosaic Trisomy 13 [24]. They may also present with a normal phenotype altogether, as is the case with mosaic Trisomy 22 [27]. Subchromosomal aberrations, including 22q11.2DS deletion syndrome, also known as DiGeorge syndrome, can present with cardiac defects as well [20,28].

Since genetic causes are identifiable in about 35% of patients with the disease [29], the employment of models used to discover associations between individual candidate genes and their phenotype in simple organisms is indispensable [30]. Compared to other in vivo models, particularly mammalian models, which can often be more expensive and difficult to deploy for large-scale candidate gene screening due to ethical reasons [30], simple invertebrate models offer a better and often cheaper alternative. Specific genes for which a cardiovascular association has been found can then be evaluated further in more complex models [30,31]. The evolutionary conservation in the genetic networks that direct cardiac development allows for the use of distantly related animal species to study cardiac development [32]; however, the greater the evolutionary gap between organisms, the greater the difference in form and function, as opposed to species that relatively recently diverged [33]. Thus, while distantly related species can be used to evaluate the function of orthologous genes, more closely related species are chosen when recapitulation of relevant aspects related to form and function is required as well [1]. On the other hand, regarding *D. melanogaster* and other similar invertebrate organisms, they are often characterized not only by relative simplicity in form, at least compared to mammalian vertebrates/mammals, but can also exhibit simpler genetic networks. Around 13.601 *D. melanogaster* genes have been identified [34], with ~70 genes associated with congenital heart disease [35]. The simplicity in the cardiac gene networks governing *D. melanogaster* heart formation better facilitates the identification of single gene effects during the progression of cardiac morphogenesis; in addition, they can be used to discern whether a particular gene or locus may have any effect on heart development at all, if disrupted, in the case of candidate gene evaluations [36].

The purpose of this comprehensive review is to group and summarize studies that evaluate the effects of gene mutations on *D. melanogaster* cardiac development. A review of relevant literature ranging from 1990 to 2025 (present) has been carried out. Studies employing *D. melanogaster* as a screening tool, when these include genes affecting cardiac development, are also included. Overall, target genes have been grouped into categories based on their function; this includes genes comprising the cardiac regulatory networks that orchestrate heart development; genes involved in cellular metabolism and protein synthesis/trafficking; genes and factors involved in the migration and alignment of cardiac progenitors during assembly of the cardiac tube/dorsal vessel; genes involved in the establishment of segmentation, polarity, and other relevant signaling pathways; homeotic genes involved in the establishment of the animal body plan; and finally, genes encoding for histone modifiers and other chromatin-regulating enzymes with an effect on heart development. Orthologs of these genes in vertebrates/mammals, along with their effects on cardiac development. Finally, any associations between gene orthologs and human congenital heart defects have been incorporated as well.

In general, this text thus aims to compare the ancient cardiac gene network, along with any additional genes involved in heart development in *D. melanogaster*, to the more developmentally complex vertebrate cardiac gene networks. Similarly, phenotypes resulting from perturbations in these genes in *D. melanogaster* will be compared to the corresponding perturbations seen when orthologs of these same genes are disrupted in vertebrates/humans.

## 2. *Drosophila melanogaster*

### 2.1. Arthropod Cardiovascular Systems

Arthropod body plans exhibit a segmented architecture, which consists of a variable number of similar units, known as segments, along the anteroposterior axis [37]. Regarding the cardiovascular system, all arthropods generally possess an open circulatory system composed of a dorsally located contractile heart, arteries, and a hemocoel. Hemocoels are body spaces through which hemolymph circulates freely [38,39]. The contractile heart, in particular, is a solenoid structure composed of a posterior cardiac chamber and an anteriorly placed aorta from which it is separated by an aortic valve; this structure is known as the dorsal vessel. In the dorsal vessel, the aorta is situated in the thoracic segments, while the cardiac chamber is found within the abdominal segments. As with all arthropods, the circulatory system is open; hemolymph enters the heart via specialized ostia functioning as inflow tracts, flows through the dorsal vessel in a retrograde/anterograde manner depending on the conditions [40], and is finally pumped toward the body cavity. In this space, the hemocoel, the hemolymph flows and intermixes with both interstitial and extracellular fluids [30,41]. As with most arthropods [32], these systems are low-hydrostatic-pressure systems since the circulating hemolymph is not separated from interstitial fluids and thus not responsible for tissue oxygenation [42]. This contrasts with circulatory systems in mammalian vertebrates/humans, where interstitial fluids and blood are clearly separated, flowing through high-pressure circuits [43,44].

### 2.2. Anatomy and Histology of the Dorsal Vessel

In *D. melanogaster*, molecular distinction between the anterior dorsal vessel (aorta) and the posterior dorsal vessel (aorta, heart chamber), as well as variation in genetic expression along the anteroposterior axis, can be attributed to differential *Hox* gene expression [45]. In the embryonic/larval heart, the anterior dorsal vessel spans both thoracic and abdominal segments (T3–A4), while the posterior dorsal vessel is found in abdominal segments (A4–A7) [46]. In the adult fly, on the other hand, the anterior dorsal vessel is limited within thoracic segments (T1–T3), while the posterior dorsal vessel spans both thoracic and abdominal segments (T3–A5) [47]. Histolysis of abdominal segments A6 and A7 has already occurred during metamorphosis, altering the overall length of the dorsal vessel in the adult [46,47]. Regarding cellular composition, it is composed of a single cardiac cell layer derived from cardioblasts, surrounded by non-contractile pericardial cells [48]. Some of these pericardial cell populations, including odd-skipped (*Odd*)+ pericardial cells (OPCs), will eventually fulfill nephrocytic functions in the adult fly, participating in hemolymph filtration [49,50]. Since these cell types are closely related to hemopoietic lymph gland cells, they also participate in immune cell responses [51]. During embryonic life, there are three paired openings or ostia along the dorsal vessel, located at the boundaries of segments A5/A6, A6/A7, and A7/A8. They are abutted by specialized ostial cardiac cells and form passive inflow tracts [40,52,53]. No such openings can be found in the anterior dorsal vessel [47]. Intracardiac valves can also be identified, facilitating the unidirectional flow of hemolymph with each heartbeat. Intracardiac valves are composed of valve cells, specialized cardiac cells originating from tinman (*tin*)+ cardiac cells, which are characterized by their large intracellular volume and unique sarcomere arrangement [54,55].

In the anterior-most dorsal vessel, an outflow tract can be observed, formed by specialized Even-skipped (*Eve*)+ tinman+ (*tin*)+ pericardial cell (EPC) populations; these cells assemble into an outflow hanging structure, which attaches the anterior-most dorsal vessel cardioblasts to the cuticle [56] via further association with ladybird (*lb*)*+* heart-anchoring cells derived from the dorsal epidermis, generating a funnel-shaped tip [56]. Additional structures observed in this area include a pair of cardiac outflow muscles on either side, derived from the pharyngeal mesoderm [57]. EPC populations in the thoracic segments, under the influence of *Hox* genes such as Antennapedia (*Antp*), differentiate into wing heart pericardial cells (WHPCs), while EPC groups not subjected to *Hox* gene expression comprise an outflow hanging structure previously described in [56]. Additional pericardial cell groups include end-of-the-line pericardial cells (ELPCs) [51,58], found only in abdominal segment A7 [51] (Supplementary Table S1).

### 2.3. Growth of the Dorsal Vessel During Development

**Figure 1:**
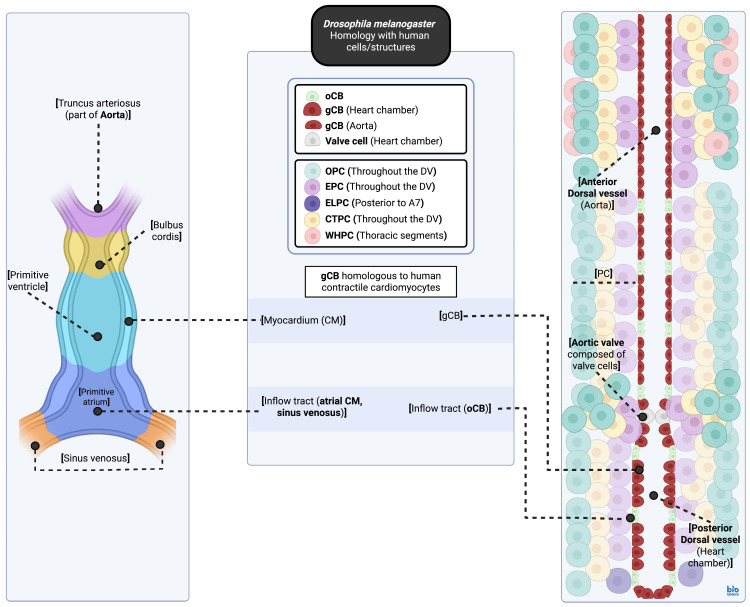
Comparison of *D. melanogaster* and *H. sapiens* embryonic heart structures, with cells carrying out similar functions highlighted. General cell group/tissue types found in each organism are also noted. Structures compared represent late-stage embryonic forms, and as such, the *D. melanogaster* dorsal vessel shown possesses only one aortic valve and three ostia on either side of the heart chamber. The single heart tube stage of cardiac development found in *H. sapiens* is shown to facilitate comparison, as later human embryonic stages diverge into more species-specific structures. For a complete list of all gene abbreviations, see Supplementary Table S9. CM, cardiomyocyte; CTPC, cut+/tinman+ pericardial cell; DV, dorsal vessel; ELPC, end-of-the-line pericardial cell; EPC, even-skipped+/tinman+ pericardial cell; OPC, odd-skipped+ pericardial cell; PC, pericardial cell; WHPC, wing heart pericardial cell; gCB, generic cardioblast; oCB, ostial cardioblast. [32,56,62,68,69,70]. Created in BioRender. Stougiannou, T. (2025) https://BioRender.com/wpq4alf.

The dorsal vessel enlarges mainly via cellular growth, alongside the enlarging animal body, during larval stages L1, L2, and L3; loss of some mononucleated pericardial cells occurs during this period [59,60]. Transition from pupa to adult form, also known as metamorphosis, is associated with a reduction in overall cardiac cell numbers (~104 to 84) and an increase in ostia pairs from three to five. There may also be evidence for the existence of a posteriorly located terminal opening in the adult, allowing for the retrograde flow of hemolymph during episodes of heartbeat reversal [40,52,61]. During the transition from larva to adult, the number of intracardiac valves also increases from one to three. Eventually, the adult structure is a four-chambered heart with an anteriorly located aorta and heart chambers arranged in series [62] (Figure 1).

A ventral longitudinal muscle layer inferior to the cardiac tube, derived from larval alary muscles, also emerges during metamorphosis. This layer morphologically separates the pericardial and abdominal cavities [63]. Alary muscles attach to the dorsal vessel indirectly via extracellular matrix proteins such as pericardin [64] and Viking, both of which are collagen IV-like proteins [65]. This allows the dorsal vessel to anchor to the outer epidermis and, as such, stabilize it within the body cavity of the fly [66]. Cardiac chamber remodeling also occurs during metamorphosis; posterior-most segments undergo histolysis, as has already been stated [46], and new neural connections are formed [41]. Accessory pulsatile organs facilitating hemolymph flow toward the limbs, wings, and antennae also emerge during this transitional period [52,67].

## 3. Cardiac Gene Regulatory Networks During Development

**Figure 2:**
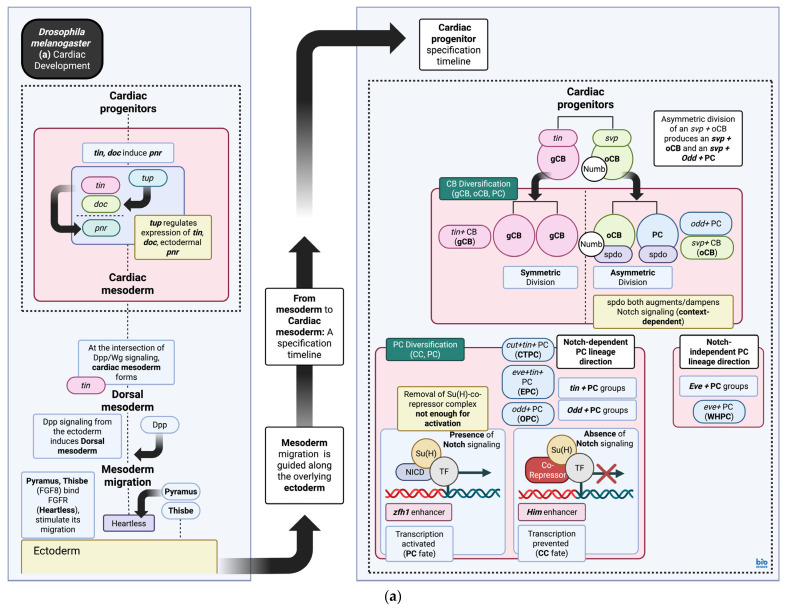

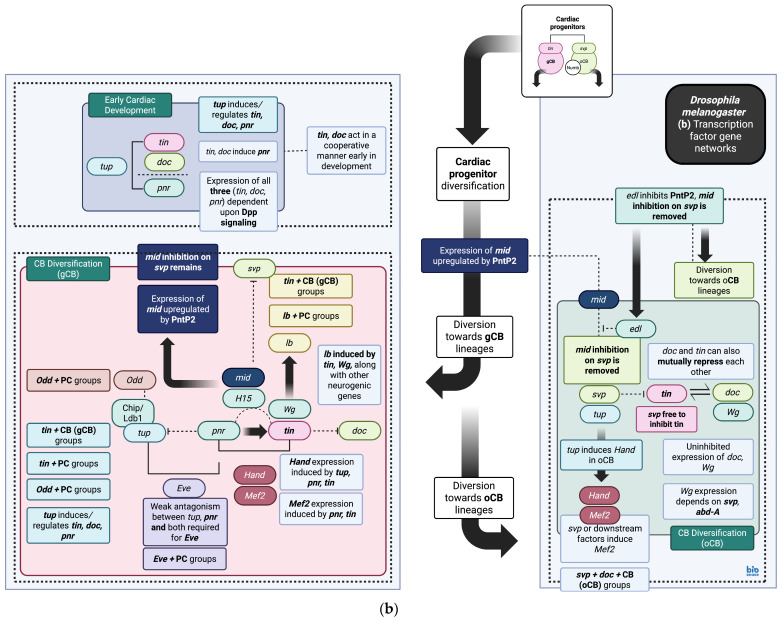
(**a**) **Cardiac transcription factor gene networks**: **Early stages of cardiac development and cardioblast proliferation**. A closer look at the cell specification timeline, from migrating mesoderm to cardiac mesoderm. Initially, FGF/FGFR signaling via the FGF8-like molecules Pyramus and Thisbe (originating in the ectoderm), and the FGF receptor Heartless (found in the mesoderm) allows for the migration of the mesoderm alongside the externally lying ectoderm. Additional signaling via Dpp (BMP signaling) induces specification of dorsal mesoderm and expression of tin, which will eventually aid in the specification of the cardiac mesoderm. Expression of *tin* is gradually restricted from the mesoderm to the dorsal mesoderm and, eventually, the cardiac mesoderm, along with doc. Specification of cardiac mesoderm occurs at the intersection of Dpp/Wg (ectoderm) signaling within cells expressing *tin*. Another early cardiac gene is tup, identified in the ectoderm, the Amnioserosa, and the mesoderm; in the ectoderm, *tup*, along with *pnr*, facilitates expression of *Dpp*, while in the mesoderm, *tup* can facilitate early expression of *tin*. As the cardiac mesoderm has been specified, *tin* and *doc* cooperate, collectively inducing the expression of *pnr* (mesoderm). *Tin* regulates the expression of both itself and doc. Later in development, cardioblast diversification occurs. *Tin*+ cardioblasts (gCB) will generate working contractile cardiac cells; they usually divide symmetrically to generate two *tin*+ cardioblast daughters. *Svp*+ cardioblasts (oCBs) divide asymmetrically; lineage diversion between cardioblast and pericardial cell fates depends on inhibition and activation of Notch signaling, respectively. Active Notch signaling induces the pericardial cell fate, while Notch suppression via the transmembrane protein Numb allows for diversion toward the oCB fate. This division is potentiated by spdo, which encodes for a transmembrane protein as well; when Numb is present, it inhibits spdo localization to the cell surface, inhibiting Notch signaling. In cell daughters with no Numb protein, spdo reinforces Notch signaling. Notch is activated by the transmembrane ligand Delta, present in the neighboring cardiac cells, thus inducing Notch signaling. Downstream of Notch/NICD (NICD forms part of the Notch receptor), pericardial cell fate is triggered by the activation of genes associated with the *zfh1* and *Him* enhancers. Via Him enhancers, this occurs with the removal of repressive transcriptional complexes, including the Su(H)/Co-repressor and other transcription factors (Notch-permissive), while with *zfh1* enhancers, removal of repressive complexes is not enough, as an additional transcriptional activation complex, such as NICD/Su(H), and other transcription factors are required (Notch-instructive). While some pericardial cells specify via Notch signaling (*tin*+ pericardial cells, *odd*+ pericardial cells), others (*eve*+ pericardial cells) specify in a Notch-independent manner, as gene transcription can occur in both the presence/absence of NICD, via Eve-specific transcription factors. Created in BioRender. Stougiannou, T. (2025) https://BioRender.com/t94v106. (**b**) **Cardiac transcription factor gene networks: Transcription factor gene networks during early cardiac development and cardioblast diversification**. A closer look at the transcription factor gene networks that direct cardioblast diversification. During early cardiac development, *tin* and *doc* are among the earliest transcription factor genes activated, with tup inducing expression of *tin* (cardiac mesoderm), *doc*, and *pnr* (ectoderm). *Tin* and *doc* can also induce the expression of *pnr*, while *tup*, in turn, regulates the expression of all three (*tin*, *doc*, *pnr*). Thus, during these early stages, *tin* and *doc* act cooperatively. As development progresses and progenitor populations diversify, *tin* and *doc* now antagonize each other, with *tin* repressing *doc* and *doc* also capable of repressing *tin* (mutual repression). During gCB lineage specification, *pnr* induces expression of *tin* via *mid/H15*; both *tin* and *pnr* can then induce the expression of genes relevant to the proliferation, survival, and differentiation of cardiac cells (*Hand*, *Mef2*). While *tup* can regulate the expression of *tin*, *doc*, and *pnr*, it can also induce the expression of *Odd* via interactions with the adaptor proteins Chip/Ldb1. In addition, both *tup* and *pnr* are required for the expression of *Eve*. Expression of *tup* can be identified in *tin+* CB (gCB), *tin*+, and *odd*+ PC. *Tin*, in concert with Wg signaling and other neurogenic genes, can also activate the expression of *lb*. In the gCB lineages, *mid* (which usually activates *tin*), is upregulated by PntP2, a transcription factor encoded by *pnt*. *Mid* will eventually suppress *svp*, preventing the *svp*-mediated inhibition of *tin* and thus ensuring direction toward the gCB lineage. On the other hand, inhibition of PntP2 by edl removes the mid-mediated repression on *svp*; *svp* is then free to suppress *tin* and allow for the activation of *doc*, ensuring direction toward the oCB lineage. As *tin* inhibition is removed, Wg expression can also be identified, a process that further depends on *abd-A* and *svp*. *Hand* and *Mef2* expression in oCB is triggered by *tup* and *svp*, respectively. PntP2 expression is generally induced by EGF/EGFR signaling, while PntP1 is constitutively active; both can activate *mid* in the gCB lineage diversification pathway, leading to inhibition of *svp* and thus allowing for the *tin*-mediated suppression of *doc*. As, Amnioserosa; BMP, bone morphogenetic protein; CB, cardioblast; CC, cardiac cell; CTPC, cut+ tinman+ pericardial cell; Chip, LIM domain-binding protein 1 (Drosophila ortholog); Dpp, Decapentaplegic; EGF, epidermal growth factor; EGFR, epidermal growth factor receptor; ELPC, end-of-the-line pericardial cell; EPC, even-skipped+ tinman+ pericardial cells; FGF, fibroblast growth factor; FGFR, fibroblast growth factor receptor; Him, holes in muscle; Ldb1, LIM do-main-binding protein 1; NICD, Notch intracellular domain; OPC, odd-skipped (Odd) pericardial cell; PC, pericardial cell; PntP1, Pointed P1; PntP2, Pointed P2; Su(H), Suppressor of Hairless; TF, transcription factor; WHPC, wing heart pericardial cell; Wg, Wingless; abd-A, abdominal-A; edl, ETS domain lacking; gCB, generic cardioblast; lb, ladybird; mid, midline; oCB, ostial cardioblast; zfh1, zinc finger homeobox 1. For a complete list of all gene abbreviations, see Supplementary Table S9 [50,51,56,58,76,77,78,79,80,81,82,83,84,85,86,87,88,89,90]. Created in BioRender. Stougiannou, T. (2025) https://BioRender.com/r76k422.

*D. melanogaster* embryos possess centrolecithal eggs that undergo superficial cleavage due to the presence of a centrally located mass of yolk [71,72]. Eventually, the blastoderm emerges during early development, comprising peripherally localized cells arranged around a central yolk; this structure is considered homologous to the blastula observed during embryonic development in other species [73]. Invagination of cells from this peripheral layer toward the center commences during gastrulation, during which stage, development of the cardiovascular system also commences [51,74]. This is facilitated by a complex interplay of transcription factors that comprise the core cardiac gene regulatory network, which directs specification of the cardiac mesoderm, leading to the eventual derivation of the various cardiac cell groups, as illustrated in Figure 2a, b, and described in detail in the associated figure captions (Figure 2). Additional details regarding the function of each gene described in this section, as well as all other sections of this text, along with information about their mammalian/human orthologs, can be found in Supplementary Table S8. As cardioblasts and pericardial cells diversify, they also undergo defined movements in space; though cardiac cell group aggregations can initially be seen bilaterally, these will eventually converge toward the center and align with those of the contralateral side. This migrational movement, observed as cardiac leading-edge activity, parallels the movement of the overlying ectodermal epithelium converging toward the midline as well. This movement is regulated by distinct signaling pathways, including FGF and Slit/Robo signaling [75], explored in more detail in Section 4. Eventually, a solenoid structure positioned in the dorsal midline of the animal body will form [32].

## 4. *Drosophila melanogaster* and Congenital Heart Disease

### 4.1. Evolution of the Heart

Primitive organs resembling a heart first appear in the tree of life about 500 million years ago; earlier hearts are generally simpler in structure, with more complex cardiac systems eventually appearing, reflecting specific adaptations depending on species and environment [36]. The appearance of bilaterians is associated with the emergence of the mesoderm [91], and with it, the cardiac mesoderm and the heart [92]. Development of the heart in these organisms is dependent upon a genetic regulatory network, although genes associated with this network, such as *Mef*-*2*, have been found in species without bilateral symmetry as well. This only highlights the primordial origins of this regulatory network [93]. Simple tubular heart structures are usually found in animals that have evolved earlier in the phylogenetic tree, including invertebrates and arthropods. Tubular hearts are also observed during the early stages of vertebrate embryonic development [92]. The occurrence of morphologically similar structures in developmental stages across different species represents an example of convergent evolution, often reflected in the model of the “developmental hourglass”. While initial developmental stages differ, many animals eventually reach a period that is morphologically conserved among different phyla, also known as the phylotypic period. Once this period is complete, development once again becomes less conserved among different species, leading to the emergence of different animal forms. The presence of a phylotypic period represents the need to generate a viable animal body plan, a process directed by gene regulatory networks conserved among different species [94]. Accumulation of genetic changes, such as gene duplications, has contributed to genomic evolution and the emergence of more complex genetic networks, in turn orchestrating the development of more complex vertebrate cardiac systems [33,36].

### 4.2. Homology Between Drosophila melanogaster and Homo sapiens

Relationships between organisms can be defined based on the presence of the most recent common ancestor; this allows for the correct identification of genealogical relationships. These relationships in the evolutionary history (phylogeny) of an organism [95] can be summarized with phylogenetic trees; in this manner, a graphical depiction of the evolutionary history of a particular species in relation to other closely related species can be carried out [96]. The phylogeny of a species [96] can differ from the genealogies of specific genes (gene trees) within the species [95]. Often, gene tree topologies, even when part of the same species, may differ, exhibiting a topology different from that of the organism where they are found [97]. This may reflect alterations in gene sequences due to various events, including horizontal gene transfer, which allows genetic material to flow between organisms in a manner other than vertical transfer; the latter usually occurs in the context of traditional relationships of descent [98]. In eukaryotic organisms characterized by a membrane-enveloped nucleus and germ-line segregation of genetic material, however, the rate of non-vertical gene transfer is greatly diminished compared to Bacteria or Archaea [99], though this topic is still under debate [98].

Homology, a term used for over 150 years, often with variations in definition, has come to describe the degree of similarity derived from an evolutionary relationship, owing to the presence of a common ancestor [100,101]. It is important to note that similarities alone are not enough to characterize homology [102], as similarities between organisms can be attributed to events such as convergent or parallel evolution [103], allowing similar features or characteristics to develop in non-closely related animals [104]. The concept of homology can be applied to genes as well; though a particular gene possesses a specific function in the ancestral species, over time, with the accumulation of genetic changes and eventual evolutionary divergence, homologous genes emerge across different species, exhibiting additional functions or new functions compared to the ancestral gene [100]. While homologous genes can be characterized by percent sequence similarity, sequence similarity itself is not a defining characteristic of homology, as once again, an evolutionary relationship must be present [105].

Homology can have many different forms, including partial homology [100], paralogy [106], xenology [107], and orthology [106,108]. Terms such as xenology and partial homology describe homologous characters due to horizontal gene transfer (xenology) [107], as well as differing homology relationships occurring between different areas within the same gene, a phenomenon often due to genetic recombination or other events affecting gene subregions (partial homology) [100]. Paralogous genes result from gene duplication and can often be found in the same species, as is the case with hemoglobin and myoglobin in *H. sapiens* [109] or the neuromancer 1 (*nmr1*) (*H15*)/neuromancer 2 (*nmr2*) (*mid*) and dorsocross *1*/*2*/*3* (*doc1*, *doc2*, *doc3*) genes in *D. melanogaster* [86,108]. Gene duplication has contributed to the emergence of more complex traits along the evolutionary timeline; for example, duplications in ancient Homeobox (*Hox*) gene clusters have been associated with the emergence of complex cardiac forms and cardiac chambers [110,111].

Orthologous genes are homologous genes that have emerged due to speciation events that led to the splitting of the evolutionary lineage [100]; they often retain similar or equivalent functions among different organisms [108]. They can be classified based on the number of orthologs that exist within each compared species, a phenomenon described as cardinality; 1-to-1 (1:1) pairwise orthology refers to the presence of one orthologous gene in each species; 1-to-N (1:N) refers to the presence of more than one ortholog in the other species, possibly due to duplication events in a previous ancestor and finally; and many-to-many (N:N) orthology refers to many orthologs found in both species under comparison [112]. To predict orthology between genes across different species, specialized tools can be used, including the Drosophila RNAi Screening Center Integrative Ortholog Prediction Tool (DIOPT) (https://www.flyrnai.org/cgi-bin/DRSC_orthologs.pl, accessed on 15 July 2025), which has also been used to describe orthologous relationships in this review as well. Through this tool, orthology predictions from other similar tools (Homologene, OMA, Isobase, Phylome, RoundUp, InParanoid, orthoMCL, TreeFam, and Ensembl Compara) can be integrated into one score, reflecting the number of tools that support an orthologous relationship [113].

Examples of 1:N orthology include the *tin*:*NKX2* pairing; the *NKX2* gene family comprises the genes *NKX2.1*, *NKX2.2*, *NKX2.3*, *NKX2.4*, *NKX2.5*, and *NKX2.6* [114], all considered paralogous genes originating from an ancient duplication event in vertebrates [115]. However, *NKX2* family genes are orthologous not only with *tin*, but also with genes such as scarecrow (*scro*) and ventral nervous system defective (*vnd*), which are involved in *D. melanogaster* neurogenesis [116,117], as well as bagpipe *(bap)*, which is involved in development of *D. melanogaster* midgut musculature [118]; this further highlights the complexity of orthologous gene relationships [108]. Though *tin* possesses an orthologous relationship with all *NK2* genes, the orthologous relationship between *tin*:*NKX2.5*, in particular, generates the highest DIOPT score [113]. Furthermore, while *tin* and the *NKX2* genes are all orthologous, their functional contribution to the developing cardiac system is different. Thus, though the fly *tin* is important for the emergence and specification of cardiac mesoderm, in vertebrates, the ortholog *NKX2.5* is not required for the very early stages of cardiac mesoderm specification, though it is, nevertheless, indispensable for physiological heart development [119,120]. In addition, there is functional redundancy between different *NKX2* paralogues, for example, with *NKX2.3* and *NKX2.5*, during cardiac development in vertebrates [121]. Despite these orthologous relationships, *NKX2* genes cannot generally substitute for *tin* with regard to its cardiogenic function in *D. melanogaster*, with the exception of zebrafish *NKX2.3;* in return, neither can *tin* substitute for *NKX2.5* in associated assays [122]. This showcases the functional divergence between these two gene groups in different species; alternatively, these observations may also reflect the loss of some cardiogenic functions that could have been present in the ancestral gene [122].

*Doc*, another gene involved in early cardiac development, comprises three paralogues, *doc1*, *doc2*, and *doc3* [123]; these are all orthologous to the vertebrate T-box transcription factor genes *TBX2*, *TBX3*, and *TBX6*, an example of N:N orthology [123]. *TBX6* is involved in mesoderm and paraxial mesoderm induction, as well as indirect induction of cardiovascular cell lineages in pluripotent stem cell lines in vitro [124]. *TBX2*, *TBX3*, *TBX4*, and *TBX5* in vertebrates are all orthologous to the *D. melanogaster* gene *bifid* (optomotor blind [*omb*]); in associated studies, removal or deficiency of *bifid* results in lethality and various defects in eye [125] and wing development [126]. Interestingly, however, the function of *bifid* in the fly cannot be rescued with *TBX2*, another example of the functional divergence observed between gene orthologs across different species. However, in the same experiment, *D. melanogaster* bifid protein administration also does not restore function, which may point to sensitivity in protein dosage as the explanation for the lack of phenotype rescue [127]. Additional genes identified in both *D. melanogaster* and *H. sapiens* include *Mef2*, with *MEF2* in vertebrates comprising the paralogues *MEF2A* through *MEF2D* [90]. Another example of gene paralogues in *D. melanogaster* includes the bric-a-brac (*bab*) locus with paralogues *bab1* and *bab2* [128]; *bab2*, in particular, is implicated in the diversification of cardioblasts/pericardial cell groups [129,130], while both *bab1* and *bab2* function synergistically in imaginal disk development [128].

Homology can be evident with morphological stages during development as well. An example of this is the gastrula, an embryonic stage identifiable across many different species, including *D. melanogaster* and *H. sapiens*, even though the developmental processes that lead to its emergence are different. In flies, for example, cells from the single-layered blastoderm surrounding the central yolk cell invaginate, a process described as epithelial folding. Eventually, they undergo changes that result in the generation of a three-dimensional embryonic gastrula [131]. In mammals and humans, on the other hand, the amount of yolk present during embryonic development is considerably less, and cells undergo more complex movements. These include ingression or cell detachment from an epithelial layer leading to individual movement [132]; involution or cell rolling against a surface [133]; and finally, convergent extension or convergence and extension, a method of cellular rearrangement aimed at deriving a specific shape via narrowing along one dimension and extending along another [134]. Thus, it is evident that while different mechanisms are active in each species, the form produced can be identified as a gastrula. The gastrula is found across many different species, from invertebrates such as *D. melanogaster*, anamniotes, and amphibians to amniotes such as reptiles, birds, and all the mammalian groups [133].

Developmental homology is also evident between the two species due to conservation in the genetic programs that culminate in the establishment of the body axes, particularly the anteroposterior and dorsoventral axes. This could be attributed to the presence of a common ancestor in both *D. melanogaster* and *H. sapiens* predating deuterostome and polyphyletic protostome divergence. Deuterostomes include the Chordata, which comprise mammalian species such as *H. sapiens*, as well as simpler organisms such as echinoderms and Hemichordata [135]; polyphyletic protostomes, on the other hand, include phyla such as arthropods, including *D. melanogaster* [136]. Though a dorsoventral axis exists in both species (*D. melanogaster* and *H. sapiens*), it is inverted between the two [137], with the nerve chord located ventrally in invertebrates [138] and dorsally in vertebrates [139]. This difference has been attributed to axis inversion or, alternatively, the presence of a common ancestor with diffuse dorsoventral axis organization [140,141]. As a result of this difference in orientation, cardiac structures in *D. melanogaster* are located dorsally, as opposed to their ventral localization in many mammalian vertebrates, including humans [142].

Regarding anatomical characteristics, there is no homology between *D. melanogaster* and *H. sapiens*; in the former, cardiac structure is tubular, with chambers arranged in series in the dorsal section of the animal body. In the latter, cardiac structure is considerably more complex due to additional events of rightward looping driven by left–right asymmetry [36,143], chamber/endocardial cushion development [144,145], and septae and valve formation [146,147], which occur after the single heart tube stage during embryonic development. Regarding physiological characteristics, however, the two hearts are considered homologous by some [148,149]; cardiac flow in *D. melanogaster* is pulsatile with cycle-dependent hemolymph transport, while characteristics such as flow velocities within the heart chambers and across the aorta, cardiac output, and mechanics relating to the function of the heart as a pump are comparable as well [148,149].

### 4.3. Drosophila melanogaster and Models of Congenital Heart Disease

The homology in physiological function, coupled with the conservation of gene regulatory networks that govern cardiac development in both *D. melanogaster* and mammalian vertebrates [150], allows for the use of the former in genetic cardiac disease modeling [84]. In these models, mutations in genes that drive developmental networks or generally affect the process of cardiac development can result in congenital heart disease [151]. In addition, the cardiac gene regulatory network is simpler in *D. melanogaster* due to fewer genetic redundancies [36,152]. In short, a subset of cardiac transcription factor genes becomes initially active, eventually activating other similar genes, as well as genes implicated in cardiac structure/function and associated signaling pathways. Transcription factors carry out this function by binding to cis-regulatory elements such as promoters, enhancers [153], and downstream promoter elements [154]. Mutations in these early factors can be easily tracked and assessed in *D. melanogaster*, as the animal does not depend on a cardiovascular system for oxygen transport; this facilitates the evaluation of phenotypes and the contribution of candidate genes in these phenotypes that would otherwise result in early embryonic lethality in vertebrate models [32,142].

#### 4.3.1. Methods for the Evaluation of Gene Function in *D. melanogaster*

Varying approaches can be used to investigate gene function in the *D. melanogaster* system, including loss-of-function studies [142], gene knockout, and gene knockdown. While knockdown comprises transcriptional/translational suppression in gene expression, causing reduced protein production without genome modification, knockout involves ablation of genes or larger genomic loci altogether [155]. Knockdown can include tools such as ribonucleic acid interference (RNAi) [156] and morpholino antisense nucleotide knockdown [157]. These, however, can also be associated with off-target effects [158] or phenotypes originating due to the toxicity of the products themselves; furthermore, they usually only lead to a partial loss-of-function phenotype [155]. Knockout, on the other hand, can include targeted nuclease-based approaches, such as ZFN [159], TALEN [160], and CRISPR/Cas9 [161]. While these systems can also be associated with off-target effects, these can be tackled with further refinements, including the use of more than one nuclease to achieve cleavage (Cas9 nickase [Cas9n]), as well as the refinement of short guiding RNA sequences [162,163]. Additional methods involving alteration in gene structure include in vitro mutagenesis; the gene products generated in these cases exhibit a change in function or reduced function [164]. In vitro mutagenesis can involve site-directed mutagenesis, usually employed in cases where the wild-type target sequence is known and involves the synthesis of an oligonucleotide primer. Changes induced in this manner involve substitutions or deletions [165]. Others, such as gene disruption mutagenesis or knockout, can involve DNA insertion and recombination techniques to abolish gene function, including the highly specific recombination knockout techniques mentioned previously, as well as techniques involving DNA-alkylating agents or DNA insertion using transposons, both of which lack target specificity [166]. Mutations that result in the complete absence of a gene product and its associated function are often called null or amorphic mutations [155]. Often, a continuous region within a chromosome can be absent or deleted, affecting several genomic loci, described as a deficiency. Deficiencies can be used to evaluate phenotype severity associated with a particular allele, constituting definitive null alleles [167].

Methods to increase expression levels in a gene of interest can also be applied, as this too can induce perturbation in cellular and molecular processes. While the term “overexpression” is often used interchangeably with terms such as “misexpression” or “ectopic expression”, the latter two are often used in studies involving metazoan models to describe the expression of a particular gene within a cell group, tissue, or developmental time frame that it is not normally found in [168]. However, many studies with metazoans, including *D. melanogaster*, use the term “overexpression” for this purpose as well [63,169,170,171]. Mechanisms used to induce gene overexpression can include mutations in the enhancer area of a gene, leading to increased gene expression [168]. Additionally, GAL4 systems can be employed, comprising a transcriptional activator isolated from yeast and modified to drive expression in a tissue-specific manner, along with the gene of interest or a transgene whose expression is controlled by an upstream activation sequence (UAS_G_), bound by GAL4. These systems have been very commonly employed in *D. melanogaster* studies to evaluate gene function and associated phenotypes [172,173]. Tools culminating in overexpression can also be employed to increase the expression of mutated genes that, when produced, still retain the ability to interfere with the function of other proteins, including the function of a wild-type protein. The phenotype produced with such mutations is usually dominant, hence the term “Dominant Negative” mutation [168], considered a non-loss-of-function effect [174].

Additional strategies for inducing gene overexpression include the use of heat-shock systems, comprising transgenes that include the gene of interest, along with a promoter derived from a heat-shock protein. Expression in these cases is dependent on applied temperatures [175,176]. Temperature-sensitive mutations involving genes that encode for a functional product at the permissive (low) temperatures and a non-functional gene product at non-permissive (high) temperatures can also be applied. Temperature instability in these cases is most commonly due to a thermolabile protein product, which can become unstable or exhibit defects in folding under non-permissive temperatures. Temperature-sensitive mutations are useful for inducing changes in gene expression, including loss-of-function, at desired timepoints during an experiment [177]. These are usually classified as conditional mutations [178].

#### 4.3.2. Genes Involved in the Cardiac Gene Regulatory Networks: Mutations and Phenotypes

Many mutations contributing to congenital heart defects, both as de novo mutations and inherited or syndromic mutations, can be attributed to disruptions in cardiac transcription factor genes. As mentioned earlier and throughout this text, these genes encode for transcription factors that will, in turn, regulate the expression of similar or other gene types, all collectively involved in cardiac morphogenesis. Early factors activated during morphogenesis include *msh-2*, *tin*, and tailup (*tup*). The gene *msh-2* encodes a homeobox transcription factor implicated in early mesoderm development; as a result, *msh-2* loss-of-function studies exhibit a complete absence of the dorsal vessel and visceral muscle in *D. melanogaster.* In other cases, although somatic muscles can be identified, they are often abnormal [179]. In murine models, mutations in the *msh-2* ortholog *MSX-2* affect cardiac mesoderm precursors that will eventually assemble into the outflow tract, leading to defects in morphogenetic rotational movements in the truncus arteriosus [180].

Among the earliest cardiac transcription factors identified in *D. melanogaster* experiments, *tin* is involved in dorsal mesoderm and cardiac mesoderm specification, as well as cardiac development and cardioblast diversification [181,182,183]. Null mutations involving *tin* usually result in the complete absence of cardiac/dorsal somatic muscle, with disruption in somatic muscle arrangement in each segment [181]. Mutations also affect the expression of another early transcription factor gene, *doc*, from Stage 12 of embryonic development and onward [182]. The *tin* gene also possesses a downstream core promoter element, which, when affected by site-directed mutagenesis, exhibits reduced expression; additional targets also affected include *doc*, *svp*, *Mef2*, and *Odd.* This eventually leads to specification of fewer cardioblasts with functional defects in the dorsal vessel [161]. In line with similar experiments [181,182,183], somatic and visceral muscles are not as affected [161].

*NKX2.5* is also indispensable for cardiac development in vertebrate models. However, *NKX2.5* is not necessary for the initial stages of cardiac mesoderm specification [161,184], even though ablation of *NKX2.5* in early developmental stages still leads to embryonic lethality and cardiac defects in murine models [185]. *NKX2.5* ablation in later stages affects the development of the ventricles [186], ventricular septum, and cardiac conduction system and can lead to arrhythmias [185]. Various *NKX2.5* variants have also been associated with atrial septal defects and hypoplastic left heart syndrome in humans [187]. Furthermore, though downstream promoter region motifs have been identified in *NKX2.5*, their effect on *NKX2.5* levels is yet to be determined [161]. The effects of *NKX2.5* variants, including the variant K158N (*D. melanogaster* ortholog *R321N*), have been examined as well. In individual flies, it is associated with defects in differentiation, although initial cardiac specification occurs normally [183]. Through the *D. melanogaster* model, this variant has been associated with a pathophysiologic mechanism involving disruption between DNA and cofactor binding [183]. Thus, although the variant is still of unknown clinical significance, the phenotypes demonstrated both in vitro and in vivo may point to some effects that may also be present in vertebrate/human populations as well, requiring further study [183].

Additional transcription factor genes whose perturbation leads to visible cardiac defects in the *D. melanogaster* model include *svp* [188]; the paralogues *H15* (*nmr1*) and *mid* (*nmr2*) [113]; the paralogues *doc1*, *doc2*, and *doc3* [78]; *Eve* [189]; *tup* [80]; *Hand* [190,191]; and *D-mef2* [113]. Loss-of-function mutations in *tup* result in a hypoplastic dorsal vessel with severe morphological defects, including gaps and distortion in the structure, along with a reduction in cardioblast populations [80,81]. This gene is also expressed in valve cells, alary muscles, and thoracic–alary-related muscles; mutations in these cases usually affect the myofibrillar organization of valve tissue [62,192]. The vertebrate orthologs *ISL1* and *ISL2* are also similarly required during early development as part of the early cardiac transcription factor network. They are involved in the regulation of second heart field progenitor groups as these emerge and expand, contributing to the development of the outflow tract [193]. *ISL1* further contributes to the development of the atrial septum, the sinoatrial and atrioventricular nodes [194], and the endothelial and vascular smooth muscle cell groups [195]. In vertebrate models, *ISL1* knockout in mice has been associated with the complete absence of the atria, the right ventricle, and the outflow tract [193], while defects in cardiac looping and development of the arterial pole have been described with *ISL2* mutations in zebrafish [196]. In human genetic studies, *ISL1* variants and mutations have been described in cases of ventricular septal defects and double outlet right ventricles [197].

The *TBX20* transcription factors *H15* and *mid* also participate in early cardiac development, with mutations in *D. melanogaster* affecting the expression of other transcription factors. More specifically, *H15*/*mid* mutations are associated with a reduction in *tin*; upregulation in *Eve* and *Odd* expression; and finally, effects on cardioblast/pericardial cell diversification divisions, cardioblast alignment, and the general spatial arrangement of the cells in the midline [113,198,199]. If mutations are reproduced in adult animals, these usually bring about functional disruption in cardiac structure/myofibrillar arrangement [200]. In *D. melanogaster* studies, this is often measured as the effect on cardiac function (heart failure) induced by a stressor, in this case, in the form of electrical pacing [201]. Similar to the interaction between *H15*/*mid* and *tin*, *TBX20* interacts with *NKX2.5*, revealing a genetic association that has persisted throughout multiple lineage diversifications and across different species [202]. *TBX20* similarly interacts with *GATA4*/*5* and *TBX5* [203]. In vertebrates, *TBX20* contributes to the development of the atrioventricular canal and ventricular cells, while *TBX20* knockdown in murine models is associated with hypoplasia of the right ventricle and outflow tract, valvular defects, and outflow tract septation anomalies [204]. *TBX20* mutations and variants have also been associated with heart defects in humans, including septal defects, double outlet right ventricle [205], congenital mitral valve prolapse/regurgitation, congenital defects in the conduction system [203], bicuspid aortic valves, and hypoplastic left heart syndrome [205].

As previously mentioned, *doc* comprises three paralogues, *doc1*, *doc2*, and *doc3*; the absence of these genes in *D. melanogaster* is associated with embryonic death [78]. *Doc* genes exhibit orthologous relationships with *TBX6*, *TBX2*, and *TBX3*; in vertebrates, *TBX6* is involved in left–right patterning during early mouse development [206], the regulation of skeletal musculature development [207] via effects on axial and paraxial mesoderm development, and regulation of cardiac progenitor differentiation in vitro [124]. *TBX2* is involved in the development of the outflow tract and atrioventricular canal, while *TBX3* is associated with the development of both atrial and ventricular cardiomyocytes, as well as the cardiac conduction system [78,208]. *TBX6* disruption has been associated with skeletal defects [209], while a deletion in the genomic locus that also contains *TBX6* has been associated with pulmonary atresia with ventricular septal defect, a severe form of Tetralogy of Fallot in humans, along with other candidate genes [210]. *TBX2* mutations are associated with defects in outflow tract septation and atrioventricular canal development in animal models [208] and contribute to the development of Tetralogy of Fallot, single ventricle, and single atrium defects in humans. Both *TBX2* and *TBX3* have been associated with craniofacial defects in animal models [211]. *TBX3* has also been implicated in congenital heart defects in *H. sapiens*, including Tetralogy of Fallot, here as well, along with transposition of the great arteries [212].

*Eve* is mostly associated with the diversification of cardioblast/pericardial cell populations in *D. melanogaster* models, and related defects include disruption in pericardial cell populations [189]. In vertebrates, the corresponding orthologs, *EVX1* and *EVX2*, are involved in the development of limbs and genitalia [213], but no cardiac defects have yet been associated with either, as most cases described in the literature describe defects in limb development [213,214]. The transcription factor gene *svp* is another factor that contributes to the diversification of cardioblast/pericardial cell groups, with loss of expression usually associated with a corresponding loss of cardioblasts that express *svp* [113,188]. These cardioblasts normally go on to form specialized cardiac cells that line the ostia in the dorsal vessel, functioning as inflow tracts for the circulating hemolymph [113,188]. In vertebrates, one of the *svp* orthologs corresponds to *NR2F2*, a gene that regulates epithelial-to-mesenchymal transition, and contributes to and later maintains atrial cardiomyocyte identity [215]. *NR2F2* is expressed in the developing atria, aorta, and coronary vessels [216] and also contributes to the development of the atrioventricular canal [215] and coronary vessels [217]. Since ostia can be thought of/function as inflow tracts [113,188], similar to atrial chambers in the vertebrate heart, this could point to a conserved function across different cardiac systems. *NR2F2* mutations in humans have been associated with various septal defects, including atrioventricular canal defects [216], double outlet right ventricle, and Tetralogy of Fallot. *NR2F2* variants/mutations that affect the cooperation of *NR2F2* with *GATA4* have also been associated with congenital bicuspid aortic valve [218].

Finally, *Hand* is a bHLH transcription factor, and *D-mef2* encodes transcription factors that are mainly associated with activation of structural and functional genes in cardioblasts/pericardial cells and hematopoietic progenitors [78]. Mutations in these genes are associated with dorsal vessel hypoplasia (*Hand*) [191] and cardiac tissue differentiation defects (*D-mef2*) [190]. In vertebrates, *HAND2* interacts with Notch signaling and is involved in the development of the endocardium, ventricular trabeculation and septation, and coronary vessel maturation [219]. *MEF2C* and *MEF2A*, vertebrate orthologs of *D-mef2*, are involved in the development of the right ventricle, cardiomyocyte development and differentiation, and cardiac looping [220]. The contributions of these factors to cardiac development is further evident by the effects of their mutations, as in animal models, *HAND2* mutations are associated with defects in ventricular myocardial tissue, along with reduced trabeculation and defects in septation [219]; the *MEF2C* and *MEF2A* mutations are also associated with the failure of right ventricular development and cardiac looping defects [220,221] (Table 1 and Supplementary Table S2).

**Table 1:** *D. melanogaster* genes comprising the core cardiac regulatory network and corresponding orthologs with the highest DIOPT score, along with any associations with congenital heart defects in humans. ASD, atrial septal defect; BAV, bicuspid aortic valve; DORV, double outlet right ventricle; HLHS, hypoplastic left heart syndrome; MR, mitral regurgitation; MVP, mitral valve prolapse; PDA, patent ductus arteriosus; PFO, patent foramen ovale; PTA, persistent truncus arteriosus; TOF, Tetralogy of Fallot; VSD, ventricular septal defect. For a complete list of all gene abbreviations, see Supplementary Table S9.

Gene	Ortholog	DIOPT Score	Congenital Heart Defect	Reference
*msh-2*	*MSX1*	16	VSD	[222]
	*MSX2*		Dextrocardia, dextroversion, and PFO; radial agenesis with Hunter McAlpine syndrome (mental retardation, craniofacial and skeletal abnormalities, characteristic facial attributes)	[223]
*tin*	*NKX2.5*	5	VSD, ASD, HLHS	[10,187]
*tup*	*ISL1*, *ISL2*	16	DORV in combination with VSD (heterozygous mutations)	[197]
*H15*	*TBX20*	11	DORV, VSD, ASD, TOF, PTA, PFO, BAV, MVP/MR, total anomalous pulmonary venous connection, congenital atrioventricular block, HLHS	[203,205]
*mid*		13		
*doc1*, *doc2*, *doc3*	*TBX6*	10	Pulmonary atresia with VSD (severe form of TOF)	[210]
	*TBX2*		TOF, single ventricle, single atrium	[212]
	*TBX3*		TOF, transposition of the great arteries	[212]
*svp*	*NR2F2*	13	DORV, VSD, ASD, TOF, PDA, BAV	[217]
*Eve*	*EVX1*	10	Defects in limb development	[213,214]
	*EVX2*		*EVX1* and *EVX2* have not yet been associated with congenital heart defects in *H. sapiens*	
*Hand*	*HAND2*	15	DORV, VSD, pulmonary stenosis, outflow tract malformations	[214,224]
*D-mef2*	*MEF2A*, *MEF2C*	13	DORV, VSD, PDA, pulmonary atresia with VSD	[225,226,227,228]

#### 4.3.3. Genes Involved in Cellular Metabolism and Protein Synthesis/Trafficking: Mutations and Phenotypes

Additional genes involved in lipid [229,230,231] and glucose metabolism [232], as well as genes implicated in proteostasis [151,233], can also contribute to heart development and thus be implicated in the pathological heart phenotypes observed in the *D. melanogaster* model. HMG-CoA reductase (HMGCR), along with other enzymes in the mevalonate pathway and the G protein Gγ1, are all implicated in cardioblast–pericardial cell associations; in particular, modification of Gγ1 by geranylgeranylation allows for its appropriate intracellular localization, facilitating adhesion between cardioblasts and pericardial cells. Mutations in these enzymes result in the “broken-hearted” phenotype in flies, with cardioblast–pericardial cell dissociation and embryonic lethality [229]. HMGCR inhibition in humans has been reportedly associated with both cardiac (atrial and ventricular septal defects, hypoplastic aorta) and central nervous system malformations [234,235]. Glucose metabolism can also lead to derangements in cardiac development via an effect on endothelial nitric oxide synthase transcription. More specifically, hyperglycemia can reduce transcription at the *Nos3* locus encoding for endothelial nitric oxide synthase, leading to increased expression of Jarid, a regulator of histone methyltransferase. As a result, there is reduced nitric oxide production. Eventually, Notch expression is inhibited, and with it, the progression of cardiac development [232]. Hyperglycemia, in concert with genetic mutations, can affect cardiac development in the *D. melanogaster* system, with effects on myofibril arrangement and fibrosis, further shedding light on the mechanisms implicated in the cardiac malformations in infants of hyperglycemic mothers [232]. Evaluation of genes involved in proteostasis with unknown function in the context of congenital heart disease has shown variable defects in cardiac development in the fly, ranging from complete absence of the dorsal vessel to minimal effects on myofibril and actin organization [233], as well as partial to complete dorsal vessel atrophy [151]. Evaluation of the genes found to be implicated in hypoplastic left heart syndrome in humans has also been carried out, with relevant fly phenotypes ranging from cardiac dilation and disruption in adenosine triphosphate synthesis to mitochondrial defects [230,231,236,237]. Genes associated with Tetralogy of Fallot and hypertrophic cardiomyopathy in mammalian vertebrates/humans have also been evaluated in *D. melanogaster*, with results ranging from cardiac constriction to cardiac dilation and effects on embryonic survival [152].

**Figure 3:**
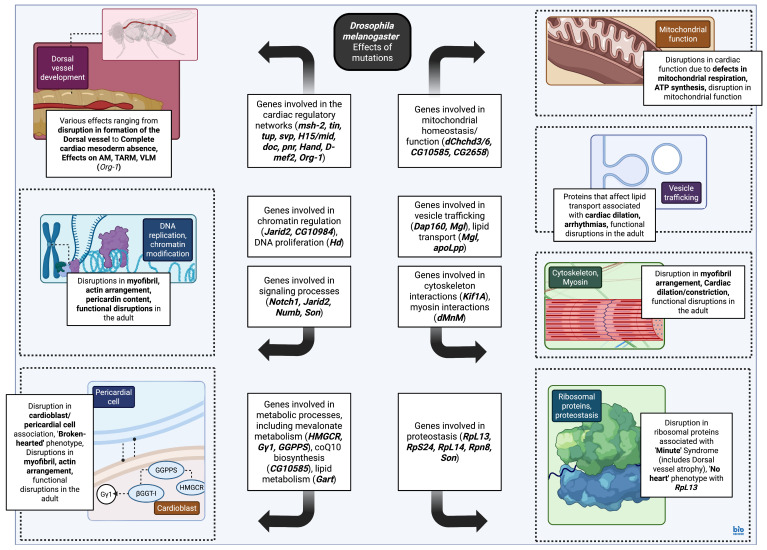
Effects of mutations affecting genes involved in cardiac gene regulatory networks, cellular metabolism, and protein synthesis/trafficking. AM, alary muscle; ATP, adenosine triphosphate; DNA, deoxyribonucleic acid; TARM, thoracic alary-related muscle; VLM, ventral longitudinal muscle; apoLpp, a homolog of apolipoprotein B; coQ10, Coenzyme Q10. For a complete list of all gene abbreviations, see Supplementary Table S9 [62,63,80,113,127,151,152,179,181,182,188,190,191,192,198,199,200,229,230,232,233,236,237,238,239,240,241,242,243,244] Created in BioRender. Stougiannou, T. (2025) https://BioRender.com/yiig8v2.

While *D. melanogaster* exhibits distinct progenitor populations after cardioblast diversification events, no grouping analogous to the first heart field and second heart field present in mammalian vertebrates can be identified. Instead, genes with homology to these populations are distributed across all cardiac progenitors in the fruit fly [51]. Recent evidence, however, suggests that the ventral longitudinal muscle may be an appropriate model for the study of genetic interactions implicated in second heart field [90] development, as derivation of the ventral longitudinal muscle is facilitated by the *Org-1*-mediated suppression of *tup* [63,238], a genetic interaction mirrored in second heart field development with the *Org-1* ortholog *TBX1*, the *tup* ortholog *ISL1*, and FGF/FGFR signaling [63,90]. Despite similarities in the genetic network, however, this interaction leads to modified skeletal muscle formation in *D. melanogaster* and cardiac muscle formation in mammalian vertebrates [90] (Supplementary Table S3 and Figure 3).

#### 4.3.4. Genes Involved in Cardiac Progenitor Migration, Alignment, and Dorsal Vessel Assembly During *Drosophila melanogaster* Embryonic Development: Mutations and Phenotypes

As cardiac mesoderm becomes specified and differentiates to eventually generate progenitors such as cardioblasts and pericardial cells, it also undergoes defined movements in space. Cardiac mesoderm can be initially seen bilaterally, appearing as segmented sections, owing to the combined action of Dpp and segmented Wg expression [182]. Though it has been previously thought that the cardiac mesoderm moves passively as a result of its attachment to the overlying ectoderm, it is now known that cardiac progenitors move autonomously as a result of cellular and intercellular events [84]. Migrating cellular groups move dorsally, eventually making contact with contralateral populations, an event associated with dorsal closure of the embryo [84]. Migration, alignment, and positioning of cells across one another are mediated via conserved pathways employing Slit/Roundabout (Robo) and Roundabout2 (Robo2) signaling [77,85]. Both Slit and Robo are expressed in the same cell, acting in an autocrine manner [245]; the proteins accumulate between rows of migrating cells, facilitating their alignment [77]. Furthermore, Slit/Robo facilitates apicolateral cell polarization in relation to the presumptive lumen by cooperating with disks-large (*dlg*), dystroglycan (*dg*), and shotgun (*shg*) [246]. Slit/Robo is further regulated by *nmr* [77,199].

Slit/Robo signaling also involves integrins and their transmembrane receptors; in general, integrins in *D. melanogaster* comprise three alpha (α) subunits (αPS1, αPS2, and αPS3) encoding for the proteins known as multiple edematous wings (Mew), Inflated (If), scab (scb), and 2 beta (β) subunits (βPS, βν). βPS encodes for myospheroid (mys) [247]. Integrins localize on the presumptive luminal aspect of migrating cardioblasts, guiding their alignment and polarization [83]; they also facilitate intercellular connections between alary muscle and pericardial cells [247,248]. Integrins accumulate apically in the cell due to the effects of Robo, and in return, apical localization of Slit/Robo is facilitated/stabilized by integrins [83]. Usually, sites between contralateral cardioblasts that will eventually form the lumen are repulsed due to Slit/Robo interactions, while areas in the dorsal and ventral areas attach via DE-Cadherin interactions, facilitated by Shg [249]. Slit/Robo also facilitate the formation of the outflow tract [57], while integrins further regulate the localization of pericardin, which, under physiological conditions, is found in the basal cardioblast domain between adjacent cardioblasts and pericardial cells [83,250]. Once cells reach the midline, dorsal interconnections between cells are generated, and afterward, ventral interconnections. The latter are usually mediated by cell division control protein 42 (Cdc42), a small GTPase protein that is part of the actomyosin network, along with other proteins that regulate actin polymerization [251]. Migrating cardioblasts exhibit cellular protrusions rich in actin, which are regulated by actin regulator proteins such as Enabled (Ena) [84,252]. Migration is facilitated by matrix metalloproteinases, mutations in which usually lead to variable defects in lumen formation and disruption in the collective cardioblast migration, resulting in “cardia–bifida” [253,254]; similarly, matrix metalloproteinase mutations contribute to “cardia–bifida” in vertebrates as well [255].

To achieve this regulation, Cdc42 interacts with *tin*, Zipper (non-muscle myosin) [256], and dishevelled-associated activator of morphogenesis (dDAAM) [257], a member of the diaphanous-related formin (DRF) family [258,259]. Cdc42 also facilitates heart function in adult flies via its effect on the K+ channels, and the interaction between *cdc42*/*tin* is conserved in mammalian vertebrates, with disruptions usually leading to increased QRS intervals and other arrhythmias [260] (Supplementary Table S4).

#### 4.3.5. Genes Involved in the Establishment of Segmentation and Polarity During *Drosophila melanogaster* Embryonic Development: Mutations and Phenotypes

Mesoderm migration in *D. melanogaster* occurs in response to FGF signaling mediated via the FGF8-like ligands Pyramus, Thisbe, and the FGFR receptor Heartless; Pyramus and Thisbe originate in the ectoderm, and Heartless is found in the mesoderm [261]. In vertebrates, FGF signaling is similarly involved in the coordination of cellular movements during gastrulation, specification of axial/paraxial mesoderm, and dorsoventral patterning with specification of dorsal and posterior cellular fates, always in coordination with other similar morphogens [262]. Mutations in the FGF signaling pathway thus affect early mesoderm migration and disrupt cardioblast and pericardial cell diversification in later stages [261]. In vertebrates, FGF10 has been implicated in cardiomyocyte proliferation during the elongation phase of the linear heart tube via recruitment of second heart field cardiac progenitors. While FGF10 mutations are associated with defects in pulmonary arteries/veins and ventricular apex localization, *Fgfr2b* mutations affecting FGFR function are implicated in ventricular septal defects, poor ventricular trabeculation, and defects in the alignment of the outflow tract [263]. In addition, FGF8, along with BMP2/4, also has a place in vertebrate cardiac development, participating in second heart field proliferation, migration, and formation of the arterial pole in the developing linear heart tube [264].

Eventually, the cardiac mesoderm emerges, a process that involves signaling via Dpp for specification of the dorsal mesoderm and later via combined Wg/Dpp signals for eventual cardiac mesoderm derivation. *Wg* is a segment polarity gene, expressed in the overlying ectoderm in a segmental pattern; it is implicated in the development of the nervous system, body segmentation, and heart morphogenesis [265]. *Dpp* encodes for a BMP-like protein and participates in the dorsoventral patterning of the *D. melanogaster* embryo; Dpp when combined with Wg signaling, culminates in the eventual specification of cardiac mesoderm [176,266,267]. Disruption of Wg/Dpp signaling affects mesoderm and cardiac mesoderm specification and can cause ectopic heart tissue formation in cases of overexpression [266,268]. Disruption of Wg/Dpp signals in later developmental stages leads to disruption of cardioblast and pericardial cell diversification [267].

The need for Wg signaling is mirrored in mammalian vertebrates, albeit in a more complex manner; in these animals, canonical Wnt signaling can both induce and suppress mesoderm specification. While mesoderm induction requires Wnt signaling, the cardiac mesoderm specification that follows does not; on the contrary, it is suppressed by Wnt signaling. This helps to more clearly demarcate areas where cardiogenic tissue will eventually appear in the embryo [269]. In zebrafish, expression of Wnt8 right before gastrulation increases the number of cardiac progenitors that will eventually be generated afterward, while expression of Wnt8 after gastrulation onset, during which time cardiac development also transpires, prevents the further generation of cardiac progenitors [270]. Regarding other Wnt ligands, Wnt8a is expressed throughout the developing vertebrate heart; Wnt2a/Wnt2b are associated with the atria and inflow tracts; and finally, Wnt5a and Wnt11 are expressed mainly in the outflow tract [271]. Furthermore, while canonical Wnt signaling is associated with the development of cardiac valve cells in mammalian vertebrates, in *D. melanogaster*, pygopus has been associated with this event instead [55]. Although pygopus is a component of the canonical Wnt signaling pathway, with its product functioning alongside Wg, Armadillo, and T cell factor/lymphoid-enhancer factor (TCF), no interactions have been observed between it and other components of the pathway during D. melanogaster heart development. This may suggest that pygopus functions independently of Wnt signaling, via a mechanism that affects actin organization and arrangement [55]; in mammalian vertebrates, similar mechanisms are mediated via non-canonical Wnt/planar cell polarity (PCP) signaling [272]. Evidence of non-canonical signaling in *D. melanogaster* may also be found during *svp*+ cardioblast during specification [170,171]. BMP signaling is also implicated in heart development in vertebrates, including the maintenance of *NKX2.5* expression [273], while in zebrafish, BMP signaling can also facilitate cardiac tissue regeneration [274]. Most *Wg*/*Wnt* mutations in *D. melanogaster* disrupt early stages of dorsal vessel development, with severe cases leading to absence of heart formation; the early pattern of activation of Wg signaling in the migration of the mesoderm also translates into widespread defects resulting in embryonic lethality, affecting both somatic and visceral muscles, as well as variable defects in ectoderm and endoderm development [176,275,276]. In later stages, disruptions in the diversification of cardioblast and pericardial cell populations also occur [265], particularly affecting the expression of *svp*, *Eve*, and *Odd* [170,171], along with defects in cardiac valve cell formation [55,277,278]. In vertebrates, loss of Wnt5a has been associated with outflow tract defects such as persistent truncus arteriosus [279], loss of Wnt11 with ventricular septal defects and double outlet right ventricles in mice [280], and ventricular septal defect and Tetralogy of Fallot in humans [281]. Both ligands (Wnt5a and Wnt11) normally signal through the non-canonical Wnt pathway [282].

Hedgehog (Hh) signaling in *D. melanogaster* maintains segmental *Wg* expression [283] and regulates the development of various heart progenitor groups. This is carried out via RAS/MAPK signaling owing to effects on the EGFR-associated protease rhomdoid, involved in the specification of *eve+* populations. FGF signaling via Heartless also converges on the activation of RAS. Alternatively, Hh inhibits Cubitus interruptus (Ci), which normally inhibits this pathway, thus removing the inhibition and allowing for upregulation of *eve*+ populations and the suppression of *lb+* populations instead. As a result, Hh mutations can lead to variable effects on heart development, depending on timing, ranging from decreases in heart progenitor populations to disruption in the diversification of cardioblast and pericardial cell groups [176,284]. In vertebrates, Shh signaling regulates the timing of cardiomyocyte differentiation during development via activation of appropriate gene regulatory networks [285], as well as endocardium and second heart field development [286]. Disruptions in this pathway have been associated with defects in cardiac looping and left-to-right animal body patterning defects, including situs inversus, dextrocardia, atrioventricular septal defects, transposition of the great arteries, and double outlet right ventricle [287].

EGF/EFGR signaling is also conserved in *D. melanogaster* development, facilitating, in concert with other signaling pathways, the generation of diverse cardiac cell fates [86]. In vertebrates, EGFR signaling via the Erb-B2 Receptor Tyrosine Kinase (RTK) 2 (ERBB) group mediates diverse functions during cardiac development, including proliferation/growth of cardiac progenitors, valvulogenesis, and regulation of intercellular interactions [288]. As with *D. melanogaster*, in vertebrates, Notch signaling restricts cardiac cell fate [289] via upregulation of *su* (*H*) homologs [290]. Notch signaling pathways in vertebrates allow non-myogenic cell fates [291], including cells of the conduction system, to be generated [292], while experimental upregulation of Notch signaling inhibits cardiomyocyte proliferation [293]. Signaling pathways regulating the derivation of ventral longitudinal muscle from alary muscle, including Heartless and Notch signaling, have been shown to act in a similar manner in mammalian vertebrates, allowing for the derivation of second heart field cardiac progenitors [294,295]. Notch signaling disruptions, at least through mutations in sanpodo and Numb, affect the diversification of cardioblast and pericardial cells [296] (Supplementary Table S5).

#### 4.3.6. Genes Involved in the Formation of the Animal Body Plan During *Drosophila melanogaster* Embryonic Development: Mutations and Phenotypes

Homeodomain or *Hox* genes encode for factors [297] necessary for the proper development and patterning of organisms; they exhibit evolutionary conservation between animal groups, from *D. melanogaster* and *D. rerio* (zebrafish) to mammalian vertebrates and humans. They are generally characterized by the presence of a conserved DNA sequence termed the homeobox sequence, which encodes for a DNA-binding domain in the final protein [298]. Based on phylogenetic classification, there are 11 groups of homeodomain-containing genes in animals [299], namely, ANTP, PRD, LIM, POU, HNF, SINE, TALE, CUT, PROS, ZF, and CERS; the *Hox* gene group is classified within the ANTP group [300]. While *Hox* genes appear in animal groups after the evolutionary divergence of Cnidaria and Bilaterians, they are arranged in chromosome clusters only in Bilaterians. *Hox* gene expression is spatially and temporally regulated and confers different cellular identities depending on their relevant position with regard to the anteroposterior body axis [301,302]. The correlation between placement within the chromosome and position in the animal body where activity from a particular *Hox* gene dominates is conserved as well [303]. *Hox* genes located posteriorly on each chromosome additionally exhibit spatial regulation along the proximodistal axis in vertebrates [301] via histone-modifying protein complexes such as the Trithorax group (TrxG) [304] and Polycomb group (PcG).

In *D. melanogaster*, as in most insects, there are eight *Hox* genes clustered together, albeit split across two different chromosomes [300], comprising the Homeotic Complex (HOM-C) [305,306]. The ANTP Complex (ANT-C) and the Bithorax Complex (BX-C) of *Hox* genes can be recognized; ANT-C contains *Antp* and is involved in the specification of T2 (mesothorax) [307] and A1 [308,309], while BX-C comprises Ultrabithorax (*Ubx*), Abdominal-A (*Abd-A*), and Abdominal-B (*Abd-B*) and is involved in the specification [303] of T3 and A2-A8 [310]. *Abd-A* is also implicated in the specification of cardiac identity [309].

*Abd-A* exhibits the highest expression levels in *tin*+ cardioblasts of A6–A7, the posterior *tin*+ cardioblasts of segment A5, and the *svp*+ cardioblasts in the segment borders of A5/A6, A6/A7, and A7/A8. Lower expression levels are observed in some A5 *tin*+ cardioblasts, as well as in *tin*+ and *svp*+ cardioblasts in A8; a general range of A5–A8 associated with the posterior dorsal vessel (heart chamber) is thus observed [45,47]. *Abd-A* also contributes to alary muscle formation in the posterior dorsal vessel [311]. *Abd-B*, on the other hand, exhibits a general range of A6–A7, with its expression suppressing cardiac morphogenesis and contributing to the formation of a heart terminus (A8) during embryonic development [45,47]. During metamorphosis, *Abd-B* expression is regulated by *Nacα*, a NAC chaperone subunit. This allows for dorsal vessel remodeling during the larval and pupa stages [312], culminating in the eventual histolysis of segments A6–A7 in response to ecdysone secretion [46]. *Ubx* exhibits its highest expression in *tin+* cardioblasts of A3, with lower expression in *svp*+ cardioblasts of the A3/A4 border and *tin*+ cardioblasts of A2 and A5, with even lower expression in segments T3-A1; a general range of T3 to A1–A5 is thus observed [45,47,307,309]. It is also expressed in the alary muscles of the anterior dorsal vessel [307,309]. Finally, *Antp*, along with other homeotic genes of the ANT-C, contributes to the specification of mesothorax (T2) structures, including lymph glands and the Ring gland (T3, A1). It exhibits its highest expression in *tin*+ cardioblasts of A2 and *svp*+ cardioblasts of the A1/A2 border, with lower expression in *tin*+ cardioblasts of T3 and A2 and in *tin*– cardioblasts of A2. *Antp* expression in the posterior dorsal vessel is repressed by *Ubx* [45,307,308].

Null mutations and ectopic expression of homeotic genes of the BX-C and ANT-C groups lead to variable disruptions in the specification of anterior (aorta) and posterior dorsal vessel (heart) identity and heart tube morphogenesis [45,46,47,308,309,311,312,313]. Evolution associated with multiple rounds of duplication and divergence in the ancestral *Hox* gene cluster eventually resulted in the generation of 39 genes in vertebrates. These are arranged into four gene clusters, *HoxA*, *HoxB*, *HoxC*, and *HoxD*, and comprise seven gene families: the anterior *Hox1* and *Hox2* groups; the *Hox3* group; the central *Hox4*, *Hox5*, and *Hox6*–*8* groups; and finally, the posterior *Hox9*–*13* groups [299,314]. Furthermore, as a result of these duplication events, paralogous *Hox* genes can be found at the same relevant locations within each cluster and exhibit some functional equivalence [110]. *Hox* genes may also be implicated in vertebrate cardiac development, including migration of cardiac progenitors via binding of the transcription factor Mesoderm Posterior BHLH Transcription Factor 1 (Mesp1) to *Hoxb1* regulatory sequences [315] and the development of the outflow tract [316]. Congenital heart disease has also been associated with *Hoxa1* gene mutations in both humans [317] and mice [316]. In general, of the ANTP homeotic gene group in vertebrates, anterior *Hox* families such as *Hox1* have been mostly associated with heart defects [318,319,320,321,322,323], with *Hox3* groups mostly associated with carotid artery malformations in mammalian vertebrates [324,325]. Experimental deletion of the *Hoxa*/*Hoxb* clusters in mice results in an atavistic heart phenotype with absence of rightward looping [315]. On the other hand, in *D. melanogaster*, more posteriorly located groups (BX-C) are the ones mainly associated with cardiac development and thus, upon their perturbation, cardiac defects result instead [45,46,47,308,309,311,312,313]. This association is reflected in the localization of the heart chamber in mammalian vertebrates/humans compared to *D. melanogaster* in the animal body plan [303] (Supplementary Table S6).

#### 4.3.7. Genes Involved in Histone Modification During *Drosophila melanogaster* Embryonic Development: Mutations and Phenotypes

As with the transcriptional regulation imposed on *Hox* gene expression, gene transcription differences across different tissues and timepoints, in general, can be established via the action of chromatin-binding and chromatin-modifying factors. Differential histone modifications can often distinguish differentially functioning areas of the genome, with high levels of monomethylation at Lysine 4 of Histone 3 (H3K4me) generally associated with enhancer sequences and high levels of trimethylation at H3K4 associated with active promoter sequences. Apart from H3K4me marks, acetylation at Lysine 27 of Histone 3 (H3K27ac) is also associated with activated enhancer sequences [326]. H3K36 histone marks are also associated with active chromatin [327]. Methylation of H3K4, H3K36, and H3K27 can be carried out by histone-modifying enzymes, including protein complexes associated with SET-containing domain 1 (Set1) (COMPASS) [327,328,329]. The COMPASS series of complexes comprises the core subunits Set1, Trithorax (Trx), and Trithorax-related (Trr). Each of these proteins is the core subunit of a specific COMPASS complex, though all three also share common subunits, including absent, small, or homeotic disks 2 (Ash2); Dpy-30-like 1 (Dpy-30L1); retinoblastoma-binding protein 5 (Rbbp5); and will die slowly (Wds). Other subunits are unique to one specific COMPASS complex, including WD repeat domain 82 (Wdr82) [Set1-COMPASS], Menin 1 (Mnn1) [Trx-COMPASS], and PAX Transcription activation domain-interacting protein (Ptip) [Trr-COMPASS]. Finally, others, such as Host cell factor (Hcf) [Set1, Trx-COMPASS], are found only in specific COMPASS complexes [328]. Experimental knockdown of these subunits in the *D. melanogaster* system leads to variable effects on cardiac structure and function during larval and adult stages, as well as lethality on emergence from the pupal stage, also known as eclosion [35,327,328,330,331].

The proposed mechanism of action for the Set1-, Trx-, and Trr-COMPASS series of complexes during *D. melanogaster* development includes activation of Set1- and Trr-COMPASS during Stages 13 and 14. During this time, cardiac progenitors begin their migration toward the midline. While Set1-COMPASS exhibits steady activity throughout development, Trr-COMPASS is mainly active only during these earlier stages. More specifically, *Trr* exhibits a drop in expression of ~40% during Stage 16 [331]. In later developmental stages [Stages 16–17], cardiac progenitor migration results in a closed heart tube, and the Trx-COMPASS complex, along with Set1-COMPASS, further contributes to completion of heart development [328]. Histone methylation is important for physiologic adult heart function as well, as evident from the dysregulation in structure and function in relevant experiments [35,327,328,330,331]. Many of the above genes have been associated with heart defects in vertebrate models as well, including *Lysine methyltransferase 2C* (*KMT2C*) and *Lysine methyltransferase 2D* (*KMT2D*) (encoding for core subunits of the COMPASS complex series in vertebrates as well) [328], and are associated with defects such as ventricular septal defects, Tetralogy of Fallot [332], and Kabuki syndrome [333]. Kabuki syndrome comprises multiple congenital defects, including distinct facial features, skeletal abnormalities, intellectual disability, and congenital heart defects [334]. DNA methylation may also represent a cause of adult heart dysfunction in vertebrates as well, as upregulation of DNA methyltransferases 1 (*DNMT1*) and 3 (*DNMT3*) can upregulate (Wnt1/β-catenin signaling) or downregulate (pERK1/2 signaling) cellular pathways that promote cardiac fibrosis and heart failure with preserved ejection fraction [335] (Table 2 and Supplementary Table S7). Additional details for each of the genes described in Section 4 and their mammalian/human orthologs can be found in Supplementary Table S8.

**Table 2:** Summary of D. melanogaster genes presented in this review; associated defects observed in the *D. melanogaster* model; corresponding ortholog with the highest DIOPT score, including weighted scores in parentheses; and associations with any congenital heart defects in animal models/humans. In cases where an ortholog cannot be found based on the DIOPT tool, other sources are employed, including the relevant literature. APOB, apolipoprotein B; ASD, atrial septal defect; BAV, bicuspid aortic valve; DORV, double outlet right; Dpp, Decapentaplegic; Dpy-30L1, Dpy-30 like 1; EGFR, epidermal growth factor receptor; EcR, ecdysone receptor; FGF8, fibroblast growth factor 8; FGFR3, fibroblast growth factor receptor 3; MMP2–14, matrix metalloproteinase 2–14; MR, mitral regurgitation; MVP, mitral valve prolapse; PFO, patent foramen ovale; PTA, persistent truncus arteriosus; TIMP3, tissue inhibitor of metalloproteinase 3; TOF, Tetralogy of Fallot; Trr, Trithorax-related; Trx, Trithorax; VSD, ventricular septal defect; VEGF, vascular endothelial growth factor; N/A, not applicable. For a complete list of all gene abbreviations, see Supplementary Table S9.

Gene	*D. melanogaster* Model Defect	Ortholog	DIOPT Score	Vertebrate Model Defect/ Congenital Heart Defect	Study (Reference)
*Abd-A*	*Abd-A* deficiency associated with loss of heart chamber and cardiac cardioblast identity, reduction in posterior dorsal vessel (heart chamber) diameter now similar to the anterior dorsal vessel (aorta), absence of cellular dimorphism between anterior (aorta) and posterior dorsal vessel (heart chamber) with smaller volume cells present throughout	*HOXB6*, *HOXC6*, *HOXA6*	5 (4.87)	Combined deletions in *HOXA*, *HOXB* clusters generally associated with defects in cardiac looping and appearance of primitive/atavistic heart morphologies [110] (mouse)	Lo et al., 2002 [45], Lovato et al., 2002 [47], Ponzielli et al., 2002 [309], Perrin et al., 2004 [308], Ryan et al., 2005 [313], Monier et al., 2005 [46], LaBeau et al., 2009 [311]
				*HOXB6* variants associated with thoracic aortic dissection; *HOXA5*, *HOXB6*, *HOXC6* may correlate with vascular smooth muscle cell de-differentiation in these cases [336] (human)	
	*Abd-A* overexpression/ectopic expression induces a cardiac identity in the anterior dorsal vessel			*HOXA6* has not yet been specifically associated with cardiac development or congenital heart defects [337]	
*Abd-B*	*Abd-B* deficiency associated with increase in posterior dorsal vessel (heart chamber) diameter; increase in cardioblast number with disorganization in their arrangement; and dilation of heart terminus (A6-A8); *Abd-B* deficiency also rescues the *Nacα* KD-induced “No-heart” phenotype	*HOXA10*	6 (6.01)	Combined deletions in *HOXA*, *HOXB* clusters generally associated with defects in cardiac looping and appearance of primitive/atavistic heart morphologies [110] (Mouse)	Lo et al., 2002 [45], Lovato et al., 2002 [47], Perrin et al., 2004 [308], Schroeder et al., 2022 [312]
				*HOXA10* misexpression/overexpression early during embryoid body development restricts specification to a cardiac lineage and impairs differentiation of *NKX2.5* expressing progenitor cells into differentiated cardiomyocytes [338] (in vitro models)	
	*Abd-B* overexpression/ectopic expression associated with suppression of cardiac morphogenesis and myogenesis and defects in somatic muscle formation			*HOXA10* has not yet been specifically associated with congenital heart defects [337]	
*Antp*	*Antp* deficiency associated with mild defects in cardioblast differentiation in segment A1	*HOXA7*, *HOXA1*, *HOXA3*	9 (8.94)	Combined deletions in *HOXA*, *HOXB* clusters generally associated with defects in cardiac looping and appearance of primitive/atavistic heart morphologies [110] (mouse)	Lo et al., 2002 [45], Perrin et al., 2004 [308]
				*HOXA1* mutations associated with defects in brainstem, ventilation, inner ear, and craniofacial morphology, along with cardiac malformations, including TOF, interrupted aortic arch, and aberrant subclavian artery [316] (mouse)	
				*HOXB1* mutations associated with VSD, shorter outflow tract, upregulation of FGF/ERK, BMP/SMAD in the pharyngeal region, premature myocardial differentiation [318] (mouse)	
				*HOXA3* mutations associated with defects in the 3rd pharyngeal artery (carotid artery system), thyroid and parathyroid glands, and carotid body morphology [339] (mouse)	
				*HOXA1* (homozygous mutations) associated with Athabascan Brainstem Dysgenesis, Bosley–Salih–Alorainy Syndrome (defects in brainstem, inner ear, cognitive function, and cardiac malformations) [316,317] (human)	
				*HOXA3* loss due to 5.6 Mb deletion at chromosome 7p15.1–p15.3 associated with defects in facial, hand–foot morphology, supernumerary nipples, hypospadias, and hearing defects; hand–foot and genital defects associated with *HOXA13* deletion in the same locus [340] (human)	
				*HOXA7* has not yet been specifically associated with cardiac development or congenital heart defects [337]	
*apoLpp*	*apoLpp* absence associated with cardiac arrhythmia	*APOB*, *LOC400499*	3 (2.88, 2.82)	*APOB* mutations reduce cardiomyocyte proliferation due to an upregulation of cell cycle inhibitors and pro-apoptotic factors and downregulation of cell cycle genes (in vitro models)	Theis et al., 2020 [230]
				*APOB* mutation associated with a case presenting with cleft lip and palate, DORV, dextrocardia, transposition of the great arteries and hypoplastic right ventricle, along with multisystem defects in the thyroid, nervous system, and eyes though direct association with a causative pathway has been made [341]; maternal dysregulation in lipid profiles; *APOB* expression associated with higher rates of congenital heart defects in offspring (VSD, TOF, pulmonary valve stenosis) [342] (human)	
*Apt*	*Apt* mutations associated with late embryonic/early larval stage lethality, abnormal dorsal vessel morphology with absent cardioblast/pericardial cells	N/A	N/A	N/A	Su et al., 1999 [343], Liu et al., 2014 [344]
*Ash1*	*Ash1* mutations associated with disruption in physiologic heart function, with disruption in actin filament arrangement; reduction in cardiac myofibril density; increase in pericardin (cardiac fibrosis); increase in systolic diameter and heart period; adult lethality (Adult)	*ASH1L*	14 (13.69)	*AH1L* knockdown associated with reduced expression of genes such as *HOXA6*, *HOXA10* [345] (in vitro models)	J. Zhu et al., 2023 [327]
				*ASH1L* variants associated with defects in coronary vascular branching and single left coronary arteries [346,347,348] (human)	
*Ash2*	*Ash2* mutations associated with disruption in physiologic heart function, with disruption in actin filament arrangement; reduction in cardiac myofibril density, cardioblast numbers, and increase in pericardin (cardiac fibrosis); increase in systolic diameter and heart period; adult lethality (adult)	*ASH2L*	17 (16.75)	Absence of *ASH2L* leads to early embryonic lethality; interaction with *TBX1* may act as a modulating factor for DiGeorge-like syndrome phenotypes (craniofacial defects, immune dysfunction and cardiac defects) [349] (mouse)	Zhu et al., 2024 [328]
				*ASH2L* has not yet been directly associated with congenital heart defects in *H. sapiens* (human)	
*bab2*	*Bab2* mutations associated with disruption in the localization of eve+ pericardial cell groups	*BTBD18*	4 (3.91)	*BTBD18* has not yet been associated with congenital heart defects in *H. sapiens* [214] (human)	Junion et al., 2007 [130], Couderc et al., 2002 [128]
*bic*	bic knockdown throughout development associated with reduction in systolic and diastolic diameter (Embryo), ectopic *Abd-B* expression during metamorphosis leading to aberrant histolysis, leading to ‘No-heart’ phenotype with absent pericardin, cardiac cell dispersal, and fat cell accumulation (Pupa, Adult); bicaudal phenotype with embryo developing with a mirror image duplication of the posterior axis (embryo)	*BTF3*, *BTF3L4*	15 (14.87, 14.80)	*BTF3*, *BTF3L4* have not yet been associated with cardiac development or congenital heart defects	Schroeder et al., 2022 [312]
*bifid* (*also known as omb*)	*bifid* mutations associated with embryonic lethality; human *TBX2*, *TBX2-R20Q*, *TBX2-R305H* variants cannot rescue *bifid* mutation phenotypes in *D. melanogaster*	*TBX2*, *TBX3*	12 (11.89, 11.85)	Mutations associated with postnatal lethality with craniofacial defects (double heterozygous loss for *TBX2*, *TBX3*); lack of constriction between left atrium and left ventricle (atrioventricular canal) [211]; atrioventricular canal defects; pericardial edema, defects in palate and limb development (mouse)	Liu et al., 2018 [127]
				*TBX2* variants associated with TOF, single ventricle, single atrium (human)	
				*TBX3* variants associated with TOF and transposition of the great arteries [212] (human)	
*Bre1*	*Bre1* mutations associated with reduction in cardiac myofibril density and adult lethality (adult)	*RNF40*	16 (15.74)	*RNF20*, *RNF40* knockdown results in defects in ciliogenesis at the left–right organizer and as a result in left–right patterning; defects in cardiac looping [350] (frog)	Zhu et al., 2017 [35]
				*RNF20*, *RNF40* deletion (mosaic deletion) results in defects in cardiomyocyte maturation [351] (mouse)	
				*RNF40* variants associated with HLHS [352] (human)	
*Cdc42*	*Cdc42* mutations associated with disruption in myofibril arrangement; disruption in physiologic heart function with increase in diastolic interval; cardiac arrhythmia (adult)	*CDC42*	12 (12)	*CDC42* mutations/loss associated with embryonic lethality, reduced cardiac growth with small ventricles (including right ventricle hypoplasia [353]), and enlarged right atrium; deep apical cleft between adjacent ventricular walls; thin ventricular walls with VSD; reduction in the thickness of compact myocardium; reduced cardiomyocyte proliferation throughout; defects in cardiomyocyte cell-to-cell adhesion; disruption in N-cadherin and β-catenin localization within cardiomyocytes [354]; defects in outflow tract septation and aortic arch patterning; craniofacial defects and thymus aplasia; impairment of normal cardiac neural crest cell migration (regulated by BMP2) [355] (mouse)	Qian et al., 2011 [260], Voglet et al., 2014 [251]
				*CDC42* variants/mutations associated with multisystem congenital defects, including cardiac defects such as VSD, ASD, PDA, and PFO; total anomalous pulmonary venous return; coarctation of the aorta; and pulmonary stenosis [356] (human)	
*CG10585*	*CG10585* mutations associated with disruption in physiologic heart function with increase in systolic and diastolic diameter	*PDSS2*	16 (15.8)	*PDSS2* mutations associated with coQ10 deficiency and defects in the mitochondrial respiratory chains; increase in reactive oxygen species; oxidative stress in some tissues, such as the kidneys, leading to renal failure [357] (mouse)	Schroeder et al., 2019 [233]
				*PDSS2* variants associated with nephrotic syndrome and hypertrophic cardiomyopathy in infants [358]; may contribute to more severe phenotypes in congenital heart defects (human)	
*CG10984*	*CG10984* mutations associated with disruption in myofibril arrangement	*ANKRD12*	9 (8.85)	*ANKRD12* overexpression associated with defects in the sinus venosus; defects in cardiac rotation; anomalous communications between venous and arterial circulations; defects in the fossa ovalis [359] (mouse)	Schroeder et al., 2019 [233]
*CG2658*	*CG2658* mutations associated with disruption in actin filament and myofibril arrangement	*SPG7*	12 (12.06)	Constitutive activation of *SPG7* associated with constitutive activation of a mitochondrial mAAA protease; upregulating ATP and reactive oxygen species production and eventually upregulating cell proliferation [360] (in vitro models)	Schroeder et al., 2019 [233]
				*SPG7* variants associated with atrioventricular canal defects (human)	
*D-mef2*	*D-mef2* loss causes absence of cardiac, somatic, and visceral muscle differentiation	*MEF2C*, *MEF2A*	13 (12.96, 12.86)	*MEF2C*, *MEF2A* mutations (homozygous loss) associated with failure of cardiac looping, failure of right ventricle development (mouse)	Lilly et al., 1995 [239], Hu et al., 2011 [113], Lin et al., 1997 [361]
				DORV [225], VSD [226], PDA [227], pulmonary atresia with VSD [228] (human)	
*Dap160*	*Dap160* mutations associated with minimal effects on actin filament arrangement	*ITSN1*	16 (15.8)	*ITSN1* mutations associated with ASD, 21q deletion syndrome (craniofacial dysmorphias, developmental delay, behavior abnormalities, and various systemic manifestations) [362]; congenital heart defects associated with Down syndrome (partial Trisomy 21 phenotype) [363] (Human)	Schroeder et al., 2019 [233]
*dChchd3*/*6*	*dChchd3*/*6* mutations associated with disruption in physiologic heart function with increase in systolic diameter and systolic dysfunction; cardiac arrhythmia; disruption in cell energy production	*CHCHD3*, *CHCHD6*	11 (6)	*CHCHD3*, *CHCHD6* mutations reduce cardiomyocyte proliferation; rate of oxygen consumption after oligomycin-induced inhibition of ATP synthase; levels of sarcomeric F-actin (in vitro models)	Birker et al., 2023 [237]
				*CHCHD*, *CHCHD6* variants enriched in HLHS (human)	
*dMnM*	*dMnM* mutations associated with variable effects on heart structure with cardiac dilation (mild knockdown) and cardiac constriction (strong knockdown) if knockdown cardiac-specific; reduction in survival of adult animals with defects in locomotion if knockdown muscle-specific	*TTN*	4 (3.81)	*MYOM2*, *TTN* variants associated with TOF (human)	Auxerre-Plantié et al., 2020 [152]
*doc1 doc2 doc3*	*doc* mutations/loss associated with early embryonic lethality	*TBX6*, *TBX2*, *TBX3*	10 (9.88)	*TBX6* associated with defects in mesoderm development, including defects in somite development and skeletal muscle formation [364]; *TBX6* is involved in the pathological cardiac hypertrophy response in adult individuals [365] (mouse)	Han and Olson, 2005 [78]
				Deletion in the genomic locus containing *TBX6* associated with pulmonary atresia with ventricular septal defect, a severe form of TOF [210]	
				Mutations associated with postnatal lethality with craniofacial defects (double heterozygous loss for *TBX2* and *TBX3*); *TBX2* mutations associated with lack of constriction between left atrium and left ventricle (atrioventricular canal) [211]; atrioventricular canal defects and defects in outflow tract septation [208]; pericardial edema; defects in palate and limb development (mouse)	
				*TBX2* variants associated with TOF, single ventricle, single atrium [212] (human)	
				*TBX3* variants associated with TOF and transposition of the great arteries [212] (human)	
*Dpp*	*Dpp* mutations/overexpression associated with expansion of pericardial cells into the ventral region of the dorsal mesoderm with disruption of normal gene marker expression in cardioblast/pericardial cell groups	*BMP2*	12 (11.84)	Loss of *BMP2* leads to reduced cardiac jelly tissue, defects in atrioventricular canal morphogenesis, and loss of atrioventricular canal endocardial cushion cellularization (absent epithelial-to-mesenchymal transition) [366]; DORV; VSD; atrioventricular canal defects [367] (mouse)	Lockwood and Bodmer, 2002 [268], Johnson et al., 2007 [267]
				VSD, ASD, TOF [367] (human)	
*Dpy-30L1*	*Dpy-30L1* mutations associated with disruption in physiologic heart function, with disruption in actin filament arrangement; reduction in cardiac myofibril density, cardioblast numbers, and increase in pericardin (cardiac fibrosis); increase in systolic diameter and heart period; adult lethality (adult)	*DPY30*	10 (9.95)	*DPY30* has not yet been directly associated with congenital heart defects	Zhu et al., 2024 [328]
*Dscam*	*Dscam* associated with variable defects in leading-edge, ranging from reduction in migration velocity and reduction in filopodia per segment to reduction in leading-edge lamellipodial activity (Embryo); overexpression associated with an increase in heart failure rate after electrical-pacing-induced stress (Adult)	*DSCAM*	12 (12.01, 11.84)	*DSCAM* mutations/overexpression due to increased gene dose associated with septal defects in both the perimembranous regions and the muscular regions; defects in the outflow tracts, including failure of outflow tract septation into pulmonary arterial and aortic trunks, DORV, and defects in atrioventricular canal morphogenesis and atrioventricular canal defects; atrial and atrioventricular canal defects may be due to defects in the myocardial tissue that contributes to their development, along with loss of WNT signaling that downregulates cardiac mesoderm progenitor proliferation in the inflow tract [368] (mouse)	Grossman et al., 2011 [369], Raza and Jacobs, 2016 [370]
				*DSCAM* variants/overexpression due to increased gene dose associated with the emergence of congenital heart defects associated with Down syndrome (VSD, ASD, atrioventricular canal defects, TOF, PDA) [371] (human)	
*EcR*	*EcR* mutations associated with inhibition of cardiac remodeling in the posterior dorsal vessel (heart chamber); Dorsal vessel maintains larval morphology with absence of histolysis in segments A6-A7; and absence of remodeling in *Abd-A*+ cardioblasts	*NR1H2*, *NR1H3*	12 (11.88, 11.7)	*NR1H2*, *NR1H3* have not yet been associated with cardiac development or congenital heart defects	Monier et al., 2005 [46]
*Egfr*	*Egfr* mutations associated with disruption of relative cardioblast/pericardial cell subpopulations with reduction in generic cardioblast populations and increase in ostial cardioblast populations	*ERBB4*	13 (12.87)	*ERBB4* mutations associated with embryonic lethality; cardiac defects including reduced trabeculation (hypotrabeculation) with thin myocardial walls and defects in endocardial cushion formation [372]; dysregulation of valve mesenchyme proliferation [373] (Mouse)	Schwarz et al., 2018 [86]
				*ERBB4* variants associated with defects in the development of the left ventricular outflow tract, including aortic stenosis, HLHS [374], and HRHS [375]; coarctation of the aorta [374]; increased rate of bioprosthetic aortic valve stenosis associated with local foreign tissue reaction [376] (human)	
*Eve*	*Eve* mutations associated with reduction in pericardial cell populations	*EVX2*	10 (10.04)	*EVX2* mutations associated with defects in limb development, although they have not yet been associated with congenital heart defects [213,214] (human)	Fujioka et al., 2005 [189]
*fz*	fz mutations associated with defects in endoderm (midgut), mesoderm, and ectoderm (cuticle, wings, wing imaginal disks); absence of cardiac development if both *fz* and *Dfz2*	*FZD1*, *FZD2*	15 (14.87)	*FZD* mutations associated with multiple effects during development, including neural tube defects [377] (frog)	Bhanot et al., 1999 [275], Chen and Struhl, 1999 [276]
				*FZD1*, *FZD2* mutations associated with defects in palate closure, ventricular septum, correct position of the outflow tract, neural tube defects, and inner ear defects [378] (mouse)	
				*FZD1*, *FZD2* have not yet been directly associated with congenital heart defects in *H. sapiens* (human)	
*Gart*	*Gart* mutations associated with minimal effects on actin filament arrangement, with disruption in myofibril arrangement	*GART*	17 (16.75)	*GART* mutations associated with ASD, 21q deletion syndrome (craniofacial dysmorphias, developmental delay, behavior abnormalities, and various systemic manifestations) [362]; congenital heart defects associated with Down syndrome [379] (human)	Schroeder et al., 2019 [233]
*GGPPS*	*GGPPS* mutations and relevant pathway protein mutations associated with “Broken-hearted” phenotype with dissociation of cardioblast/pericardial cell adhesion; embryonic lethality	*GGPS1*	16 (15.72)	*GGPS1* mutations may be a cause of reduction in GGPP, in turn leading to reduced binding affinity of Rho GTPases for GTP, disrupt their localization below the plasma membrane, leading to vascular destabilization and the progressive dilatation and rupture of cerebral vessels [380] (zebrafish)	Yi et al., 2006 [229]
				Infantile hemangioma [381]; cerebral cavernous malformations [382] due to disruption in the mevalonate pathway (human)	
*Gia*	*Gia* mutations associated with “Broken-hearted” phenotype with dissociation between cardioblast/pericardial cells, disruption in cell-to-cell adhesion protein distribution, and disruption in cardioblast alignment; late embryonic/early larval stage lethality	*ADGRF3*, *ADGRF4*, *ADGRD1*, *ADGRE2*, *ADGRG3*, *ADGRG6*, *ADGRL1*, *ADGRG7*, *ADGRF5*, *ADGRD2*, *ADGRG2*, *ADGRE1*, *ADGRE5*, *ADGRG4*, *ADGRL4*, *ADGRL2*, *ADGRE3*, *ADGRL3*	2 (1.81)	*ADGRG6* mutations secondary to placental defects; global inactivation of *ADGRG6* associated with embryonic lethality and ventricular myocardium thinning, with no effect on heart patterning or myocardium maturation [383] (mouse)	Patel et al., 2016 [384]
				*ADGRG6* mutations secondary to placental defects; mutations in *ADGRG6* have no effect on cardiac development [383] (zebrafish)	
				Combined *ADGRF5*, *ADGRL4* mutations associated with DORV; outflow tract malformations; and aortic arch artery defects, including double aortic arch, embryonic lethality, postnatal renal thrombotic microangiopathy, hemolysis, and splenomegaly [385] (mouse)	
				*ADGRL2* mutations/loss associated with defects in vascular remodeling [386] (zebrafish) (mouse)	
				*ADGRF3*, *ADGRF4*, *ADGRD1*, *ADGRE2*, *ADGRG3*, *ADGRL1*, *ADGRG7*, *ADGRD2*, *ADGRG2*, *ADGRE1*, *ADGRE5*, *ADGRG4*, *ADGRL4*, *ADGRE3*, and *ADGRL3* have not yet been associated with cardiac development or congenital heart defects; *ADGRF4* associated with enamel mineralization [387]; *ADGRL1* implicated in neurodevelopmental disorders [388]; *ADGRG7* implicated in familial endometriosis [389]; *ADGRG2* implicated in congenital bilateral absence of the vas deferens [390]; *ADGRL4* involved in vascular remodeling during development [385]; *ADGRL3* involved in neurogenesis [391]	
*H15* (*nmr1*)	*H15* mutations associated with disruption in cardioblast/pericardial cell diversification divisions; mild cardiac defects	*TBX20*	11 (10.73)	*TBX20* mutations associated with hypoplasia in the outflow tract and right ventricle (complete knockdown), lack of septation in the outflow tract with PTA, right ventricle hypoplasia, valve defects [204] (mouse)	Reim et al., 2005 [198], Hu et al., 2011 [113]
				*TBX20* mutations associated with DORV, VSD, ASD, TOF, PTA, PFO, BAV [205], MVP/MR, total anomalous pulmonary venous connection, and congenital atrioventricular block [203]; HLHS [205] (human)	
*Hand*	*Hand* mutations/knockout associated with hypoplastic dorsal vessel with reduction in wall thickness; late embryonic/early larval lethality	*HAND2*	15 (14.74)	*HAND2* mutations/absence associated with early embryonic lethality; valve defects, such as tricuspid atresia; double inlet left ventricle; hypoplastic myocardial tissue; rightward shift of the interventricular septum with larger left and smaller right ventricle; hypotrabeculated myocardial tissue with multiple interventricular septa; hypervascularization with multiple coronary arteries [219] (mouse)	Han et al., 2006 [191], Lo et al., 2007 [190]
				*HAND2* variants associated with DORV, VSD, and outflow tract malformations [214]; pulmonary stenosis [224] (human)	
*Hcf*	*Hcf* mutations associated with disruption in physiologic heart function, with disruption in actin filament arrangement; reduction in diastolic diameter and heart rate; adult lethality (adult)	*HCFC1*, *HCFC2*	8 (8.14, 7.98)	*HCFC1* mutations lead to defects in craniofacial development; no evidence of a pathologic cardiac phenotype [392] (zebrafish)	Huang et al., 2022 [330]
				*HCFC1* mutations associated with X-linked form of combined methylmalonic acidemia and hyperhomocysteinemia [393]; *HCFC1* has not yet been associated with congenital heart defects	
				*HCFC2* has not yet been associated with cardiac development or congenital heart defects	
*Hd*	*Hd* mutations associated with disruption in actin filament and myofibril arrangement	*DONSON*	15 (14.77)	*DONSON* mutations associated with ASD, 21q deletion syndrome (craniofacial dysmorphias, developmental delay, behavior abnormalities, and various systemic manifestations) [233,362]; microcephaly; and short stature [394] (human)	Schroeder et al., 2019 [233]
*Hh*	*Hh* mutations associated with variable effects on cardiac development ranging from reduction in cardiac cell numbers and no dorsal vessel formation to no effect on dorsal vessel formation, depending on timing of gene mutation	*SHH*	15 (14.79)	*SHH* protein mutations/*SHH*-related signaling pathway mutations associated with heart defects related to the establishment of left–right asymmetry due to dysfunction of midline structures [286], including situs inversus, dextrocardia, defects in pharyngeal arch patterning, atrioventricular septal defects, transposition of the great arteries, and DORV [287] (mouse)	Park et al., 1996 [176], Liu et al., 2006 [284]
				Possible association with TOF and 22q11.2DS deletion syndromes [395] (human)	
*HMGCR*	*HMGCR* mutations associated with “Broken-hearted” phenotype with dissociation of cardioblast/pericardial cell adhesion; embryonic lethality	*HMGCR*	15 (14.77)	Inhibition of the HMGCR pathway leads to vascular destabilization and the progressive dilatation and rupture of cerebral vessels [380] (zebrafish)	Yi et al., 2006 [229]
				*HMGCR* mutations associated with infantile hemangioma [381] and cerebral cavernous malformations [382] due to disruption in the mevalonate pathway (human)	
*Htl*	*Htl* mutations associated with defects in mesoderm migration alongside ectoderm with absence of visceral mesoderm (embryo)	*FGFR3*	15 (14.72)	*FGFR3* deficiency affects bone development during postnatal growth [396]; disrupts FGF8-mediated migration of cardiac Neural crest cells (mouse)	Kadam et al., 2009 [261], Dorey and Amaya, 2010 [262]
				*FGFR3* mutations associated with achondroplasia with associated cardiovascular defects in 20% of patients from a patient cohort of 37, including VSD, ASD, pulmonary stenosis, and coarctation of the aorta [397] (human)	
				*FGFR2B* mutations associated with ventricular septal defects, disruption in outflow tract alignment, poor ventricular trabeculation [398], and fewer epicardial-derived cells in the compact myocardium due to impaired movement of cardiac fibroblasts within the myocardium during development [399] (mouse)	
				*FGFR2B* mutations/variants have not yet been directly associated with congenital heart defects in *H. sapiens* (human)	
*Jarid2*	*Jarid2* mutations associated with increased levels of pericardin (cardiac fibrosis); embryonic lethality	*JARID2*	14 (13.79)	*JARID2* deficiency associated with increased ventricular trabeculation and non-compaction of the ventricular wall [400] (mouse)	Basu et al., 2017 [232]
				*JARID2* mutations have not yet been directly associated with congenital heart defects; *JARID2* variants associated with a distinct neurodevelopmental syndrome [401] (human)	
*Kif1A*	*Kif1A* deficiency shows no effect on dorsal vessel structure or function	*KIF1A*	15 (14.79)	*KIF1A* variants identified in left-sided heart defects, HLHS (human)	Akasaka et al., 2020 [236]
	*Kif1A* overexpression associated with disruption in myofibrillar arrangement, fewer valves, and increased collagen deposition (cardiac fibrosis)				
*Kismet*	*Kismet* mutations associated with disruption in physiologic heart function with reduction in cardiac myofibril density and increase in Prc (cardiac fibrosis) (larva); reduction in cardiac myofibril, cardioblast numbers, and increase in pericardin (cardiac fibrosis); adult lethality (adult)	*CHD7*	12 (11.85)	*CHD7 mutations* associated with defects in truncus arteriosus and outflow tract positioning due to defects in cardiac neural crest cell function [402] (frog)	Zhu et al., 2017 [35]
				*CHD7* mutations associated with CHARGE-like syndrome phenotype (vestibular dysfunction, heart defects) and hypoplastic pharyngeal arch arteries [403] (heterozygous loss of *CHD7*) (mouse)	
				*CHD7* variants associated with ASD [214] and CHARGE syndrome (otolith defects, coloboma; craniofacial malformations; and heart defects, such as VSD, ASD, conotruncal defects, and defects in endocardial cushion development) [403] (human)	
*Kuz*	*Kuz* mutations associated with variable defects, ranging from rudimentary/missing heart, disruption in cardioblast alignment, and disorganized heart with disruption in cardioblast alignment to hyperplastic heart with increase in all cardioblast populations and reduction in some pericardial cell groups and lymph gland cells	*ADAM10*, *ADAM17*	14 (13.89)	*ADAM10* disruption in endothelial cells associated with early embryonic death, impaired *SNAIL*, *BMP2* expression in cardiac tissues, and *NOTCH1*-like phenotype, including impaired vascular morphogenesis with reduction in aortic and cardinal vein size, impaired epithelial-to-mesenchymal transition, and defects in ventricular trabeculation [404]; defects in differentiation of coronary artery endothelial cells with enlarged heart and defects in myocardial compaction, upregulation of venous, and immature endothelial markers [405] (mouse)	Albrecht et al., 2006 [406]
				ADAM17 variants associated with the right ventricular hypertrophy in TOF due to possible effects on HB-EGF/ErbB signaling [407] (human)	
				*ADAM10* has not yet been associated with congenital heart defects, possibly due to the embryonic lethality of *ADAM10* mutations [407]	
*lanA*	*lanA* mutations associated with complete cardioblast/pericardial cell dissociation with random migration patterns in animals with *scb*, *lanA* mutations	*LAMA5*	14 (13.87)	*LAMA5* has not yet been directly associated with congenital heart defects; *LAMA5* variants associated with a systemic developmental syndrome characterized by glomerulopathy [408]	Stark et al., 1997 [409], Nishiyama et al., 2005 [410]
*Lid*	*Lid* mutations associated with adult lethality (Adult)	*KDM5A*	17 (16.75)	Inhibition of *KDM5A* shifts cardiac progenitors toward the mature stage via upregulation of genes associated with oxidative phosphorylation, fatty acid oxidation, and sarcomere organization [411] (in vitro models)	Zhu et al., 2017 [35]
				*KDM5A* variants associated with VSD, TOF, and patent foramen ovale [412] (human)	
*Lpt*	*Lpt* mutations associated with “Broken-hearted” phenotype (embryo); disruption in physiologic heart function, with disruption in actin filament arrangement; reduction in cardiac myofibril density, cardioblast numbers, and increase in pericardin (cardiac fibrosis); reduction in diastolic diameter and heart rate (adult); late embryonic/early larval stage lethality; adult lethality	*KMT2D*, *KMT2C*	8 (7.89)	*KMT2D* mutations associated with mild aortic narrowing (heterozygous loss), embryonic lethality, absence of somites, headfolds (homozygous loss), embryonic lethality, disorganized interventricular septum, and absence of outflow tract septation into aorta/pulmonary artery (conditional deletion in cardiac tissues only) [333] (mouse)	Huang et al., 2022 [330]
				*KMT2D* variants associated with VSD, ASD, obstructive lesions [214], Kabuki Syndrome [413], and HLHS [414] (human)	
				*KMT2C* has not yet been associated with cardiac development or congenital heart defects; *KMT2C* variants/deletion associated with Kleefstra 2 syndrome [415] and a neurodevelopmental syndrome distinct from Kleefstra and Kabuki syndrome [416]	
*mgl*	*mgl* mutations associated with cardiac dilation with increased end diastolic diameter and cardiac arrhythmia	*LRP2*	14 (14.01)	*LRP2* mutations reduce cardiomyocyte proliferation due to an upregulation of cell cycle inhibitors, pro-apoptotic factors, and downregulation of cell cycle genes (in vitro models)	Theis et al., 2020 [230], Riedel et al., 2011 [231]
				*LRP2* mutations associated with hypoplastic heart phenotype with reduction in ventricular cardiomyocyte numbers and reduced ventricular dimensions, with an associated reduction in contractility and bradycardia (zebrafish)	
				*LRP2* variants enriched 3-fold in patients with HLHS compared to healthy controls (10% compared to 3.4%) (human)	
*mid (nmr2)*	*mid* mutations associated with disruption in physiological cardiac function	*TBX20*	13 (12.78)	*TBX20* mutations associated with hypoplasia in the outflow tract, right ventricle (complete knockdown), lack of septation in the outflow tract (PTA), right ventricle hypoplasia, and valve defects [204] (mouse)	Reim et al., 2005 [198], Hu et al., 2011 [113]
				*TBX20* variants associated with DORV, VSD, ASD, TOF, PTA, PFO, BAV [205], MVP/MR, total anomalous pulmonary venous connection, congenital atrioventricular block [203], and HLHS [205] (human)	
*mmp1*	*mmp1* mutations associated with disruption in cardioblast arrangement, cardiac lumen formation with reduced diameter, or absence of cardiac lumen formation; absence of cardioblast shape changes/filopodia and variable defects in leading-edge ranging from reduction in migration velocity and number of filopodia per segment to leading-edge lamellipodial activity (embryo)	*MMP14*, *MMP2*	12 (11.9)	*MMP2* mutations between the primitive streak stage and the 14 somite stages associated with failure of heart tube formation, variations of the “cardia–bifida” phenotype, alterations in looping direction within cells proliferating in the dorsal mesocardium and anterior heart field, and failure of heart tube bending in later stages [417] (chicken)	Raza et al., 2017 [253], Hughes et al., 2020 [254]
				*MMP14* mutations associated with death in the early postnatal period and defects in skeleton, skeletal muscle, and lung development [418] (mouse)	
	*mmp1* overexpression associated with “cardia–bifida” phenotype with disruption in adhesion junction and myofibril arrangement; incomplete dorsal vessel; luminal and abluminal Viking plaques (Embryo)			*MMP2*, *MMP9*, *MMP14* associated with unicommissural aortic valves characterized by congenital fusion of adjacent cusps of two commissures [419] (human)	
*mmp2*	*mmp2* mutations associated with disruption in cardioblast arrangement, cardiac lumen formation with absence of cardiac lumen formation, absence of cardioblast shape changes/filopodia, and variable defects in leading-edge ranging from reduction in migration velocity, and number of filopodia per segment and leading-edge lamellipodial activity (embryo)	*MMP15*, *MMP9*	10 (10)	*Snail1* mutations reduce/downregulate levels of *MMP15*; reduce cell migration; and, due to *Snail1* deficiency, cellularity in atrioventricular endocardial cushions [420] (mouse)	Raza et al., 2017 [253], Hughes et al., 2020 [254]
				*MMP15* variants associated with congenital heart defects, cholestasis, and dysmorphism [421] (human)	
	*mmp2* overexpression associated with “Cardia-Bifida” phenotype with midline tearing, incomplete dorsal vessel, and luminal and abluminal Viking plaques (Embryo)			Elevated MMP9 expression contributes to extracellular matrix degradation, activates a proteinase-activated receptor-1 signaling cascade, and contributes to cardiomyocyte dysfunction and heart failure in single ventricle cases [422]; *MMP2*, *MMP9*, *MMP14* variants associated with unicommissural aortic valves characterized by congenital fusion of adjacent cusps of two commissures [419]; *MMP9* variants associated with ascending aortic aneurysm, thoracic aortic dissection [423] and aortic stenosis [424] (human)	
*Mnn1*	*Mnn1* mutations associated with disruption in physiologic heart function, with disruption in actin filament arrangement; reduction in cardiac myofibril density and cardioblast numbers and increase in pericardin (cardiac fibrosis); increase in systolic diameter and reduction in diastolic diameter; adult lethality (adult)	*MEN1*	14 (13.86)	*MEN1* mutations associated with reduced growth during embryonic development with body hemorrhages; defects in neural tube development [425] (mouse)	Zhu et al., 2024 [328]
				*MEN1* has not yet been directly associated with congenital heart defects in *H. sapiens* (human)	
*msh-2*	*msh-2* knockout associated with absence of visceral muscle and absence of dorsal vessel	*MSX2*, *MSX1*	16 (15.8)	*MSX2* mutations/knockout associated with reduced accumulation of second heart field (SHF) precursors to the developing outflow tract; increased accumulation of mesenchymal precursors in the conotruncal endocardial cushions disrupts rotation of the truncus arteriosus and leads to alignment defects in the outflow tract [180]; *MSX2*/*MSX1* mutations associated with defects in cardiac neural crest cell development and associated structures [426] (mouse)	Bodmer et al., 2011 [179], Hu et al.,2011 [113]
				*MSX1* variants associated with VSD [222] (human)	
				*MSX2* mutations associated with craniosynostosis [427], complex heart defect (dextrocardia, dextroversion, PFO) cases with radial agenesis, along with other characteristics of Hunter–McAlpine syndrome (intellectual disability, craniofacial and skeletal abnormalities, and characteristic facial attributes) [223] (human)	
*Nacα*	*Nacα* knockdown throughout development associated with reduction in systolic and diastolic diameter (embryo), ectopic *Abd-B* expression during metamorphosis leading to aberrant histolysis, leading to “No-heart” phenotype with absent pericardin, cardiac cell dispersal, and fat cell accumulation (pupa, adult); bicaudal phenotype with embryo developing with a mirror image duplication of the posterior axis (embryo)	*NACA*		Loss of *NACA* disrupts skeletal muscle development, including myofibrillar organization, paralysis with little muscle contraction, disorganization in thick, and thin myosin filaments [428]; disruption in hematopoietic niche function with defects in hematopoiesis [429] (zebrafish)	Schroeder et al., 2022 [312]
				*NACA* variants associated with TOF [430] (human)	
*Netrin* (*netA*/*netB*)	Netrin mutations associated with variable defects in leading-edge, ranging from reduction in migration velocity and reduction in filopodia per segment to reduction in leading-edge lamellipodial activity	*NTN1*	14 (13.77)	*NTN1* mutations/loss associated with defects in aortic arch artery formation and defects in guidance in developing vasculature abnormal thyroid morphogenesis due to defects in vascular development [431] (zebrafish)	Raza and Jacobs, 2016 [370]
				*NTN1* mutations associated with embryonic lethality (global loss) and increase in interventricular septum thickness with no overt cardiac phenotype (cardiomyocyte-specific loss) [432] (mouse)	
				*NTN1* variants associated with a case presenting with VSD, ASD, and PDA and congenital hypothyroidism due to thyroid dysgenesis [431] (human)	
*Notch*	*Notch* mutations associated with increased levels of pericardin (cardiac fibrosis), reduced levels of cell actin, and embryonic lethality	*NOTCH1*, *NOTCH2*, *NOTCH3*	12 (11.91, 11.77, 11.67)	*NOTCH1* variants (heterozygous mutations) associated with progressive aortic valve calcification due to release of inhibition in osteogenic and pro-inflammatory pathways due to differential histone acetylation at H3K27 *NOTCH1* enhancers [433] (in vitro models)	Basu et al., 2017 [232]
				*NOTCH3* mutations lead to mild defects only, while combined *NOTCH2/NOTCH3* mutations lead to severe vascular defects and embryonic lethality [434] (mouse)	
				*NOTCH1* mutations associated with VSD [214], TOF, BAV, HLHS, various septal defects, and functional single ventricles [214]; Adams–Oliver syndrome (scalp defects and vascular abnormalities) [435]; obstructive lesions [214] (human)	
				*NOTCH2* mutations associated with ASD, malformation of the outflow tracts, obstructive lesions [214], and Alagille syndrome (multisystem disorder with heart defects) (human)	
				*NOTCH3* mutations associated with cerebral arteriopathy with subcortical infarcts and leukoencephalopathy [436] (human)	
*Numb*	*Numb* mutations associated with disruption in myofibril arrangement; reduced levels of cell actin; disruption in diversification of cardioblast cell groups and tin+ cardioblast alignment; embryonic lethality	*NUMB*	12 (11.93)	*NUMB* mutations associated with defects in differentiation of second heart field (SHF) progenitors, upregulation of Notch signaling, defects in cardiomyocyte proliferation, outflow tract and atrioventricular canal septation, and embryonic lethality (loss of both *NUMB* and *NUMBL*) [437] (mouse)	Basu et al., 2017 [232], Gajewski et al., 2000 [296]
				*NUMB* variants associated with cases of heterotaxy/dextrocardia and additional congenital heart defects, including DORV, VSD, pulmonary stenosis, superior–inferior ventricle, left superior vena cava [438] (human)	
*Org-1*	*Org-1* mutations/knockouts associated with severe defects/absence of Alary muscles, thoracic alary-related muscles, and ventral longitudinal muscle	*TBX1*	12 (11.8)	*TBX1* mutations associated with PTA and reduced ability to form brachiomeric muscles (homozygous loss) [439] (mouse)	Schaub et al., 2012 [241], Boukhatmi et al., 2014 [238]
				*TBX1* mutations associated with phenocopy of the 22q11.2DS deletion syndrome with cardiac outflow tract defects (DiGeorge syndrome) (craniofacial defects, immune dysfunction, and cardiac defects) with cardiac outflow tract defects, reduced proliferation of second heart field progenitors (SHF), and aortic arch patterning defects [439] (human)	
*pnr*	*pnr* mutations associated with disruption in specification of cardioblast cell groups	*GATA4*	12 (11.8)	*GATA4* mutations associated with early embryonic lethality due to defects in the extraembryonic endoderm, cardiac bifida, and absence of fusion in the midline; absence of proepicardium; hypoplastic ventricular tissue [440]; valve defects (mouse)	Han and Olson, 2005 [78]
				*GATA4* variants associated with DORV, double-inlet left ventricle, VSD, ASD, atrioventricular septal defect, TOF, and BAV [440]; defects in outflow tract alignment, dextrocardia, and pulmonary stenosis [195] (human)	
*Ptip*	*Ptip* mutations associated with disruption in physiologic heart function, with disruption in actin filament arrangement, reduction in cardiac myofibril density, and cardioblast numbers and increase in pericardin (cardiac fibrosis); increase in systolic diameter and reduction in diastolic diameter; adult lethality (adult)	*PAXIP1*	11 (10.8)	*PAXIP1* mutations associated with early embryonic lethality (mouse)	Zhu et al., 2024 [328]
				*PAXIP1* variants associated with BAV [441] (human)	
*pygo*	*pygo* mutations associated with absence of cardiac valve cell differentiation with lack of high-density myofibrils; absence of physiological posterior dorsal vessel (heart chamber) wall thickening in the valve region; loss of normal heart chamber constriction at valve site (valve site dilation)	*PYGO2*	7 (7.01)	Combined loss of *PYGO1*, *PYGO2* leads to defects in cardiac development after gastrulation including cardiac edema, craniofacial defects, and defects/dysregulation in swimbladder inflation [442] (zebrafish)	Tang et al., 2014 [55]
				Combined loss of *PYGO1*, *PYGO2* leads to embryonic lethality between E13.5 and E4.5, severe defects between E10.5 and E14.5 with hypoplastic ventricular myocardial tissue, atrial dilation, smaller and thinner atrioventricular valves, defects in chamber septation, and defects in outflow tract development, including transposition of the great arteries, hypoplastic aorta, hypoplastic pulmonary artery [442] (mouse)	
				*PYGO1*, *PYGO2* have not yet been specifically associated with congenital heart defects in *H. Sapiens* [442] (Human)	
*pyr*	*pyr* mutations/absence associated with defects in mesoderm migration alongside ectoderm, mesoderm aberrant with multilayer formation, severe defects in dorsal mesoderm specification, reduction/absence of *eve+* groups (embryo)	*FGF8*	--	*FGF8* mutations associated with absence of endoderm and embryonic mesoderm, embryonic lethality during gastrulation; defects in cardiac looping, development of the outflow tract, anterior heart field and survival of cardiac neural crest cells as they migrate toward the outflow tract leading to outflow tract septation defects [443] (mouse)	Kadam et al., 2009 [261], Dorey and Amaya, 2010 [262]
				*FGF8* mutations associated with 22q11.2DS deletion syndrome (craniofacial defects, immune dysfunction, and cardiac defects) phenotypes [444] (human)	
*Rbbp5*	*Rbbp4* mutations associated with disruption in physiologic heart function, with disruption in actin filament arrangement; reduction in cardiac myofibril density and cardioblast numbers and increase in pericardin (cardiac fibrosis); increase in systolic diameter and heart period, adult lethality (adult)	*RBBP5*	16 (15.8)	Increased RBBP4 expression due to loss of c-Jun regulation increases H3K4 methylation at cardiogenic genes, upregulates cardiomyocyte generation [445] (in vitro models)	Zhu et al., 2024 [328]
				*RBBP4* variants in the 1p35 locus associated with ASD, characterized as a risk modifier for Down syndrome [446] (human)	
*Robo*	*Robo* mutations associated with varying effects ranging from no effect on cardioblast migration with defects ranging and mild effects on midline cardioblast alignment to severe effects (gaps, intercalation, and double rows) with *Robo*/*Robo2* mutations	*ROBO3*, *ROBO1*, *ROBO2*	10 (9.68, 9.67, 9.62)	*ROBO1*/*ROBO2* mutations/loss associated with defects in the membranous ventricular septum, thickened and immature semilunar and atrioventricular valves, bicuspid aortic cushions with BAV, downregulation of NOTCH and HEY/HES downstream effectors leading to downregulation in NOTCH signaling [447], partial absence of the pericardium with severe reduction in sinus horn myocardium, hypoplastic caval veins, persistent left inferior caval vein [448], and complete absence of SLIT2 and SLIT3 binding (mouse)	Qian et al., 2005b [77], MacMullin and Jacobs, 2006 [449], Medioni et al., 2008 [246], Santiago-Martínez et al., 2008 [249], Zmojdzian et al., 2008 [57], Zmojdzian et al., 2018 [56], Raza and Jacobs, 2016 [370]
				*ROBO1* mutations/loss associated with defects in the membranous ventricular septum, downregulation of NOTCH and HEY/HES downstream effectors leading to downregulation in NOTCH signaling [447], partial absence of the pericardium [448], and absence of SLIT3 binding [448] (mouse)	
				*ROBO2* mutations alone are not associated with defects in a murine cardiac development model of SLIT/ROBO signaling [448] (mouse)	
				*ROBO1* variants associated with VSD (both in the membranous and muscular septum), ASD [450], malformation of the outflow tracts [214], TOF [451], BAV [452], overriding aorta, defects in canal veins [450], ascending aortic aneurysm [453] (human)	
				*ROBO2* variants associated with cardiac malformations in a case presenting with neurodevelopmental delay and multisystem defects due to del(3)(p12.3p14.1) (3p interstitial deletion) encompassing 31 open reading frames [454], BAV [453] (human)	
*Robo2*	*Robo2* mutations associated with variable defects in dorsal closure and dorsal vessel (delayed migration, gaps, blisters, twists, and midline crossing of cardiac progenitors); highest phenotype severity with *sli*/*scb*, *Robo2* mutations	*ROBO1*, *ROBO3*	9 (8.77, 8.68)	*ROBO3* variants associated with TOF, BAV, and coarctation of the aorta [453] (human)	
*RpL13*	*RpL13* mutations associated with “No-heart” phenotype with complete absence of dorsal vessel and constrictions in posterior dorsal vessel remnants	*RPL13*	16 (15.8)	*RPL13* mutations associated with downregulation of genes related to cell cycle progression (particularly during the S and G2 phases) and cardiac progenitor, cardiomyocyte proliferation; disproportionate increase in fibroblasts compared to cardiomyocytes (in vitro models)	Schroeder et al., 2019 [233]
				*RPL13* variants associated with complete atrioventricular canal defect [455] (human)	
*RpL14*	*RpL14* mutations associated with “Minute” syndrome with impaired development, fertility, and cardiac function; partial dorsal vessel atrophy with reduced levels of pericardin	*RPL14*	16 (15.8)	*RPL14* mutations associated with “Minute”-like phenotype (impaired development, fertility, and cardiac function) (zebrafish)	Nim et al., 2021 [151]
				*RPL14 has* not yet been associated with congenital heart defects in *H. sapiens* [151] (human)	
*Rpn8*	*Rpn8* mutations associated with partial dorsal vessel atrophy	*PSMD7*	16 (15.79)	*PSMD12* variants associated with Stankiewicz–Isidor syndrome (neurodevelopmental defects, cardiac defects) (human)	Nim et al., 2021 [151]
*RpS24*	*RpS24* mutations associated with “Minute” Syndrome with impaired development, fertility and cardiac function; complete dorsal vessel atrophy with increased levels of pericardin (cardiac fibrosis), and visible breaks in dorsal vessel structure	*RPS24*	16 (15.8)	*RPS24* mutations associated with the congenital heart defects presenting with Diamond Blackfan Anemia (”Minute”-like phenotype with impaired growth, bone marrow function, and congenital heart defects) [151] (human)	Nim et al., 2021 [151]
*Scny*	*Scny* mutations associated with reduction in cardiac myofibril density and adult lethality (adult)	*USP36*	11 (10.89)	USP36 variants associated with coronary artery structural variants and an increased risk of coronary artery disease [456] (human)	Zhu et al., 2017 [35]
*Set1*	*Set1* mutations associated with disruption in physiologic heart function, with disruption in actin filament arrangement; reduction in cardiac myofibril density and cardioblast numbers and increase in pericardin (cardiac fibrosis); increase in heart period; increased lethality; metabolic dysregulation (upregulation of carbohydrate metabolism genes, downregulation of lipid metabolism genes) (adult)	*SETD1A*	12 (11.85)	*SETD1A* associated with a case of airway defects, characteristic facies and body features, along with congenital heart defects, including ASD and pulmonary hypertension [457] (human)	J. Zhu et al., 2023 [327]
*Set2*	*Set1* mutations associated with disruption in physiologic heart function, with disruption in actin filament arrangement; reduction in cardiac myofibril density; increase in pericardin (cardiac fibrosis); increase in heart period; adult lethality (adult)	*SETD2*	13 (12.64)	*SETD2* mutations associated with defects in coronary vascular development with greater effects on left ventricular coronary vasculature, ventricular non-compaction, and embryonic lethality mid-gestation; no effects on other peripheral vasculature [458] (mouse)	J. Zhu et al., 2023 [327]
				*SETD2* has not yet been associated with congenital heart defects in *H. sapiens* [413] (human)	
*Shg*	*Shg* mutations/loss associated with absence of cardiac lumen formation with extracellular space accumulating between contralateral cardioblasts	*CELSR1*, *CELSR3*, *CELSR2*	2 (2.01, 2.01, 2.01)	*CELSR1* mutations associated with anteroposterior axis shortening due to defects in convergence and extension during zebrafish embryonic development, neural tube defects, enlarged pericardium [459] (zebrafish)	Santiago-Martínez et al., 2008 [249], Zmojdzian et al., 2008 [57], Zmojdzian et al., 2018 [56]
				*CELSR1*, *CELSR2*, *CELSR3* variants associated with neural tube defects and congenital heart defects, including DORV, VSD, ASD, PDA, and pulmonary stenosis, and aortic stenosis [459] (human)	
*Sli*	*Sli* mutations associated with variable defects in dorsal closure and dorsal vessel (delayed migration, gaps, blisters, twists, and midline crossing of cardiac progenitors), with highest phenotype severity with *sli*/*scb*, *robo2* mutations	*SLIT1*, *SLIT2*, *SLIT3*	15 (14.87, 14.87, 14.82)	*SLIT1* mutations associated with dysregulation in axonal guidance during development of the optic chiasm [460] (mouse)	Qian et al., 2005b [77], MacMullin and Jacobs, 2006 [449], Medioni et al., 2008 [246], Santiago-Martínez et al., 2008 [249], Zmojdzian et al., 2008 [57], Zmojdzian et al., 2018 [56], Raza and Jacobs, 2016 [370]
				*SLIT2* mutations/loss associated with thickened and immature semilunar valves [447] (mouse)	
				*SLIT3* mutations/loss associated with defects in the membranous ventricular septum, thickened and immature atrioventricular valves [447], severe reduction in sinus horn myocardium, hypoplastic caval veins, persistent left inferior caval vein [448], and enlarged right ventricle [461] (mouse)	
				*SLIT1*, *ROBO4* variants associated with a case presenting with BAV, ascending aorta aneurysm, and BAV [453] (human)	
				*SLIT2* variants associated with BAV [453] (human)	
				*SLIT3* variants associated with congenital heart defects in a case presenting with cardiac and renal malformation [462] and BAV with mitral regurgitation [453] (human)	
*Smox*	*Smox* mutations associated with adult lethality (adult)	*SMAD3*	14 (13.87)	*SMAD3* has not yet been specifically associated with cardiac development or congenital heart defects	Zhu et al., 2017 [35]
*Son*	*Son* mutations associated with disruption in actin filament and myofibril arrangement	*SON*	9 (8.77)	*SON* mutations associated with downregulation of genes related to cell cycle progression (particularly during the S, G2 phases) and cardiac progenitor, cardiomyocyte proliferation, disproportionate increase in fibroblasts compared to cardiomyocytes, and loss of embryonic stem cell pluripotency (in vitro models)	Schroeder et al., 2019 [233]
				*SON* variants associated with VSD and ASD, along with intellectual disability and developmental delay, 21q deletion syndrome (craniofacial dysmorphias, developmental delay, behavior abnormalities, and various systemic manifestations) [362] (Human)	
*Src42A*	*Src42A* mutations associated with “Open heart” phenotype with absence of cardioblast migration in the posterior dorsal vessel; absence of cardiac leading-edge activity; persistence of the Amnioserosa near the midline	*FRK*	13 (12.95)	*FRK* has not yet been specifically associated with cardiac development or congenital heart defects	Vanderploeg and Jacobs, 2017 [248]
*svp*	*svp* mutations/knockout associated with disruption in cardioblast phenotype and loss of svp+ cardioblast groups	*NR2F2*	13 (12.76)	*NR2F2* mutations associated with early embryonic lethality (homozygous loss) or lethality during puberty (heterozygous loss) [463] (mouse)	Lo and Frasch, 2001 [188], Hu et al., 2011 [113]
				*NR2F2* variants associated with DORV, VSD, ASD, TOF, PDA, BAV [217] (human)	
*ths*	*ths* mutations associated with defects in mesoderm migration, alongside ectoderm, mesoderm aberrant with multilayer formation, and subtle effects on *eve+* groups (embryo)	*FGF8*	1 (0.9)	*FGF8* mutations/knockout associated with absence of endoderm and embryonic mesoderm, embryonic lethality during gastrulation; defects involving cardiac looping, development of the outflow tract, anterior heart field, and survival of cardiac neural crest cells as they migrate toward the outflow tract, leading to outflow tract septation defects [443] (mouse)	Kadam et al., 2009 [261], Dorey and Amaya, 2010 [262]
				*FGF8* mutations contribute to 22q11.2DS deletion syndrome (craniofacial defects, immune dysfunction, and cardiac defects) [444] (human)	
*timp*	*timp* mutations associated with “Ectopic ECM” phenotype with longitudinal alary muscle arrangement along the dorsal vessel; disruption in pericardin arrangement with ectopic pericardin; and disruption in somatic muscle alignment (embryo)	*TIMP3*	15 (14.8)	*TIMP3*, *TIMP4* expression increased in embryonic cardiac tissues during episodes of maternal hypoxia, leading to inhibition of cardiomyocyte proliferation and maternal hypoxia associated with reduction in ventricular wall thickness [464] (rat)	Hughes et al., 2020 [254]
				*TIMP1* haploinsufficiency combined with *TIMP3* variants associated with BAV, aortopathy/aortic aneurysm in Turner syndrome [465,466] (human)	
*tin*	*tin* knockout associated with “No-heart” phenotype with absence of cardiac and dorsal somatic muscle	*NKX2*–*5*	5 (4.87)	*NKX2*–*5* mutations knockout associated with embryonic lethality, defects in cardiac morphology, and conduction with thin ventricular walls and septum defects (VSD), disruption in acetylcholine-based ventricular conduction, and cardiac arrhythmia (mouse) [185]	Bodmer et al., 1992 [181], Hu et al., 2011 [113], Yin and Frasch, 1998 [266]
				VSD, ASD, HLHS [10] (human)	
*Tkv*	*Tkv* overexpression associated with ectopic heart tissue formation in the ventral visceral mesoderm	*BMPR1B*	14 (13.74)	*BMP1RB has* not yet been associated with congenital heart defects in *H. sapiens* (human)	Yin and Frasch, 1998 [266]
*Trr*	*Trr* mutations associated with “Broken-hearted” phenotype (Embryo), disruption in physiologic heart function, with disruption in actin filament arrangement, reduction in cardiac myofibril density and cardioblast numbers, and increase in pericardin (cardiac fibrosis); reduction in diastolic/systolic diameter and heart rate (adult); late embryonic/early larval stage lethality; adult lethality; metabolic dysregulation (downregulation of muscle development genes; downregulation of ion transport genes) (Adult)	*KMT2C*	12 (11.73)	*KMT2C* mutations increase risk for the emergence of conotruncal defects in 22q11.2DS deletion syndrome (craniofacial defects, immune dysfunction, and cardiac defects) [467]; Kleefstra Syndrome (intellectual disability, autism spectrum disorder, and craniofacial defects) [416] (human)	J. Zhu et al., 2023 [327], Huang et al., 2022 [330]
*Trx*	*Trx* c in physiologic heart function, with disruption in actin filament arrangement, reduction in cardiac myofibril density and cardioblast numbers and increase in pericardin (cardiac fibrosis); increase in heart period; metabolic dysregulation (downregulation of muscle development genes; upregulation of ion transport genes) (adult)	*KMT2A*	13 (12.84)	*KMT2A* mutations associated with defects in the axial skeleton, hematopoiesis (Heterozygous loss), Embryonic lethality (Homozygous loss) [468] (mouse)	J. Zhu et al., 2023 [327]
				*KMT2A* variants associated with Wiedeman–Steiner Syndrome (excessive hair growth, short stature, distinct facial features, and heart defects) [469] (human)	
*tup*	*tup* mutations/loss associated with hypoplastic dorsal vessel with reduction in all cardioblast populations, disruption in pericardial cell alignment, and disruption in valve myofibril arrangement	*ISL1*, *ISL2*	16 (15.8, 15.75)	Deficiency of *ISL1* leads to complete absence of most of the atrial tissue, the right ventricle, and the outflow tract [193] (mouse)	Tao et al., 2007 [80]
				Deficiency of *ISL2a* leads to defects in cardiac looping, and deficiency of *ISL2b* is associated with defects in development of the arterial pole [196] (zebrafish)	
				*ISL1* variant associated with DORV in combination with VSD (heterozygous mutations) [197] (human)	
				*ISL2 has* not yet been associated with congenital heart defects in *H. sapiens* (human)	
*UbcD6*	*UbcD6* mutations associated with disruption in physiologic heart function, with reduction in cardiac myofibril density and increase in pericardin (cardiac fibrosis) (larva); reduction in cardiac myofibril density and cardioblast numbers and increase in Prc (cardiac fibrosis); adult lethality (adult)	*UBE2B*	15 (14.8)	Absence of monoubiquitylation at H2Bub1 (*RNF20* mutations), carried out by a complex involving RNF20, RNF40, UBE2B, associated with ventricular septum and ventricular compact myocardium thinning and abnormal sarcomere structure [350] (mouse)	Zhu et al., 2017 [35]
				*UBE2B* variants associated with TOF and right aortic arch [352] (human)	
*Ubx*	*Ubx* deficiency associated with disruption in anterior dorsal vessel structure; pericardial cell arrangement and cardioblast differentiation in segments T3-A1 and A2; absence of alary muscle formation in the anterior dorsal vessel with loss of the anterior 3 alary muscle pairs	*HOXB6*, *HOXC6*, *HOXC5*, *HOXA7*, *HOXB7*, *HOXB5*, *HOXA5*, *HOXD4*, *HOXA4*, *HOXB4*, *HOXC4*	4 (3.91, 3.91, 3.91, 3.91, 3.91, 3.91, 3.91, 3.81, 3.81, 3.81, 3.81)	Combined deletions in *HOXA*, *HOXB* clusters generally associated with defects in cardiac looping and appearance of primitive/atavistic heart morphologies [110] (mouse)	Lo et al., 2002 [45], Lovato et al., 2002 [47], Ponzielli et al., 2002 [309], Perrin et al., 2004 [308], Monier et al., 2005 [46], Ryan et al., 2005 [313], LaBeau et al., 2009 [311]
	*Ubx* overexpression/ectopic expression represses *Antp* expression and induces A5 segment identity in A1–A4 *tin+* cardioblasts			*HOXB7*, *HOXD8* cardiac expression altered in embryos after maternal alcohol consumption, via RNA-sequencing data [470]; *HOXB7* gain-of-function mutation associated with VSD, along with other congenital defects (cleft palate, renal anomalies, skeletal abnormalities [craniocervical, costosternal regions]) [471] (mouse)	
				*HOXB5* mutations associated with PDA [472] (animal models)	
				*HOXA1*, *HOXA2*, *HOXA3*, *HOXA4*, *HOXA13* mutations associated with 7p15 deletion syndrome (defects in facial, hand-foot morphology, supernumerary nipples, hypospadias, and hearing defects) [473] (human)	
				*HOXB6* variants associated with thoracic aortic dissection; *HOX* genes (*HOXA5*, *HOXB6*, *HOXC6*) may correlate with vascular smooth muscle cell de-differentiation in these cases [336] (human)	
				*HOXC4*, *HOXC5*, *HOXC6* variants associated with increased risk for simple congenital heart disease (human) [474]	
				*HOXA7*, *HOXB4*, *HOXD4* have not yet been specifically associated with cardiac development or congenital heart defects [337]	
*Vegf*	*Vegf* mutations associated with disruption in physiologic heart function with reduction in systolic motion (embryo) and cardiac output (larva), as well as disruption in ostial and aortic valve function	*PDGFA*	9 (8.83)	Loss of *PDGFA* leads to atrial and ventricular myocardial hypertrophy, defects in epicardial and endocardial cell groups, and aortic dilatation [475] (mouse)	Wu and Sato, 2008 [149]
				Increased maternal levels of PDGFAA associated with HLHS in the fetus [476] (human)	
*Wdr82*	*Wdr82* mutations associated with disruption in physiologic heart function with, disruption in actin filament arrangement; reduction in cardiac myofibril density and cardioblast numbers and increase in pericardin (cardiac fibrosis); increase in systolic diameter and reduction in diastolic diameter; adult lethality (adult)	*WDR82*	16 (15.8)	*WDR82* has not yet been directly associated with congenital heart defects in *H. sapiens* (human)	Zhu et al., 2024 [328]
*Wds*	*Wds* mutations associated with disruption in physiologic heart function, reduction in cardiac myofibril density and increase in pericardin (larva); reduction in cardiac myofibril density, pericardin, and cardioblast numbers; adult lethality (adult)	*WDR5*	16 (15.75)	*WDR5 mutations* associated with defects in cilia formation and left–right patterning [477] (frog)	Zhu et al., 2017 [35], Zhao et al., 2023 [478]
				*WDR5* variants associated with conotruncal defects with right aortic arch and mild heterotaxy phenotype [477] (human)	
*Wg*	*Wg* mutations associated with variable effects on cardiac development, ranging from no dorsal vessel formation, severe effects with reduction in cardioblast/pericardial cell numbers to no effects on dorsal vessel formation, depending on timing of gene mutation	*WNT1*, *WNT7A*, *WNT5A*	15 (14.7)	There are 19 Wnt proteins in mammalian vertebrates, many of which are implicated in cardiac development and associated with cardiac defects, including outflow tract defects and vascular smooth muscle defects [271]; *WNT1* implicated in neural crest development [479]	Wu et al., 1995 [265], Lockwood and Bodmer, 2002 [268]
				*WNT1* possibly implicated in HLHS (human) [479]	
				*WNT5A* mutations associated with disruption of second heart field (SHF) progenitor migration to the outflow tract, outflow tract defects, including PTA [279]	
				*WNT5A* variants associated with conotruncal defects [480], BAV [481] (human)	
				*WNT7A* cardiac expression altered in embryos after maternal alcohol consumption, based on RNA-sequencing data [470] (mouse)	
				Levels of DNA methylation in various genes, including *WNT7A*, may be associated with TOF [482] (human)	
				*WNT11* mutations associated with defects in ventricle and outflow tract formation [280] (mouse)	
				*WNT11* variants/mutations associated with VSD, TOF [281] (human)	
*DWnt4*, *Wnt4*	*DWnt4*, *Wnt4 mutations*, overall, not as severe as *Wg* mutations with disruption of normal gene marker expression in pericardial cell groups; disruption in the expression of pericardin and *Dmef2;* defects in cardioblast alignment with absence of unique morphology (constricted, elongated) of ostia progenitor cells in the posterior dorsal vessel; absence of ostia formation (embryo)	*WNT9B*	8 (7.76)	*WNT9B* mutations associated with enlargement of endocardial cushions, with septal cushion defects, valve defects and death in utero while endocardial-specific *WNT9B* deficiency does not affect valve development or survival [483] (zebrafish)	Tauc et al., 2012 [171], Graba et al., 1995 [170], Chen et al., 2016 [277]
				*WNT9B* variants associated with Alagille syndrome (multisystem disorder with heart defects) [484]; complex risk locus on chromosome 17 interacting with *WNT9B*, among others, associated with septal defects (VSD, ASD) and left-side congenital heart defects [485] (human)	
*wun2*, *wun*	Wun2/wun *mutations* associated with variable defects in dorsal closure and dorsal vessel structure ranging from delayed ectoderm leading-edge migration, gaps, multiple lumens, and loose cardioblast/pericardial cell attachment to luminal ectoderm/Amnioserosa remnants with disruption in midline cardioblast assembly (embryo)	*PLPP3*, *PLPP1*	15 (14.8)	*PLPP3* associated with extraembryonic vascular defects and early embryonic lethality [486] (mouse)	Haack et al., 2014 [84]
				*PLPP1 has* not yet been associated with cardiac development or congenital heart defects	
*αPS3* *(scb)*	*αPS3 mutations* associated with variable defects ranging from reduction/disruption of pericardial cell arrangement to complete cardioblast/pericardial cell dissociation with random migration patterns; absence of cardiac lumen formation	*ITGA4*, *ITGA5*	7 (6.76)	*ITGA4* mutations associated with defects in vascular development, absence of epicardium leading to embryonic lethality due to cardiac hemorrhage, defects in pericyte and presumptive vascular smooth muscle cell motility [487], and endocardial extrusions [488] (mouse)	Stark et al., 1997 [409], Moreira et al., 2013 [489], Vanderploeg et al., 2012 [83]
				*ITGA5* mutations associated with defects in endocardial morphology, endocardial differentiation with delayed formation of the endocardial sheet, pericardial edema, defects in cardiac looping, and defects in valve development; combined *ITGA4*, *ITGA5* mutations lead to severe defects in endocardial and myocardial migration, cardia–bifida possibly due to defects in anterior endodermal sheet formation; single *ITGA4* mutations show no cardiac defects in zebrafish [490] (zebrafish)	
				*ITGA5* mutations associated with defects in cardiac morphology, including defects in endocardial and myocardial migration, although less severe than fibronectin 1 mutations, resulting in cardia–bifida [490] (mouse)	
				*ITGA4* mutations possibly associated with a case presenting with DOLV, outlet VSD, large coronary arterio-ventricular fistula, hypertrabeculation, and poor compaction of the right ventricle [488]; *ITGA4* variants also associated with aortic stenosis [424] (human)	
*βPS (mys)*	*βPS mutations* associated with variable defects, including cardioblast displacement (most severe with *mys*); reduction in leading-edge activity	*ITGB1*	16 (15.82)	*ITGB1* mutation associated with expansion in endoderm formation in iPSC cultures [491] (in vitro models)	Stark et al., 1997 [409], Moreira et al., 2013 [489], Vanderploeg et al., 2012 [83]
				*FLNC* mutations/loss lead to disruption of the ITGB1-mediated interaction between FLNC and other factors, disrupting the interactions between actin filaments and extracellular matrix in cardiomyocytes during cardiac development; this leads to embryonic lethality and cardiac defects such as ventricular wall malformations and reduced cardiomyocyte proliferation [492] (mouse)	
				*ITGB1* has not yet been specifically associated with congenital heart defects in *H. sapiens* (human)	

## 5. Conclusions

The evolutionary timeline of species emergence is complex, and the timeline of new gene emergence is equally or even more so; as species diverge, and new phyla emerge, new complexities arise, driven by the complexities of the genetic networks orchestrating the development of their body plans. Not only do new genes arise via duplication, but these genes can then be inherited and, with the passage of time and across different species, they may lose some of the functions found in the ancestral gene and only carry out some of these original functions in the new organism, an event termed subfunctionalization. They may even acquire new functions altogether, a phenomenon known as neofunctionalization [493].

As these different sets of functions arise, all these homologous genes now participate in updated or completely new regulatory networks; furthermore, interactions between different genes can also be conserved and carried into the new species. An example of this is the conserved interactions between *Org-1* and its suppression of *tup;* in the fly, this interaction leads to the derivation of ventral longitudinal muscles, a skeletal muscle that interacts with and attaches to the dorsal vessel. In vertebrates, this interaction has been conserved between the *Org-1* ortholog *TBX1* and the *tup* ortholog *ISL1*; however, in this case, the resulting tissue that emerges comprises second heart field progenitors that contribute to elongation of the linear heart tube [63,90,238]. These conserved relationships become even more complex, as many of these orthologous relationships between genes connected by a common ancestor exhibit N:N relationship cardinality [112], which, in turn, makes genetic modeling in distantly related species complex as well [494].

Another caveat of modeling in distantly related species is the obvious divergence in animal form and function; though many animals will exhibit a phylotypic stage or “evolutionary hourglass” during stages of their embryogenesis, the animal body plan may differ greatly, depending on the species [495]. The fly and the human exhibit no anatomical homology and only some physiological homology in terms of heart structure and function; utilizing *D. melanogaster* and other relatively simple species, however, allows for the evaluation of genes whose deletion would be embryonically lethal to mammalian vertebrates and mammals. This is due to differences in cardiovascular and respiratory function that result in embryonic lethality and the non-viability of development past a certain stage in the latter [32,142]. The widespread effect of early cardiac transcription factors in the development of systems may also be the reason why it is more difficult to identify mutations of these factors in humans; some mutations are embryonically lethal and thus would not be identified in viable individuals [496].

In the end, the simplicity of the gene networks coupled with the simplicity in the anatomy/physiology allows for simple model organisms such as *D. melanogaster* to provide a useful first step in the identification and evaluation of candidate genes involved in cardiac development and, to an extent, cardiac defects when disrupted. These genes can then be further evaluated in other model organisms to fully ascertain their function in development and their contribution to congenital heart disease.

## Data Availability

Not applicable.

## Supplementary Materials

The following supporting information can be downloaded at https://www.mdpi.com/article/10.3390/biomedicines13102569/s1, Table S1: Cardioblast (CB) and pericardial cell (PC) populations identified in *D. melanogaster* during cardiac development, along with common gene markers for each cell type; Table S2: Genes involved in the cardiac gene regulatory networks: mutations and phenotypes; Table S3: Genes involved in cellular metabolism and protein synthesis/trafficking: mutations and phenotypes; Table S4: Genes involved in cardiac progenitor migration, alignment, and dorsal vessel assembly during *Drosophila melanogaster* embryonic development: mutations and phenotypes; Table S5: Genes involved in the establishment of segmentation and polarity during *Drosophila melanogaster* embryonic development: mutations and phenotypes; Table S6: Genes involved in the development of the animal body plan during *Drosophila melanogaster* embryonic development: mutations and phenotypes; Table S7: Genes involved in histone modification during *Drosophila melanogaster* embryonic development: mutations and phenotypes; Table S8: *D. melanogaster* and *H. sapiens* orthologs, summary of function for each gene in the context of cardiovascular development. In cases where a 1-to-many (1:N) or many-to-many (N:N) ortholog relationship is present, the ortholog with the highest DIOPT score, including weighted scores in parentheses, is noted in the corresponding column with additional orthologs, in descending DIOPT score order, included in a separate column. In cases where the highest predicted score is shared between more than one *H. sapiens* ortholog, these are noted in the first ortholog column; Table S9. Complete list of gene names used throughout the manuscript and supplemental sections. References for supplementary materials include citations in main text and references [497,498,499,500,501,502,503,504,505,506,507,508,509,510,511,512,513,514,515,516,517,518,519,520,521,522,523,524,525,526,527,528,529,530,531,532,533,534,535,536,537,538,539,540,541,542,543,544,545,546,547,548,549,550,551,552,553,554,555,556,557,558,559,560,561,562,563].

## Abbreviations

The following abbreviations are used in this manuscript:

A1	Abdominal segment A1
A2	Abdominal segment A2
A3	Abdominal segment A3
A4	Abdominal segment A4
A5	Abdominal segment A5
A6	Abdominal segment A6
A7	Abdominal segment A7
A8	Abdominal segment A8
Abd-A	Abdominal-A
Abd-B	Abdominal-B
ANT-C	Antennapedia (ANTP) complex
*Antp*	Antennapedia
ANTP	Named after Antennapedia (Antp) gene in *D. melanogaster*
Ash2	Absent, small, or homeotic disks 2
*Bab1/2*	Bric-à-brac
*Bag*	Bagpipe
bHLH	Basic helix–loop–helix transcription factor
BMP2/4	Bone morphogenetic protein 2/4
BX-C	Bithorax Complex
Cas9	Clustered regularly interspaced short palindromic repeats (CRISPR)-associated protein 9
Cas9n	Clustered regularly interspaced short palindromic repeats (CRISPR)-associated protein 9 nickase
*Cdc42*	Cell division control protein 42
CERS	Ceramide synthase
Ci	Cubitus interruptus
COMPASS	Complex of proteins associated with SET-containing domain 1 (Set1)
CRISPR	Clustered regularly interspaced short palindromic repeats
CUT	Named after the cut gene in *D. melanogaster*
*D-mef2*	Drosophila myocyte enhancer factor 2
*D. melanogaster*	*Drosophila melanogaster*
dDAAM	Dishevelled-associated activator of morphogenesis
*DE-Cadherin*	Drosophila epithelial cadherin
*Dg*	Dystroglycan
DIOPT	Drosophila RNAi Screening Center Integrative ortholog prediction tool
*Dlg*	Disks-large
DNA	Deoxyribonucleic acid
*DNMT1*	DNA methyltransferase 1
*DNMT3*	DNA methyltransferase 3
*Doc1/2/3*	Dorsocross 1/2/3
*Dpp*	Decapentaplegic
Dpy-30L1	Dpy-30-like 1
DRF	Diaphanous related formin
EGFR	Epidermal growth factor receptor
ELPC	End-of-the-line pericardial cells
*Ena*	Enabled
EPC	Even-skipped (Eve)+ tinman+ (tin)+ pericardial cell
ERBB	Erb-B2 Receptor Tyrosine Kinase 2
*Eve*	Even-skipped
*EVX1/2*	Even-Skipped Homeobox ½
FGF	Fibroblast growth factor
FGF8/10	Fibroblast growth factor 8/10
FGFR	Fibroblast growth factor receptor
*FGFR2B*	Fibroblast growth factor receptor 2
GAL4	Transcription factor GAL4
Gart	Phosphoribosylglycinamide formyltransferase, phosphoribosylglycinamide synthetase, phosphoribosylaminoimidazole synthetase
GGPPS/qm	Geranylgeranyl pyrophosphate synthase
*GTPase*	Guanosine triphosphatase
Gγ1	G protein gamma (γ) subunit 1
H3K27ac	Lysine 27 of Histone 3 acetylation
H3K36	Lysine 36 of Histone 3
H3K4	Lysine 4 of Histone 3
H3K4me	Lysine 4 of Histone 3 methylation
*Hand*	Heart and neural crest derivatives
*HAND1/2*	Heart and neural crest derivatives expressed 1/2
HMGCR	Hydroxymethyl-glutaryl (HMG) CoA reductase
HNF	Named after Hnf1 (mammalian)
HOM-C	Homeotic Complex
Hox	Homeobox gene
Hox1–13	Homeobox 1–13
HoxA/B/C/D	Homeobox A/B/C/D
*If*	Inflated
*ISL1**/2*	ISL LIM Homeobox ^1^/_2_
K+	Potassium
*KMT2C*	Lysine methyltransferase 2C
*KMT2D*	Lysine methyltransferase 2D
L1	Larval stage L1
L2	Larval stage L2
L3	Larval stage L3
*lb*	ladybird
LIM	Named after Lin-11 (nematodes) ISL1 (mammalian) Mec-3 (nematodes)
MAPK	Mitogen-associated protein kinase
*Mef-2*	Myocyte enhancer factor-2
*MEF2A-2D*	Myocyte enhancer factor 2A-2D
Mesp1	Mesoderm posterior basic helix–loop–helix transcription factor (BHLH) transcription factor 1
*Mew*	Multiple edematous wings
*Mid*	Midline
Mnn1	Menin 1
*Msh-2*	MutS Homolog 2
*MSX-2*	Msh Homeobox 2
*Mys*	Myospheroid
*NK*	NK Homeobox
*NK2*	NK2 Homeobox
*NKX2.1*–*2.6*	NK2 Homeobox 1–6
*Nmr1*	Neuromancer 1
*Nmr2*	Neuromancer 2
*Nos3*	Nitric oxide synthase 3
*NR2F2*	Even-skipped homeobox 1
*Odd*	Odd-skipped
*Omb*	Optomotor blind
OPC	Odd-skipped (Odd) pericardial cells
*Org-1*	Optomotor blind-related gene 1
PcG	Polycomb group
PCP	Planar cell polarity
*pERK1/2*	Protein kinase R-like endoplasmic reticulum kinase
POU	Named after POU1F1 (mammalian) OCT1, OCT2 (mammalian) Unc-86 (nematodes)
PRD	Named after Paired (prd) gene in *D. melanogaster*
PROS	Named after the pros gene in *D. melanogaster*
Ptip	PAX transcription activation domain-interacting protein
RAS	Rat sarcoma virus
Rbbp5	Retinoblastoma binding protein 5
RNA	Ribonucleic acid
RNAi	Ribonucleic acid interference
*Robo/2*	Roundabout
RTK	Receptor Tyrosine Kinase
*Scb*	Scab
*Scro*	Scarecrow
Set1	SET-containing domain 1
*Shg*	Shotgun
SINE	Named after co: sine oculis gene in *D. melanogaster*
*Svp*	Sevenup
T1	Thoracic segment T1
T2	Thoracic segment T2
T3	Thoracic segment T3
TALE	Three-amino acid loop extension
TALEN	Transcription activator-like effector nuclease
*TBX2*–*6*, *20*	T-Box Transcription Factor 2–6, 20
*tin*	tinman
Trr	Trithorax-related
Trx	Trithorax
TrxG	Trithorax group
*Tup*	Tailup
UAS_G_	Upstream activator sequence
Ubx	Ultrabithorax
*Vnd*	Ventral nervous system defective
Wdr82	WD repeat domain 82
Wds	Will die slowly
*Wg*	Wingless
WHPCs	Wing heart pericardial cells
Wnt2a/2b/5a/8a/11	Wingless (Wg)-related integration site 2a/2b/5a/8a/11
ZF	Zinc finger
ZFN	Zinc-finger nuclease
*αPS1*–*3*	alpha subunit integrin chain 1–3
*βPS*	Beta subunit integrin chain

## References

[1] Majumdar U., Yasuhara J., Garg V. (2019). In Vivo and In Vitro Genetic Models of Congenital Heart Disease. Cold Spring Harb. Perspect. Biol..

[2] Hasan A.A., Abu Lehyah N.A.A., Al Tarawneh M.K., Abbad M.Y., Fraijat A.G., Al-Jammal R.A., Moamar D.M., Shersheer Q.A., Guthrie S.O., Starnes J.R. (2023). Incidence and Types of Congenital Heart Disease at a Referral Hospital in Jordan: Retrospective Study from a Tertiary Center. Front. Pediatr..

[3] Walsh E.P., Gonzales C., Atallah J. (2013). Multicenter Case-Control Study of Ventricular Arrhythmia in Tetralogy of Fallot. Can. J. Cardiol..

[4] Blue G.M., Mekel M., Das D., Troup M., Rath E., Ip E., Gudkov M., Perumal G., Harvey R.P., Sholler G.F. (2022). Whole Genome Sequencing in Transposition of the Great Arteries and Associations with Clinically Relevant Heart, Brain and Laterality Genes. Am. Heart J..

[5] Shi G., Zhu F., Wen C., Yan Y., Zhang H., Zhu Z., Chen H. (2023). Cardiac-Type Total Anomalous Pulmonary Venous Return Is Not Benign. J. Thorac. Cardiovasc. Surg..

[6] Aldweib N., Broberg C. (2024). Failing with Cyanosis-Heart Failure in End-Stage Unrepaired or Partially Palliated Congenital Heart Disease. Heart Fail. Clin..

[7] Kido T., Guariento A., Doulamis I.P., Porras D., Baird C.W., del Nido P.J., Nathan M. (2021). Aortic Valve Surgery After Neonatal Balloon Aortic Valvuloplasty in Congenital Aortic Stenosis. Circ. Cardiovasc. Interv..

[8] Xiao S., Cao H., Liu J., Hong L., Ma J., Zhu Y., Xie Y., Zhang Z., Shi J., Cui L. (2025). A Novel Diagnostic Model for Fetal Coarctation of the Aorta with Ventricular Septal Defect. Int. J. Cardiol..

[9] Choi Y.Y., Woo M.H., Kim G.B., Song M.K., Lee S.Y., Bae E.J., Choi M., Kim Y.-S. (2018). A Family with *NKX2.5* Gene Mutations Presenting as Familial Atrial Septal Defect and Atrioventricular Block: A Case Report. J. Genet. Med..

[10] Perrot A., Rickert-Sperling S. (2024). Human Genetics of Ventricular Septal Defect. Adv. Exp. Med. Biol..

[11] Gupta S., Donn S.M. (2024). Management of Patent Ductus Arteriosus–Evidence to Practice. Semin. Fetal Neonatal Med..

[12] Belhadjer Z., Pontailler M., Hily M., Gaudin R., Raisky O., Bonnet D., Houyel L. (2025). The Particular Anatomy of Atrioventricular Septal Defect with a Common Valvar Orifice in Patients with Down Syndrome: An Echocardiographic Study. Int. J. Cardiol..

[13] Ye X.T., Henmi S., Buratto E., Haverty M.C., Yerebakan C., Fricke T., Brizard C.P., d’Udekem Y., Konstantinov I.E. (2024). Young Infants with Symptomatic Tetralogy of Fallot: Shunt or Primary Repair?. JTCVS Open.

[14] Ottaviani G., Buja L.M., Buja L.M., Butany J. (2022). Chapter 6-Congenital Heart Disease: Pathology, Natural History, and Interventions. Cardiovascular Pathology.

[15] Griffith E.G., Musaalo K., Jackson S.H., Ribeiro E.R. (2024). Cardiovascular Disease Associated with Genetic Defects. Prog. Pediatr. Cardiol..

[16] Spendlove S.J., Bondhus L., Lluri G., Sul J.H., Arboleda V.A. (2022). Polygenic Risk Scores of Endo-Phenotypes Identify the Effect of Genetic Background in Congenital Heart Disease. Hum. Genet. Genom. Adv..

[17] Ehrlich L., Prakash S.K. (2022). Copy-Number Variation in Congenital Heart Disease. Curr. Opin. Genet. Dev..

[18] Behiry E.G., Al-Azzouny M.A., Sabry D., Behairy O.G., Salem N.E. (2019). Association of NKX2-5, GATA4, and TBX5 Polymorphisms with Congenital Heart Disease in Egyptian Children. Mol. Genet. Genom. Med..

[19] Mustafa H.J., Jacobs K.M., Tessier K.M., Narasimhan S.L., Tofte A.N., McCarter A.R., Cross S.N. (2020). Chromosomal Microarray Analysis in the Investigation of Prenatally Diagnosed Congenital Heart Disease. Am. J. Obstet. Gynecol. MFM.

[20] El-Ella S.S.A., El Gendy F., Tawfik M.A.M., El Sobky E., Khattab A., El-mekkawy M. (2012). Chromosome 22 Microdeletion in Children with Syndromic Congenital Heart Disease by Fluorescent in Situ Hybridization (FISH). Egypt. J. Med. Hum. Genet..

[21] Han S., Zhang Y., Meng M., Hou Z., Meng P., Zhao Y., Gao H., Tang J., Liu Z., Yang L. (2020). Generation of Human iPSC Line from a Patient with Tetralogy of Fallot, YAHKMUi001-A, Carrying a Mutation in TBX1 Gene. Stem Cell Res..

[22] Lin A.E., Santoro S., High F.A., Goldenberg P., Gutmark-Little I. (2020). Congenital Heart Defects Associated with Aneuploidy Syndromes: New Insights into Familiar Associations. Am. J. Med. Genet. Part C Semin. Med. Genet..

[23] Choudhury T.Z., Garg V. (2022). Molecular Genetic Mechanisms of Congenital Heart Disease. Curr. Opin. Genet. Dev..

[24] Albar R.F., Alghamdi M.S., Almasrahi A.M., Aldawsari M.K., Aljahdali F.F., Alhwaity A.S. (2021). A Six-Year-Old Child With Mosaic Trisomy 13. Cureus.

[25] Trevisan V., Meroni A., Leoni C., Sirchia F., Politano D., Fiandrino G., Giorgio V., Rigante D., Limongelli D., Perri L. (2024). Trisomy 22 Mosaicism from Prenatal to Postnatal Findings: A Case Series and Systematic Review of the Literature. Genes.

[26] Milani D.A.Q., Chauhan P.R. (2023). Genetics, Mosaicism. StatPearls [Internet].

[27] Phung V., Singh K.E., Danon S., Tan C.A., Dabagh S. (2023). Non-Mosaic Trisomy 22 and Congenital Heart Surgery Using the Shared Decision Making Model: A Case Report. BMC Pediatr..

[28] Agarwal M., Kumar V., Dwivedi A. (2023). Diagnosis of 22q11.2 Deletion Syndrome in Children with Congenital Heart Diseases and Facial Dysmorphisms. Med. J. Armed Forces India.

[29] Simmons M.A., Brueckner M. (2017). The Genetics of Congenital Heart Disease…Understanding and Improving Long Term Outcomes in Congenital Heart Disease: A Review for the General Cardiologist and Primary Care Physician. Curr. Opin. Pediatr..

[30] Zhao Y., van de Leemput J., Han Z. (2023). The Opportunities and Challenges of Using Drosophila to Model Human Cardiac Diseases. Front. Physiol..

[31] Beller M., Oliver B. (2006). One Hundred Years of High-Throughput Drosophila Research. Chromosome Res..

[32] Hillyer J.F., Pass G. (2020). The Insect Circulatory System: Structure, Function, and Evolution. Annu. Rev. Entomol..

[33] Vivien C.J., Hudson J.E., Porrello E.R. (2016). Evolution, Comparative Biology and Ontogeny of Vertebrate Heart Regeneration. NPJ Regen. Med..

[34] Adams M.D., Celniker S.E., Holt R.A., Evans C.A., Gocayne J.D., Amanatides P.G., Scherer S.E., Li P.W., Hoskins R.A., Galle R.F. (2000). The Genome Sequence of *Drosophila melanogaster*. Science.

[35] Zhu J., Fu Y., Nettleton M., Richman A., Han Z. (2017). High Throughput in Vivo Functional Validation of Candidate Congenital Heart Disease Genes in Drosophila. eLife.

[36] Olson E.N. (2006). Gene Regulatory Networks in the Evolution and Development of the Heart. Science.

[37] Clark E., Peel A.D., Akam M. (2019). Arthropod Segmentation. Development.

[38] Klowden M.J., Palli S.R., Klowden M.J., Palli S.R. (2023). Chapter 7-Circulatory Systems. Physiological Systems in Insects.

[39] Hillyer J.F., Strand M.R. (2014). Mosquito Hemocyte-Mediated Immune Responses. Curr. Opin. Insect Sci..

[40] Wasserthal L.T. (2007). Drosophila Flies Combine Periodic Heartbeat Reversal with a Circulation in the Anterior Body Mediated by a Newly Discovered Anterior Pair of Ostial Valves and `venous’ Channels. J. Exp. Biol..

[41] Rotstein B., Paululat A. (2016). On the Morphology of the Drosophila Heart. J. Cardiovasc. Dev. Dis..

[42] Monahan-Earley R., Dvorak A.M., Aird W.C. (2013). Evolutionary Origins of the Blood Vascular System and Endothelium. J. Thromb. Haemost..

[43] Farmer C.G. (1999). Evolution of the vertebrate cardio-pulmonary system. Annu. Rev. Physiol..

[44] Stephenson A., Adams J.W., Vaccarezza M. (2017). The Vertebrate Heart: An Evolutionary Perspective. J. Anat..

[45] Lo P.C.H., Skeath J.B., Gajewski K., Schulz R.A., Frasch M. (2002). Homeotic Genes Autonomously Specify the Anteroposterior Subdivision of the *Drosophila* Dorsal Vessel into Aorta and Heart. Dev. Biol..

[46] Monier B., Astier M., Sémériva M., Perrin L. (2005). Steroid-Dependent Modification of Hox Function Drives Myocyte Reprogramming in the Drosophila Heart. Development.

[47] Lovato T.L., Nguyen T.P., Molina M.R., Cripps R.M. (2002). The Hox Gene Abdominal-A Specifies Heart Cell Fate in the Drosophila Dorsal Vessel. Development.

[48] Lehmacher C., Abeln B., Paululat A. (2012). The Ultrastructure of *Drosophila* Heart Cells. Arthropod Struct. Dev..

[49] Ward E.J., Coulter D.E. (2000). *Odd-Skipped* Is Expressed in Multiple Tissues during *Drosophila* Embryogenesis. Mech. Dev..

[50] Ward E.J., Skeath J.B. (2000). Characterization of a Novel Subset of Cardiac Cells and Their Progenitors in the Drosophila Embryo. Development.

[51] Huang X., Fu Y., Lee H., Zhao Y., Yang W., van de Leemput J., Han Z. (2023). Single-Cell Profiling of the Developing Embryonic Heart in Drosophila. Development.

[52] Pass G., Tögel M., Krenn H., Paululat A. (2015). The Circulatory Organs of Insect Wings: Prime Examples for the Origin of Evolutionary Novelties. Zool. Anz. J. Comp. Zool..

[53] Jürgens K.J., Drechsler M., Paululat A. (2024). An Anatomical Atlas of *Drosophila melanogaster*—The Wild-Type. Genetics.

[54] Lammers K., Abeln B., Hüsken M., Lehmacher C., Psathaki O.E., Alcorta E., Meyer H., Paululat A. (2017). Formation and Function of Intracardiac Valve Cells in the Drosophila Heart. J. Exp. Biol..

[55] Tang M., Yuan W., Bodmer R., Wu X., Ocorr K. (2014). The Role of Pygopus in the Differentiation of Intra-Cardiac Valves in Drosophila. Genesis.

[56] Zmojdzian M., de Joussineau S., Da Ponte J.P., Jagla K. (2018). Distinct Subsets of Eve-Positive Pericardial Cells Stabilise Cardiac Outflow and Contribute to Hox Gene-Triggered Heart Morphogenesis in Drosophila. Development.

[57] Zmojdzian M., Da Ponte J.P., Jagla K. (2008). Cellular Components and Signals Required for the Cardiac Outflow Tract Assembly in Drosophila. Proc. Natl. Acad. Sci. USA.

[58] Reim I., Frasch M. (2010). Genetic and Genomic Dissection of Cardiogenesis in the Drosophila Model. Pediatr. Cardiol..

[59] Kawasaki Y., Matsumoto A., Miyaki T., Kinoshita M., Kakuta S., Sakai T., Ichimura K. (2019). Three-Dimensional Architecture of Pericardial Nephrocytes in *Drosophila melanogaster* Revealed by FIB/SEM Tomography. Cell Tissue Res..

[60] Lim H.-Y., Wang W., Chen J., Ocorr K., Bodmer R. (2014). ROS Regulate Cardiac Function via a Distinct Paracrine Mechanism. Cell Rep..

[61] Sláma K. (2013). Physiology of Heartbeat Reversal in Adult *Drosophila melanogaster* (Diptera: Drosophilidae). Eur. J. Entomol..

[62] Meyer C., Drechsler M., Meyer H., Paululat A. (2023). Differentiation and Function of Cardiac Valves in the Adult Drosophila Heart. J. Exp. Biol..

[63] Schaub C., März J., Reim I., Frasch M. (2015). Org-1-Dependent Lineage Reprogramming Generates the Ventral Longitudinal Musculature of the Drosophila Heart. Curr. Biol..

[64] Drechsler M., Schmidt A.C., Meyer H., Paululat A. (2013). The Conserved ADAMTS-like Protein Lonely Heart Mediates Matrix Formation and Cardiac Tissue Integrity. PLoS Genet..

[65] Blice-Baum A.C., Guida M.C., Hartley P.S., Adams P.D., Bodmer R., Cammarato A. (2019). As Time Flies by: Investigating Cardiac Aging in the Short-Lived *Drosophila* Model. Biochim. Biophys. Acta (BBA) Mol. Basis Dis..

[66] Bataillé L., Lebreton G., Boukhatmi H., Vincent A. (2024). Insights and Perspectives on the Enigmatic Alary Muscles of Arthropods. Front. Cell Dev. Biol..

[67] Tögel M., Pass G., Paululat A. (2008). The *Drosophila* Wing Hearts Originate from Pericardial Cells and Are Essential for Wing Maturation. Dev. Biol..

[68] Farmer A.J., Katariya R., Islam S., Rayhan M.d.S.A., Inlow M.H., Ahmad S.M., Schwab K.R. (2025). Trithorax Is an Essential Regulator of Cardiac Hox Gene Expression and Anterior-Posterior Patterning of the Drosophila Embryonic Heart Tube. Biol. Open.

[69] Koehler S., Huber T.B. (2023). Insights into Human Kidney Function from the Study of Drosophila. Pediatr. Nephrol..

[70] Molina M.R., Cripps R.M. (2001). Ostia, the Inflow Tracts of the *Drosophila* Heart, Develop from a Genetically Distinct Subset of Cardial Cells. Mech. Dev..

[71] Gilbert S.F. (2000). An Introduction to Early Developmental Processes. Developmental Biology.

[72] Jaeger J., Manu, Reinitz J. (2012). *Drosophila* Blastoderm Patterning. Curr. Opin. Genet. Dev..

[73] Hickman C.P., Keen S., Eisenhour D., Larson A., l’Anson H. (2023). Integrated Principles of Zoology.

[74] Gomez J.M., Bevilacqua C., Thayambath A., Heriche J.-K., Leptin M., Belmonte J.M., Prevedel R. (2025). Highly Dynamic Mechanical Transitions in Embryonic Cell Populations during Drosophila Gastrulation. Nat. Commun..

[75] Dondi C., Bertin B., Da Ponte J.-P., Wojtowicz I., Jagla K., Junion G. (2021). A Polarized Nucleus-Cytoskeleton-ECM Connection in Migrating Cardioblasts Controls Heart Tube Formation in Drosophila. Development.

[76] Han Z., Bodmer R. (2003). Myogenic Cells Fates Are Antagonized by Notch Only in Asymmetric Lineages of the Drosophila Heart, with or without Cell Division. Development.

[77] Qian L., Liu J., Bodmer R. (2005). Slit and Robo Control Cardiac Cell Polarity and Morphogenesis. Curr. Biol..

[78] Han Z., Olson E.N. (2005). Hand Is a Direct Target of Tinman and GATA Factors during Drosophila Cardiogenesis and Hematopoiesis. Development.

[79] Zaffran S., Reim I., Qian L., Lo P.C., Bodmer R., Frasch M. (2006). Cardioblast-Intrinsic Tinman Activity Controls Proper Diversification and Differentiation of Myocardial Cells in Drosophila. Development.

[80] Tao Y., Wang J., Tokusumi T., Gajewski K., Schulz R.A. (2007). Requirement of the LIM Homeodomain Transcription Factor Tailup for Normal Heart and Hematopoietic Organ Formation in *Drosophila melanogaster*. Mol. Cell Biol..

[81] Mann T., Bodmer R., Pandur P. (2009). The Drosophila Homolog of Vertebrate Islet1 Is a Key Component in Early Cardiogenesis. Development.

[82] Bodmer R., Frasch M., Rosenthal N., Harvey R.P. (2010). Development and Aging of the *Drosophila* Heart. Heart Development and Regeneration.

[83] Vanderploeg J., Vazquez Paz L.L., MacMullin A., Jacobs J.R. (2012). Integrins Are Required for Cardioblast Polarisation in Drosophila. BMC Dev. Biol..

[84] Haack T., Schneider M., Schwendele B., Renault A.D. (2014). *Drosophila* Heart Cell Movement to the Midline Occurs through Both Cell Autonomous Migration and Dorsal Closure. Dev. Biol..

[85] Ahmad S.M. (2017). Conserved Signaling Mechanisms in Drosophila Heart Development. Dev. Dyn..

[86] Schwarz B., Hollfelder D., Scharf K., Hartmann L., Reim I. (2018). Diversification of Heart Progenitor Cells by EGF Signaling and Differential Modulation of ETS Protein Activity. eLife.

[87] Panta M., Kump A.J., Dalloul J.M., Schwab K.R., Ahmad S.M. (2020). Three Distinct Mechanisms, Notch Instructive, Permissive, and Independent, Regulate the Expression of Two Different Pericardial Genes to Specify Cardiac Cell Subtypes. PLoS ONE.

[88] Wang H., Kim J., Wang Z., Yan X.-X., Dean A., Xu W. (2020). Crystal Structure of Human LDB1 in Complex with SSBP2. Proc. Natl. Acad. Sci. USA.

[89] Babaoglan A.B., O’Connor-Giles K.M., Mistry H., Schickedanz A., Wilson B.A., Skeath J.B. (2009). Sanpodo: A Context-Dependent Activator and Inhibitor of Notch Signaling during Asymmetric Divisions. Development.

[90] Bileckyj C., Blotz B., Cripps R.M. (2023). Drosophila as a Model to Understand Second Heart Field Development. J. Cardiovasc. Dev. Dis..

[91] Favarolo M.B., López S.L. (2018). Notch Signaling in the Division of Germ Layers in Bilaterian Embryos. Mech. Dev..

[92] Hariri F., Nemer M., Nemer G. (2012). T-Box Factors: Insights into the Evolutionary Emergence of the Complex Heart. Ann. Med..

[93] Spring J., Yanze N., Jösch C., Middel A.M., Winninger B., Schmid V. (2002). Conservation of *Brachyury*, *Mef2*, and *Snail* in the Myogenic Lineage of Jellyfish: A Connection to the Mesoderm of Bilateria. Dev. Biol..

[94] Cridge A.G., Dearden P.K., Brownfield L.R. (2016). Convergent Occurrence of the Developmental Hourglass in Plant and Animal Embryogenesis?. Ann. Bot..

[95] Liu L., Yu L., Kubatko L., Pearl D.K., Edwards S.V. (2009). Coalescent Methods for Estimating Phylogenetic Trees. Mol. Phylogenetics Evol..

[96] Nixon K.C., Levin S.A. (2001). Phylogeny. Encyclopedia of Biodiversity.

[97] Pamilo P., Nei M. (1988). Relationships between Gene Trees and Species Trees. Mol. Biol. Evol..

[98] Blais C., Archibald J.M. (2021). The Past, Present and Future of the Tree of Life. Curr. Biol..

[99] Soucy S.M., Huang J., Gogarten J.P. (2015). Horizontal Gene Transfer: Building the Web of Life. Nat. Rev. Genet..

[100] Mindell D.P., Meyer A. (2001). Homology Evolving. Trends Ecol. Evol..

[101] Mabee P.M., Balhoff J.P., Dahdul W.M., Lapp H., Mungall C.J., Vision T.J. (2020). A Logical Model of Homology for Comparative Biology. Syst. Biol..

[102] Ochoterena H., Vrijdaghs A., Smets E., Claßen-Bockhoff R. (2019). The Search for Common Origin: Homology Revisited. Syst. Biol..

[103] Cerca J. (2023). Understanding Natural Selection and Similarity: Convergent, Parallel and Repeated Evolution. Mol. Ecol..

[104] Gabora L., Maloy S., Hughes K. (2013). Convergent Evolution. Brenner’s Encyclopedia of Genetics.

[105] Webber C., Ponting C.P. (2004). Genes and Homology. Curr. Biol..

[106] Fitch W.M. (2000). Homology: A Personal View on Some of the Problems. Trends Genet..

[107] Mallo D., de Oliveira Martins L., Posada D. (2014). Unsorted Homology within Locus and Species Trees. Syst. Biol..

[108] Gabaldón T., Koonin E.V. (2013). Functional and Evolutionary Implications of Gene Orthology. Nat. Rev. Genet..

[109] Hardison R.C. (2012). Evolution of Hemoglobin and Its Genes. Cold Spring Harb. Perspect. Med..

[110] Soshnikova N., Dewaele R., Janvier P., Krumlauf R., Duboule D. (2013). Duplications of Hox Gene Clusters and the Emergence of Vertebrates. Dev. Biol..

[111] Zou Y., Yang J., Zhou J., Liu G., Shen L., Zhou Z., Su Z., Gu X. (2024). Anciently Duplicated Genes Continuously Recruited to Heart Expression in Vertebrate Evolution Are Associated with Heart Chamber Increase. J. Exp. Zool. Part B: Mol. Dev. Evol..

[112] Zahn-Zabal M., Dessimoz C., Glover N.M. (2020). Identifying Orthologs with OMA: A Primer. F1000Research.

[113] Hu Y., Flockhart I., Vinayagam A., Bergwitz C., Berger B., Perrimon N., Mohr S.E. (2011). An Integrative Approach to Ortholog Prediction for Disease-Focused and Other Functional Studies. BMC Bioinform..

[114] Cao C., Li L., Zhang Q., Li H., Wang Z., Wang A., Liu J. (2023). Nkx2.5: A Crucial Regulator of Cardiac Development, Regeneration and Diseases. Front. Cardiovasc. Med..

[115] Mio C., Baldan F., Damante G. (2023). NK2 Homeobox Gene Cluster: Functions and Roles in Human Diseases. Genes Dis..

[116] Yoo S., Nair S., Kim H.-J., Kim Y., Lee C., Lee G., Park J.H. (2020). Knock-in Mutations of Scarecrow, a Drosophila Homolog of Mammalian Nkx2.1, Reveal a Novel Function Required for Development of the Optic Lobe in *Drosophila melanogaster*. Dev. Biol..

[117] Jiménez F., Martin-Morris L.E., Velasco L., Chu H., Sierra J., Rosen D.R., White K. (1995). Vnd, a Gene Required for Early Neurogenesis of Drosophila, Encodes a Homeodomain Protein. EMBO J..

[118] Chmykhalo V.K., Amendola D., Shidlovskii Y.V., Lebedeva L.A., Schedl P., Giordano E. (2025). Functional Role of Bap170 Domains in Enhancer-Dependent Gene Activity in *Drosophila melanogaster*. Dokl. Biochem. Biophys..

[119] Scott I.C., Bruneau B.G. (2012). Life Before *Nkx2.5*. Current Topics in Developmental Biology.

[120] Lien C.-L., Wu C., Mercer B., Webb R., Richardson J.A., Olson E.N. (1999). Control of Early Cardiac-Specific Transcription of Nkx2-5 by a GATA-Dependent Enhancer. Development.

[121] Fu Y., Yan W., Mohun T.J., Evans S.M. (1998). Vertebrate Tinman Homologues XNkx2-3 and XNkx2-5 Are Required for Heart Formation in a Functionally Redundant Manner. Development.

[122] Park M., Lewis C., Turbay D., Chung A., Chen J.-N., Evans S., Breitbart R.E., Fishman M.C., Izumo S., Bodmer R. (1998). Differential Rescue of Visceral and Cardiac Defects in Drosophila by Vertebrate Tinman-Related Genes. Proc. Natl. Acad. Sci. USA.

[123] Reim I., Frasch M., Schaub C., Frasch M. (2017). T-Box Genes in *Drosophila* Mesoderm Development. Current Topics in Developmental Biology.

[124] Sadahiro T., Isomi M., Muraoka N., Kojima H., Haginiwa S., Kurotsu S., Tamura F., Tani H., Tohyama S., Fujita J. (2018). Tbx6 Induces Nascent Mesoderm from Pluripotent Stem Cells and Temporally Controls Cardiac versus Somite Lineage Diversification. Cell Stem Cell.

[125] Pflugfelder G.O., Roth H., Poeck B., Kerscher S., Schwarz H., Jonschker B., Heisenberg M. (1992). The Lethal(1)Optomotor-Blind Gene of *Drosophila melanogaster* Is a Major Organizer of Optic Lobe Development: Isolation and Characterization of the Gene. Proc. Natl. Acad. Sci. USA.

[126] Grimm S., Pflugfelder G.O. (1996). Control of the Gene Optomotor-Blind in Drosophila Wing Development by Decapentaplegic and Wingless. Science.

[127] Liu N., Schoch K., Luo X., Pena L.D.M., Bhavana V.H., Kukolich M.K., Stringer S., Powis Z., Radtke K., Mroske C. (2018). Functional Variants in TBX2 Are Associated with a Syndromic Cardiovascular and Skeletal Developmental Disorder. Hum. Mol. Genet..

[128] Couderc J.-L., Godt D., Zollman S., Chen J., Li M., Tiong S., Cramton S.E., Sahut-Barnola I., Laski F.A. (2002). The Bric à Brac Locus Consists of Two Paralogous Genes Encoding BTB/POZ Domain Proteins and Acts as a Homeotic and Morphogenetic Regulator of Imaginal Development in Drosophila. Development.

[129] Bourbon H.-M.G., Benetah M.H., Guillou E., Mojica-Vazquez L.H., Baanannou A., Bernat-Fabre S., Loubiere V., Bantignies F., Cavalli G., Boube M. (2022). A Shared Ancient Enhancer Element Differentially Regulates the Bric-a-Brac Tandem Gene Duplicates in the Developing Drosophila Leg. PLoS Genet..

[130] Junion G., Bataillé L., Jagla T., Ponte J.P.D., Tapin R., Jagla K. (2007). Genome-Wide View of Cell Fate Specification: Ladybird Acts at Multiple Levels during Diversification of Muscle and Heart Precursors. Genes Dev..

[131] Rauzi M., Hočevar Brezavšček A., Ziherl P., Leptin M. (2013). Physical Models of Mesoderm Invagination in *Drosophila* Embryo. Biophys. J..

[132] Salinas-Saavedra M., Rock A.Q., Martindale M.Q. (2018). Germ Layer-Specific Regulation of Cell Polarity and Adhesion Gives Insight into the Evolution of Mesoderm. eLife.

[133] Nájera G.S., Weijer C.J. (2023). The Evolution of Gastrulation Morphologies. Development.

[134] Tada M., Heisenberg C.-P. (2012). Convergent Extension: Using Collective Cell Migration and Cell Intercalation to Shape Embryos. Development.

[135] Holland L.Z. (2015). Evolution of Basal Deuterostome Nervous Systems. J. Exp. Biol..

[136] Martindale M.Q. (2013). Evolution of Development: The Details Are in the Entrails. Curr. Biol..

[137] Ruggiero M.A., Gordon D.P., Orrell T.M., Bailly N., Bourgoin T., Brusca R.C., Cavalier-Smith T., Guiry M.D., Kirk P.M. (2015). A Higher Level Classification of All Living Organisms. PLoS ONE.

[138] Court R., Namiki S., Armstrong J.D., Börner J., Card G., Costa M., Dickinson M., Duch C., Korff W., Mann R. (2020). A Systematic Nomenclature for the Drosophila Ventral Nerve Cord. Neuron.

[139] Arendt D. (2018). Animal Evolution: Convergent Nerve Cords?. Curr. Biol..

[140] Gerhart J. (2000). Inversion of the Chordate Body Axis: Are There Alternatives?. Proc. Natl. Acad. Sci. USA.

[141] Arendt D., Nübler-Jung K. (1994). Inversion of Dorsoventral Axis?. Nature.

[142] Bier E., Bodmer R. (2004). *Drosophila*, an Emerging Model for Cardiac Disease. Gene.

[143] Saijoh Y., Hamada H. (2020). Making the Right Loop for the Heart. Dev. Cell.

[144] Dor Y., Camenisch T.D., Itin A., Fishman G.I., McDonald J.A., Carmeliet P., Keshet E. (2001). A Novel Role for VEGF in Endocardial Cushion Formation and Its Potential Contribution to Congenital Heart Defects. Development.

[145] Jiao K., Langworthy M., Batts L., Brown C.B., Moses H.L., Baldwin H.S. (2006). Tgfβ Signaling Is Required for Atrioventricular Cushion Mesenchyme Remodeling during in Vivo Cardiac Development. Development.

[146] Jensen B., Wang T., Moorman A.F.M. (2019). Evolution and Development of the Atrial Septum. Anat. Rec..

[147] Poelmann R.E., Groot A.C.G., Vicente-Steijn R., Wisse L.J., Bartelings M.M., Everts S., Hoppenbrouwers T., Kruithof B.P.T., Jensen B., de Bruin P.W. (2014). Evolution and Development of Ventricular Septation in the Amniote Heart. PLoS ONE.

[148] Choma M.A., Suter M.J., Vakoc B.J., Bouma B.E., Tearney G.J. (2011). Physiological Homology between *Drosophila melanogaster* and Vertebrate Cardiovascular Systems. Dis. Models Mech..

[149] Wu M., Sato T.N. (2008). On the Mechanics of Cardiac Function of Drosophila Embryo. PLoS ONE.

[150] Venken K.J.T., Sarrion-Perdigones A., Vandeventer P.J., Abel N.S., Christiansen A.E., Hoffman K.L. (2016). Genome Engineering: *Drosophila melanogaster* and Beyond. Wiley Interdiscip. Rev. Dev. Biol..

[151] Nim H.T., Dang L., Thiyagarajah H., Bakopoulos D., See M., Charitakis N., Sibbritt T., Eichenlaub M.P., Archer S.K., Fossat N. (2021). A Cis-Regulatory-Directed Pipeline for the Identification of Genes Involved in Cardiac Development and Disease. Genome Biol..

[152] Auxerre-Plantié E., Nielsen T., Grunert M., Olejniczak O., Perrot A., Özcelik C., Harries D., Matinmehr F., Dos Remedios C., Mühlfeld C. (2020). Identification of MYOM2 as a Candidate Gene in Hypertrophic Cardiomyopathy and Tetralogy of Fallot, and Its Functional Evaluation in the Drosophila Heart. Dis. Models Mech..

[153] Wittkopp P.J., Kalay G. (2012). Cis-Regulatory Elements: Molecular Mechanisms and Evolutionary Processes Underlying Divergence. Nat. Rev. Genet..

[154] Szallasi Z. (2001). To Kill Two Birds with One Stone: A General Concept in Gene Regulation?. Trends Pharmacol. Sci..

[155] Zimmer A.M., Pan Y.K., Chandrapalan T., Kwong R.W.M., Perry S.F. (2019). Loss-of-Function Approaches in Comparative Physiology: Is There a Future for Knockdown Experiments in the Era of Genome Editing?. J. Exp. Biol..

[156] Haiyong H. (2018). RNA Interference to Knock Down Gene Expression. Methods Mol. Biol..

[157] Ghosh A., Banerjee A., Gupta S., Sinha S. (2024). A Unified Phosphoramidite Platform for the Synthesis of Morpholino Oligonucleotides and Diverse Chimeric Backbones. J. Am. Chem. Soc..

[158] Kok F.O., Shin M., Ni C.-W., Gupta A., Grosse A.S., van Impel A., Kirchmaier B.C., Peterson-Maduro J., Kourkoulis G., Male I. (2015). Reverse Genetic Screening Reveals Poor Correlation between Morpholino-Induced and Mutant Phenotypes in Zebrafish. Dev. Cell.

[159] Paschon D.E., Lussier S., Wangzor T., Xia D.F., Li P.W., Hinkley S.J., Scarlott N.A., Lam S.C., Waite A.J., Truong L.N. (2019). Diversifying the Structure of Zinc Finger Nucleases for High-Precision Genome Editing. Nat. Commun..

[160] Becker S., Boch J. (2021). TALE and TALEN Genome Editing Technologies. Gene Genome Ed..

[161] Sloutskin A., Itzhak D., Vogler G., Pozeilov H., Ideses D., Alter H., Adato O., Shachar H., Doniger T., Shohat-Ophir G. (2024). From Promoter Motif to Cardiac Function: A Single DPE Motif Affects Transcription Regulation and Organ Function in Vivo. Development.

[162] Tsai S.Q., Joung J.K. (2016). Defining and Improving the Genome-Wide Specificities of CRISPR–Cas9 Nucleases. Nat. Rev. Genet..

[163] Fu Y., Sander J.D., Reyon D., Cascio V.M., Joung J.K. (2014). Improving CRISPR-Cas Nuclease Specificity Using Truncated Guide RNAs. Nat. Biotechnol..

[164] Arkin M., Maloy S., Hughes K. (2001). In Vitro Mutagenesis. Brenner’s Encyclopedia of Genetics.

[165] Franke J.D., Montague R.A., Rickoll W.L., Kiehart D.P. (2007). An MYH9 Human Disease Model in Flies: Site-Directed Mutagenesis of the Drosophila Non-Muscle Myosin II Results in Hypomorphic Alleles with Dominant Character. Hum. Mol. Genet..

[166] Lin S.-C., Chang Y.-Y., Chan C.-C. (2014). Strategies for Gene Disruption in Drosophila. Cell Biosci..

[167] Roote J., Russell S. (2012). Toward a Complete Drosophiladeficiency Kit. Genome Biol..

[168] Prelich G. (2012). Gene Overexpression: Uses, Mechanisms, and Interpretation. Genetics.

[169] Jagla K., Frasch M., Jagla T., Dretzen G., Bellard F., Bellard M. (1997). Ladybird, a New Component of the Cardiogenic Pathway in Drosophila Required for Diversification of Heart Precursors. Development.

[170] Graba Y., Gieseler K., Aragnol D., Laurenti P., Mariol M.-C., Berenger H., Sagnier T., Pradel J. (1995). DWnt-4, a Novel Drosophila Wnt Gene Acts Downstream of Homeotic Complex Genes in the Visceral Mesoderm. Development.

[171] Tauc H.M., Mann T., Werner K., Pandur P. (2012). A Role for Drosophila Wnt-4 in Heart Development. Genesis.

[172] Elliott D.A., Brand A.H., Dahmann C. (2008). The GAL4 System. Drosophila: Methods and Protocols.

[173] Xu Y., Gan E.-S., Ito T. (2023). Misexpression Approaches for the Manipulation of Flower Development. Methods Mol. Biol..

[174] Veitia R.A. (2023). Dominant Negative Variants and Cotranslational Assembly of Macromolecular Complexes. BioEssays.

[175] Harrington S.A., Backhaus A.E., Fox S., Rogers C., Borrill P., Uauy C., Richardson A. (2020). A Heat-Shock Inducible System for Flexible Gene Expression in Cereals. Plant Methods.

[176] Park M., Wu X., Golden K., Axelrod J.D., Bodmer R. (1996). The Wingless Signaling Pathway Is Directly Involved in *Drosophila* Heart Development. Dev. Biol..

[177] Schramm T., Lubrano P., Pahl V., Stadelmann A., Verhülsdonk A., Link H. (2023). Mapping Temperature-sensitive Mutations at a Genome Scale to Engineer Growth Switches in *Escherichia coli*. Mol. Syst. Biol..

[178] Susman M., Brenner S., Miller J.H. (2001). Conditional Lethality. Encyclopedia of Genetics.

[179] Bodmer R., Jan L.Y., Jan Y.N. (1990). A New Homeobox-Containing Gene, Msh-2, Is Transiently Expressed Early during Mesoderm Formation of Drosophila. Development.

[180] Chen Y.-H., Ishii M., Sun J., Sucov H.M., Maxson R.E. (2007). *Msx1* and *Msx2* Regulate Survival of Secondary Heart Field Precursors and Post-Migratory Proliferation of Cardiac Neural Crest in the Outflow Tract. Dev. Biol..

[181] Bodmer R. (1993). The Gene Tinman Is Required for Specification of the Heart and Visceral Muscles in Drosophila. Development.

[182] Reim I., Frasch M. (2005). The Dorsocross T-Box Genes Are Key Components of the Regulatory Network Controlling Early Cardiogenesis in Drosophila. Development.

[183] Lovato T.L., Blotz B., Bileckyj C., Johnston C.A., Cripps R.M. (2023). Modeling a Variant of Unknown Significance in the Drosophila Ortholog of the Human Cardiogenic Gene *NKX2.5*. DMM Dis. Models Mech..

[184] Targoff K.L., Schell T., Yelon D. (2008). *Nkx* Genes Regulate Heart Tube Extension and Exert Differential Effects on Ventricular and Atrial Cell Number. Dev. Biol..

[185] Terada R., Warren S., Lu J.T., Chien K.R., Wessels A., Kasahara H. (2011). Ablation of Nkx2-5 at Mid-Embryonic Stage Results in Premature Lethality and Cardiac Malformation. Cardiovasc. Res..

[186] Targoff K.L., Colombo S., George V., Schell T., Kim S.-H., Solnica-Krezel L., Yelon D. (2013). Nkx Genes Are Essential for Maintenance of Ventricular Identity. Development.

[187] Elliott D.A., Kirk E.P., Yeoh T., Chandar S., McKenzie F., Taylor P., Grossfeld P., Fatkin D., Jones O., Hayes P. (2003). Cardiac Homeobox Gene NKX2-5 Mutations and Congenital Heart Disease: Associations with Atrial Septal Defect and Hypoplastic Left Heart Syndrome. J. Am. Coll. Cardiol..

[188] Lo P.C.H., Frasch M. (2001). A Role for the COUP-TF-Related Gene Seven-up in the Diversification of Cardioblast Identities in the Dorsal Vessel of *Drosophila*. Mech. Dev..

[189] Fujioka M., Wessells R.J., Han Z., Liu J., Fitzgerald K., Yusibova G.L., Zamora M., Ruiz-Lozano P., Bodmer R., Jaynes J.B. (2005). Embryonic Even Skipped–Dependent Muscle and Heart Cell Fates Are Required for Normal Adult Activity, Heart Function, and Lifespan. Circ. Res..

[190] Lo P.C.H., Zaffran S., Sénatore S., Frasch M. (2007). The Drosophila Hand Gene Is Required for Remodeling of the Developing Adult Heart and Midgut during Metamorphosis. Dev. Biol..

[191] Han Z., Yi P., Li X., Olson E.N. (2006). Hand, an Evolutionarily Conserved bHLH Transcription Factor Required for Drosophila Cardiogenesis and Hematopoiesis. Development.

[192] Meyer C., Bataillé L., Drechsler M., Paululat A. (2023). Tailup Expression in Larval and Adult Cardiac Valve Cells. Genesis.

[193] Cai C.-L., Liang X., Shi Y., Chu P.-H., Pfaff S.L., Chen J., Evans S. (2003). Isl1 Identifies a Cardiac Progenitor Population That Proliferates Prior to Differentiation and Contributes a Majority of Cells to the Heart. Dev. Cell.

[194] Sun Y., Liang X., Najafi N., Cass M., Lin L., Cai C.-L., Chen J., Evans S.M. (2007). Islet 1 Is Expressed in Distinct Cardiovascular Lineages, Including Pacemaker and Coronary Vascular Cells. Dev. Biol..

[195] Li Y., Du J., Deng S., Liu B., Jing X., Yan Y., Liu Y., Wang J., Zhou X., She Q. (2024). The Molecular Mechanisms of Cardiac Development and Related Diseases. Signal Transduct. Target. Ther..

[196] Witzel H.R., Cheedipudi S., Gao R., Stainier D.Y.R., Dobreva G.D. (2017). Isl2b Regulates Anterior Second Heart Field Development in Zebrafish. Sci. Rep..

[197] Wang Z., Song H.-M., Wang F., Zhao C.-M., Huang R.-T., Xue S., Li R.-G., Qiu X.-B., Xu Y.-J., Liu X.-Y. (2019). A New ISL1 Loss-of-Function Mutation Predisposes to Congenital Double Outlet Right Ventricle. Int. Heart J..

[198] Reim I., Mohler J.P., Frasch M. (2005). *Tbx20*-Related Genes, *Mid* and *H15*, Are Required for *Tinman* Expression, Proper Patterning, and Normal Differentiation of Cardioblasts in *Drosophila*. Mech. Dev..

[199] Qian L., Liu J., Bodmer R. (2005). *Neuromancer* Tbx20-Related Genes (*H15/Midline*) Promote Cell Fate Specification and Morphogenesis of the *Drosophila* Heart. Dev. Biol..

[200] Qian L., Mohapatra B., Akasaka T., Liu J., Ocorr K., Towbin J.A., Bodmer R. (2008). Transcription Factor Neuromancer/TBX20 Is Required for Cardiac Function in Drosophila with Implications for Human Heart Disease. Proc. Natl. Acad. Sci. USA.

[201] Ocorr K., Vogler G., Bodmer R. (2014). Methods to Assess *Drosophila* Heart Development, Function and Aging. Methods.

[202] Stennard F.A., Costa M.W., Elliott D.A., Rankin S., Haast S.J.P., Lai D., McDonald L.P.A., Niederreither K., Dolle P., Bruneau B.G. (2003). Cardiac T-Box Factor Tbx20 Directly Interacts with Nkx2-5, GATA4, and GATA5 in Regulation of Gene Expression in the Developing Heart. Dev. Biol..

[203] Gao X., Yan B. (2023). The Mechanism and Diagnostic Value of Tbx20 in Cardiovascular Diseases. Gene Rep..

[204] Takeuchi J.K., Mileikovskaia M., Koshiba-Takeuchi K., Heidt A.B., Mori A.D., Arruda E.P., Gertsenstein M., Georges R., Davidson L., Mo R. (2005). Tbx20 Dose-Dependently Regulates Transcription Factor Networks Required for Mouse Heart and Motoneuron Development. Development.

[205] Sun Q., Li Q., Qin Z., Wen Y., Liu C. (2024). The Role of TBX20 Gene Mutations in the Pathogenesis of Congenital Heart Disease: Functional Analysis and Genetic Association Study. Cardiology.

[206] Hadjantonakis A.-K., Pisano E., Papaioannou V.E. (2008). Tbx6 Regulates Left/Right Patterning in Mouse Embryos through Effects on Nodal Cilia and Perinodal Signaling. PLoS ONE.

[207] Windner S.E., Doris R.A., Ferguson C.M., Nelson A.C., Valentin G., Tan H., Oates A.C., Wardle F.C., Devoto S.H. (2015). Tbx6, Mesp-b and Ripply1 Regulate the Onset of Skeletal Myogenesis in Zebrafish. Development.

[208] Harrelson Z., Kelly R.G., Goldin S.N., Gibson-Brown J.J., Bollag R.J., Silver L.M., Papaioannou V.E. (2004). Tbx2 Is Essential for Patterning the Atrioventricular Canal and for Morphogenesis of the Outflow Tract during Heart Development. Development.

[209] Sparrow D.B., McInerney-Leo A., Gucev Z.S., Gardiner B., Marshall M., Leo P.J., Chapman D.L., Tasic V., Shishko A., Brown M.A. (2013). Autosomal Dominant Spondylocostal Dysostosis Is Caused by Mutation in TBX6. Hum. Mol. Genet..

[210] Xie H., Hong N., Zhang E., Li F., Sun K., Yu Y. (2019). Identification of Rare Copy Number Variants Associated With Pulmonary Atresia With Ventricular Septal Defect. Front. Genet..

[211] Singh R., Hoogaars W.M., Barnett P., Grieskamp T., Rana M.S., Buermans H., Farin H.F., Petry M., Heallen T., Martin J.F. (2012). Tbx2 and Tbx3 Induce Atrioventricular Myocardial Development and Endocardial Cushion Formation. Cell. Mol. Life Sci..

[212] Xie H., Zhang E., Hong N., Fu Q., Li F., Chen S., Yu Y., Sun K. (2018). Identification of TBX2 and TBX3 Variants in Patients with Conotruncal Heart Defects by Target Sequencing. Hum. Genom..

[213] Goodman F.R., Majewski F., Collins A.L., Scambler P.J. (2002). A 117-Kb Microdeletion Removing HOXD9–HOXD13 and EVX2 Causes Synpolydactyly. Am. J. Hum. Genet..

[214] Yang A., Alankarage D., Cuny H., Ip E.K.K., Almog M., Lu J., Das D., Enriquez A., Szot J.O., Humphreys D.T. (2022). CHDgene: A Curated Database for Congenital Heart Disease Genes. Circ. Genom. Precis. Med..

[215] Dohn T.E., Ravisankar P., Tirera F.T., Martin K.E., Gafranek J.T., Duong T.B., VanDyke T.L., Touvron M., Barske L.A., Crump J.G. (2019). Nr2f-Dependent Allocation of Ventricular Cardiomyocyte and Pharyngeal Muscle Progenitors. PLoS Genet..

[216] Al Turki S., Manickaraj A.K., Mercer C.L., Gerety S.S., Hitz M.-P., Lindsay S., D’Alessandro L.C.A., Swaminathan G.J., Bentham J., Arndt A.-K. (2014). Rare Variants in *NR2F2* Cause Congenital Heart Defects in Humans. Am. J. Hum. Genet..

[217] Mansoor W., Heidari M.M., Khatami M., Hadadzadeh M., Tabrizi F., Darvand Araghi M.H. (2025). Rare Pathogenic *NR2F2* (COUP-TFII) Variants as Potential Etiological Causes in Pediatric Patients with Congenital Heart Diseases (CHDs). Hell. J. Cardiol..

[218] Wang J., Abhinav P., Xu Y.-J., Li R.-G., Zhang M., Qiu X.-B., Di R.-M., Qiao Q., Li X.-M., Huang R.-T. (2019). NR2F2 Loss-of-function Mutation Is Responsible for Congenital Bicuspid Aortic Valve. Int. J. Mol. Med..

[219] George R.M., Firulli B.A., Podicheti R., Rusch D.B., Mannion B.J., Pennacchio L.A., Osterwalder M., Firulli A.B. (2023). Single Cell Evaluation of Endocardial Hand2 Gene Regulatory Networks Reveals HAND2-Dependent Pathways That Impact Cardiac Morphogenesis. Development.

[220] Clapham K.R., Singh I., Capuano I.S., Rajagopal S., Chun H.J. (2019). MEF2 and the Right Ventricle: From Development to Disease. Front. Cardiovasc. Med..

[221] Materna S.C., Sinha T., Barnes R.M., Lammerts van Bueren K., Black B.L. (2019). Cardiovascular Development and Survival Require *Mef2c* Function in the Myocardial but Not the Endothelial Lineage. Dev. Biol..

[222] Li F.-F., Han Y., Shi S., Li X., Zhu X.-D., Zhou J., Shao Q.-L., Li X.-Q., Liu S.-L. (2015). Characterization of Transcriptional Repressor Gene MSX1 Variations for Possible Associations with Congenital Heart Diseases. PLoS ONE.

[223] Jamsheer A., Sowińska A., Simon D., Jamsheer-Bratkowska M., Trzeciak T., Latos-Bieleńska A. (2013). Bilateral Radial Agenesis with Absent Thumbs, Complex Heart Defect, Short Stature, and Facial Dysmorphism in a Patient with Pure Distal Microduplication of 5q35.2-5q35.3. BMC Med. Genet..

[224] Sun Y.-M., Wang J., Qiu X.-B., Yuan F., Li R.-G., Xu Y.-J., Qu X.-K., Shi H.-Y., Hou X.-M., Huang R.-T. (2016). A HAND2 Loss-of-Function Mutation Causes Familial Ventricular Septal Defect and Pulmonary Stenosis. G3 Genes Genomes Genet..

[225] Lu C.-X., Wang W., Wang Q., Liu X.-Y., Yang Y.-Q. (2018). A Novel MEF2C Loss-of-Function Mutation Associated with Congenital Double Outlet Right Ventricle. Pediatr. Cardiol..

[226] Zeng Z.-H., Chen H.-X., Liu X.-C., Yang Q., He G.-W. (2022). Functional Significance of Novel Variants of the MEF2C Gene Promoter in Congenital Ventricular Septal Defects. Am. J. Med. Genet. Part A.

[227] Qiao X.-H., Wang F., Zhang X.-L., Huang R.-T., Xue S., Wang J., Qiu X.-B., Liu X.-Y., Yang Y.-Q. (2017). MEF2C Loss-of-Function Mutation Contributes to Congenital Heart Defects. Int. J. Med. Sci..

[228] Kodo K., Nishizawa T., Furutani M., Arai S., Ishihara K., Oda M., Makino S., Fukuda K., Takahashi T., Matsuoka R. (2012). Genetic Analysis of Essential Cardiac Transcription Factors in 256 Patients With Non-Syndromic Congenital Heart Defects. Circ. J..

[229] Yi P., Han Z., Li X., Olson E.N. (2006). The Mevalonate Pathway Controls Heart Formation in Drosophila by Isoprenylation of Gγ1. Science.

[230] Theis J.L., Vogler G., Missinato M.A., Li X., Nielsen T., Zeng X.-X.I., Martinez-Fernandez A., Walls S.M., Kervadec A., Kezos J.N. (2020). Patient-Specific Genomics and Cross-Species Functional Analysis Implicate LRP2 in Hypoplastic Left Heart Syndrome. eLife.

[231] Riedel F., Vorkel D., Eaton S. (2011). Megalin-Dependent Yellow Endocytosis Restricts Melanization in the Drosophila Cuticle. Development.

[232] Basu M., Zhu J.-Y., LaHaye S., Majumdar U., Jiao K., Han Z., Garg V. (2017). Epigenetic Mechanisms Underlying Maternal Diabetes-Associated Risk of Congenital Heart Disease. JCI Insight.

[233] Schroeder A.M., Allahyari M., Vogler G., Missinato M.A., Nielsen T., Yu M.S., Theis J.L., Larsen L.A., Goyal P., Rosenfeld J.A. (2019). Model System Identification of Novel Congenital Heart Disease Gene Candidates: Focus on RPL13. Hum. Mol. Genet..

[234] Edison R.J., Muenke M. (2004). Central Nervous System and Limb Anomalies in Case Reports of First-Trimester Statin Exposure. N. Engl. J. Med..

[235] Edison R.J., Muenke M. (2005). Gestational Exposure to Lovastatin Followed by Cardiac Malformation Misclassified as Holoprosencephaly. N. Engl. J. Med..

[236] Akasaka T., Ocorr K., Lin L., Vogler G., Bodmer R., Grossfeld P. (2020). Overexpression of Kif1A in the Developing Drosophila Heart Causes Valvar and Contractility Defects: Implications for Human Congenital Heart Disease. J. Cardiovasc. Dev. Dis..

[237] Birker K., Ge S., Kirkland N.J., Theis J.L., Marchant J., Fogarty Z.C., Missinato M.A., Kalvakuri S., Grossfeld P., Engler A.J. (2023). Mitochondrial MICOS Complex Genes, Implicated in Hypoplastic Left Heart Syndrome, Maintain Cardiac Contractility and Actomyosin Integrity. eLife.

[238] Boukhatmi H., Schaub C., Bataillé L., Reim I., Frendo J.-L., Frasch M., Vincent A. (2014). An Org-1–Tup Transcriptional Cascade Reveals Different Types of Alary Muscles Connecting Internal Organs in Drosophila. Development.

[239] Lilly B., Zhao B., Ranganayakulu G., Paterson B.M., Schulz R.A., Olson E.N. (1995). Requirement of MADS Domain Transcription Factor D-MEF2 for Muscle Formation in Drosophila. Science.

[240] Ryan K.M., Hendren J.D., Helander L.A., Cripps R.M. (2007). The NK Homeodomain Transcription Factor Tinman Is a Direct Activator of *Seven-up* in the *Drosophila* Dorsal Vessel. Dev. Biol..

[241] Schaub C., Nagaso H., Jin H., Frasch M. (2012). Org-1, the Drosophila Ortholog of Tbx1, Is a Direct Activator of Known Identity Genes during Muscle Specification. Development.

[242] Pareek G., Thomas R.E., Pallanck L.J. (2018). Loss of the Drosophila M-AAA Mitochondrial Protease Paraplegin Results in Mitochondrial Dysfunction, Shortened Lifespan, and Neuronal and Muscular Degeneration. Cell Death Dis..

[243] He L., Wu B., Shi J., Du J., Zhao Z. (2023). Regulation of Feeding and Energy Homeostasis by Clock-Mediated *Gart* in *Drosophila*. Cell Rep..

[244] Tao Y., Zhang Q., Wang H., Yang X., Mu H. (2024). Alternative Splicing and Related RNA Binding Proteins in Human Health and Disease. Signal Transduct. Target. Ther..

[245] Blockus H., Chédotal A. (2016). Slit-Robo Signaling. Development.

[246] Medioni C., Astier M., Zmojdzian M., Jagla K., Sémériva M. (2008). Genetic Control of Cell Morphogenesis during *Drosophila melanogaster* Cardiac Tube Formation. J. Cell Biol..

[247] Maartens A.P., Brown N.H. (2015). The Many Faces of Cell Adhesion during *Drosophila* Muscle Development. Dev. Biol..

[248] Vanderploeg J., Jacobs J.R. (2017). Mapping Heart Development in Flies: Src42A Acts Non-Autonomously to Promote Heart Tube Formation in Drosophila. Vet. Sci..

[249] Santiago-Martínez E., Soplop N.H., Patel R., Kramer S.G. (2008). Repulsion by Slit and Roundabout Prevents Shotgun/E-Cadherin-Mediated Cell Adhesion during Drosophila Heart Tube Lumen Formation. J. Cell Biol..

[250] Chartier A., Zaffran S., Astier M., Sémériva M., Gratecos D. (2002). Pericardin, a Drosophila Type IV Collagen-like Protein Is Involved in the Morphogenesis and Maintenance of the Heart Epithelium during Dorsal Ectoderm Closure. Development.

[251] Vogler G., Liu J., Iafe T.W., Migh E., Mihály J., Bodmer R. (2014). Cdc42 and Formin Activity Control Non-Muscle Myosin Dynamics during Drosophila Heart Morphogenesis. J. Cell Biol..

[252] Hansen S.D., Mullins R.D. (2015). Lamellipodin Promotes Actin Assembly by Clustering Ena/VASP Proteins and Tethering Them to Actin Filaments. eLife.

[253] Raza Q.S., Vanderploeg J.L., Jacobs J.R. (2017). Matrix Metalloproteinases Are Required for Membrane Motility and Lumenogenesis during Drosophila Heart Development. PLoS ONE.

[254] Hughes C.J.R., Turner S., Andrews R.M., Vitkin A., Jacobs J.R. (2020). Matrix Metalloproteinases Regulate ECM Accumulation but Not Larval Heart Growth in *Drosophila melanogaster*. J. Mol. Cell. Cardiol..

[255] Linask K.K., Han M., Cai D.H., Brauer P.R., Maisastry S.M. (2005). Cardiac Morphogenesis: Matrix Metalloproteinase Coordination of Cellular Mechanisms Underlying Heart Tube Formation and Directionality of Looping. Dev. Dyn..

[256] Verma D., Singh A., Singh J., Mutsuddi M., Mukherjee A. (2024). Regulation of Notch Signaling by Non-Muscle Myosin II Zipper in Drosophila. Cell Mol. Life Sci..

[257] Molnár I., Migh E., Szikora S., Kalmár T., Végh A.G., Deák F., Barkó S., Bugyi B., Orfanos Z., Kovács J. (2014). DAAM Is Required for Thin Filament Formation and Sarcomerogenesis during Muscle Development in Drosophila. PLoS Genet..

[258] Migh E., Götz T., Földi I., Szikora S., Gombos R., Darula Z., Medzihradszky K.F., Maléth J., Hegyi P., Sigrist S. (2018). Microtubule Organization in Presynaptic Boutons Relies on the Formin DAAM. Development.

[259] Gombos R., Migh E., Antal O., Mukherjee A., Jenny A., Mihály J. (2015). The Formin DAAM Functions as Molecular Effector of the Planar Cell Polarity Pathway during Axonal Development in Drosophila. J. Neurosci..

[260] Qian L., Wythe J.D., Liu J., Cartry J., Vogler G., Mohapatra B., Otway R.T., Huang Y., King I.N., Maillet M. (2011). Tinman/Nkx2-5 Acts via miR-1 and Upstream of Cdc42 to Regulate Heart Function across Species. J. Cell Biol..

[261] Kadam S., McMahon A., Tzou P., Stathopoulos A. (2009). FGF Ligands in Drosophila Have Distinct Activities Required to Support Cell Migration and Differentiation. Development.

[262] Dorey K., Amaya E. (2010). FGF Signalling: Diverse Roles during Early Vertebrate Embryogenesis. Development.

[263] Hubert F., Payan S.M., Rochais F. (2018). FGF10 Signaling in Heart Development, Homeostasis, Disease and Repair. Front. Genet..

[264] Hutson M.R., Zeng X.L., Kim A.J., Antoon E., Harward S., Kirby M.L. (2010). Arterial Pole Progenitors Interpret Opposing FGF/BMP Signals to Proliferate or Differentiate. Development.

[265] Wu X., Golden K., Bodmer R. (1995). Heart Development in *Drosophila* Requires the Segment Polarity Gene *Wingless*. Dev. Biol..

[266] Yin Z., Frasch M. (1998). Regulation and Function of Tinman during Dorsal Mesoderm Induction and Heart Specification in Drosophila. Dev. Genet..

[267] Johnson A.N., Burnett L.A., Sellin J., Paululat A., Newfeld S.J. (2007). Defective Decapentaplegic Signaling Results in Heart Overgrowth and Reduced Cardiac Output in Drosophila. Genetics.

[268] Lockwood W.K., Bodmer R. (2002). The Patterns of *Wingless*, *Decapentaplegic*, and *Tinman* Position the *Drosophila* Heart. Mech. Dev..

[269] Mazzotta S., Neves C., Bonner R.J., Bernardo A.S., Docherty K., Hoppler S. (2016). Distinctive Roles of Canonical and Noncanonical Wnt Signaling in Human Embryonic Cardiomyocyte Development. Stem Cell Rep..

[270] Ueno S., Weidinger G., Osugi T., Kohn A.D., Golob J.L., Pabon L., Reinecke H., Moon R.T., Murry C.E. (2007). Biphasic Role for Wnt/β-Catenin Signaling in Cardiac Specification in Zebrafish and Embryonic Stem Cells. Proc. Natl. Acad. Sci. USA.

[271] Cohen E.D., Tian Y., Morrisey E.E. (2008). Wnt Signaling: An Essential Regulator of Cardiovascular Differentiation, Morphogenesis and Progenitor Self-Renewal. Development.

[272] Tanegashima K., Zhao H., Dawid I.B. (2008). WGEF Activates Rho in the Wnt–PCP Pathway and Controls Convergent Extension in Xenopus Gastrulation. EMBO J..

[273] Shi Y., Katsev S., Cai C., Evans S. (2000). BMP Signaling Is Required for Heart Formation in Vertebrates. Dev. Biol..

[274] Vasudevarao M.D., Posadas Pena D., Ihle M., Bongiovanni C., Maity P., Geissler D., Mohammadi H.F., Rall-Scharpf M., Niemann J., Mommersteeg M.T.M. (2025). BMP Signaling Promotes Zebrafish Heart Regeneration via Alleviation of Replication Stress. Nat. Commun..

[275] Bhanot P., Fish M., Jemison J.A., Nusse R., Nathans J., Cadigan K.M. (1999). Frizzled and DFrizzled-2 Function as Redundant Receptors for Wingless during Drosophila Embryonic Development. Development.

[276] Chen C., Struhl G. (1999). Wingless Transduction by the Frizzled and Frizzled2 Proteins of Drosophila. Development.

[277] Chen Z., Zhu J., Fu Y., Richman A., Han Z. (2016). Wnt4 Is Required for Ostia Development in the *Drosophila* Heart. Dev. Biol..

[278] Trujillo G.V., Nodal D.H., Lovato C.V., Hendren J.D., Helander L.A., Lovato T.L., Bodmer R., Cripps R.M. (2016). The Canonical Wingless Signaling Pathway Is Required but Not Sufficient for Inflow Tract Formation in the *Drosophila melanogaster* Heart. Dev. Biol..

[279] Schleiffarth J.R., Person A.D., Martinsen B.J., Sukovich D.J., Neumann A., Baker C.V.H., Lohr J.L., Cornfield D.N., Ekker S.C., Petryk A. (2007). Wnt5a Is Required for Cardiac Outflow Tract Septation in Mice. Pediatr. Res..

[280] van Vliet P.P., Lin L., Boogerd C.J., Martin J.F., Andelfinger G., Grossfeld P.D., Evans S.M. (2017). Tissue Specific Requirements for WNT11 in Developing Outflow Tract and Dorsal Mesenchymal Protrusion. Dev. Biol..

[281] Touma M., Kang X., Gao F., Zhao Y., Cass A.A., Biniwale R., Xiao X., Eghbali M., Coppola G., Reemtsen B. (2017). Wnt11 Regulates Cardiac Chamber Development and Disease during Perinatal Maturation. JCI Insight.

[282] Mohamed I.A., El-Badri N., Zaher A. (2019). Wnt Signaling: The Double-Edged Sword Diminishing the Potential of Stem Cell Therapy in Congenital Heart Disease. Life Sci..

[283] Von Ohlen T., Hooper J.E. (1997). Hedgehog Signaling Regulates Transcription through Gli/Ci Binding Sites in the *Wingless* Enhancer. Mech. Dev..

[284] Liu J., Qian L., Wessells R.J., Bidet Y., Jagla K., Bodmer R. (2006). Hedgehog and RAS Pathways Cooperate in the Anterior–Posterior Specification and Positioning of Cardiac Progenitor Cells. Dev. Biol..

[285] Rowton M., Perez-Cervantes C., Hur S., Jacobs-Li J., Lu E., Deng N., Guzzetta A., Hoffmann A.D., Stocker M., Steimle J.D. (2022). Hedgehog Signaling Activates a Mammalian Heterochronic Gene Regulatory Network Controlling Differentiation Timing across Lineages. Dev. Cell.

[286] Inoue S., Nosetani M., Nakajima Y., Sakaki S., Kato H., Saba R., Takeshita N., Nishikawa K., Ueyama A., Matsuo K. (2025). Sonic Hedgehog Signaling Regulates the Optimal Differentiation Pace from Early-Stage Mesoderm to Cardiogenic Mesoderm in Mice. Dev. Growth Differ..

[287] Constable S., Mukhopadhyay S. (2020). Ubiquitin Tunes Hedgehog in Matters of the Heart. Dev. Cell.

[288] Sanchez-Soria P., Camenisch T.D. (2010). ErbB Signaling in Cardiac Development and Disease. Semin. Cell Dev. Biol..

[289] MacGrogan D., Münch J., de la Pompa J.L. (2018). Notch and Interacting Signalling Pathways in Cardiac Development, Disease, and Regeneration. Nat. Rev. Cardiol..

[290] Bray S., Furriols M. (2001). Notch Pathway: Making Sense of Suppressor of Hairless. Curr. Biol..

[291] Rones M.S., McLaughlin K.A., Raffin M., Mercola M. (2000). Serrate and Notch Specify Cell Fates in the Heart Field by Suppressing Cardiomyogenesis. Development.

[292] Chau M.D.L., Tuft R., Fogarty K., Bao Z.-Z. (2006). Notch Signaling Plays a Key Role in Cardiac Cell Differentiation. Mech. Dev..

[293] Zhao L., Borikova A.L., Ben-Yair R., Guner-Ataman B., MacRae C.A., Lee R.T., Burns C.G., Burns C.E. (2014). Notch Signaling Regulates Cardiomyocyte Proliferation during Zebrafish Heart Regeneration. Proc. Natl. Acad. Sci. USA.

[294] Kwon C., Qian L., Cheng P., Nigam V., Arnold J., Srivastava D. (2009). A Regulatory Pathway Involving Notch1/β-Catenin/Isl1 Determines Cardiac Progenitor Cell Fate. Nat. Cell Biol..

[295] Park E.J., Watanabe Y., Smyth G., Miyagawa-Tomita S., Meyers E., Klingensmith J., Camenisch T., Buckingham M., Moon A.M. (2008). An FGF Autocrine Loop Initiated in Second Heart Field Mesoderm Regulates Morphogenesis at the Arterial Pole of the Heart. Development.

[296] Gajewski K., Choi C.Y., Kim Y., Schulz R.A. (2000). Genetically Distinct Cardial Cells within the Drosophila Heart. Genesis.

[297] Salomone J., Farrow E., Gebelein B. (2024). Homeodomain Complex Formation and Biomolecular Condensates in Hox Gene Regulation. Semin. Cell Dev. Biol..

[298] Wellik D.M. (2024). Hox Genes and Patterning the Vertebrate Body. Current Topics in Developmental Biology.

[299] Holland P.W., Booth H.A.F., Bruford E.A. (2007). Classification and Nomenclature of All Human Homeobox Genes. BMC Biol..

[300] Hubert K.A., Wellik D.M. (2023). Hox Genes in Development and Beyond. Development.

[301] Deschamps J., Duboule D. (2017). Embryonic Timing, Axial Stem Cells, Chromatin Dynamics, and the Hox Clock. Genes Dev..

[302] Epstein M., Pillemer G., Yelin R., Yisraeli J.K., Fainsod A. (1997). Patterning of the Embryo along the Anterior-Posterior Axis: The Role of the Caudal Genes. Development.

[303] Maeda R.K., Karch F. (2009). Chapter 1 The Bithorax Complex of *Drosophila*. Current Topics in Developmental Biology.

[304] Rosales-Vega M., Hernández-Becerril A., Murillo-Maldonado J.M., Zurita M., Vázquez M. (2018). The Role of the Trithorax Group TnaA Isoforms in Hox Gene Expression, and in Drosophila Late Development. PLoS ONE.

[305] Mark M., Rijli F.M., Chambon P. (1997). Homeobox Genes in Embryogenesis and Pathogenesis. Pediatr. Res..

[306] Mulhair P.O., Holland P.W.H. (2024). Evolution of the Insect Hox Gene Cluster: Comparative Analysis across 243 Species. Semin. Cell Dev. Biol..

[307] Rosales-Vega M., Reséndez-Pérez D., Vázquez M. (2024). Antennapedia: The Complexity of a Master Developmental Transcription Factor. Genesis.

[308] Perrin L., Monier B., Ponzielli R., Astier M., Semeriva M. (2004). *Drosophila* Cardiac Tube Organogenesis Requires Multiple Phases of Hox Activity. Dev. Biol..

[309] Ponzielli R., Astier M., Chartier A., Gallet A., Thérond P., Sémériva M. (2002). Heart Tube Patterning in Drosophila Requires Integration of Axial and Segmental Information Provided by the Bithorax Complex Genes and Hedgehog Signaling. Development.

[310] Maeda R.K., Karch F. (2006). The ABC of the BX-C: The Bithorax Complex Explained. Development.

[311] LaBeau E.M., Trujillo D.L., Cripps R.M. (2009). Bithorax Complex Genes Control Alary Muscle Patterning along the Cardiac Tube of *Drosophila*. Mech. Dev..

[312] Schroeder A.M., Nielsen T., Lynott M., Vogler G., Colas A.R., Bodmer R. (2022). Nascent Polypeptide-Associated Complex and Signal Recognition Particle Have Cardiac-Specific Roles in Heart Development and Remodeling. PLoS Genet..

[313] Ryan K.M., Hoshizaki D.K., Cripps R.M. (2005). Homeotic Selector Genes Control the Patterning of *Seven-up* Expressing Cells in the *Drosophila* Dorsal Vessel. Mech. Dev..

[314] Duboule D. (2007). The Rise and Fall of Hox Gene Clusters. Development.

[315] Lescroart F., Wang X., Lin X., Swedlund B., Gargouri S., Sànchez-Dànes A., Moignard V., Dubois C., Paulissen C., Kinston S. (2018). Defining the Earliest Step of Cardiovascular Lineage Segregation by Single-Cell RNA-Seq. Science.

[316] Makki N., Capecchi M.R. (2012). Cardiovascular Defects in a Mouse Model of HOXA1 Syndrome. Hum. Mol. Genet..

[317] Tischfield M.A., Bosley T.M., Salih M.A.M., Alorainy I.A., Sener E.C., Nester M.J., Oystreck D.T., Chan W.-M., Andrews C., Erickson R.P. (2005). Homozygous HOXA1 Mutations Disrupt Human Brainstem, Inner Ear, Cardiovascular and Cognitive Development. Nat. Genet..

[318] Roux M., Laforest B., Capecchi M., Bertrand N., Zaffran S. (2015). *Hoxb1* Regulates Proliferation and Differentiation of Second Heart Field Progenitors in Pharyngeal Mesoderm and Genetically Interacts with *Hoxa1* during Cardiac Outflow Tract Development. Dev. Biol..

[319] Roux M., Laforest B., Eudes N., Bertrand N., Stefanovic S., Zaffran S. (2017). *Hoxa1* and *Hoxb1* Are Required for Pharyngeal Arch Artery Development. Mech. Dev..

[320] Lufkin T., Dierich A., LeMeur M., Mark M., Chambon P. (1991). Disruption of the Hox-1.6 Homeobox Gene Results in Defects in a Region Corresponding to Its Rostral Domain of Expression. Cell.

[321] Godwin A.R., Stadler H.S., Nakamura K., Capecchi M.R. (1998). Detection of Targeted GFP-Hox Gene Fusions during Mouse Embryogenesis. Proc. Natl. Acad. Sci. USA.

[322] Makki N., Capecchi M.R. (2010). Hoxa1 Lineage Tracing Indicates a Direct Role for Hoxa1 in the Development of the Inner Ear, the Heart, and the Third Rhombomere. Dev. Biol..

[323] Chisaka O., Capecchi M.R. (1991). Regionally Restricted Developmental Defects Resulting from Targeted Disruption of the Mouse Homeobox Gene Hox-1.5. Nature.

[324] Kameda Y., Watari-Goshima N., Nishimaki T., Chisaka O. (2003). Disruption of the Hoxa3 Homeobox Gene Results in Anomalies of the Carotid Artery System and the Arterial Baroreceptors. Cell Tissue Res..

[325] Chisaka O., Kameda Y. (2005). Hoxa3 Regulates the Proliferation and Differentiation of the Third Pharyngeal Arch Mesenchyme in Mice. Cell Tissue Res..

[326] Sharifi-Zarchi A., Gerovska D., Adachi K., Totonchi M., Pezeshk H., Taft R.J., Schöler H.R., Chitsaz H., Sadeghi M., Baharvand H. (2017). DNA Methylation Regulates Discrimination of Enhancers from Promoters through a H3K4me1-H3K4me3 Seesaw Mechanism. BMC Genom..

[327] Zhu J., Liu C., Huang X., van de Leemput J., Lee H., Han Z. (2023). H3K36 Di-Methylation Marks, Mediated by Ash1 in Complex with Caf1-55 and MRG15, Are Required during Drosophila Heart Development. J. Cardiovasc. Dev. Dis..

[328] Zhu J.-Y., van de Leemput J., Han Z. (2024). Distinct Roles of COMPASS Subunits to Drosophila Heart Development. Biol. Open.

[329] Krogan N.J., Dover J., Khorrami S., Greenblatt J.F., Schneider J., Johnston M., Shilatifard A. (2002). COMPASS, a Histone H3 (Lysine 4) Methyltransferase Required for Telomeric Silencing of Gene Expression. J. Biol. Chem..

[330] Huang W., Zhu J., Fu Y., van de Leemput J., Han Z. (2022). *Lpt*, *Trr*, and *Hcf* Regulate Histone Mono- and Dimethylation That Are Essential for *Drosophila* Heart Development. Dev. Biol..

[331] Zhu J.-Y., Lee H., Huang X., van de Leemput J., Han Z. (2023). Distinct Roles for COMPASS Core Subunits Set1, Trx, and Trr in the Epigenetic Regulation of Drosophila Heart Development. Int. J. Mol. Sci..

[332] Borland S., Tenin G., Williams S., Monaghan R., Baxter M., Ray D., Abraham S., Keavney B. (2019). BS9 KMT2C—A Tetralogy of Fallot Candidate Gene. Heart.

[333] Ang S.-Y., Uebersohn A., Spencer C.I., Huang Y., Lee J.-E., Ge K., Bruneau B.G. (2016). KMT2D Regulates Specific Programs in Heart Development via Histone H3 Lysine 4 Di-Methylation. Development.

[334] Yuan S.-M. (2013). Congenital Heart Defects in Kabuki Syndrome. Cardiol. J..

[335] Rabkin S.W., Wong C.N. (2023). Epigenetics in Heart Failure: Role of DNA Methylation in Potential Pathways Leading to Heart Failure with Preserved Ejection Fraction. Biomedicines.

[336] Liu P., Zhang J., Du D., Zhang D., Jin Z., Qiu W., Zhou X., Dong S., Zhou M., Zhao H. (2021). Altered DNA Methylation Pattern Reveals Epigenetic Regulation of Hox Genes in Thoracic Aortic Dissection and Serves as a Biomarker in Disease Diagnosis. Clin. Epigenetics.

[337] Zhou Y., Wu Q., Guo Y. (2024). Deciphering the Emerging Landscape of HOX Genes in Cardiovascular Biology, Atherosclerosis and beyond (Review). Int. J. Mol. Med..

[338] Behrens A.N., Iacovino M., Lohr J.L., Ren Y., Zierold C., Harvey R.P., Kyba M., Garry D.J., Martin C.M. (2013). Nkx2-5 Mediates Differential Cardiac Differentiation through Interaction with Hoxa10. Stem Cells Dev..

[339] Diman N.Y.S.-G., Remacle S., Bertrand N., Picard J.J., Zaffran S., Rezsohazy R. (2011). A Retinoic Acid Responsive Hoxa3 Transgene Expressed in Embryonic Pharyngeal Endoderm, Cardiac Neural Crest and a Subdomain of the Second Heart Field. PLoS ONE.

[340] Jun K.R., Seo E.-J., Lee J.-O., Yoo H.-W., Park I.-S., Yoon H.-K. (2011). Molecular Cytogenetic and Clinical Characterization of a Patient with a 5.6-Mb Deletion in 7p15 Including HOXA Cluster. Am. J. Med. Genet. Part A.

[341] Munabi N.C.O., Mikhail S., Toubat O., Webb M., Auslander A., Sanchez-Lara P.A., Manojlovic Z., Schmidt R.J., Craig D., Magee W.P. (2022). High Prevalence of Deleterious Mutations in Concomitant Nonsyndromic Cleft and Outflow Tract Heart Defects. Am. J. Med. Genet. Part A.

[342] Smedts H.P.M., van Uitert E.M., Valkenburg O., Laven J.S.E., Eijkemans M.J.C., Lindemans J., Steegers E.A.P., Steegers-Theunissen R.P.M. (2012). A Derangement of the Maternal Lipid Profile Is Associated with an Elevated Risk of Congenital Heart Disease in the Offspring. Nutr. Metab. Cardiovasc. Dis..

[343] Su M.-T., Venkatesh T.V., Wu X., Golden K., Bodmer R. (1999). The Pioneer Gene, *Apontic*, Is Required for Morphogenesis and Function of the *Drosophila* Heart. Mech. Dev..

[344] Liu Q.-X., Wang X.-F., Ikeo K., Hirose S., Gehring W.J., Gojobori T. (2014). Evolutionarily Conserved Transcription Factor Apontic Controls the G1/S Progression by Inducing Cyclin E during Eye Development. Proc. Natl. Acad. Sci. USA.

[345] Gregory G.D., Vakoc C.R., Rozovskaia T., Zheng X., Patel S., Nakamura T., Canaani E., Blobel G.A. (2007). Mammalian ASH1L Is a Histone Methyltransferase That Occupies the Transcribed Region of Active Genes. Mol. Cell Biol..

[346] Ji W., Ferdman D., Copel J., Scheinost D., Shabanova V., Brueckner M., Khokha M.K., Ment L.R. (2020). De Novo Damaging Variants Associated with Congenital Heart Diseases Contribute to the Connectome. Sci. Rep..

[347] Jin S.C., Homsy J., Zaidi S., Lu Q., Morton S., DePalma S.R., Zeng X., Qi H., Chang W., Sierant M.C. (2017). Contribution of Rare Inherited and de Novo Variants in 2,871 Congenital Heart Disease Probands. Nat. Genet..

[348] Homsy J., Zaidi S., Shen Y., Ware J.S., Samocha K.E., Karczewski K.J., DePalma S.R., McKean D., Wakimoto H., Gorham J. (2015). De Novo Mutations in Congenital Heart Disease with Neurodevelopmental and Other Birth Defects. Science.

[349] Stoller J.Z., Huang L., Tan C.C., Huang F., Zhou D.D., Yang J., Gelb B.D., Epstein J.A. (2010). Ash2l Interacts with Tbx1 and Is Required during Early Embryogenesis. Exp. Biol. Med..

[350] Barish S., Berg K., Drozd J., Berglund-Brown I., Khizir L., Wasson L.K., Seidman C.E., Seidman J.G., Chen S., Brueckner M. (2023). The H2Bub1-Deposition Complex Is Required for Human and Mouse Cardiogenesis. Development.

[351] VanDusen N.J., Lee J.Y., Gu W., Butler C.E., Sethi I., Zheng Y., King J.S., Zhou P., Suo S., Guo Y. (2021). Massively Parallel in Vivo CRISPR Screening Identifies RNF20/40 as Epigenetic Regulators of Cardiomyocyte Maturation. Nat. Commun..

[352] Robson A., Makova S.Z., Barish S., Zaidi S., Mehta S., Drozd J., Jin S.C., Gelb B.D., Seidman C.E., Chung W.K. (2019). Histone H2B Monoubiquitination Regulates Heart Development via Epigenetic Control of Cilia Motility. Proc. Natl. Acad. Sci. USA.

[353] Liu Y., Wang J., Li J., Wang R., Tharakan B., Zhang S.L., Tong C.W., Peng X. (2017). Deletion of Cdc42 in Embryonic Cardiomyocytes Results in Right Ventricle Hypoplasia. Clin. Transl. Med..

[354] Li J., Liu Y., Jin Y., Wang R., Wang J., Lu S., VanBuren V., Dostal D.E., Zhang S.L., Peng X. (2017). Essential Role of Cdc42 in Cardiomyocyte Proliferation and Cell-Cell Adhesion during Heart Development. Dev. Biol..

[355] Liu Y., Jin Y., Li J., Seto E., Kuo E., Yu W., Schwartz R.J., Blazo M., Zhang S.L., Peng X. (2013). Inactivation of Cdc42 in Neural Crest Cells Causes Craniofacial and Cardiovascular Morphogenesis Defects. Dev. Biol..

[356] Martinelli S., Krumbach O.H.F., Pantaleoni F., Coppola S., Amin E., Pannone L., Nouri K., Farina L., Dvorsky R., Lepri F. (2018). Functional Dysregulation of CDC42 Causes Diverse Developmental Phenotypes. Am. J. Hum. Genet..

[357] Quinzii C.M., Garone C., Emmanuele V., Tadesse S., Krishna S., Dorado B., Hirano M. (2013). Tissue-Specific Oxidative Stress and Loss of Mitochondria in CoQ-Deficient Pdss2 Mutant Mice. FASEB J..

[358] Iványi B., Rácz G.Z., Gál P., Brinyiczki K., Bódi I., Kalmár T., Maróti Z., Bereczki C. (2018). Diffuse Mesangial Sclerosis in a PDSS2 Mutation-Induced Coenzyme Q10 Deficiency. Pediatr. Nephrol..

[359] Murphy N.P., Lubbers E.R., Mohler P.J. (2020). Advancing Our Understanding of AnkRD1 in Cardiac Development and Disease. Cardiovasc. Res..

[360] Almontashiri N.A.M., Chen H.-H., Mailloux R.J., Tatsuta T., Teng A.C.T., Mahmoud A.B., Ho T., Stewart N.A.S., Rippstein P., Harper M.E. (2014). SPG7 Variant Escapes Phosphorylation-Regulated Processing by AFG3L2, Elevates Mitochondrial ROS, and Is Associated with Multiple Clinical Phenotypes. Cell Rep..

[361] Lin Q., Schwarz J., Bucana C., N Olson E. (1997). Control of Mouse Cardiac Morphogenesis and Myogenesis by Transcription Factor MEF2C. Science.

[362] Pavone P., Falsaperla R., Ruggieri M., Marino S.D., Parano E., Pappalardo X.G. (2023). A Young Boy with 21q21.1 Microdeletion Showing Speech Delay, Spastic Diplegia, and MRI Abnormalities: Original Case Report. Glob. Med. Genet..

[363] Weisfeld-Adams J.D., Tkachuk A.K., Maclean K.N., Meeks N.L., Scott S.A. (2016). A de Novo 2.78-Mb Duplication on Chromosome 21q22.11 Implicates Candidate Genes in the Partial Trisomy 21 Phenotype. npj Genom. Med..

[364] Chapman D.L., Cooper-Morgan A., Harrelson Z., Papaioannou V.E. (2003). Critical Role for *Tbx6* in Mesoderm Specification in the Mouse Embryo. Mech. Dev..

[365] Peralta T.M., Zelarayán L.C. (2025). Dot1L-H3K79me2-Tbx6 Axis: A Novel Therapeutic Target for Preventing Cardiac Failure. Circ. Res..

[366] Ma L., Lu M.-F., Schwartz R.J., Martin J.F. (2005). Bmp2 Is Essential for Cardiac Cushion Epithelial-Mesenchymal Transition and Myocardial Patterning. Development.

[367] Bobos D., Soufla G., Angouras D.C., Lekakis I., Georgopoulos S., Melissari E. (2023). Investigation of the Role of BMP2 and -4 in ASD, VSD and Complex Congenital Heart Disease. Diagnostics.

[368] Dunlevy L., Bennett M., Slender A., Lana-Elola E., Tybulewicz V.L., Fisher E.M.C., Mohun T. (2010). Down’s Syndrome-like Cardiac Developmental Defects in Embryos of the Transchromosomic Tc1 Mouse. Cardiovasc. Res..

[369] Grossman T.R., Gamliel A., Wessells R.J., Taghli-Lamallem O., Jepsen K., Ocorr K., Korenberg J.R., Peterson K.L., Rosenfeld M.G., Bodmer R. (2011). Over-Expression of DSCAM and COL6A2 Cooperatively Generates Congenital Heart Defects. PLoS Genet..

[370] Raza Q., Jacobs J.R. (2016). Guidance Signalling Regulates Leading Edge Behaviour during Collective Cell Migration of Cardiac Cells in *Drosophila*. Dev. Biol..

[371] Mollo N., Scognamiglio R., Conti A., Paladino S., Nitsch L., Izzo A. (2023). Genetics and Molecular Basis of Congenital Heart Defects in Down Syndrome: Role of Extracellular Matrix Regulation. Int. J. Mol. Sci..

[372] Brown G.S., Jang J., Li D. (2023). Growth Factors and Their Roles in Cardiac Development and Regeneration: A Narrative Review. Pediatr. Med..

[373] Iwamoto R., Mine N., Mizushima H., Mekada E. (2017). ErbB1 and ErbB4 Generate Opposing Signals Regulating Mesenchymal Cell Proliferation during Valvulogenesis. J. Cell Sci..

[374] McBride K.L., Zender G.A., Fitzgerald–Butt S.M., Seagraves N.J., Fernbach S.D., Zapata G., Lewin M., Towbin J.A., Belmont J.W. (2011). Association of Common Variants in ERBB4 with Congenital Left Ventricular Outflow Tract Obstruction Defects. Birth Defects Res. Part A Clin. Mol. Teratol..

[375] Giannakou A., Sicko R.J., Kay D.M., Zhang W., Romitti P.A., Caggana M., Shaw G.M., Jelliffe-Pawlowski L.L., Mills J.L. (2018). Copy Number Variants in Hypoplastic Right Heart Syndrome. Am. J. Med. Genet. Part A.

[376] Foth R., Shomroni O., Sigler M., Hörer J., Cleuziou J., Paul T., Eildermann K. (2021). Screening for Potential Targets to Reduce Stenosis in Bioprosthetic Heart Valves. Sci. Rep..

[377] Alrefaei A.F. (2021). Frizzled Receptors (FZD) Play Multiple Cellular Roles in Development, in Diseases, and as Potential Therapeutic Targets. J. King Saud. Univ. Sci..

[378] Yu H., Smallwood P.M., Wang Y., Vidaltamayo R., Reed R., Nathans J. (2010). Frizzled 1 and Frizzled 2 Genes Function in Palate, Ventricular Septum and Neural Tube Closure: General Implications for Tissue Fusion Processes. Development.

[379] Li C.-M., Guo M., Salas M., Schupf N., Silverman W., Zigman W.B., Husain S., Warburton D., Thaker H., Tycko B. (2006). Cell Type-Specific over-Expression of Chromosome 21 Genes in Fibroblasts and Fetal Hearts with Trisomy 21. BMC Med. Genet..

[380] Eisa-Beygi S., Hatch G., Noble S., Ekker M., Moon T.W. (2013). The 3-Hydroxy-3-Methylglutaryl-CoA Reductase (HMGCR) Pathway Regulates Developmental Cerebral-Vascular Stability via Prenylation-Dependent Signalling Pathway. Dev. Biol..

[381] Holm A., Graus M.S., Wylie-Sears J., Tan J.W.H., Alvarez-Harmon M., Borgelt L., Nasim S., Chung L., Jain A., Sun M. (2025). An Endothelial SOX18–Mevalonate Pathway Axis Enables Repurposing of Statins for Infantile Hemangioma. J. Clin. Invest..

[382] Nishimura S., Mishra-Gorur K., Park J., Surovtseva Y.V., Sebti S.M., Levchenko A., Louvi A., Gunel M. (2017). Combined HMG-COA Reductase and Prenylation Inhibition in Treatment of CCM. Proc. Natl. Acad. Sci. USA.

[383] Torregrosa-Carrión R., Piñeiro-Sabarís R., Siguero-Álvarez M., Grego-Bessa J., Luna-Zurita L., Fernandes V.S., MacGrogan D., Stainier D.Y.R., de la Pompa J.L. (2021). Adhesion G Protein-Coupled Receptor Gpr126/Adgrg6 Is Essential for Placental Development. Sci. Adv..

[384] Patel M.V., Zhu J., Jiang Z., Richman A., VanBerkum M.F.A., Han Z. (2016). Gia/Mthl5 Is an Aorta Specific GPCR Required for *Drosophila* Heart Tube Morphology and Normal Pericardial Cell Positioning. Dev. Biol..

[385] Lu S., Liu S., Wietelmann A., Kojonazarov B., Atzberger A., Tang C., Schermuly R.T., Gröne H.-J., Offermanns S. (2017). Developmental Vascular Remodeling Defects and Postnatal Kidney Failure in Mice Lacking Gpr116 (Adgrf5) and Eltd1 (Adgrl4). PLoS ONE.

[386] Tanaka K., Chen M., Prendergast A., Zhuang Z., Nasiri A., Joshi D., Hintzen J., Chung M., Kumar A., Mani A. (2024). Latrophilin-2 Mediates Fluid Shear Stress Mechanotransduction at Endothelial Junctions. EMBO J..

[387] Chiba Y., Yoshizaki K., Saito K., Ikeuchi T., Iwamoto T., Rhodes C., Nakamura T., de Vega S., Morell R.J., Boger E.T. (2020). G Protein-Coupled Receptor Gpr115 (Adgrf4) Is Required for Enamel Mineralization Mediated by Ameloblasts. J. Biol. Chem..

[388] Vitobello A., Mazel B., Lelianova V.G., Zangrandi A., Petitto E., Suckling J., Salpietro V., Meyer R., Elbracht M., Kurth I. (2022). ADGRL1 Haploinsufficiency Causes a Variable Spectrum of Neurodevelopmental Disorders in Humans and Alters Synaptic Activity and Behavior in a Mouse Model. Am. J. Hum. Genet..

[389] Oliveira F.G., Rosa-E-Silva J.C., Gomes A.G., Grzesiuk J.D., Vidotto T., Squire J.A., Panepucci R.A., Meola J., Martelli L. (2024). Identification of a Rare Copy Number Polymorphic Gain at 3q12.2 with Candidate Genes for Familial Endometriosis. Rev. Bras. Ginecol. Obs..

[390] Tan M.-Q., Tang Y. (2021). Gene mutations in congenital bilateral absence of the vas deferens: An update. Zhonghua Nan Ke Xue.

[391] Vidal O.M., Vélez J.I., Arcos-Burgos M. (2022). ADGRL3 Genomic Variation Implicated in Neurogenesis and ADHD Links Functional Effects to the Incretin Polypeptide GIP. Sci. Rep..

[392] Quintana A.M., Geiger E.A., Achilly N., Rosenblatt D.S., Maclean K.N., Stabler S.P., Artinger K.B., Appel B., Shaikh T.H. (2014). *Hcfc1b*, a Zebrafish Ortholog of *HCFC1*, Regulates Craniofacial Development by Modulating *Mmachc* Expression. Dev. Biol..

[393] Yu H.-C., Sloan J.L., Scharer G., Brebner A., Quintana A.M., Achilly N.P., Manoli I., Coughlin C.R., Geiger E.A., Schneck U. (2013). An X-Linked Cobalamin Disorder Caused by Mutations in Transcriptional Coregulator *HCFC1*. Am. J. Hum. Genet..

[394] Reynolds J.J., Bicknell L.S., Carroll P., Higgs M.R., Shaheen R., Murray J.E., Papadopoulos D.K., Leitch A., Murina O., Tarnauskaitė Ž. (2017). Mutations in DONSON Disrupt Replication Fork Stability and Cause Microcephalic Dwarfism. Nat. Genet..

[395] Washington Smoak I., Byrd N.A., Abu-Issa R., Goddeeris M.M., Anderson R., Morris J., Yamamura K., Klingensmith J., Meyers E.N. (2005). *Sonic Hedgehog* Is Required for Cardiac Outflow Tract and Neural Crest Cell Development. Dev. Biol..

[396] Dell’Era P., Ronca R., Coco L., Nicoli S., Metra M., Presta M. (2003). Fibroblast Growth Factor Receptor-1 Is Essential for In Vitro Cardiomyocyte Development. Circ. Res..

[397] Stoll C., Alembik Y., Dott B., Roth M.-P. (2022). Associated Anomalies in Cases with Achondroplasia. Eur. J. Med. Genet..

[398] Marguerie A., Bajolle F., Zaffran S., Brown N.A., Dickson C., Buckingham M.E., Kelly R.G. (2006). Congenital Heart Defects in Fgfr2-IIIb and Fgf10 Mutant Mice. Cardiovasc. Res..

[399] Vega-Hernández M., Kovacs A., De Langhe S., Ornitz D.M. (2011). FGF10/FGFR2b Signaling Is Essential for Cardiac Fibroblast Development and Growth of the Myocardium. Development.

[400] Mysliwiec M.R., Bresnick E., Lee Y. (2010). Abstract 21584: Jarid2/Jumonji Dependent Epigenetic Control of Notch1 Expression Is Required for Normal Cardiac Development. Circulation.

[401] van der Laan L., Rooney K., Haghshenas S., Silva A., McConkey H., Relator R., Levy M.A., Valenzuela I., Trujillano L., Lasa-Aranzasti A. (2023). Functional Insight into and Refinement of the Genomic Boundaries of the JARID2-Neurodevelopmental Disorder Episignature. Int. J. Mol. Sci..

[402] Bajpai R., Chen D.A., Rada-Iglesias A., Zhang J., Xiong Y., Helms J., Chang C.-P., Zhao Y., Swigut T., Wysocka J. (2010). CHD7 Cooperates with PBAF to Control Multipotent Neural Crest Formation. Nature.

[403] Han P., Hang C.T., Yang J., Chang C.-P. (2011). Chromatin Remodeling in Cardiovascular Development and Physiology. Circ. Res..

[404] Zhang C., Tian L., Chi C., Wu X., Yang X., Han M., Xu T., Zhuang Y., Deng K. (2010). Adam10 Is Essential for Early Embryonic Cardiovascular Development. Dev. Dyn..

[405] Farber G., Parks M.M., Lustgarten Guahmich N., Zhang Y., Monette S., Blanchard S.C., Di Lorenzo A., Blobel C.P. (2019). ADAM10 Controls the Differentiation of the Coronary Arterial Endothelium. Angiogenesis.

[406] Albrecht S., Wang S., Holz A., Bergter A., Paululat A. (2006). The ADAM Metalloprotease Kuzbanian Is Crucial for Proper Heart Formation in *Drosophila melanogaster*. Mech. Dev..

[407] Xie Y., Ma A., Wang B., Peng R., Jing Y., Wang D., Finnell R.H., Qiao B., Wang Y., Wang H. (2019). Rare Mutations of ADAM17 from TOFs Induce Hypertrophy in Human Embryonic Stem Cell-Derived Cardiomyocytes via HB-EGF Signaling. Clin Sci.

[408] Kaimori J.-Y., Kikkawa Y., Motooka D., Namba-Hamano T., Takuwa A., Okazaki A., Kobayashi K., Tanigawa A., Kotani Y., Uno Y. (2022). A Heterozygous *LAMA5* Variant May Contribute to Slowly Progressive, Vinculin-Enhanced Familial FSGS and Pulmonary Defects. JCI Insight.

[409] Stark K.A., Yee G.H., Roote C.E., Williams E.L., Zusman S., Hynes R.O. (1997). A Novel α Integrin Subunit Associates with β PS and Functions in Tissue Morphogenesis and Movement during Drosophila Development. Development.

[410] Nishiyama M., Takase M., Tanaka Y., Gamo S. (2005). Ether-Resistant Mutant of Laminin Alpha Subunit (*LanA*) in *Drosophila melanogaster*. Int. Congr. Ser..

[411] Deogharia M., Venegas-Zamora L., Agrawal A., Shi M., Jain A.K., McHugh K.J., Altamirano F., Marian A.J., Gurha P. (2024). Histone Demethylase KDM5 Regulates Cardiomyocyte Maturation by Promoting Fatty Acid Oxidation, Oxidative Phosphorylation, and Myofibrillar Organization. Cardiovasc. Res..

[412] Szot J.O., Cuny H., Blue G.M., Humphreys D.T., Ip E., Harrison K., Sholler G.F., Giannoulatou E., Leo P., Duncan E.L. (2018). A Screening Approach to Identify Clinically Actionable Variants Causing Congenital Heart Disease in Exome Data. Circ. Genom. Precis. Med..

[413] Zhu J., van de Leemput J., Han Z. (2023). The Roles of Histone Lysine Methyltransferases in Heart Development and Disease. J. Cardiovasc. Dev. Dis..

[414] Sun H., Yi T., Hao X., Yan H., Wang J., Li Q., Gu X., Zhou X., Wang S., Wang X. (2020). Contribution of Single-Gene Defects to Congenital Cardiac Left-Sided Lesions in the Prenatal Setting. Ultrasound Obstet. Gynecol..

[415] Whitford W., Taylor J., Hayes I., Smith W., Snell R.G., Lehnert K., Jacobsen J.C. (2024). A Novel 11 Base Pair Deletion in KMT2C Resulting in Kleefstra Syndrome 2. Mol. Genet. Genom. Med..

[416] Rots D., Choufani S., Faundes V., Dingemans A.J.M., Joss S., Foulds N., Jones E.A., Stewart S., Vasudevan P., Dabir T. (2024). Pathogenic Variants in KMT2C Result in a Neurodevelopmental Disorder Distinct from Kleefstra and Kabuki Syndromes. Am. J. Hum. Genet..

[417] Cantemir V., Cai D.H., Reedy M.V., Brauer P.R. (2004). Tissue Inhibitor of Metalloproteinase-2 (TIMP-2) Expression during Cardiac Neural Crest Cell Migration and Its Role in proMMP-2 Activation. Dev. Dyn..

[418] Muñoz-Sáez E., Moracho N., Learte A.I.R., Arroyo A.G., Sánchez-Camacho C. (2021). Dynamic Expression of Membrane Type 1-Matrix Metalloproteinase (Mt1-Mmp/Mmp14) in the Mouse Embryo. Cells.

[419] Fealey M.E., Edwards W.D., Miller D.V., Maleszewski J.J. (2012). Unicommissural Aortic Valves: Gross, Histological, and Immunohistochemical Analysis of 52 Cases (1978-2008). Cardiovasc. Pathol..

[420] Tao G., Levay A.K., Gridley T., Lincoln J. (2011). *Mmp15* Is a Direct Target of Snai1 during Endothelial to Mesenchymal Transformation and Endocardial Cushion Development. Dev. Biol..

[421] Abdelrahman H.A., Akawi N., Al-Shamsi A.M., Ali A., Al-Jasmi F., John A., Hertecant J., Al-Gazali L., Ali B.R. (2022). Bi-Allelic Null Variant in Matrix Metalloproteinase-15, Causes Congenital Cardiac Defect, Cholestasis Jaundice, and Failure to Thrive. Clin. Genet..

[422] Nakano S.J., Siomos A.K., Garcia A.M., Nguyen H., SooHoo M., Galambos C., Nunley K., Stauffer B.L., Sucharov C.C., Miyamoto S.D. (2017). Fibrosis-Related Gene Expression in Single Ventricle Heart Disease. J. Pediatr..

[423] Gorący I., Grudniewicz S., Safranow K., Ciechanowicz A., Jakubiszyn P., Gorący A., Brykczyński M. (2020). Genetic Polymorphisms of MMP1, MMP9, COL1A1, and COL1A2 in Polish Patients with Thoracic Aortopathy. Dis. Markers.

[424] Song C., Wei S., Fan Y., Jiang S. (2022). Bioinformatic-Based Identification of Genes Associated with Aortic Valve Stenosis. Heart Surg. Forum.

[425] Bertolino P., Radovanovic I., Casse H., Aguzzi A., Wang Z.-Q., Zhang C.-X. (2003). Genetic Ablation of the Tumor Suppressor Menin Causes Lethality at Mid-Gestation with Defects in Multiple Organs. Mech. Dev..

[426] Ishii M., Han J., Yen H.-Y., Sucov H.M., Chai Y., Maxson R.E. (2005). Combined Deficiencies of Msx1 and Msx2 Cause Impaired Patterning and Survival of the Cranial Neural Crest. Development.

[427] Bernardini L., Castori M., Capalbo A., Mokini V., Mingarelli R., Simi P., Bertuccelli A., Novelli A., Dallapiccola B. (2007). Syndromic Craniosynostosis Due to Complex Chromosome 5 Rearrangement and MSX2 Gene Triplication. Am. J. Med. Genet. Part A.

[428] Li H., Randall W.R., Du S.-J. (2009). skNAC (Skeletal Naca), a Muscle-Specific Isoform of Naca (Nascent Polypeptide-Associated Complex Alpha), Is Required for Myofibril Organization. FASEB J..

[429] Murayama E., Sarris M., Redd M., Le Guyader D., Vivier C., Horsley W., Trede N., Herbomel P. (2015). NACA Deficiency Reveals the Crucial Role of Somite-Derived Stromal Cells in Haematopoietic Niche Formation. Nat. Commun..

[430] Liu L., Wang H.-D., Cui C.-Y., Qin Y.-Y., Fan T.-B., Peng B.-T., Zhang L.-Z., Wang C.-Z. (2017). Whole Exome Sequencing Identifies Novel Mutation in Eight Chinese Children with Isolated Tetralogy of Fallot. Oncotarget.

[431] Opitz R., Hitz M.-P., Vandernoot I., Trubiroha A., Abu-Khudir R., Samuels M., Désilets V., Costagliola S., Andelfinger G., Deladoëy J. (2015). Functional Zebrafish Studies Based on Human Genotyping Point to Netrin-1 as a Link Between Aberrant Cardiovascular Development and Thyroid Dysgenesis. Endocrinology.

[432] Matos-Nieves A., Greskovich S.C., Choudhury T.Z., Manivannan S., Ueyama Y., Rao A.S., Cameron E.M., Garg V. (2025). Expression of Netrin-1 in the Developing Mouse Heart. Gene Expr. Patterns.

[433] Theodoris C.V., Li M., White M.P., Liu L., He D., Pollard K.S., Bruneau B.G., Srivastava D. (2015). Human Disease Modeling Reveals Integrated Transcriptional and Epigenetic Mechanisms of NOTCH1 Haploinsufficiency. Cell.

[434] Wang Q., Zhao N., Kennard S., Lilly B. (2012). Notch2 and Notch3 Function Together to Regulate Vascular Smooth Muscle Development. PLoS ONE.

[435] Stanley K.J., Kalbfleisch K.J., Moran O.M., Chaturvedi R.R., Roifman M., Chen X., Manshaei R., Martin N., McDermott S., McNiven V. (2024). Expanding the Phenotypic Spectrum of NOTCH1 Variants: Clinical Manifestations in Families with Congenital Heart Disease. Eur. J. Hum. Genet..

[436] Meester J.A.N., Verstraeten A., Alaerts M., Schepers D., Van Laer L., Loeys B.L. (2019). Overlapping but Distinct Roles for NOTCH Receptors in Human Cardiovascular Disease. Clin. Genet..

[437] Zhao C., Guo H., Li J., Myint T., Pittman W., Yang L., Zhong W., Schwartz R.J., Schwarz J.J., Singer H.A. (2014). Numb Family Proteins Are Essential for Cardiac Morphogenesis and Progenitor Differentiation. Development.

[438] Liu C., Cao R., Xu Y., Li T., Li F., Chen S., Xu R., Sun K. (2018). Rare Copy Number Variants Analysis Identifies Novel Candidate Genes in Heterotaxy Syndrome Patients with Congenital Heart Defects. Genome Med..

[439] Nomaru H., Liu Y., De Bono C., Righelli D., Cirino A., Wang W., Song H., Racedo S.E., Dantas A.G., Zhang L. (2021). Single Cell Multi-Omic Analysis Identifies a Tbx1-Dependent Multilineage Primed Population in Murine Cardiopharyngeal Mesoderm. Nat. Commun..

[440] Afouda B.A. (2022). Towards Understanding the Gene-Specific Roles of GATA Factors in Heart Development: Does GATA4 Lead the Way?. Int. J. Mol. Sci..

[441] Theis J.L., Niaz T., Sundsbak R.S., Fogarty Z.C., Bamlet W.R., Hagler D.J., Olson T.M. (2022). CELSR1 Risk Alleles in Familial Bicuspid Aortic Valve and Hypoplastic Left Heart Syndrome. Circ. Genom. Precis. Med..

[442] Cantù C., Felker A., Zimmerli D., Prummel K.D., Cabello E.M., Chiavacci E., Méndez-Acevedo K.M., Kirchgeorg L., Burger S., Ripoll J. (2018). Mutations in Bcl9 and Pygo Genes Cause Congenital Heart Defects by Tissue-Specific Perturbation of Wnt/β-Catenin Signaling. Genes Dev..

[443] Itoh N., Ohta H., Nakayama Y., Konishi M. (2016). Roles of FGF Signals in Heart Development, Health, and Disease. Front. Cell Dev. Biol..

[444] Moon A. (2007). The Role of Fgf8 in Cardiovascular Development and Human Congenital Heart Disease. FASEB J..

[445] Zhong H., Zhang R., Li G., Huang P., Zhang Y., Zhu J., Kuang J., Hutchins A.P., Qin D., Zhu P. (2023). C-JUN Is a Barrier in hESC to Cardiomyocyte Transition. Life Sci. Alliance.

[446] Feldman E.R., Li Y., Cutler D.J., Rosser T.C., Wechsler S.B., Sanclemente L., Rachubinski A.L., Elliott N., Vyas P., Roberts I. (2025). Genome-Wide Association Studies of Down Syndrome Associated Congenital Heart Defects Suggests a Genetically Heterogeneous Risk for CHD in DS. Genet. Epidemiol..

[447] Mommersteeg M.T.M., Yeh M.L., Parnavelas J.G., Andrews W.D. (2015). Disrupted Slit-Robo Signalling Results in Membranous Ventricular Septum Defects and Bicuspid Aortic Valves. Cardiovasc. Res..

[448] Mommersteeg M.T.M., Andrews W.D., Ypsilanti A.R., Zelina P., Yeh M.L., Norden J., Kispert A., Chédotal A., Christoffels V.M., Parnavelas J.G. (2013). Slit–Roundabout Signaling Regulates the Development of the Cardiac Systemic Venous Return and Pericardium. Circ. Res..

[449] MacMullin A., Jacobs J.R. (2006). Slit Coordinates Cardiac Morphogenesis in *Drosophila*. Dev. Biol..

[450] Zhao J., Mommersteeg M.T.M. (2018). Slit–Robo Signalling in Heart Development. Cardiovasc. Res..

[451] Kruszka P., Tanpaiboon P., Neas K., Crosby K., Berger S.I., Martinez A.F., Addissie Y.A., Pongprot Y., Sittiwangkul R., Silvilairat S. (2017). Loss of Function in ROBO1 Is Associated with Tetralogy of Fallot and Septal Defects. J. Med. Genet..

[452] Jaouadi H., Gérard H., Théron A., Collod-Béroud G., Collart F., Avierinos J.-F., Zaffran S. (2022). Identification of Non-Synonymous Variations in ROBO1 and GATA5 Genes in a Family with Bicuspid Aortic Valve Disease. J. Hum. Genet..

[453] Jaouadi H., Jopling C., Bajolle F., Théron A., Faucherre A., Gerard H., Al Dybiat S., Ovaert C., Bonnet D., Avierinos J.-F. (2023). Expanding the Phenome and Variome of the ROBO-SLIT Pathway in Congenital Heart Defects: Toward Improving the Genetic Testing Yield of CHD. J. Transl. Med..

[454] Ţuţulan-Cuniţă A.C., Papuc S.M., Arghir A., Rötzer K.M., Deshpande C., Lungeanu A., Budişteanu M. (2012). 3p Interstitial Deletion: Novel Case Report and Review. J. Child. Neurol..

[455] Digilio M.C., Pugnaloni F., De Luca A., Calcagni G., Baban A., Dentici M.L., Versacci P., Dallapiccola B., Tartaglia M., Marino B. (2019). Atrioventricular Canal Defect and Genetic Syndromes: The Unifying Role of Sonic Hedgehog. Clin. Genet..

[456] Iyer K.R., Clarke S.L., Guarischi-Sousa R., Gjoni K., Heath A.S., Young E.P., Stitziel N.O., Laurie C., Broome J.G., Khan A.T. (2025). Unveiling the Genetic Landscape of Coronary Artery Disease Through Common and Rare Structural Variants. J. Am. Heart Assoc..

[457] Jin L., Mo W., Yan Y., Wang Y. (2023). Novel Mutation in the SETD1A Gene in a Newborn Patient Associating with Congenital Airway and Heart Defeats: A Case Report. Medicine.

[458] Chen F., Chen J., Wang H., Tang H., Huang L., Wang S., Wang X., Fang X., Liu J., Li L. (2021). Histone Lysine Methyltransferase SETD2 Regulates Coronary Vascular Development in Embryonic Mouse Hearts. Front. Cell Dev. Biol..

[459] Qiao X., Liu Y., Li P., Chen Z., Li H., Yang X., Finnell R.H., Yang Z., Zhang T., Qiao B. (2016). Genetic Analysis of Rare Coding Mutations of CELSR1–3 in Congenital Heart and Neural Tube Defects in Chinese People. Clin Sci.

[460] Conceição R., Evans R.S., Pearson C.S., Hänzi B., Osborne A., Deshpande S.S., Martin K.R., Barber A.C. (2019). Expression of Developmentally Important Axon Guidance Cues in the Adult Optic Chiasm. Invest. Ophthalmol. Vis. Sci..

[461] Liu J., Zhang L., Wang D., Shen H., Jiang M., Mei P., Hayden P.S., Sedor J.R., Hu H. (2003). Congenital Diaphragmatic Hernia, Kidney Agenesis and Cardiac Defects Associated with Slit3-Deficiency in Mice. Mech. Dev..

[462] Sanna-Cherchi S., Khan K., Westland R., Krithivasan P., Fievet L., Rasouly H.M., Ionita-Laza I., Capone V.P., Fasel D.A., Kiryluk K. (2017). Exome-Wide Association Study Identifies GREB1L Mutations in Congenital Kidney Malformations. Am. J. Hum. Genet..

[463] Qiao X.-H., Wang Q., Wang J., Liu X.-Y., Xu Y.-J., Huang R.-T., Xue S., Li Y.-J., Zhang M., Qu X.-K. (2018). A Novel NR2F2 Loss-of-Function Mutation Predisposes to Congenital Heart Defect. Eur. J. Med. Genet..

[464] Tong W., Xue Q., Li Y., Zhang L. (2011). Maternal Hypoxia Alters Matrix Metalloproteinase Expression Patterns and Causes Cardiac Remodeling in Fetal and Neonatal Rats. Am. J. Physiol. Heart Circ. Physiol..

[465] Corbitt H., Morris S.A., Gravholt C.H., Mortensen K.H., Tippner-Hedges R., Silberbach M., Maslen C.L. (2018). GenTAC Registry Investigators TIMP3 and TIMP1 Are Risk Genes for Bicuspid Aortic Valve and Aortopathy in Turner Syndrome. PLoS Genet..

[466] Corbitt H., Gutierrez J., Silberbach M., Maslen C.L. (2019). The Genetic Basis of Turner Syndrome Aortopathy. Am. J. Med. Genet. Part C Semin. Med. Genet..

[467] Zhao Y., Wang Y., Shi L., McDonald-McGinn D.M., Crowley T.B., McGinn D.E., Tran O.T., Miller D., Lin J.-R., Zackai E. (2023). Chromatin Regulators in the TBX1 Network Confer Risk for Conotruncal Heart Defects in 22q11.2DS. npj Genom. Med..

[468] Ogino J., Dou Y. (2024). Histone Methyltransferase KMT2A: Developmental Regulation to Oncogenic Transformation. J. Biol. Chem..

[469] Sheppard S.E., Campbell I.M., Harr M.H., Gold N., Li D., Bjornsson H.T., Cohen J.S., Fahrner J.A., Fatemi A., Harris J.R. (2021). Expanding the Genotypic and Phenotypic Spectrum in a Diverse Cohort of 104 Individuals with Wiedemann-Steiner Syndrome. Am. J. Med. Genet. Part A.

[470] Abraham S., Lindo C., Peoples J., Cox A., Lytle E., Nguyen V., Mehta M., Alvarez J.D., Yooseph S., Pacher P. (2022). Maternal Binge Alcohol Consumption Leads to Distinctive Acute Perturbations in Embryonic Cardiac Gene Expression Profiles. Alcohol Clin. Exp. Res..

[471] Argào E.A., Kern M.J., Branford W.W., Scott W.J., Potter S.S. (1995). Malformations of the Heart, Kidney, Palate, and Skeleton in α-MHC-*Hoxb-7* Transgenic Mice. Mech. Dev..

[472] Bergwerff M., DeRuiter M.C., Gittenberger-de Groot A.C. (1999). Comparative Anatomy and Ontogeny of the Ductus Arteriosus, a Vascular Outsider. Anat Embryol.

[473] Wu J., Dou B., Meng G., Wang H., Hou Y., Xia J., Bai Y., Kong X. (2020). Phenotypic and genetic characteristics of a child with 7p15 deletion syndrome. Zhonghua Yi Xue Yi Chuan Xue Za Zhi.

[474] Gong L., Qiu G., Jiang H., Xu X., Zhu H., Sun K. (2005). Analysis of Single Nucleotide Polymorphisms and Haplotypes in HOXC Gene Cluster within Susceptible Region 12q13 of Simple Congenital Heart Disease. Zhonghua Yi Xue Yi Chuan Xue Za Zhi.

[475] Schatteman G.C., Loushin C., Li T., Hart C.E. (1996). PDGF-A Is Required for Normal Murine Cardiovascular Development. Dev. Biol..

[476] Steurer M.A., Norton M.E., Baer R.J., Shaw G.M., Keating S., Moon-Grady A.J., Chambers C.D., Jelliffe-Pawlowski L.L. (2017). The Association of Maternal Lymphatic Markers and Critical Congenital Heart Defects in the Fetus-A Population Based Case-Control Study. Am. J. Med. Genet. Part A.

[477] Kulkarni S.S., Khokha M.K. (2018). WDR5 Regulates Left-Right Patterning via Chromatin-Dependent and -Independent Functions. Development.

[478] Zhao T., Wang M., Li Z., Li H., Yuan D., Zhang X., Guo M., Qian W., Cheng D. (2023). Wds-Mediated H3K4me3 Modification Regulates Lipid Synthesis and Transport in Drosophila. Int. J. Mol. Sci..

[479] Crucean A., Alqahtani A., Barron D.J., Brawn W.J., Richardson R.V., O’Sullivan J., Anderson R.H., Henderson D.J., Chaudhry B. (2017). Re-Evaluation of Hypoplastic Left Heart Syndrome from a Developmental and Morphological Perspective. Orphanet J. Rare Dis..

[480] Li P., Li H., Zheng Y., Qiao B., Duan W., Huang L., Liu W., Wang H. (2015). Variants in the Regulatory Region of WNT5A Reduced Risk of Cardiac Conotruncal Malformations in the Chinese Population. Sci. Rep..

[481] Lee A., Wei S., Schwertani A. (2019). A Notch More: Molecular Players in Bicuspid Aortic Valve Disease. J. Mol. Cell Cardiol..

[482] Nelson J.S., Kwok C., Braganca N.E., Lopez D.L., Espina Rey A.P., Robinson M., Ebert S.N. (2022). Comparison of DNA Methylation Patterns across Tissue Types in Infants with Tetralogy of Fallot. Birth Defects Res..

[483] Goddard L.M., Duchemin A.-L., Ramalingan H., Wu B., Chen M., Bamezai S., Yang J., Li L., Morley M., Wang T. (2017). Hemodynamic Forces Sculpt Developing Heart Valves through a KLF2-WNT9B Paracrine Signaling Axis. Dev. Cell.

[484] Spinner N.B., Loomes K.M., Krantz I.D., Gilbert M.A., Adam M.P., Feldman J., Mirzaa G.M., Pagon R.A., Wallace S.E., Amemiya A. (1993). Alagille Syndrome. GeneReviews^®^.

[485] Broberg M., Ampuja M., Jones S., Ojala T., Rahkonen O., Kivelä R., Priest J., Palotie A., Ollila H.M., Helle E. (2024). Genome-Wide Association Studies Highlight Novel Risk Loci for Septal Defects and Left-Sided Congenital Heart Defects. BMC Genom..

[486] Escalante-Alcalde D., Morales S.L., Stewart C.L. (2009). Generation of a Reporter-Null Allele of Ppap2b/Lpp3and Its Expression during Embryogenesis. Int. J. Dev. Biol..

[487] Grazioli A., Alves C.S., Konstantopoulos K., Yang J.T. (2006). Defective Blood Vessel Development and Pericyte/pvSMC Distribution in *A4 Integrin*-Deficient Mouse Embryos. Dev. Biol..

[488] Palmquist-Gomes P., Ruiz-Villalba A., Guadix J.A., Romero J.P., Bessiéres B., MacGrogan D., Conejo L., Ortiz A., Picazo B., Houyel L. (2023). Origin of Congenital Coronary Arterio-Ventricular Fistulae from Anomalous Epicardial and Myocardial Development. Exp. Mol. Med..

[489] Moreira C.G.A., Jacinto A., Prag S. (2013). *Drosophila* Integrin Adhesion Complexes Are Essential for Hemocyte Migration In Vivo. Biol. Open.

[490] Schumacher J.A., Wright Z.A., Owen M.L., Bredemeier N.O., Sumanas S. (2020). Integrin A5 and Integrin A4 Cooperate to Promote Endocardial Differentiation and Heart Morphogenesis. Dev. Biol..

[491] Vickers A., Tewary M., Laddach A., Poletti M., Salameti V., Fraternali F., Danovi D., Watt F.M. (2021). Plating Human iPSC Lines on Micropatterned Substrates Reveals Role for ITGB1 nsSNV in Endoderm Formation. Stem Cell Rep..

[492] Zhou X., Fang X., Ithychanda S.S., Wu T., Gu Y., Chen C., Wang L., Bogomolovas J., Qin J., Chen J. (2023). Interaction of Filamin C With Actin Is Essential for Cardiac Development and Function. Circ. Res..

[493] Rastogi S., Liberles D.A. (2005). Subfunctionalization of Duplicated Genes as a Transition State to Neofunctionalization. BMC Evol. Biol..

[494] Clarence T., Robert N.S.M., Sarigol F., Fu X., Bates P.A., Simakov O. (2023). Robust 3D Modeling Reveals Spatiosyntenic Properties of Animal Genomes. iScience.

[495] Irie N., Sehara-Fujisawa A. (2007). The Vertebrate Phylotypic Stage and an Early Bilaterian-Related Stage in Mouse Embryogenesis Defined by Genomic Information. BMC Biol..

[496] Papaioannou V.E., Behringer R.R. (2012). Early Embryonic Lethality in Genetically Engineered Mice: Diagnosis and Phenotypic Analysis. Vet. Pathol..

[497] Asadzadeh J., Neligan N., Kramer S.G., Labrador J.-P. (2016). Tinman Regulates NetrinB in the Cardioblasts of the Drosophila Dorsal Vessel. PLoS ONE.

[498] Li H., Janssens J., De Waegeneer M., Kolluru S.S., Davie K., Gardeux V., Saelens W., David F.P.A., Brbić M., Spanier K. (2022). Fly Cell Atlas: A Single-Nucleus Transcriptomic Atlas of the Adult Fruit Fly. Science.

[499] Wang W., Bouhours M., Gracheva E.O., Liao E.H., Xu K., Sengar A.S., Xin X., Roder J., Boone C., Richmond J.E. (2008). ITSN-1 Controls Vesicle Recycling at the Neuromuscular Junction and Functions in Parallel with DAB-1. Traffic.

[500] Bandura J.L., Beall E.L., Bell M., Silver H.R., Botchan M.R., Calvi B.R. (2005). Humpty Dumpty Is Required for Developmental DNA Amplification and Cell Proliferation in Drosophila. Curr. Biol..

[501] Grant J., Saldanha J.W., Gould A.P. (2010). A Drosophila Model for Primary Coenzyme Q Deficiency and Dietary Rescue in the Developing Nervous System. Dis. Models Mech..

[502] Rodríguez-Vázquez M., Vaquero D., Parra-Peralbo E., Mejía-Morales J.E., Culi J. (2015). Drosophila Lipophorin Receptors Recruit the Lipoprotein LTP to the Plasma Membrane to Mediate Lipid Uptake. PLoS Genet..

[503] Ding M., Zheng L., Li Q.F., Wang W.L., Peng W.D., Zhou M. (2021). Exercise-Training Regulates Apolipoprotein B in Drosophila to Improve HFD-Mediated Cardiac Function Damage and Low Exercise Capacity. Front. Physiol..

[504] Lai K., Amsterdam A., Farrington S., Bronson R.T., Hopkins N., Lees J.A. (2009). Many Ribosomal Protein Mutations Are Associated with Growth Impairment and Tumor Predisposition in Zebrafish. Dev. Dyn..

[505] Johnson A.N., Mokalled M.H., Haden T.N., Olson E.N. (2011). JAK/Stat Signaling Regulates Heart Precursor Diversification in Drosophila. Development.

[506] Daubresse G., Deuring R., Moore L., Papoulas O., Zakrajsek I., Waldrip W.R., Scott M.P., Kennison J.A., Tamkun J.W. (1999). The Drosophila Kismet Gene Is Related to Chromatin-Remodeling Factors and Is Required for Both Segmentation and Segment Identity. Development.

[507] Koemans T.S., Kleefstra T., Chubak M.C., Stone M.H., Reijnders M.R.F., de Munnik S., Willemsen M.H., Fenckova M., Stumpel C.T.R.M., Bok L.A. (2017). Functional Convergence of Histone Methyltransferases EHMT1 and KMT2C Involved in Intellectual Disability and Autism Spectrum Disorder. PLoS Genet..

[508] Karpe F., Pinnick K.E. (2015). Biology of Upper-Body and Lower-Body Adipose Tissue--Link to Whole-Body Phenotypes. Nat. Rev. Endocrinol..

[509] Joll J.E., Riley L.A., Bersi M.R., Nyman J.S., Merryman W.D. (2022). Sclerostin Ablation Prevents Aortic Valve Stenosis in Mice. Am. J. Physiol.-Heart Circ. Physiol..

[510] Vann K.R., Sharma R., Hsu C.-C., Devoucoux M., Tencer A.H., Zeng L., Lin K., Zhu L., Li Q., Lachance C. (2025). Structure-Function Relationship of ASH1L and Histone H3K36 and H3K4 Methylation. Nat. Commun..

[511] Zhou L., Canagarajah B., Zhao Y., Baibakov B., Tokuhiro K., Maric D., Dean J. (2017). BTBD18 Regulates a Subset of piRNA-Generating Loci through Transcription Elongation in Mice. Dev. Cell.

[512] Fritz K.R., Zhang Y., Ruest L.B. (2019). Cdc42 Activation by Endothelin Regulates Neural Crest Cell Migration in the Cardiac Outflow Tract. Dev. Dyn..

[513] Bakovic P., Mirosevic V., Svagusa T., Sepac A., Kulic A., Milicic D., Gasparovic H., Rudez I., Urlic M., Sikiric S. (2025). Reduced Expression of UPRmt Proteins HSP10, HSP60, HTRA2, OMA1, SPG7, and YME1L Is Associated with Accelerated Heart Failure in Humans. Biomedicines.

[514] Jahncke J.N., Wright K.M. (2023). The Many Roles of Dystroglycan in Nervous System Development and Function: Dystroglycan and Neural Circuit Development: Dystroglycan and Neural Circuit Development. Dev. Dyn..

[515] Bertero A., Madrigal P., Galli A., Hubner N.C., Moreno I., Burks D., Brown S., Pedersen R.A., Gaffney D., Mendjan S. (2015). Activin/Nodal Signaling and NANOG Orchestrate Human Embryonic Stem Cell Fate Decisions by Controlling the H3K4me3 Chromatin Mark. Genes Dev..

[516] Yang Z., Shah K., Khodadadi-Jamayran A., Jiang H. (2016). Dpy30 Is Critical for Maintaining the Identity and Function of Adult Hematopoietic Stem Cells. J. Exp. Med..

[517] Sadeghi M.B., Nakhaee A., Saravani R., Sargazi S. (2021). Significant Association of LXRβ (NR1H2) Polymorphisms (Rs28514894, Rs2303044) with Type 2 Diabetes Mellitus and Laboratory Characteristics. J. Diabetes Metab. Disord..

[518] Zheng Z.-G., Zhu S.-T., Cheng H.-M., Zhang X., Cheng G., Thu P.M., Wang S.P., Li H.-J., Ding M., Qiang L. (2021). Discovery of a Potent SCAP Degrader That Ameliorates HFD-Induced Obesity, Hyperlipidemia and Insulin Resistance via an Autophagy-Independent Lysosomal Pathway. Autophagy.

[519] Guo X., Zhong J., Zhao Y., Fu Y., Sun L.-Y., Yuan A., Liu J., Chen A.F., Pu J. (2024). LXRα Promotes Abdominal Aortic Aneurysm Formation Through UHRF1 Epigenetic Modification of miR-26b-3p. Circulation.

[520] Lammers S., Barrera V., Brennecke P., Miller C., Yoon J., Balolong J., Anderson M.S., Ho Sui S., Steinmetz L.M., von Andrian U.H. (2023). Ehf and Fezf2 Regulate Late Medullary Thymic Epithelial Cell and Thymic Tuft Cell Development. Front. Immunol..

[521] Zhou J., Chehab R., Tkalcevic J., Naylor M.J., Harris J., Wilson T.J., Tsao S., Tellis I., Zavarsek S., Xu D. (2005). Elf5 Is Essential for Early Embryogenesis and Mammary Gland Development during Pregnancy and Lactation. EMBO J..

[522] Alotaibi H. (2023). The Transcription Factor ELF5 Is Essential for Early Preimplantation Development. Mol. Biol. Rep..

[523] Faiella A., D’Esposito M., Rambaldi M., Acampora D., Balsfiore S., Stornaiuolo A., Mallamaci A., Migliaccio E., Gulisano M., Simeone A. (1991). Isolation and Mapping of EVx1, a Human Homeobox Gene Homologus to Even-Skipped, Localized at the 5′ End of Hox1 Locus on Chromosome 7. Nucleic Acids Res..

[524] Szabo L., Morey R., Palpant N.J., Wang P.L., Afari N., Jiang C., Parast M.M., Murry C.E., Laurent L.C., Salzman J. (2015). Statistically Based Splicing Detection Reveals Neural Enrichment and Tissue-Specific Induction of Circular RNA during Human Fetal Development. Genome Biol..

[525] Sato A., Scholl A.M., Kuhn E.B., Stadt H.A., Decker J.R., Pegram K., Hutson M.R., Kirby M.L. (2011). FGF8 Signaling Is Chemotactic for Cardiac Neural Crest Cells. Dev. Biol..

[526] Zhang X., Cai S., Chen L., Yuan R., Nie Y., Ding S., Fang Y., Zhu Q., Chen K., Wei H. (2019). Integrated miRNA-mRNA Transcriptomic Analysis Reveals Epigenetic-Mediated Embryonic Muscle Growth Differences between Wuzhishan and Landrace Pigs1. J. Anim. Sci..

[527] Voges H.K., Foster S.R., Reynolds L., Parker B.L., Devilée L., Quaife-Ryan G.A., Fortuna P.R.J., Mathieson E., Fitzsimmons R., Lor M. (2023). Vascular Cells Improve Functionality of Human Cardiac Organoids. Cell Rep..

[528] Jiang D.-S., Yi X., Li R., Su Y.-S., Wang J., Chen M.-L., Liu L.-G., Hu M., Cheng C., Zheng P. (2017). The Histone Methyltransferase Mixed Lineage Leukemia (MLL) 3 May Play a Potential Role in Clinical Dilated Cardiomyopathy. Mol. Med..

[529] Novotny E., Compton S., Liu P.P., Collins F.S., Chandrasekharappa S.C. (2009). In Vitro Hematopoietic Differentiation of Mouse Embryonic Stem Cells Requires the Tumor Suppressor Menin and Is Mediated by Hoxa9. Mech. Dev..

[530] Zhang H.-L., Luo T.-H., Feng L., Zhao Y., Li W.-Y., Xu J., Zhang Q., Xu L.-H., Zheng S., Li G. (2007). Microarray Analysis of Gene Expression in Men1 Knockout Embryoid Body Reveals Genetic Events Involved in Early Mouse Embryonic Development. Biochem. Biophys. Res. Commun..

[531] Lopes M., Goupille O., Cloment C.S., Lallemand Y., Cumano A., Robert B. (2011). Msx Genes Define a Population of Mural Cell Precursors Required for Head Blood Vessel Maturation. Development.

[532] Berger F., Berkholz J., Breustedt T., Ploen D., Munz B. (2012). Skeletal Muscle-Specific Variant of Nascent Polypeptide Associated Complex Alpha (skNAC): Implications for a Specific Role in Mammalian Myoblast Differentiation. Eur. J. Cell Biol..

[533] Azhdari M., zur Hausen A. (2025). Wnt/β-Catenin and Notch Signaling Pathways in Cardiovascular Disease: Mechanisms and Therapeutics Approaches. Pharmacol. Res..

[534] Paolini A., Fontana F., Pham V.-C., Rödel C.J., Abdelilah-Seyfried S. (2021). Mechanosensitive Notch-Dll4 and Klf2-Wnt9 Signaling Pathways Intersect in Guiding Valvulogenesis in Zebrafish. Cell Rep..

[535] Yilbas A., Hamilton A., Wang Y., Mach H., Lacroix N., Davis D.R., Chen J., Li Q. (2014). Activation of GATA4 Gene Expression at the Early Stage of Cardiac Specification. Front. Chem..

[536] Rivera-Feliciano J., Lee K.-H., Kong S.W., Rajagopal S., Ma Q., Springer Z., Izumo S., Tabin C.J., Pu W.T. (2006). Development of Heart Valves Requires Gata4 Expression in Endothelial-Derived Cells. Development.

[537] Jászai J., Brand M. (2002). Cloning and Expression of *Ventrhoid*, a Novel Vertebrate Homologue of the *Drosophila* EGF Pathway Gene *Rhomboid*. Mech. Dev..

[538] Li D., Ma Q. (2025). Ubiquitin-Specific Protease: An Emerging Key Player in Cardiomyopathy. Cell Commun. Signal..

[539] Fei X., Song C., Cui J., Li Y., Lei X., Tang H. (2025). The Role of Deubiquitinases in Cardiovascular Diseases: Mechanisms and Therapeutic Implications. Front. Cardiovasc. Med..

[540] Fraile J.M., Campos-Iglesias D., Rodríguez F., Astudillo A., Vilarrasa-Blasi R., Verdaguer-Dot N., Prado M.A., Paulo J.A., Gygi S.P., Martín-Subero J.I. (2018). Loss of the Deubiquitinase USP36 Destabilizes the RNA Helicase DHX33 and Causes Preimplantation Lethality in Mice. J. Biol. Chem..

[541] Kranz A., Anastassiadis K. (2020). The Role of SETD1A and SETD1B in Development and Disease. Biochim. Biophys. Acta Gene Regul. Mech..

[542] Wansleeben C., Meijlink F. (2011). The Planar Cell Polarity Pathway in Vertebrate Development. Dev. Dyn..

[543] Humeres C., Venugopal H., Frangogiannis N.G. (2022). Smad-Dependent Pathways in the Infarcted and Failing Heart. Curr. Opin. Pharmacol..

[544] Wang W., Song B., Anbarchian T., Shirazyan A., Sadik J.E., Lyons K.M. (2016). Smad2 and Smad3 Regulate Chondrocyte Proliferation and Differentiation in the Growth Plate. PLoS Genet..

[545] Jiang H., Bai L., Song S., Yin Q., Shi A., Zhou B., Lian H., Chen H., Xu C.-R., Wang Y. (2023). EZH2 Controls Epicardial Cell Migration during Heart Development. Life Sci. Alliance.

[546] França M.M., Mendonca B.B. (2022). Genetics of Ovarian Insufficiency and Defects of Folliculogenesis. Best Pract. Res. Clin. Endocrinol. Metab..

[547] Searcy R.D., Yutzey K.E. (1998). Analysis of Hox gene expression during early avian heart development. Dev. Dyn..

[548] Garcia-Padilla C., Dueñas A., Franco D., Garcia-Lopez V., Aranega A., Garcia-Martinez V., Lopez-Sanchez C. (2022). Dynamic MicroRNA Expression Profiles During Embryonic Development Provide Novel Insights Into Cardiac Sinus Venosus/Inflow Tract Differentiation. Front. Cell Dev. Biol..

[549] Hrycaj S.M., Marty-Santos L., Cebrian C., Rasky A.J., Ptaschinski C., Lukacs N.W., Wellik D.M. (2018). Hox5 Genes Direct Elastin Network Formation during Alveologenesis by Regulating Myofibroblast Adhesion. Proc. Natl. Acad. Sci. USA.

[550] Morioka N., Ganier C., Watt F.M. (2025). Fetal Fibroblast Heterogeneity Defines Dermal Architecture during Human Embryonic Skin Development. J. Investig. Dermatol..

[551] Kang J., Gu Y., Li P., Johnson B.L., Sucov H.M., Thomas P.S. (2008). PDGF-A as an Epicardial Mitogen during Heart Development. Dev. Dyn..

[552] Moore K., Fulmer D., Guo L., Koren N., Glover J., Moore R., Gensemer C., Beck T., Morningstar J., Stairley R. (2021). PDGFRα: Expression and Function during Mitral Valve Morphogenesis. J. Cardiovasc. Dev. Dis..

[553] Bi Y., Lv Z., Wang Y., Hai T., Huo R., Zhou Z., Zhou Q., Sha J. (2011). WDR82, a Key Epigenetics-Related Factor, Plays a Crucial Role in Normal Early Embryonic Development in Mice. Biol. Reprod..

[554] Paolini A., Sharipova D., Lange T., Abdelilah-Seyfried S. (2023). Wnt9 Directs Zebrafish Heart Tube Assembly via a Combination of Canonical and Non-Canonical Pathway Signaling. Development.

[555] Smyth S.S., Kraemer M., Yang L., Van Hoose P., Morris A.J. (2020). Roles for Lysophosphatidic Acid Signaling in Vascular Development and Disease. Biochim. Biophys. Acta Mol. Cell Biol. Lipids.

[556] Lu C., Wu X., Meng X., Liu Y., Yang T., Zeng Y., Chen Y., Huang Y., Fang Z., Yang X. (2024). Silver Nanoparticles Exposure Impairs Cardiac Development by Suppressing the Focal Adhesion Pathway in Zebrafish. Int. J. Nanomed..

[557] Geng Z., Wang J., Pan L., Li M., Zhang J., Cai X., Chu M. (2017). Microarray Analysis of Differential Gene Expression Profile Between Human Fetal and Adult Heart. Pediatr. Cardiol..

[558] Barth J.L., Clark C.D., Fresco V.M., Knoll E.P., Lee B., Argraves W.S., Lee K.-H. (2010). Jarid2 Is among a Set of Genes Differentially Regulated by Nkx2.5 during Outflow Tract Morphogenesis. Dev. Dyn..

[559] Brauer P.R., Cai D.H. (2002). Expression of Tissue Inhibitor of Metalloproteinases (TIMPs) during Early Cardiac Development. Mech. Dev..

[560] Chandran L., Backer W., Schleutker R., Kong D., Beati S.A.H., Luschnig S., Müller H.-A.J. (2023). Src42A Is Required for E-Cadherin Dynamics at Cell Junctions during Drosophila Axis Elongation. Development.

[561] Niikura Y., Tabata Y., Tajima A., Inoue I., Arai K., Watanabe S. (2006). Zebrafish Numb Homologue: Phylogenetic Evolution and Involvement in Regulation of Left–Right Asymmetry. Mech. Dev..

[562] Oh Y., Abid R., Dababneh S., Bakr M., Aslani T., Cook D.P., Vanderhyden B.C., Park J.G., Munshi N.V., Hui C.-C. (2024). Transcriptional Regulation of the Postnatal Cardiac Conduction System Heterogeneity. Nat. Commun..

[563] Zhang Y., Huang L., Wang C., Gao D., Zuo Z. (2013). Phenanthrene Exposure Produces Cardiac Defects during Embryo Development of Zebrafish (*Danio rerio*) through Activation of MMP-9. Chemosphere.

