# Supplementary material for: In Vivo Models of Cardiovascular Disease: Drosophila melanogaster as a Genetic Model of Congenital Heart Disease
Source: Biomedicines. 2025 Oct 21;13(10):2569. doi: 10.3390/biomedicines13102569 (PMC12561791; doi:10.3390/biomedicines13102569)
Supplement: Supplementary file 1 [file biomedicines-13-02569-s001.zip › biomedicines-3903996-supplementary_File S1.pdf]

Review

In Vivo Models of Cardiovascular Disease: *Drosophila melanogaster* as a Genetic Model of Congenital Heart Disease

Supplementary Information

Theodora M Stougiannou [1,*], Maria Koutini [1], Fotios Mitropoulos [2] and Dimos Karangelis [1]

1 Department of Cardiothoracic Surgery, University General Hospital, Democritus University of Thrace, 68100 Alexandroupolis, Greece
2 Department of Paediatric, Congenital and Adult Cardiac Surgery, Mitera Hospital, Marousi, 15123 Athens, Greece
* Correspondence: theodorastougiannos@gmail.com

Supplementary Information File S1-Section A

Table S1. Cardioblast (CB) and pericardial cell (PC) populations identified in D. melanogaster during cardiac development along with common gene markers for each cell type.

Notes: BRC-Z1, Broad Complex Z1 Zinc finger protein; CB, Cardioblast; CTPC, Cut+ /tinman+ (tin+) pericardial cell; Doc1, Dorsocross 1; Doc3, Dorsocross 3; ELPC, End of the line pericardial cell; EPC, Even-skipped+ (Eve+)/tinman+ (tin+) pericardial cell; Mef2, Myocyte enhancing factor 2; NetB, Netrin B; OPC, Odd-skipped+ (Odd+) pericardial cell; Rab5, Ras-related protein in brain 5; Robo, Roundabout; Tg, Transglutaminase; WHPC, Wing heart pericardial cell; Wg, Wingless; doc, dorsocross; edl, ETS domain lacking; eve, even-skipped; fas, fas cell surface death receptor; gCB, generic Cardioblast; lb, ladybird; mid, midline; ndae1, driven anion exchanger 1; oCB, ostial Cardioblast; odd, odd-skipped; pnt, pointed; pvr, Platelet-derived growth factor (PDGF)/Vascular endothelial growth factor (VEGF) receptor; pyr, pyramus; svp, sevenup; tin, tinman; tup, tailup; unc5, behaviourally uncoordinated 5.

Cell type	Gene	Reference
Cardiac progenitor (Stages 11 to 13)	*tin, svp*	[1] [2]
gCB (*tin+lb–*)	*tin, mid, H15, pnt, fas, NetB, unc5, ndae1, Slit, Robo*	[3] [4] [5] [6] [7] [8]
gCB (*tin+ lb+*)	*tin, lb, Slit, Robo*	[9] [8] [10]
oCB	*svp, doc, edl, lb, tup, Wg, pyr, Tg, Slit, Robo, tin* (limited), *fas* (limited)	[11] [1] [1] [6] [8]
Contractile cardiac cell	*tin, Mef2, BRC-Z1, rab5*	[12] [13] [14]
Ostial cardiac cell	*svp, Mef2, BRC-Z1, Wg, Doc1, Doc3, rab5*	[10] [12] [13] [15] [14]
OPC	*odd, tup*	[16] [6] [17]
EPC	*eve, tin*	[16] [3] [6]
WHPC	*eve*	[1] [3]
ELPC	*cut*	[1]
CTPC	*cut, tin*	[1]
Valve cell	*tin, rab5*	[18] [14]

Table S2. Genes involved in the Cardiac Gene Regulatory Networks: Mutations and Phenotypes.

Notes: ADv, Anterior Dorsal vessel; AM, Alary muscle; Abd-A, Abdominal A; BTBD18, BTB Domain Containing 18; CB, Cardioblast; COUP-TF, Chicken ovalbumin upstream promoter transcription factor (TF); D-mef2, Drosophila Myocyte enhancer factor 2; DIOPT, Drosophila RNAi Screening Center Integrative Ortholog Prediction Tool; DNA, Deoxyribonucleic acid; DPE, Downstream promoter element; DPR, Downstream promoter region; Dv, Dorsal vessel; Eve, Even-skipped; GBR, Germ band retraction; GRN, Gene regulatory network; Gata1/2/3/6, GATA binding protein 1/2/3/6; Gata4, GATA binding protein 4; HCHE, Heart- and Neural crest derivatives-expressed protein (Hand) cardiac and hematopoietic enhancer; Hand, Heart- and Neural crest derivatives-expressed protein; Hox, Homeobox; Hz, Hertz; Islet-1, Insulin-related protein 1; KD, Knockdown; LOF, Loss-of-function; Lbx, Ladybird homeobox; Mef2, Myocyte enhancer factor 2; Mef2a, Myocyte enhancer factor 2a; Mef2c, Myocyte enhancer factor 2c; Msh-2, MutS homolog 2; Msx2, MutS homolog 2 (Msh) homeobox 2; Nkx2.5, Nk2 homeobox 5; Nr2f2, Nuclear Receptor subfamily 2 group F member 2; Odd, Odd-skipped; PC, Pericardial cell; PDv, Posterior Dorsal vessel; Prc, Pericardin; RNAi, Ribonucleic acid interference; SCE, sevenup (svp) cardiac enhancer; SV, Stroke volume; TAMR, Thoracic alary muscle related; TF, Transcription factor; Tbx20, T-box transcription factor TBX20; Tbx5, T-box transcription factor TBX5; Tbx6, T-box transcription factor TBX6; Ubx, Ultrabithorax; Wg, Wingless; bHLH, basic Helix-Loop-Helix; bab2, bric-a-brac 2; bpm, beats per minute; doc, dorsocross; h, hour(s); lb, ladybird; mid, midline; nmr1, neuromancer 1; nmr2, neuromancer 2; pnr, pannier; sec, second(s); srp, serpent; svp, sevenup; tin, tinman; tup, tailup; zfh1, Zinc finger homeobox 1; αPS2, α subunit integrin chain 2; μm, micrometers.

**Gene**	***Ortholog***	**Gene information**	**Mutation**	**Mutation phenotype**	**Study (**Reference**)**
msh-2	***MSX2***	Msh-homeobox TF gene, implicated in early mesoderm development	**Deficiency**	No effect on mesodermal invagination at ventral furrow, No effect on mesodermal migration, Absence of visceral muscle, Absence of Dv, Some somatic muscle present (abnormal) **(Embryo)**	Bodmer et al., 1990 [19], Hu et al., 2011 **[20]**
tin	***NKX2.5***	Nk2-homeobox TF gene, implicated in in mesoderm, cardiac mesoderm specification; acts cooperatively with *doc*, *pnr* in early stages, antagonizes *doc* expression in later stages (CB diversification)	**Null mutation, Deficiency**	Absence of cardiac and dorsal somatic muscle, No effect on hindgut visceral muscle, Disruption in segmental somatic muscle arrangement with fewer muscles in each segment **(Embryo)**	Bodmer, 1993 [21], Hu et al., 2011 **[20]**
tin			**Null mutation, Deficiency**	No effect on *doc* expression [**Stage 11**], Reduction/Absence of *doc* expression [**Stage 12 onwards**] (*tin*)	Reim and Frasch, 2005 [22]
R321N **variant *(tin)***	***K158N*** variant ***(NKX2.5)***	**Unknown clinical significance**, mutation in the Nkx2.5 α-helix domain, disruption in Nkx2.5/Tbx5 interaction	**Screening**	Reduced binding affinity for DNA, by ~ 80%, Reduction in tin-dependent enhancer activation (by ~ 100), Reduction in *doc* binding affinity (*in vitro*)	Lovato et al., 2023 [23]
				No effect on CB/PC patterning, No effect on cardiac specification (**Embryo**); No effect on myofibril arrangement, No effect on Dv diameter **(Larva)**	

Gene	*Ortholog*	Gene information	Mutation	Mutation phenotype	Study (Reference)
				No effect on gross Dv patterning/diameter, Disruption in β-integrin arrangement with increase in curve/straight ratio (by ~ 0.1) (More severe with fragmentation when combined with ***tin**^{EC40}* null allele) **(Adult)**	
**DPE *tin***	[DPR effect on ***NKX2.5*** expression not evaluated]	Core promoter element, similar motif found in promoters of developmentally regulated genes (including Hox genes)	**Partial LOF** [Reduced *tin* expression]	Reduction in *tin* expression (by ~ 3 at **3-4 h/4-5 h**, ~ 1.5 at **5-6 h),** Reduction in tinman protein levels (by ~ 0.25 **at 2-8 h)**, Reduction in *svp* expression (by ~ 0.5) and *doc* expression (by ~ 0.7 at **4-6 h**), Increase in *Mef2* expression (by ~ 0.5) and *Eve* (by ~ 2 at **4-6 h**), Misexpression of *Odd*, Reduction in *tin*+, *svp*+, *Mef2*+ CB numbers with fewer CB specified (**ADv**)	Sloutskin et al., 2024 [24]
				No effect on gross Dv morphology, No effect on somatic/visceral muscle	
				Reduction in diastolic diameter (by ~ 20 μm), Reduction in fractional shortening (by ~ 10%), Reduction in SV (by ~ 1250 $μm^3$), Increase in systolic interval (by ~ 0.06 sec)	
D-mef2	***MEF2C, MEF2A***	MADS-domain TF gene, implicated in upregulation of cardiac structural genes	**Null mutation, Deficiency**	Absence of cardiac, somatic, visceral muscle differentiation, Absence of contractile protein expression, No effect on myoblast specification/localization **(Embryo)**	Lilly et al., 1995 [25], Hu et al., 2011 [20]
svp	***NR2F2***	COUP-TF gene, implicated in CB/PC diversification	**Null mutation**	Loss of *tin* repression in *svp*+ CB with acquisition of *tin*+ CB fate, Continuous *tin* expression along the Dv **(Embryo)**	Lo and Frasch, 2001 [2], Hu et al., 2011 [20]
			**Overexpression**	Absence of *tin* expression in CB, PC **(Embryo)**	
**SCE *svp***		*svp* expression regulated by *tin* via binding at SCE	**Partial LOF** **[**Reduced SCE *svp* function**]**	Absence of cardiac enhancer activity [**Stage 11**] with some recovery in expression later in development [**Stage 16**]	Ryan et al., 2007 [26]
mid (nmr2), H15 (nmr1)	***TBX20***	T-box TF gene, implicated in cardiac development, CB/PC diversification	**Null mutation, Deficiency**	Disruption in the late phase of *tin* expression, Disruption in CB/PC diversification, No effect on *tin, Eve, Odd, zfh1* PC expression **(Embryo)**	Reim et al., 2005 [9], Hu et al., 2011 [20]

**Gene**	***Ortholog***	**Gene information**	**Mutation**	**Mutation phenotype**	**Study (**Reference**)**
			**Ectopic expression**	Expansion in *tin* CB expression, Increase in CB apoptosis, Reduction in *Eve* expression (somatic muscle), Reduction in *Eve, Odd* PC expression **(Embryo)**	
mid (nmr2), H15 (nmr1)		***mid (nmr2)*** expression detectable in developing embryo from **Stage 11** onwards, ***H15 (nmr1)*** detectable after **Stage 13**	**Null mutation, KD** [RNAi]	Mild cardiac defects, No effect on early cardiac markers, Disruption in CB alignment (intercalation, gaps, clustering, misorientation), Reduction in *tin* expression [**Stage 14 onwards**] (*nmr1*), Absence of *lb* expression, Expansion in *Eve, Odd* expression **(Embryo)**	Qian et al., 2005a [27]
			**Overexpression** [mesoderm]	Reduction in *Eve* expression (directly/indirectly via *lb*), Expansion in *lb* expression, Reduction in *Eve, Odd*+ PC expression **(Embryo)**	
mid (nmr2), H15 (nmr1)		Implicated in physiological cardiac function	**Null mutation, KD** [RNAi]	Increase in heart failure rate after electrical pacing-induced stress (by ~ 0.4 for ***H15 [nmr1],*** by ~ 0.7 for ***mid [nmr2]***), Disruption in myofibrillar arrangement [***mid (nmr2)] [H15 (nmr1)]***, Increase in heart failure rate after electrical pacing-induced stress (by ~ 0.35 for combined ***H15 (nmr1)/mid (nmr2)/tin***) (**Adult**)	Qian et al., 2008 [28]
pnr	***GATA4***	GATA TF gene, implicated in cardiac development		No effect on *doc* expression [**Stage 11**], Absence of *doc* expression [**Stage 12 onwards**], Disruption in CB specification	
doc	***TBX6***	T-box TF gene, implicated in cardiac development		Increased lethality at embryonic stages (embryo viability decreased to 63%) (combined *tin/doc/pnr*) **(Embryo)**	
srp	***GATA1, GATA2, GATA3, GATA6***	GATA TF gene, implicated in midgut development, hematopoietic lymph gland development	**Ectopic expression** [mesoderm]	Increased HCHE expression (somatic muscle), Mild effects on CB, PC proliferation, No effect on hematopoietic lymph gland progenitors (***tin***); Increased CB proliferation with ectopic CB, Increased HCHE expression (CB), Increased PC proliferation, No effect on hematopoietic lymph gland progenitors (***pnr***); Reduction in CB, PC numbers, Disruption in PC alignment, Increased hematopoietic lymph gland progenitor proliferation (***srp***) **(Embryo)**	Han and Olson, 2005 [29]
tin, pnr, srp		*tin, pnr, srp* regulate *Hand* expression via binding at HCHE			

Gene	*Ortholog*	Gene information	Mutation	Mutation phenotype	Study (Reference)
Hand	***HAND2***	bHLH TF, identified in CB, PC, hematopoietic lymph gland progenitors; Expression regulated by *tin, pnr, srp* via binding at HCHE	**Null mutation**	Lethality at late embryonic/early larval stages (60% of embryos hatch), Reduction/Absence of hematopoietic lymph glands **(Embryo)**; Hypoplastic Dv (gaps in PDv), Reduction in Dv wall thickness, Morphological defects in ~ 20% of individuals with severe defects in ~3% (**Larva**)	Han et al., 2006 [30], Lo et al., 2007 [31]
			**Overexpression [**mesodermal**]**	Increased lethality at late larval stages, Disruption in muscle development	
		Remodeling of Dv during metamorphosis	**Null mutation**	No effect on gross Dv morphology (**Embryo**); Semi-lethality between the 1st/2nd larva instar phase (**Larva**)	Lo et al., 2007 [31]
				Reduced survival (max lifespan reduced to ~ 10 days for 84% of all individuals), Disorganization in myofibrillar arrangement, Reduction in systolic diameter (by ~ 10 μm), Reduction in diastolic diameter (by ~ 40 μm), Reduction in heart rate (by ~ 10 bpm) (**Adult**)	
Eve	***EVX2***	Implicated in segmentation, Repression of *Ubx, Wg* transcription, CB/PC diversification	**Null mutation, Deficiency**	No effect on midline CB alignment, Reduction in PC number/segment (by ~ 2) (**Embryo**)	Fujioka et al., 2005 [32]
				Reduction in heart rate (by ~ 1.5 Hz in **pupae**, by ~ 0.9 (**Adult**), Increase in heart failure rate after electrical pacing-induced stress (up to ~ 0.5, ~ 0.8, for 2 separate lines) (**Adult**)	
tup	***ISL1, ISL2***	LIM homeodomain TF, implicated in cardiac development and CB/PC diversification; activates *Hand, srp, Odd*	**Null mutation, Deficiency**	Hypoplastic Dv, Disruption in CB alignment with gaps and severe distortions, Reduction in CB number (both *tin+, svp/doc+* groups), Absence of most lymph glands with rudimentary structures only, Absence of *Odd+* PC	Tao et al., 2007 [33]
		Implicated in early cardiac development, regulation of *pnr* expression (ectoderm) [Stage 10], *tin, doc, pnr* expression (cardiac mesoderm) [Stage 11-16]	**Null mutation, Deficiency**	Reduction in *tin, doc, pnr* expression, 'GBR' phenotype in 19% of individuals, Severe effects on Dv morphology with loss of *D-mef2+* CB, Disruption in Prc arrangement, Loss of *Odd+* PC in 63% of all individuals with reduction in lymph gland size, Loss of *Eve+* PC	Mann et al., 2009 [17]

**Gene**	***Ortholog***	**Gene information**	**Mutation**	**Mutation phenotype**	**Study (**Reference**)**
		Implicated in AM, TAMR development, expressed in cardiac valve cells	**KD [**RNAi**]**	No effect on mean valve area (~ 900 μm^2 for all groups), no effect on mean valvosome number (~ 3 for all groups), no effect on valve cell positioning, Disruption in myofibrillar arrangement in valve/cardiac cells (longitudinal arrangement) (**similar myofibrillar disruption with LOF *Abd-A, tin* mutations**)	Meyer et al., 2023a [34], Meyer et al., 2023b [18]
bab2	***BTBD18***	*Lb* implicated in *bab2* (CB/PC diversification) and *inflated* (αPS2 integrin) upregulation, terminal muscle cell differentiation	**KD [**RNAi**]**	Expansion in *lb* expression (*Eve*+ cells), Disruption in *Eve*+ PC localization with *Eve*+ groups now located dorsally	Junion et al., 2007 [35]
			**Overexpression [**mesoderm**]**	Severe reduction in *lb*+ cell number, Reduction in *tin*+ CB number	

Table S3. Genes involved in cellular metabolism and protein synthesis/trafficking: Mutations and Phenotypes.

Notes: AM, Alary muscle; APOB, Apolipoprotein B; ASD, Atrial septal defect; ATP, Adenosine triphosphate; Ankrd11, Ankyrin repeat domain containing 11; CB, Cardioblast; CNS, Central nervous system; CO, Cardiac output; Cdk12, Cyclin-dependent kinase 12; D. melanogaster, Drosophila melanogaster; DNA, Deoxyribonucleic acid; Dap160, Dynamin-associated protein 160 kilo Dalton (kD); Donson, Downstream neighbour of Son; Dv, Dorsal vessel; EDD, End-diastolic Diameter; ESD, End-systolic diameter; ETC , Electron transport chain; Ec, Ecdysone; GGPPS/qm, Geranylgeranyl pyrophosphate synthase; Gart, Phosphoribosylglycinamide Formyltransferase, Phosphoribosylglycinamide synthetase, Phosphoribosylaminoimidazole synthetase; Gγ1, G protein gamma (γ) subunit 1; HDAC, Histone deacetylase; HLHS, Hypoplastic left heart syndrome; HMGCR, Hydroxymethyl-glutaryl (HMG) CoA reductase; Hd, Humpty dumpy; Itsn1, Intersectin 1; KD, Knockdown; Kif1A, Kinesin family member 1A; LDL, Low-density lipoprotein; LRP2, Low-density lipoprotein-related receptor 2; MICOS, Mitochondrial contact site and cristae organization system; MYOM2, Myomesin 2; Mhc, Myosin heavy chain; Nos3, Nitrix oxide synthase 3; Org-1, Optomotor blind related gene 1; PC, Pericardial cell; Pdss2, Decaprenyl diphosphate synthase, subunit 2; Prc, Pericardin; RNA, Ribonucleic acid; RNAi, Ribonucleic acid interference; RNF149, Ring finger protein 149; RpL13, Ribosomal protein L13; RpL14, Ribosomal protein L14; RpS24, Ribosomal protein S24; Rpn8, Regulatory particle, Proteasome subunit Rpn8; SPTBN1, Spectrin β chain, brain 1; Shh, Sonic hedgehog; Son, SON Deoxyribonucleic acid (DNA) and Ribonucleic acid (RNA); Spg7, Spastic paraplegia 7; T1, Thoracic segment 1; T2, Thoracic segment 2; TARM, Thoracic-Alary muscle related; TF, Transcription factor; TGFβ, Transforming growth factor beta; Tbx1, T-box transcription factor TBX1; Tbx2, T-box transcription factor TBX2; Ubx, Ultrabithorax; VLM, Ventral longitudinal muscle; VSD, Ventricular septal defect; WT, wild type; Wnt, Wingless-related integration site; apoLpp, a homolog of apolipoprotein B; coQ10, Coenzyme Q10; dChchd3/6, drosophila Coiled-coil-helix-coiled-coil-helix-domain containing protein 6; dMnM, Drosophila Myomesin and Myosin protein; gol, goliath; lb, ladybird; matDiabetic, progeny derived from mothers with Diabetes mellitus (DM); matNormal, progeny derived from mothers without Diabetes mellitus (DM); mgl, megalin; omb, optomotor blind; pmol, picomoles; slou, slouch; tup, tailup; βGGT-I, Geranylgeranyl transferase type I beta (β) subunit; μg, micrograms; μm, micrometer.

**Gene [**Ortholog**]**	**Gene information**	**Mutation**	**Mutation phenotype**	Study (Reference)
HMGCR, Gγ1, GGPPS/qm, βGGT-I	Mevalonate metabolism pathway (*HMGCR, GGPPS/qm, βGGT-I*), implicated in Gγ1 geranylgeranylation and regulation of CB/PC adhesion	**Null mutation, Deficiency**	'**Broken-hearted'** phenotype: CB/PC dissociation with disruption in cell-cell adhesion protein distribution, Loss of physiological heart function, Increased lethality at embryonic stages (100% penetrance for *HMGCR, GGPPS/qm*, 30% penetrance for *βGGT-I*) (**Embryo**)	Yi et al., 2006 [36]
Org-1	T-Box TF gene, implicated in somatic [Stage 9], trunk visceral [Stage 11] mesoderm development	**Null mutation**	Absence of *lb, slou* expression in *Org-1*+ somatic muscle, Severe defects/absence of AM (**Embryo**)	Schaub et al., 2012 [37]
	Implicated in AM, TARM (T1, T2) development	**Null mutation**	Severe defects/Absence of AM, TARM (**Embryo**)	Boukhatmi et al., 2014 [38]
	Implicated in VLM development along with *tup, Ubx,* Ec (metamorphosis)	**KD [RNAi]**	Severe defects in VLM development with absence of VLM, Presence of syncytial AM (**Metamorphosis**)	Schaub et al., 2015 [39]
Notch1	Hyperglycemia reduces *Nos3* locus accessibility; increase in Jarid expression, inhibition of Notch expression	**KD [RNAi]**	Reduction in Notch signaling, Increased Prc content (~ 150% for matNormal, ~ 200% for matDiabetic), Reduction in cardiac cell actin content (~ 80% for both matNormal, matDiabetic) (adult); Increased lethality at embryonic stages for matNormal (15%), matDiabetic (28%) individuals (**Adult**)	Basu et al., 2017 [40]

**Gene [**Orthog**]**	**Gene information**	**Mutation**	**Mutation phenotype**	Study (Reference)
Jarid2	Transcriptional repressor of Notch, regulates histone methyltransferase complexes, implicated in inhibition of *Notch1* expression; Expression increases with reduction in NO levels	**KD [RNAi]**	Increase in Prc content (~ 250% for matNormal, No effect for matDiabetic) (adult); Increased lethality at embryonic stages for matNormal, matDiabetic individuals (~ 5%) (**Adult**)	Basu et al., 2017 [40]
		**Overexpression**	Reduction in Notch signaling, Increase in Prc content (~ 250% for matNormal, ~ 400% for matDiabetic), Reduction in actin content (~ 80% for both matNormal, matDiabetic) (adult); Increased lethality at embryonic stages for matNormal (~ 14%), matDiabetic (~ 26%) (**Adult**)	
Numb	Transcriptional repressor of Notch	**Overexpression**	Reduction in Notch signaling, No effect on Prc content, Disruption in myofibril arrangement, Reduction in actin content (~ 80% for both matNormal, matDiabetic) (adult); Increased lethality at embryonic stages for matNormal (~ 5%), matDiabetic (~ 12%) (**Adult**)	Basu et al., 2017 [40]
bifid (omb)	T-Box TF (Brachyury-like) gene, implicated in CNS, wing, abdominal segment development	**Screening [***TBX2-R20Q, TBX2-R305***]**	Increased lethality at embryonic stages, Absence of phenotype rescue with *D. melanogaster bifid,* human WT *Tbx2,* human variants *TBX2-R20Q, TBX2-R305H* (**Adult**)	Liu et al., 2018 [41]
TBX2-R20Q, TBX2-R305H	*Tbx2* variants implicated in syndromic CHD with additional craniofacial, skeletal, endocrine defects and immune deficiency in humans	**Ectopic expression [***TBX2-R20Q, TBX2-R305H***]**	Increased lethality after eclosion (most severe with *Tbx2*, 15.2% individuals survive), Reduction in eye size (most severe with *bifid* in 63%, *Tbx2* in 71.4% of individuals), Defects in phototransduction (most severe with *bifid, Tbx2),* Reduction in ON/OFF transients (by ~ 0.8 mV), amplitude (by ~ 0.6 mV), Prolongation of depolarization (by ~ 5 mV) (**Adult**)	Liu et al., 2018 [41]
RpL13	Ribosomal protein (Large ribosomal subunit), implicated in proteostasis	**KD [RNAi], Screening**	**'No heart'** phenotype: Absence of heart (larvae, adult) with constricted posterior Dv remnants (**Heart field-specific KD during Embryonic, Larva stage**), No effect (KD during **Larva**, **Adult** stage)	Schroeder et al., 2019 [42]
CG10984	Chromatin regulator, implicated in modulation of histone acetylation and recruitment of HDAC	**KD [RNAi], Screening**	Minimal effect on actin filament arrangement, Disruption in myofibrillar arrangement, Reduction in fractional shortening (by ~ 7%), No effect on diastolic diameter, Increase in systolic diameter (by ~ 5 μm) (**Adult**)	Schroeder et al., 2019 [42]

**Gene [**Orthology**]**	**Gene information**	**Mutation**	**Mutation phenotype**	Study (Reference)
CG2658	Mitochondrial protease, implicated in physiological mitochondrial function	**KD [RNAi], Screening**	Disruption in actin filament arrangement, Disruption in myofibrillar arrangement, Reduction in fractional shortening (by ~ 10%), Increase in diastolic diameter (by ~ 20 μm), Increase in systolic diameter (by ~ 10 μm) (**Adult**)	Pareek et al., 2018 [43], Schroeder et al., 2019 [42]
Dap160	Adaptor protein, implicated in neuronal synapsis morphology, synaptic vesicle recycling	**KD [RNAi], Screening**	Minimal effect on actin filament arrangement, Reduction in fractional shortening (by < 5%), Reduction in diastolic diameter (by ~ 10 μm), No effect on systolic diameter (**Adult**)	Wang et al., 2008 [44], Schroeder et al., 2019 [42]
Gart	Trifunctional enzyme, implicated in lipid metabolism, feeding and lifespan regulation	**KD [RNAi], Screening**	Minimal effect on actin filament arrangement, Disruption in myofibrillar arrangement; Reduction in fractional shortening (by < 5%), No effect on diastolic diameter, No effect on systolic diameter (**Adult**)	He et al., 2023 [45], Schroeder et al., 2019 [42]
Son	RNA-binding protein, prevents nascent poplypeptides from degradation, implicated in cell cycle/apoptosis regulation, TGFβ and Wnt signaling, Integrin-mediated adhesion	**KD [RNAi], Screening**	Disruption in actin filament arrangement, Disruption in myofibrillar arrangement, Reduction in fractional shortening (by < 5%), No effect on diastolic diameter, Increased in systolic diameter (by < 5 μm) (**Adult**)	Tao et al., 2024 [46], Schroeder et al., 2019 [42]
Hd	Implicated in DNA amplification in the *D. melanogaster* ovary, DNA proliferation	**KD [RNAi], Screening**	Disruption in actin filament arrangement, Disruption in myofibrillar arrangement; Reduction in fractional shortening (by ~ 28%), Reduction in diastolic diameter (by ~ 13 μm), No effect on systolic diameter (**Adult**)	Bandura et al., 2005 [47], Schroeder et al., 2019 [42]
CG10585	Implicated in coQ10 biosynthesis, Mitochondrial respiration	**KD [RNAi], Screening**	Reduction in fractional shortening (by ~ 88%), Increase in diastolic diameter (by ~ 5 μm), Increase in systolic diameter (by ~ 78 μm) (**Adult**)	Grant et al., 2010 [48], Schroeder et al., 2019 [42]
Mgl	Large LDL receptor-related protein, mediates receptor endocytosis, implicated in DV wing patterning (*D. melanogaster*) and cell proliferation, *LRP2* variants enriched in HLHS	**KD [RNAi], Screening**	Cardiac dilation, Increase in EDD (by ~ 15 μm), arrhythmia (**Adult**)	Theis et al., 2020 [49], Riedel et al., 2011 [50]

**Gene [**Orthology**]**	**Gene information**	**Mutation**	**Mutation phenotype**	Study (Reference)
apoLpp	Component of Lipophorin (LP ligand), implicated in lipid transport within hemolymph (*D. melanogaster*), Wnt and Shh signaling regulation (cardiac cell proliferation in higher vertebrates) (Apolipophorin)	**KD [RNAi], Screening**	Cardiac arrhythmia (**Adult**)	Theis et al., 2020 [49], Rodríguez-Vázquez et al., 2015 [51], Ding et al., 2021 [52]
dMnM	Myosin-binding protein, interacts with *Mhc,* implicated in cardiac function	**Deficiency, KD [RNAi]**	Cardiac-specific KD: Increase in EDD (most severe with Chromosomal deficiency, by ~ 20 μm), Heart dilation (Mild KD), Heart constriction (Effects dosage-specific) (Strong KD) (**Adult**)	Auxerre-Plantié et al., 2020 [53]
			Muscle-specific KD: Reduction in survival (total lifespan reduced to ~ 35 days in some lines compared to > 35 days for control), Defects in locomotion (**Adult**)	
Kif1A	Microtubule-associated protein, implicated in intracellular transport, cardiac development, *Kif1A* variants identified in Left-sided heart defects, HLHS	**Deficiency, KD [RNAi]**	No effect on Dv structure/function (**Adult**)	Akasaka et al., 2020 [54]
		**Overexpression**	No effect on cardiac development (**Embryo**)	
		**Overexpression**	Increase in ESD (by ~ 20 μm), Reduction in EDD (by ~ 10 μm), Reduction in fractional shortening (by ~ 0.1) (systolic dysfunction); Disruption in myofibrillar arrangement (thin myofibrils), Reduction in adult valve number (by ~ 1.5) with reduced myofibrillar content, Increased in Collagen Type IV content (by ~ $0.5*10^5$) (**Adult**)	
RpS24	Ribosomal protein (Small ribosomal subunit), implicated in proteostasis, *RpS24* mutations associated with DBA in humans	**KD [RNAi], Screening**	**'Minute'** Syndrome: impairment in development, fertility and cardiac function, No effect on embryo hatch rates, Complete Dv atrophy, Increased in Prc density with visible breaks in structure, No effect on heart rate (**Larva**)	Nim et al., 2021 [55]
RpL14	Ribosomal protein (Large ribosomal subunit), implicated in proteostasis, 'Minute'-like phenotype in vertebrates	**KD [RNAi], Screening**	'**Minute'** Syndrome: impairment in development, fertility and cardiac function, Reduction in PC size, Partial Dv atrophy, Reduction in Prc density, No effect on heart rate (**Larva**)	Lai et al., 2009 [56], Nim et al., 2021 [55]

**Gene [**Ortholog**]**	**Gene information**	**Mutation**	**Mutation phenotype**	Study (Reference)
Rpn8	Regulatory component of the 26S Ubiquitin-proteasome complex, implicated in proteostasis	**KD [RNAi], Screening**	No effect on embryo hatch rates, Variability in cell size, Partial Dv atrophy, No effect on heart rate (**Larva**)	Nim et al., 2021 [55]
dChchd3/6	Mitochondrial MICOS complex subunit, implicated in the maintenance of cristae morphology, ETC component assembly, mitochondrial fission/fusion, heart function (larva/adult) (*D. melanogaster*), *Chchd3/6,* gol [RNF149], Cdk12, β Spectrin [SPTBN1] variants enriched in HLHS	**KD [RNAi], Screening**	Reduction in fractional shortening (by ~ 25%), Reduction in cardiac F-actin and myosin (by ~ 0.6), Increase in systolic diameter (by ~ 50 μm) (systolic dysfunction), Reduction in CO, arrhythmogenicity, Reduction in ATP synthase (Complex V) activity and ATP levels (by ~ 30 pmol ATP/μg protein), Defects in mitochondrial fission/fusion (**Adult**)	Birker et al., 2023 [57]

Table S4. Genes involved in Cardiac progenitor migration, alignment and Dorsal vessel assembly during Drosophila melanogaster embryonic development: Mutations and Phenotypes.

Notes: ADAM, A Disintegrin and Metalloproteinase; ADv, Anterior Dorsal vessel; AM, Alary muscle; Arm, Armadillo; As, Amnioserosa; CB, Cardioblast; CCM, Collective cell migration; CHD, Congenital heart disease; COM, Cardiac outflow muscles; Cdc42, Cell division control protein 42; Chr 21, Chromosome 21; Col6A2, Collagen Type VI α2 chain; DE-Cadherin, Drosophila E-Cadherin; DN, Dominant negative; DS, Down Syndrome; Dg, Dystroglycan; Dlg, Disks-large; Dscam, Down syndrome Cell adhesion molecule; Dv, Dorsal vessel; ECM, Extracellular matrix; EPC, Eve+ pericardial cell; Eve, Even-skipped; GPCR, G-protein coupled receptor; Gia, G-protein coupled receptor in aorta; HANC, Heart-anchoring cells; KD, Knockdown; Kuz, Kuzbanian; LE, Leading Edge; LE, Leading edge; Lb, Ladybird; MMP, Matrix metalloproteinase; NetA, netrin A; NetB, Netrin B; OFT, Outflow tract; OHS, Outflow hanging structure; Odd, Odd-skipped; PC, Pericardial cell; PDv, Posterior Dorsal vessel; Prc, Pericardin; RNA, Ribonucleic acid interference; Robo2, Roundabout2; Sli, Slit; Slit, Robo, Roundabout; Src42A, How, Held out wings; T2, Thoracic segment 2; Timp, Tissue inhibitor of metalloproteinase; Vkg, Viking; WT, Wild type; Wun, Wunen; Wun2, Wunen2; Zip, Zipper; dDAAM, Dishevelled-associated activator of morphogenesis; dSUR, Drosophila Sulfonylurea receptor; lanA, Laminin A; miR-1, micro Ribonucleic acid (RNA)-1; mmp1, Matrix metalloproteinase 1; mmp2, matrix metalloproteinase 2; mys, myospheroid; scb, scab; slo, slowpoke; svp, sevenup; tin, tinman; zfh1, zinc-finger homeodomain 1; αPS3, α subunit integrin chain; βPS, β subunit integrin chain; µm, micrometers.

**Gene**	**General Information**	**Mutation**	**Mutation phenotype**	Study (Reference)
αPS3 (scb), βPS (mys), lanA	*αPS3* associates with *βPS,* implicated in tissue invagination/ movement, Morphogenesis of cardiac, salivary gland, trachea, midgut, ventral cord, As tissue	**Null mutation, Deficiency**	No effect on ADv (aorta), PDv (aorta, heart chamber) with some AM detachment and subsequent failure of maturation (**zygotic *mys* mutation with maternal βPS**), Variable defects ranging from Reduction/Disruption of PC arrangement to Complete CB/PC dissociation with random migration patterns (***scb***, *lanA*) (**Embryo**)	Stark et al., 1997 [58]
Apt	Implicated in cardiac development (late phase cardiac progenitor migration, Dv assembly)	**Null mutation, Deficiency**	Increased lethality at late embryonic/early larval stages (14% embryos hatch successfully), Abnormal Dv morphology with absent CB/PC (**Embryo**)	Su et al., 1999 [59]
Sli	Slit, Robo accumulate at dorsal midline, between contralateral CB rows during migration; both single and combination mutations evaluated	**Null mutation, Deficiency**	No effect on CB migration, Severe defects in midline CB alignment (gaps, intercalation, double rows), Disruption in CB/PC association, Disruption in cell-polarity marker localization (Dlg, Arm, Toll, α-Spectrin) (**Embryo**)	Qian et al., 2005b [60]
Robo, Robo2			No effect on CB migration, Mild defects in midline CB alignment, Absence of Slit between opposing CB (*Robo, Robo2*), Severe defects in CB alignment (gaps, intercalation, double rows) (*Robo/Robo2*) (**Embryo**)	
Sli, Robo, Robo2, αPS3 (scb), βPS (mys), lanA	Implicated in dorsal closure (ectodermal epithelium migration); cardiac LE in contact with ectoderm LE; both single and combination mutations evaluated	**Null mutation**	Variable defects in dorsal closure and Dv (delayed migration, gaps, blisters, twists, midline crossing of cardiac progenitors), Highest phenotype severity (2.6), with *sli/scb*, *robo2* mutations (**Embryo**)	MacMullin and Jacobs, 2006 [61]

Gene	General Information	Mutation	Mutation phenotype	Study (Reference)
Kuz	ADAM, implicated in the proteolytic shedding of transmembrane proteins, Notch lateral inhibition, cardiac and vascular development	**Null mutation**	Variable defects ranging from Rudimentary/missing heart (4 lines), Cell-cycle/cytokinesis with disruption in CB alignment (4 lines), Disorganized heart with disruption in CB alignment (7 lines) to Asymmetric cell division (2 lines), Hyperplastic heart with 2-fold increase in all CB (by ~ 80) and reduction in *zfh1*+ PC (by ~ 44), *Odd*+ PC (by ~ 44), lymph gland cells (8 lines) (**Embryo**)	Albrecht et al., 2006 [62]
Sli, Robo, Robo2, Dg	Implicated in cardiac lumen formation along with How, Dg; Contralateral CB interact via shape changes and filopodia formation	**Null mutation, Deficiency**	Absence of cardiac lumen formation with vacuoles between opposing CB, Absence of CB shape changes (filopodia, CB/ectoderm detachment) during migration (with ***Slit***, ***Robo, Robo2***), Variable defects ranging from WT Dv appearance with little/no cardiac lumen formation (~ 80% of individuals), Severe Dv defects (migration, gaps, twists) (~ 5% of individuals) to No effect on Dv (~ 15% of individuals with ***Dg***) (**Embryo**)	Medioni et al., 2008 [63]
Robo	DE-Cadherin interactions mediate adhesive effects between contralateral CB, Slit/Robo interactions mediate repulsive effects between contralateral CB (Robo overcomes DE-Cadherin adhesion), facilitate cardiac lumen formation	**Null mutation**	Mild defects in CB migration, Severe defects in CB alignment (gaps), Absence of cardiac lumen formation with inappropriate adherence between contralateral CB (**Embryo**)	Santiago-Martínez et al., 2008 [64]
Shg			Absence of cardiac lumen formation with extracellular space accumulating between contralateral CB (**Embryo**)	
Sli		**Overexpression**	Ectopic cardiac lumen formation (discontinuous, 2 lumens) (**Embryo**)	
Robo			Severe defects in CB alignment (loss of cell contact in ventral, dorsal surfaces (**Embryo**)	
Shg			Absence of cardiac lumen formation with inappropriate adherence between contralateral CB (**Embryo**)	
Sli, Robo	Implicated in HANC CB migration, facilitating interactions between HANC (*Lb*+), aorta, COM	**Null mutation, KD** [RNAi]	Delay in HANC CB migration (most severe with *Shg,* 90% of HANC malpositioned anterior to T2) (*Slit, Robo, Shg*) (**Embryo**)	

Gene	General Information	Mutation	Mutation phenotype	Study (Reference)
Shg	(pharyngeal mesoderm), OHS (specialized *Eve*+ PC); Formation of an OFT (T2)		Failure of HANC/ADv attachment, No effect HANC/COM attachment, Reduction in HANC numbers (**Embryo**)	Zmojdzian et al., 2008 [65], Zmojdzian et al., 2018 [3]
Eve			Reduction in COM length (by ~ 9.1 μm with EPC ablation, by ~ 7 μm with partial EPC loss) (**Embryo**)	
Cdc42	Implicated in K+ channel function (*dSUR, slo)*, miR-1-mediated regulation by tinman, impact on heart function; both single and combination mutations evaluated	**Null mutation, Deficiency, DN**	Increase in diastolic interval (by ~ 2), No effect on systolic interval, Increase in arrhythmia index (maximum by ~ 3), Disruption in myofibril arrangement (increased severity in *Cdc42/tin*), Reduction in *dSUR* mRNA (maximum by ~ 0.6)*, slo* mRNA *(*by ~ 0.4) in *Cdc42/tin* (**Adult**)	Qian et al., 2011 [66]
Dscam, Col6A2	Increased expression of genes in Distal chromosome 21 implicated in DS heart defects in DS; both single and combination mutations evaluated	**Overexpression, Screening**	Increase in heart failure rate after electrical pacing-induced stress (by ~ 35% for ***Dscam, Col6A2***, by ~ 60% for ***Dscam/Col6A2)*** (**Adult**)	Grossman et al., 2011 [67]
αPS3 (scb), βPS (mys), lanA	Implicated in cardiac LE formation, cardiac LE motility; both single and combination mutations evaluated	**Null mutation, KD [**RNAi**]**	Variable defects, including CB displacement (most severe with *mys*, 16 individuals), Reduction in **Apically/Non-Apically localized Slit** ratio (most severe with *scb*, at 1.8 ± 0.1), Reduction in **LE activity (most severe with *scb*, at** 16% ± 3**),** Absence of cardiac lumen formation **(*scb*)** (**Embryo**)	Vanderploeg et al., 2012 [68]
Cdc42	Implicated in cardiac lumen formation, via interactions with dDAAM (actin-organizing formin), *Zip* (non-muscle myosin II)	**Null mutation**	Absence of cardiac lumen formation, Disruption in CB alignment (*Cdc42*), Absence of dorsal closure (most common with *Cdc42/zip,* at **33%**), Variable defects in Dv structure (most common with *Cdc42/tin,* at **86.2%**) (**Embryo**)	Vogler et al., 2014 [69]
Wun, wun2	Lipid phosphatases, implicated in cardiac LE motility with ectoderm LE migration (dorsal closure) medial to cardiac LE	**Null mutation, Deficiency**	Variable defects in dorsal closure and Dv structure ranging from delayed ectoderm LE migration (at > 100 min), gaps, multiple lumens, loose PC/CB attachment to luminal ectoderm/As remnants (Arm) with disruption in midline CB assembly (**Embryo**)	Haack et al., 2014 [70]

**Gene**	**General Information**	**Mutation**	**Mutation phenotype**	Study (Reference)
Sli, Robo, Netrin, Dscam1	Dscam1 (Slit, Netrin receptor) implicated in CB filopodia, lamellipodia formation; CCM requires cell-cell adhesion; both single and combination mutations evaluated	**Null mutation, KD [**RNAi**]**	Variable defects in LE, ranging from Reduction in migration velocity (by ~ 0.6 µm/min for ***Sli,*** by ~ 0.4 µm/min for *Robo,* by ~ 0.4 µm/min for *netA/netB,* by ~ 0.2 µm/min for *Dscam1*), Reduction in filopodia/segment (by ~ 8 for *Slit, Robo,* by ~ 5 for *netA/netB,* by ~ 6 *for Dscam1*) to Reduction in LE lamellipodial activity (by ~ 100% for ***Sli,*** ~ 80% for *Robo,* by ~ 30% for *netA/netB,* by ~ 20% for *Dscam1*) (**Embryo**)	Raza and Jacobs, 2016 [8]
Gia	Methuselah-like GPCR, implicated in midline CB adhesion, CB/PC adhesion	**Null mutation**	Increased lethality at late embryonic/early larval stages (100% of all individuals fail to hatch into 1st instar larvae) (**Embryo, Larva**)	Patel et al., 2016 [71]
			'**Broken-hearted**' phenotype: Dissociation between CB and PC with disruption in cell-cell adhesion protein distribution, Disruption in CB alignment (**Embryo**)	
mmp1, mmp2	Implicated in regulation of cardiac LE activity, cardiac lumen formation (*mmp2*), cardiac lumen expansion (*mmp1*); both single and combination mutations evaluated	**Null mutation, KD [**RNAi**]**	Disruption in CB arrangement (***mmp1, mmp2***), Cardiac lumen formation with reduced diameter (*mmp1*), Absence of cardiac lumen formation (*mmp2, mmp1/mmp2*), Absence of CB shape changes/filopodia (75% of individuals) (**Embryo**)	Raza et al., 2017 [72]
			Variable defects in LE, ranging from Reduction in migration velocity (by ~ **0.3** µm/min for *mmp2,* by ~ **0.5** µm/min for *mmp1/mmp2*), Reduction in filopodia/segment (by ~ 3 for *mmp1,* ~ 6 for *mmp2,* ~ 7 for *mmp1/mmp2*) to Reduction in LE Lamellipodial activity (by ~ 40% for *mmp1,* ~ 60% for *mmp2,* ~ 40% for *mmp1/mmp2*) (**Embryo**)	Raza et al., 2017 [72]
Src42A	Src kinase, implicated in the phorphorylation of integrin cell-cell adhesion, Amnioserosa apoptosis	**Null mutation, DN**	'**Open heart'** phenotype: Absence of PDv CB migration with no effect on ADv CB migration, Absence of cardiac LE activity, Persistence of As near the midline (**Null mutation**); Disruption in CB aligment with aggregations, No effect on CB migration (**DN**) (**Embryo**)	Vanderploeg and Jacobs, 2017 [73]

Gene	General Information	Mutation	Mutation phenotype	Study (Reference)
Timp	Implicated in regulation of MMP activity, ECM turnover, AM attachement to Dv	**Null mutation, KD [**RNAi**]**	**'Ectopic ECM'** phenotype: Longitudinal AM arrangement along Dv, Disruption in Prc arrangement with ectopic Prc beyond Dv margins, No effect on Prc levels, Disruption in somatic muscle alignment (midline crossing) (**Embryo**)	Hughes et al., 2020 [74]
		**Overexpression**	**'Cardia Bifida'** phenotype: Midline tearing/Cardia bifida (maximum rate of 33.3%), **Incomplete Dv** (maximum rate of 76.9%) or Luminal Actin deposits, Increase in Vkg density throughout Dv, Luminal Vkg deposits **with** disruption in Vkg distribution, No effect on Prc levels (**Embryo**)	
Mmp1, mmp2	Luminal domain restriction, Regulation of ECM distribution (Vkg, Prc)	**Gene deletion, KD [**RNAi**]**	**'Cardia Bifida'** phenotype: Disruption in adhesion junction and myofibril **arrangement** (*mmp1*), Midline tearing and **Cardia bifida** (maximum rate of 13.8%) (*mmp2*), **Incomplete Dv** (maximum rate of 17.2%), Luminal and abluminal Vkg plaques (**Embryo**)	Hughes et al., 2020 [74]
		**Overexpression**	**'Ectopic ECM'** phenotype: Disruption in Prc arrangement with non-uniform distribution, Diminished/Absent Adv (aorta), Disruption in/Absence of AM, PC arrangement (**Embryo**)	

Table S5. Genes involved in the establishment of segmentation and polarity during Drosophila melanogaster embryonic development: Mutations and Phenotypes.

Notes: A5, Abdominal segment 5; A8, Abdominal segment 8; Abd-A, Abdominal-A; Adv, Anterior Dorsal vessel; BMP, Bone morphogenetic protein; BX-C, Bithorax Complex; CB, Cardioblast; CO, Cardiac output; Ci, Cubitus interruptus; DWnt4, Drosophila Wingless-related integration site 4; Dfz2, Dfrizzled-2; Dmef2, Drosophila myocyte enhancer factor 2; Dome, domeless; Dpp, Decapentaplegic; Dv, Dorsal vessel; EGF, Epidermal growth factor; EGFR, Epidermal growth factor receptor; Eve, Even-skipped; FGF, Fibroblast growth factor; FGFR, Fibroblast growth factor receptor; Fz, Frizzled; Fz2, Frizzled2; Hh, Hedgehog; JAK, Janus kinase; KD, Knockdown; MAPK, Mitogen-associated protein kinase or Extracellular signal-regulated kinase (ERK); Odd, Odd-skipped; PC, Pericardial cell; PDv, Posterior Dorsal vessel; PVR, Platelet-derived growth factor/Vascular endothelial growth factor receptor; Prc, Pericardin; RNAi, Ribonucleic acid interference; Rho, Rhomboid; Stat, Signal transducer and activator of transcription; Stat92E, Signal transducer and activator of transcription 92E; TS, Temperature sensitive; Tkv, Thickveins; UAS, Upstream activation sequence; Upd, Unpaired; VEGFR, Vascular endothelial growth factor receptor; Vegf, Vascular endothelial growth factor; Wg, wingless; Wnt, Wingless-related integration site; dpERK, Activated dually phosphorylated Extracellular signal-regulated kinase (ERK); h, hours; lb, ladybird; pyr, pyramus; spdo, sanpodo; svp, Sevenup; ths, thisbe; tin, tinman; zfh1, zinc-finger homeobox 1.

Gene	Signaling pathway	Mutation	Mutation phenotype	Study (Reference)
Wg	Wg (Wnt) signaling	**Conditional mutation [TS]**	Disruption in somatic muscle arrangement, Defects in visceral muscle patterning; Variable effects on cardiac development ranging from No heart formation **(~ 3-5 h)**, Severe effects with reduction in CB/PC number (80% reduction in *Eve*+ PC) **(~ 4-4.5 h)** to No effects on heart formation **(~ 5 h and onwards)** (**Embryo**)	Wu et al., 1995 [75]
Wg, Hh	Wg (Wnt) signaling, Hedgehog (*Hh*) signaling (maintains *Wg* expression)	**Conditional mutation [TS]**	Moderate defects in somatic muscle patterning, Variable effects on cardiac development ranging from Reduction in cardiac cell numbers (by ~ 40%) (*hh*) **(~ 3.5-4.5 h)**, Reduction in cardiac cell numbers (by ~ 80%) (*Wg*) **(~ 4.0-4.5 h)**, No heart formation **(~ 3.5-4.5 h)** to No effect on Dv formation **(~ 5.5-6.5 h)** (*Hh*) **(Embryo)**	Park et al., 1996 [76]
	Wg (Wnt) signaling, Hedgehog (*Hh*) signaling (maintains *Wg* expression)	**Overexpression**	Variable effects on cardiac development ranging from Increase in cardiac progenitor numbers with 2-fold increase in *Eve*+ populations **(~ 3-4 h)** (*Wg*), 4-fold increase in *Eve*+ populations **(~ 3-4 h)** (*Hh*) to No effect in heart formation **(~ 5-6 h)** (**Embryo**)	Park et al., 1996 [76]
Tkv, tin	Dpp (BMP) signaling, Tkv is a Type I receptor for Dpp	**Overexpression**	Ectopic *tin* expression in visceral mesoderm with ectopic heart tissue formation in the ventral visceral mesoderm (**Embryo**)	Yin and Frasch, 1998 [77]
fz, Dfz2	Wg (Wnt) signaling	**Conditional mutation [Radiation], Null mutation, Deficiency**	Increased lethality after hatching, Defects in in endoderm (midgut), mesoderm and ectoderm (cuticle) development with absence of RP2+ neurons and cardiac development (similar to *Wg*), Absence of *Eve*+ cardiac progenitors (**Embryo**)	Bhanot et al., 1999 [78]

Gene	Signaling pathway	Mutation	Mutation phenotype	Study (Reference)
fz2	Wg (Wnt) signaling	**Null mutation [Amber mutation]**	Absence of Wg signaling, Reduction in/Absence of cardiac progenitors, Absence of *Eve*+ cardiac progenitors, Defects in endoderm (midgut), mesoderm and ectoderm (cuticle, wings, wing imaginal disks) development with absence of RP2+ neurons (**Embryo**)	Chen and Struhl, 1999 [79]
Spdo, Numb	Notch signaling	**Null mutation**	Defects in CB diversification ranging from Increase in *svp*+ CB number (to ~ 8/segment), No effect on *tin*+ CB (*spdo*) to Apparent increase in *tin*+ PC with absence of physiological gaps in *tin*+ CB alignment (*Numb*) (**Embryo**)	Gajewski et al., 2000 [80]
Wg, Dpp	Wg (Wnt) signaling, Dpp (BMP) signaling	**Ectopic expression [ectoderm]**	Ectopic *tin* expression at intersections of Wg (Wnt) and Dpp signaling with ectopic heart tissue formation (**Embryo**)	Lockwood and Bodmer, 2002 [81]
Hh	Hedgehog (*Hh*) signaling, controls Ras/MAPK signaling via regulation of EGFR-associated protease Rho (*Rho*)	**Conditional mutation [TS]**	Reduction /Absence of *Eve*+ cardiac progenitors, Expansion of *lb* expression (mesoderm) (**Embryo**)	Liu et al., 2006 [82]
		**Overexpression**	Expansion in *Eve* expression, Absence of *lb* expression (mesoderm); Overexpression of Ci (Hh signaling antagonist) leads to Reduction in *Eve* expression, Expansion of *lb* expression (**Embryo**)	Liu et al., 2006 [82]
Dpp	Dpp (BMP) signaling, regulates CB diversification via *zfh1* and via *zfh1*-independent mechanisms, implicated in spatial restriction of PC in the dorsal mesoderm	**Null mutation, Deficiency**	Expansion in *tin*+, *Odd*+ expression (*zfh1*), Expansion in *mid* expression (ventral) with ectopic *tin* expression, Reduction in *Eve+, Krüppel*+ expression (*zfh1*), No effect on *tin, Odd*-mediated PC specification (*zfh1*), Expansion of PC into the ventral region of dorsal mesoderm (**Embryo**)	Johnson et al., 2007 [83]
			Reduction in average pulse distance/rate (~ 44%), Reduction in CO **(Larva)**	
Vegf	VEGF/VEFGR/PVR signaling, implicated in hemocyte migration and survival, hemocytes accompany migrating cardiac progenitors to the midline	**Null mutation**	No effect on PDv (heart chamber) structure, Reduction in systolic motion, Reversal in ostial, aortic valve opening (aortic valve opens before ostial valves) (**Embryo**)	Wu and Sato, 2008 [84]
pyr	FGF/FGFR signaling (*pyr* expressed in dorsal regions, *ths* expressed in ventral regions)	**Null mutation, Deficiency**	Defects in mesoderm migration alongside ectoderm, mesoderm aberrant with multilayer formation, Severe defects in dorsal mesoderm specification, Reduction/Absence of *Eve*+ expression (**Embryo**)	Kadam et al., 2009 [85], Dorey and Amaya, 2010 [86]

Gene	Signaling pathway	Mutation	Mutation phenotype	Study (Reference)
ths			Defects in mesoderm migration alongside ectoderm, mesoderm aberrant with multilayer formation, Subtle defects in *Eve* expression with reduction in *Eve*+ cell number/hemisegment (~ 1) (**Embryo**)	
Htl			Defects in mesoderm migration alongside ectoderm with absence of visceral mesoderm (**Embryo**)	
pyr, ths	FGF/FGFR signaling	**Overexpression**	Mesoderm migration alongside ectoderm occurs, aberrant with multilayer formation (*pyr*), No effect on mesoderm layer formation (*ths*), Ectopic dpERK (activated MAPK) expression (*pyr*) (*ths*), Ectopic *Eve*+ cell specification (from ~ 3-4 cell/hemisegment in 12 hemisegments to ~ 6 to 15 cell/hemisegment in 14 hemisegments) (*pyr*) (*ths*) (**Embryo**)	Kadam et al., 2009 [85]
upd, Stat92E	JAK/Stat signaling (Upd/Dome) acts via the transcriptional effector Stat92E, implicated in regulation of *tin* expression in the dorsal mesoderm during its restriction within the cardiac mesoderm	**Null mutation**	Loss of CB cell-cell adhesion with CB aggregation and incomplete lumen formation (*upd),* Failure of tin restriction within the dorsal mesoderm (*Stat92E*), No effect on cardiac cell proliferation, Defects in CB diversification with Increase in *tin*+ PC number (by ~ 20) and Increase in *Odd*+ PC number (by ~ 2 cells/hemisegment) (**Embryo**)	Johnson et al., 2011 [87]
DWnt4	Wg (Wnt) signaling (non-canonical, *svp*+ CB)	**Null mutation, Deficiency**	Reduction in *Eve*+ PC (Stages 10/11), Reduction in *Odd*+ PC number (only ~ 63% of individuals) (Stage 16), Loss of *svp* expression (only some segments), Disruption in Prc expression alone (~ 50% of individuals) or associated with disruption/loss of *Dmef2*+ CB; overall, *DWnt4* not as severe as *Wg* mutation (**Embryo**)	Graba et al., 1995 [88], Tauc et al., 2012 [89]
DWnt4	Wg (Wnt) signaling (non-canonical, *svp*+ CB)	**Overexpression**	Severity increases with number of *DWnt4* copies expressed, ranging from mild disruption in *Odd, svp* (one copy) to more widespread disruption in *svp*+ CB (~ 80% of embryos) and *Odd*+ (~ 90% of embryos) PC fate (**Embryo**)	Graba et al., 1995 [88], Tauc et al., 2012 [89]
pygo	Wg (Wnt) signaling (canonical), *pygo* implicated in cardiac valve cell formation independent of Wg (Wnt) signaling	**Null mutation, KD [RNAi]**	No effect on cardiac valve precursor cells (**Embryo**)<br>Absence of cardiac valve cell differentiation with lack of high-density myofibrils, Absence of physiological PDv (heart chamber) wall thickening in the valve region, Loss of normal heart chamber constriction at valve site (valve site dilation) with increase in valve/heart chamber diameter ratio (~ 0.2) (**Adult**)	Tang et al., 2014 [90]

Gene	Signaling pathway	Mutation	Mutation phenotype	Study (Reference)
		**Overexpression**	Disruption in myofibril arrangement (**Adult**)	Tang et al., 2014 [90]
Wnt4	Wg (Wnt) signaling (canonical), Highest expression in ostia cells	**Null mutation, Deficiency**	Misalignment of CB (after Stage 13 with normal Dv in ~ 30% of individuals [Stage 15]), No effect on CB specification, No effect on *svp, Wg* expression, Absence of unique morphology (constricted, elongated) of ostia progenitor cells (*svp*+ CB in PDv), Absence of ostia formation (**Embryo**)	Chen et al., 2016 [91]
Wg, svp, Abd-A	Wg (Wnt) signaling (canonical), *Wg/CG8147* expression distinguishes ostial cells	**Null mutation, Conditional mutation [TS], KD [RNAi]**	Reduction in PDv (heart chamber) diameter (*Abd-A*), No effect in PDv lumen formation (*svp*), Absence of unique ostia progenitor cell morphology (constricted, elongated) (*svp*+ CB in PDv), Reduction in axial ratio of PDv (heart chamber) *svp*+ CB (ostia cells) (~ 0.7 for *Abd-A, svp,* 0.5 for *Wg* KD, Variable effects for TS *Wg* mutation), Absence of *CG8147* expression (*Abd-A*) (*svp*) (**Embryo**)	Trujillo et al., 2016 [92]
Abd-A	Homeotic gene, part of the Bithorax Complex (BX-C) (Range: A5-A8), implicated in ostia formation along with Wg (Wnt) signaling	**Overexpression**	Ectopic *Wg, CG8147* in ADv (aorta) *svp*+ CB, Increase in ADv (aorta) axial ratio of *svp*+ CB (ostia cells) (~ 0.5), Further increase in PDv (heart chamber) *svp*+ CB with ectopic *Wg/CG8147* expression (**Embryo**)	Trujillo et al., 2016 [92]
Egfr, rho, edl	EGF/EGFR signaling, implicated in diversification of CB (gCB, oCB), PC (*Odd*+ PC, gCB fate); edl promotes oCB cell fate via PntP2 inhibition, while PntP1 and mid are expressed in gCB and antagonize oCB fate	**Null mutation, DN**	Reduction in gCB number (by ~ 50) with no effect on oCB number (gCB now comprise ~ 50% of total CB, as opposed to 73% in WT) (*rho*), Reduction in *Odd*+ PC number (by ~ 30) with no effect on *Eve*+ PC number (*rho*), Severe reduction in gCB number with almost all remaining CB acquiring oCB identity (*Egfr*), Reduction in *Odd*+ PC (by ~ 30) with no effect on *Eve*+ PC numbers (*Egfr* DN), Reduction in oCB (now comprise ~ 7% of all CB) with no effect/mild increase in gCB number (by ~ 10) (**Embryo**)	Schwarz et al., 2018 [6]

Table S6. Genes involved in the Development of the animal body plan during Drosophila melanogaster embryonic development: Mutations and Phenotypes.

Notes: A1, Abdominal segment A1; A2, Abdominal segment A2; A3, Abdominal segment A3; A4, Abdominal segment A4; A5, Abdominal segment A5; A6, Abdominal segment A6; A7, Abdominal segment A7; A8, Abdominal segment A8; ADv, Anterior dorsal vessel; AM, Alary muscles; ANTP-C, Antennapedia Complex; AP, Anteroposterior; APF, After pupa formation; Abd-A, Abdominal-A; Abd-B, Abdominal-B; Antp, Antennapedia; BX-C, Bithorax Complex; CB, Cardioblast; Col, Collagen; Dv, Dorsal vessel; Ec, Ecdysone; EcR, Ecdysone receptor; Hh, Hedgehog; KD, Knockdown; KO, Knockout; Naca, Nascent-associated polypeptide complex, alpha subunit; Odd, Odd-skipped; PC, Pericardial cell; PDv, Posterior dorsal vessel; Prc, Pericardin; RNAi, Ribonucleic acid interference; Src, Sex combs reduced; T1, Thoracic segment T1; T2, Thoracic segment T2; T3, Thoracic segment T3; TrxG, Trithorax Group genes; Ubx, Ultrabithorax; WT, Wild type; Wg, Wingless; bic, bicaudal; svp, Sevenup; tin, tinman; μm, micrometers.

**Gene**	**Mutation**	Mutation Effects/Syndrome	Study (Reference)
Abd-A	**Null mutation**	Reduction in PDv (heart chamber) diameter which is now similar to ADv (aorta) diameter, Loss of cardiac CB specification, Absence of cellular dimorphism between ADv (aorta) and PDv (heart chamber) (smaller volume cells present throughout), *tin*+ expression pattern continuous throughout the Dorsal vessel, Late *Wg* expression in *svp*+ CB undetectable (**Embryo**)	Lo et al., 2002 [93], Rosales-Vega et al., 2024 [94]
Abd-A	**Ectopic expression**	Increase in ADv (Aorta) diameter which is now similar to PDv diameter (heart chamber), Late *Wg* expression in *svp*+ CB detectable throughout (**Embryo**)	Lo et al., 2002 [93]
Abd-A	**Null mutation**	Loss of cardiac CB identity with absence of heart chamber formation, Absence of cellular dimorphism between ADv (aorta) and PDv (heart chamber) (smaller volume cells throughout) (**Embryo**)	Lovato et al., 2002 [95]
	**Ectopic expression** [ectoderm]	Absence of Dorsal closure, Absence of a linear Dv, No effect on the cellular dimorphism between ADv (aorta) (smaller volume) and PDv (heart chamber) (larger volume, rounded) CB present (**Embryo**)	Lovato et al., 2002 [95]
	**Ectopic expression** [mesoderm]	Loss of aortic CB specification and acquisition of cardiac CB identity, Absence of cellular dimorphism between ADv (aorta) and PDv (heart chamber) (larger volume, rounded cells throughout), Ectopic ostia formation, Increase in non-ostial *svp*+ CB size (~ 3 μm), Suppression of *Ubx* expression (**Embryo**)	Lovato et al., 2002 [95]
Abd-A	**Ectopic expression** [Dv]	Loss of aortic CB specification and acquisition of cardiac CB identity in A4-A5, Ectopic ostia formation, No effect on *tin, svp* expression (**Embryo**)	Ponzielli et al., 2002 [96]
Abd-A	**Null mutation, Deficiency**	Absence of cellular dimorphism between ADv (aorta) and PDv (heart chamber) (smaller volume cells throughout) with aortic CB identity, Absence of ostia formation, No effect on *tin, svp* expression, *Ubx* expression extends posteriorly into the presumptive heart region (*Abd-A*) (**Embryo**)	Ponzielli et al., 2002 [96]

Gene	Mutation	Mutation Effects/Syndrome	Study (Reference)
Abd-A/Ubx	**Null mutation, Deficiency**	No effect on early cardiac development stages, Impairment in CB differentiation with no effect on CB number/segment, Absence of AP cell polarization, Absence of cellular dimorphism between ADv (aorta) and PDv (heart chamber) (smaller volume cells throughout), No effect on *tin, svp* expression (**Embryo**)	Ponzielli et al., 2002 [96]
Abd-A, Ubx	**Null mutation, Deficiency**	Absence of cellular dimorphism between ADv (aorta) and PDv (heart chamber) (smaller volume cells throughout) with aortic CB identity (except for A1), ADv-like Prc localization, Loss of *svp*+ CB, Ectopic *Odd*+ *Col*+ *Prc*– PC, Ectopic *Hh* expression in PDv (**Embryo**)	Perrin et al., 2004 [7]
Abd-A	**Null mutation, Deficiency**	Absence of cellular dimorphism between ADv (aorta) and PDv (heart chamber) (smaller volume cells throughout), Absence of heart chamber formation, Expansion in *Ubx* expression (**Embryo**)	Perrin et al., 2004 [7]
Abd-A, Ubx	**Null mutation, Deficiency**	Absence of CB diversification with loss of *svp*+ CB (*Antp*– domains), Reduction in CB number (~ 20), Posterior expansion in *Antp* expression (**Embryo**)	Ryan et al., 2005 [15]
Abd-A	**Null mutation, Deficiency**	Absence of heart chamber formation, No effect on *svp* expression, Posterior expansion in *Ubx* expression (**Embryo**)	Ryan et al., 2005 [15], Monier et al., 2005 [97]
Abd-A	**Overexpression** [mesoderm]	Absence of cellular dimorphism between ADv (aorta) and PDv (heart chamber) (larger volume, rounded cells throughout), ADv (aorta) acquires PDv (heart chamber) identity, Expansion in *svp* expression with increase in *svp*+ clusters (by 3) with expansion in *Wg* expression in *svp*+ CB, Increase in CB number (by ~ 16) (**Embryo**)	Ryan et al., 2005 [15]
Abd-A	**Overexpression** [metamorphosis]	ADv (aorta) segments A1-A4 acquire PDv (heart chamber) identity, Disruption in myofibril arrangement, Reduction in cardiac chamber wall thickness (**Embryo**)	Monier et al., 2005 [97]
Abd-A	**KO**	No effect on AM formation (**Embryo**)	LaBeau et al., 2009 [98]
Abd-A	**Ectopic expression**	Increase in Dv length, Increase in AM number (9.50 ± 0.23 compared to 7 in WT) (**Embryo**)	LaBeau et al., 2009 [98]
Abd-A, Ubx	**KO**	Reduced AM formation with remaining AM exhibiting aberrant localization (**Embryo**)	LaBeau et al., 2009 [98]
**EcR**	**Ec/EcR binding inhibition**	Inhibition of cardiac remodeling, PDv (heart chamber) maintains larval morphology, Absence of *Wg* expression in *svp*+ CB, Absence of histolysis in A6-A7, Absence of remodeling in *Abd-A*+ CB (**Embryo**)	Monier et al., 2005 [97]

Gene	Mutation	Mutation Effects/Syndrome	Study (Reference)
Abd-B	**Ectopic expression** [mesoderm, CB]	Ectopic *Abd-B* expression suppressing Dv, somatic muscle development with absence of CB (mesoderm); No effect on CB development (CB) (**Embryo**)	Lo et al., 2002 [93], Rosales-Vega et al., 2024 [94]
Abd-B	**Ectopic expression** [mesoderm]	Suppression of cardiac morphogenesis, myogenesis with defects in somatic muscle formation; Decrease in *tin*+ CB begins mid-development [Stage 13], with very few *tin*+ CB left by late development [Stage 16] (embryo, larva, early pupa); Absence of heart (**Embryo**)	Lovato et al., 2002 [95]
Abd-B	**Null mutation**	Increase in PDv (heart chamber) diameter, Increase in CB number (total number of nuclei increases from 104 to 116), Disorganization in CB arrangement, Dilation of heart terminus (A6-A8) (**Embryo**)	Lo et al., 2002 [93], Schroeder et al., 2022 [99]
Naca, bic	**KD [RNAi]** [inducible]	Reduction in diastolic diameter (~ 40 μm), Reduction in systolic diameter (~ 20 μm) (**Embryo**)	Schroeder et al., 2022 [99]
		Severe defects **(‘No heart’** phenotype) when KD applied throughout development, Dv (aorta, heart chamber) histolysis (20 h APF, complete 40 h APF) with absent Prc, cardiac cell dispersal and fat cell accumulation (*Naca, bic*) (**Pupa, Adult**)	
Naca, bic	**KD [RNAi]** [inducible only in CB, PC]	Disorganization in sarcomere arrangement with gaps between circumferential myofibrils, Reduction in diastolic diameter (~ 10 μm), No effect in systolic diameter, Reduction in fractional shortening (~ 0.1), Reduction in systolic interval (~ 0.05) all leading to reduced contractility [CB] (**Adult**)	Schroeder et al., 2022 [99]
		Increase in systolic diameter (~ 10 μm) with no effects on contractility [PC] (**Adult**)	
Naca	**KD [RNAi]**	Ectopic Dv *Abd-B* expression (**Larva**, **Pupa**) leading to aberrant histolysis (**Metamorphosis**)	Schroeder et al., 2022 [99]
		‘**No heart’** phenotype (**Adult**)	
Abd-B, Naca	**KD [RNAi]**	Rescue of the *Naca* KD-induced **‘No heart’** phenotype via *Abd-B* KD: Restoration of myofibril arrangement albeit with reduction in diastolic diameter by ~ 20 μm, No effect in systolic diameter, Reduction in fractional shortening ratio by ~ 0.1 (**Adult**)	Schroeder et al., 2022 [99]
***Scr, Antp*, BX-C (*Ubx, Abd-A, Abd-b*)**	**Null mutation, Deficiency**	Severe effects in embryonic development, Abnormal dorsal closure, Absence of PDv (heart chamber) specification with Dv acquiring ADv (aorta) characteristics, Expansion of *tin*+ expression, Loss of *svp*+ expression, ADv-like Prc localization, *Odd*+ *Prc*–PC acquire lymph gland identity (**Embryo**)	Perrin et al., 2004 [7], Rosales-Vega et al., 2024 [94]

Gene	Mutation	Mutation Effects/Syndrome	Study (Reference)
Antp	**Null mutation**	No effect in CB number per segment, Mild effect on CB differentiation (A1 *svp*+ CB now express *tin*) (**Embryo**)	Lo et al., 2002 [93], Perrin et al., 2004 [7], Rosales-Vega et al., 2024 [94]
Antp	**Overexpression**	Expansion in *svp* expression (increase in *svp*+ clusters from 7 to 10) with no associated expansion of *Wg* expression, No effect on CB number, Absence of *Ubx* causes posterior expansion of *Antp* expression (ectopic lymph/ ring gland formation) (**Embryo**)	Ryan et al., 2005 [15]
Ubx	**Null mutation, Deficiency**	No effect on heart CB differentiation, Aorta CB differentiation disrupted in T3-A1 and A2, Disruption in ADv structure (polarization defects), Disruption in ADv PC arrangement, No effect on *Abd-A* expression, No effect on *tin, svp* expression (**Embryo**)	Ponzielli et al., 2002 [96], Rosales-Vega et al., 2024 [94]
Ubx	**Null mutation**	Incomplete cardiac progenitor migration, No effect on the cellular dimorphism between ADv (aorta) (smaller volume) and PDv (heart chamber) (larger volume, rounded) CB, Minor defects in heart chamber morphology (**Embryo**)	Lovato et al., 2002 [95]
Ubx	**Overexpression**	No effect on cardiac cell specification (**Embryo**)	Lovato et al., 2002 [95]
Ubx	**Ectopic expression**	No effect on early heart chamber development with only late heart chamber differentiation impaired, Absence of functional ostia, No effect on *Abd-A* expression, No effect on *tin, svp* expression (**Embryo**)	Lo et al., 2002 [93]
Ubx	**Null mutation, Deficiency**	*Ubx*+ domains acquire ADv (aorta) phenotype, No effect in *Ubx*– domains (**Embryo**)	Perrin et al., 2004 [7], Ponzielli et al., 2002 [96]
Ubx	**Null mutation**	No effect on A5 myofibril arrangement, Histolysis in A6-A7 occurs normally (**Metamorphosis**)	Monier et al., 2005 [97]
Ubx	**Overexpression**	A1-A4 *tin*+ CB acquire A5 identity with longitudinal myofibril arrangement, No effect on *svp*+ CB (**Metamorphosis**)	Monier et al., 2005 [97]
Ubx	**Overexpression [**Mesoderm}	Expansion in *svp* expression (increase in *svp*+ clusters from 7 to 10) with associated expansion of *Wg* expression, Repression of *Antp* expression, Increase in total CB number (by ~ 16) (**Embryo**)	Ryan et al., 2005 [15]
Ubx	**Null mutation**	Absence of ADv AM formation (anterior 3 pairs) (**Embryo**)	LaBeau et al., 2009 [98]
Ubx	**Ectopic expression**	Heart tube elongation, Increase in AM pair number (9.75 ± 0.16 compared to 7 in WT) (**Embryo**)	LaBeau et al., 2009 [98]

Table S7. Genes involved in Histone modification during Drosophila melanogaster embryonic development: Mutations and Phenotypes.

Notes: ASD, Atrial septal defect; AoV, Aortic Valve; Ash1, Absent, small or homeotic disks 1; Ash2, Absent, small or homeotic disks 2; Bre, B recognition element 1; CB, Cardioblast; COMPASS, Complex of proteins associated with Set1; CoA, Coarctation of the Aorta; Dpy-30L1, Dpy-30 like 1; H2BK120, Histone 2B Lysine 120; H3K27, Histone 3 Lysine 27; H3K4, Histone 3 Lysine 4; Hcf, Host cell factor; KD, Knockdown; KMT2C, Lysine methyltransferase 2C; KMT2D, Lysine methyltransferase 2D; Lid, Drosophila ortholog of KDM5A/5B; Lpt, Lost PHDs of Trithorax-related (Trr); Mnn1, Menin 1; Prc, Pericardin; Ptip, PAX Transcription activation domain interacting protein; RNA, Ribonucleic acid interference; Rbbp5, Retinoblastoma binding protein 5; Scny, Scrawny; Set1, SET Containing domain 1; Set2, SET Containing domain 2; Smox, drosophila ortholog of SMAD2; TOF, Tetralogy of Fallot; Trr, Trithorax related; Trx, Trithorax; UbcD6, D. melanogaster ortholog of Ubiquitin conjugating enzyme (UBE); VSD, Ventricular septal defect; Wdr82, WD repeat domain 82; Wds, Will die slowly; me, methylation; ub, Ubiquitylation; μm, micrometers.

Gene/Mutation	Gene information	Effects on Cardiac Function	Effects on Methylation Status/Gene expression/Syndromes	Study (Reference)
Wds **KD [RNAi]**	Histone acetyltransferase, H3K4, Lipid homeostasis during development	Reduction in cardiac myofibril density (~ 40%), Increase in Prc content (~ 100%) (**Larva**), Reduction in cardiac myofibril density (~ 100%), Reduction in pericardin content (~ 100%), Reduction in CB number (~ 100%) **(Adult)**	[Effects on methylation status not evaluated in the study]	Zhu et al., 2017 [100], Zhao et al., 2023 [101]
		Increased lethality day 25 post eclosion (~ 100%)		
UbcD6 **KD [RNAi]**	Ubiquitin ligase E2, H2BK120ub	Reduction in cardiac myofibril density (~ 40%), Increase in Prc content (~ 50%) (**Larva**)	[Effects on methylation status not evaluated in the study]	Zhu et al., 2017 [100]
		Reduction in cardiac myofibril density (~ 80%), Increase in Prc content (~ 20%), Reduction in CB number (~ 20%) (**Adult**)		
		Increased lethality day 25 post eclosion (~ 50%)		
Kismet **KD [RNAi]**	Helicase, Chromatin remodeling factor, Segmentation	Reduction in cardiac myofibril density (~ 40%), Increase in Prc content (~ 50%) (**Larva**)	[Effects on methylation status not evaluated in the study]	Zhu et al., 2017 [100], Daubresse et al., 1999 [102]
		Reduction in cardiac myofibril density (~ 75%), Increase in Prc content (~ 20%), Reduction in CB number (~ 40%) (**Adult**)		
		Increased lethality day 25 post eclosion (~ 40%)		
Trx	Histone methyltransferase, H3K4me	Reduction in cardiac myofibril density (~ 60%), Increase in Prc content (~ 20%) (**Larva**)	[Effects on methylation status not evaluated in the study]	Zhu et al., 2017 [100]

Gene/Mutation	Gene information	Effects on Cardiac Function	Effects on Methylation Status/Gene expression/Syndromes	Study (Reference)
**KD [RNAi]**		Reduction in cardiac myofibril density (~ 100%), Reduction in Prc content (~ 80%), Reduction in CB number (~ 90%) (**Adult**)		
		Increased lethality day 25 post eclosion (~ 100%)		
Scny **KD [RNAi]**	Ubiquitin protease, H2BK120 deubiquitylation	No effect on cardiac myofibrillar density, No effect on Prc content (**Larva**)	[Effects on methylation status not evaluated in the study]	Zhu et al., 2017 [100]
		Reduction in cardiac myofibril density (~ 20%), No effect on Prc content, No effect on CB number (**Adult**)		
		Increased lethality day 25 post eclosion (~ 30%)		
Bre1 **KD [RNAi]**	Ubiquitin ligase E3, H2BK120ub (Required for H3K4me)	No effect on cardiac myofibrillar density, No effect on Prc content (**Larva**)	[Effects on methylation status not evaluated in the study]	Zhu et al., 2017 [100]
		Reduction in cardiac myofibril density (~ 30%), No effect on Prc content, No effect on CB number (**Adult**)		
		Increased lethality day 25 post eclosion (~ 20%)		
Smox **KD [RNAi]**	Chromatin binding, H3K27	No effect on cardiac myofibrillar density, No effect on Prc content (**Larva**)	[Effects on methylation status not evaluated in the study]	Zhu et al., 2017 [100]
		No effect on cardiac myofibril density, No effect on Prc content, No effect on CB number (**Adult**)		
		Increased lethality day 25 post eclosion (~ 30%)		
Lid	Histone demethylase, H3K4	No effect on cardiac myofibrillar density, No effect on Prc content (**Larva**)	[Effects on methylation status not evaluated in the study]	Zhu et al., 2017 [100]

Gene/Mutation	Gene information	Effects on Cardiac Function	Effects on Methylation Status/Gene expression/Syndromes	Study (Reference)
**KD [RNAi]**		No effect on cardiac myofibril density, No effect on Prc content, No effect on CB number (**Adult**)		
		Increased lethality day 25 post eclosion (~ 20%)		
Lpt **KD [RNAi]**	Histone methyltransferase, H3K4me	'**Broken-hearted**' phenotype (~ 70% of embryos), Reduction in CB number (*Lpt* by ~ 30%) (**Embryo**)	Reduction in both H3K4me1 and H3K4me2 by ~ 75% (*Lpt, Lpt/Trr*)	Huang et al., 2022 [103]
		Disruption in actin filament arrangement, Reduction in cardiac myofibril density (combined *Lpt/Trr* by ~ 20%), Increase in Prc content (*Lpt, Lpt/Trr* by ~ 40%), Reduction in CB number (*Lpt/Trr* by ~ 40%) (**Adult**)		
		Reduction in diastolic diameter (*Lpt* by ~ 27 µm), Reduction in heart rate (*Lpt, Lpt/Trr* by ~ 4 beats/sec) (**Adult**)		
		Increased lethality at late embryo/early larva stages (> 95%); Increased lethality at eclosion (16% for *Lpt,* 16% for *Lpt/Trr*), Reduced survival (total lifespan reduced to ~ 30 days compared to 50 days for control)		
Trr **KD [RNAi]**	Trr-COMPASS Unique Core subunit, Histone methyltransferase, H3K4	'Broken heart' phenotype (~ 70% of embryos), Reduction in CB number (*Trr* by ~ 20%) (**Embryo**)	Reduction in both H3K4me1 and H3K4me2 by ~ 75% (*Lpt, Lpt/Trr*)	Huang et al., 2022 [103], Koemans et al., 2017 [104]
		Disruption in actin filament arrangement, Reduction in cardiac myofibril density (most severe with *Trr* by ~ 50%), Increase in Prc content (*Trr* by ~ 40%), Reduction in CB number (*Trr* by ~ 40%) (**Adult**)		
		Reduction in heart rate (*Trr* by ~ 4 beats/sec) (**Adult**)		
		Increased lethality at late embryo/early larva stages (> 95%); Increased lethality at eclosion (14% for *Trr*), Reduced survival (total lifespan reduced to ~ 30 days compared to ~ 50 days for control)		

Gene/Mutation	Gene information	Effects on Cardiac Function	Effects on Methylation Status/Gene expression/Syndromes	Study (Reference)
Hcf<br><br>**KD [RNAi]**	Set1, Trr-COMPASS Common subunit, Histone methyltransferase, H3K4	Disruption in actin filament arrangement, No effect on CB number (**Adult**)	Reduction in H3K4me1 by ~ 40%	Huang et al., 2022 [103]
		Reduction in diastolic diameter (*Hcf* by ~ 27 μm), Reduction in heart rate (*Hcf* by ~ 2 beats/sec) (**Adult**)		
		Increased lethality at eclosion (17% for *Hcf*), Reduced survival (total lifespan reduced to ~ 30 days compared to ~ 50 days for control)		
Ash1<br><br>**KD [RNAi]**	Histone methyltransferase, H3K36, Steady expression throughout development	Disruption in actin filament arrangement, Reduction in cardiac myofibril density (most severe with *ash1,* by ~ 0.4), Increase in Prc content (**Adult**)	Reduction in H3K36me2 by ~ 0.5 (*Ash1-R1288A*), No effects in H3K36me1	J. Zhu et al., 2023a [105]
		No effect on diastolic diameter, Increase in systolic diameter by 40 μm (*Ash1*), Increase in heart period (most severe with *ash1,* by ~ 0.3 sec) (**Adult**)		
		Increased lethality at eclosion (39% for *Ash1*), Reduced survival (total lifespan reduced to 20 days [*ash1*] compared to 60 days for control) (**Adult**)		
Set2<br><br>**KD [RNAi]**	Histone methyltransferase, H3K36, Steady expression throughout development	Disruption in actin filament arrangement, Reduction in cardiac myofibril density, Increase in Prc content (**Adult**)	[Effects on methylation status not evaluated in the study]	J. Zhu et al., 2023a [105]
		No effect on diastolic diameter, No effect on systolic diameter, Increase in heart period (by ~ 0.2 sec) (**Adult**)		
		Increased lethality at eclosion (26% for *Set2*), Reduced survival (total lifespan reduced to ~ 35 days [*Set2*] compared to 60 days for control) (**Adult**)		
Set1	Set1-COMPASS Unique Core subunit, Histone methyltransferase, H3K4me,	Increase in cardiac cell number /hemisegment by ~ 1 (**Larva**), Disruption in actin filament arrangement, Reduction in cardiac myofibril density,		

Gene/Mutation	Gene information	Effects on Cardiac Function	Effects on Methylation Status/Gene expression/Syndromes	Study (Reference)
**KD [RNAi]**	Steady expression throughout development	Increase in Prc content (by ~ 50%), Reduction in cardiac cell number **(Adult)**	Reduction in H3K4me2 by ~ 80% (*Set1*)	J.-Y. Zhu et al., 2023 [106]
		No effect on diastolic diameter, No effect on systolic diameter, Increase in heart period (by ~ 0.1 sec) (**Adult**)		
		Increased lethality at eclosion (26% for *Set1*), Reduced survival		
		Upregulation of carbohydrate metabolism genes, Downregulation of lipid metabolism genes (*Set1*)		
Trx **KD [RNAi]**	Trx-COMPASS Unique Core subunit, Histone methyltransferase, H3K4, Steady expression throughout development	Increase in cardiac cell number/hemisegment by ~ 1 (**Larva**), Disruption in actin filament arrangement, Reduction in cardiac myofibril density, Increase in Prc content (by ~ 50%), Reduction in cardiac cell number **(Adult)**	Reduction in H3K4me1 by ~ 100% (*Trx*)	J.-Y. Zhu et al., 2023 [106]
		No effect on diastolic diameter, No effect on systolic diameter, Increase in heart period (by ~ 0.1 sec) (**Adult**)		
		Increased lethality at eclosion (17% for *Trx*), Reduced survival		
		Downregulation of muscle development genes (*Trx, Trr*), Upregulation of ion transport genes (*Trx*)		
Trr **KD [RNAi]**	Trr-COMPASS Unique Core subunit, Histone methyltransferase, H3K4, Drop in expression by ~ 40% (Stage 16)	Increase in cardiac cell number/hemisegment by ~ 1 (**Larva**), Disruption in actin filament arrangement, Reduction in cardiac myofibril density (most severe with *Trr,* by ~ 50%), Increase in Prc content (by ~ 50%), Reduction in cardiac cells (most severe with *Trr,* by ~ 20%) **(Adult)**	Reduction in both H3K4me1 and H3K4me2, by ~ 100%, ~ 50% respectively (*Trr*)	J.-Y. Zhu et al., 2023 [106]
		Reduction in diastolic diameter by ~ 10% (*Trr*), Increase in systolic diameter by ~ 12 µm (*Trr*), Increase in heart period (most severe with *Trr,* by ~ 0.2 sec) (**Adult**)		

Gene/Mutation	Gene information	Effects on Cardiac Function	Effects on Methylation Status/Gene expression/Syndromes	Study (Reference)
		Increased lethality at eclosion (*32%* for *Trr*), Reduced survival		
		Downregulation of muscle development genes (*Trx, Trr*), Downregulation of ion transport genes (*Trr*)		
Ash2 KD [RNAi]	Set1, Trx, Trr-COMPASS Common subunit, Histone methyltransferase, H3K4	Disruption in actin filament arrangement, Reduction in cardiac myofibril density (most severe with *Dpy-30L1,* by ~ 80%), Increase in Prc content (Type IV-like Collagen) (most severe with *Dpy-30L1, Ash2* by ~ 50%), Reduction in CB number (**Adult**)	[Effects on methylation status not evaluated in the study]	Zhu et al., 2024 [107]
		No effect on diastolic diameter, Increase in systolic diameter, Increase in heart period (most severe with *Dpy-30L1,* by ~ 0.3 sec) (**Adult**)		
		Increased lethality, Reduced survival (total lifespan reduced to ~ 20 days [*Ash2*] compared to > 30 days for control)		
Dpy-30L1 KD [RNAi]	Set1, Trx, Trr-COMPASS Common subunit, Histone methyltransferase, H3K4	Disruption in actin filament arrangement, Reduction in cardiac myofibril density (most severe with *Dpy-30L1,* by ~ 80%), Increase in Prc content (Type IV-like Collagen) (most severe with *Dpy-30L1, Ash2* by ~ 50%), Reduction in CB number (most severe with *Dpy-30L1,* by ~ 50%) (**Adult**)	[Effects on methylation status not evaluated in the study]	Zhu et al., 2024 [107]
		No effect on diastolic diameter, Increase in systolic diameter (most severe with *Dpy-30L1,* by ~ 60 µm), Increase in heart period (most severe with *Dpy-30L1,* by ~ 0.3 sec) (**Adult**)		
		Increased lethality, Reduced survival (total lifespan reduced to 10 days [*Dpy-30L1*] compared to > 30 days for control)		
Rbbp5 KD [RNAi]	Set1, Trx, Trr-COMPASS Common subunit, Histone methyltransferase, H3K4	Disruption in actin filament arrangement, Reduction in cardiac myofibril density (most severe with *Dpy-30L1,* by ~ 80%), Increase in Prc content (Type IV-like Collagen), Reduction in CB number (**Adult**)	[Effects on methylation status not evaluated in the study]	Zhu et al., 2024 [107]

Gene/Mutation	Gene information	Effects on Cardiac Function	Effects on Methylation Status/Gene expression/Syndromes	Study (Reference)
		No effect on diastolic diameter, Increase in systolic diameter, Increase in heart period (**Adult**)		
		Increased lethality, Reduced survival (total lifespan reduced to ~ 30 days [*Rbbp5*] compared to > 30 days for control)		
Wdr82 **KD [RNAi]**	Set1-COMPASS Unique subunit, Histone methyltransferase, H3K4, Active during Stages 13-14	Disruption in actin filament arrangement, Reduction in cardiac myofibril density (most severe with *Wdr82,* by ~ 50%), Increase in Prc content (Type IV-like Collagen) accumulation (most severe with *Wdr82, Mnn1,* by ~ 50%), Reduction in CB number (**Adult**)	Reduction in H3K4me2 by ~ 1.1%	Zhu et al., 2024 [107]
		Reduction in diastolic diameter (most severe with *Wdr82,* by ~ 20 µm), Increase in systolic diameter (most severe with *Wdr82, Mnn1* by ~ 40 µm) (**Adult**)		
		Increased lethality at eclosion (most severe with *Wdr82,* lethality rate of 58%)		
Mnn1 **KD [RNAi]**	Trx-COMPASS Unique subunit, Histone methyltransferase, H3K4, Active during Stages 16-17	Disruption in actin filament arrangement, Reduction in cardiac myofibril density, Increase in Prc content (Type IV-like Collagen) (most severe with *Wdr82, Mnn1,* by ~ 50%), Reduction in CB number (most severe with *Mnn1,* by ~ 40%) (**Adult**)	Reduction in H3K4me1 by ~ 1.1%	Zhu et al., 2024 [107]
		Reduction in diastolic diameter, Increase in systolic diameter (most severe with *Wdr82, Mnn1* by ~ 40 µm) (**Adult**)		
		Increased lethality at eclosion		
Ptip **KD [RNAi]**	Trr-COMPASS Unique subunit, Histone methyltransferase, H3K4, Active during Stages 13-14 with Drop in expression during Stage 14E (~ 0.5)	Disruption in actin filament arrangement, Reduction in cardiac myofibril density, Increase in Prc content (Type IV-like Collagen), Reduction in CB number (**Adult**)	Reduction in both H3K4me1 and H3K4me2 by ~ 0.8%	Zhu et al., 2024 [107]
		Reduction in diastolic diameter, Increase in systolic diameter (**Adult**)		

Gene/Mutation	Gene information	Effects on Cardiac Function	Effects on Methylation Status/Gene expression/Syndromes	Study (Reference)
		Increased lethality at eclosion		

References

1. Huang, X.; Fu, Y.; Lee, H.; Zhao, Y.; Yang, W.; van de Leemput, J.; Han, Z. Single-Cell Profiling of the Developing Embryonic Heart in Drosophila. *Development* **2023**, *150*, dev201936, doi:10.1242/dev.201936.
2. Lo, P.C.H.; Frasch, M. A Role for the COUP-TF-Related Gene *Seven-up* in the Diversification of Cardioblast Identities in the Dorsal Vessel of *Drosophila*. *Mechanisms of Development* **2001**, *104*, 49–60, doi:10.1016/S0925-4773(01)00361-6.
3. Zmojdzian, M.; Joussineau, S. de; Ponte, J.P.D.; Jagla, K. Distinct Subsets of Eve-Positive Pericardial Cells Stabilise Cardiac Outflow and Contribute to Hox Gene-Triggered Heart Morphogenesis in Drosophila. *Development (Cambridge, England)* **2018**, *145*, dev158717, doi:10.1242/dev.158717.
4. Dondi, C.; Bertin, B.; Da Ponte, J.-P.; Wojtowicz, I.; Jagla, K.; Junion, G. A Polarized Nucleus-Cytoskeleton-ECM Connection in Migrating Cardioblasts Controls Heart Tube Formation in Drosophila. *Development* **2021**, *148*, dev192146, doi:10.1242/dev.192146.
5. Asadzadeh, J.; Neligan, N.; Kramer, S.G.; Labrador, J.-P. Tinman Regulates NetrinB in the Cardioblasts of the Drosophila Dorsal Vessel. *PLOS ONE* **2016**, *11*, e0148526, doi:10.1371/journal.pone.0148526.
6. Schwarz, B.; Hollfelder, D.; Scharf, K.; Hartmann, L.; Reim, I. Diversification of Heart Progenitor Cells by EGF Signaling and Differential Modulation of ETS Protein Activity. *eLife* **2018**, *7*, e32847, doi:10.7554/eLife.32847.
7. Perrin, L.; Monier, B.; Ponzielli, R.; Astier, M.; Semeriva, M. *Drosophila* Cardiac Tube Organogenesis Requires Multiple Phases of Hox Activity. *Developmental Biology* **2004**, *272*, 419–431, doi:10.1016/j.ydbio.2004.04.036.
8. Raza, Q.; Jacobs, J.R. Guidance Signalling Regulates Leading Edge Behaviour during Collective Cell Migration of Cardiac Cells in *Drosophila*. *Developmental Biology* **2016**, *419*, 285–297, doi:10.1016/j.ydbio.2016.09.005.
9. Reim, I.; Mohler, J.P.; Frasch, M. *Tbx20*-Related Genes, *Mid* and *H15*, Are Required for *Tinman* Expression, Proper Patterning, and Normal Differentiation of Cardioblasts in *Drosophila*. *Mechanisms of Development* **2005**, *122*, 1056–1069, doi:10.1016/j.mod.2005.04.006.
10. Li, H.; Janssens, J.; De Waegeneer, M.; Kolluru, S.S.; Davie, K.; Gardeux, V.; Saelens, W.; David, F.P.A.; Brbić, M.; Spanier, K.; et al. Fly Cell Atlas: A Single-Nucleus Transcriptomic Atlas of the Adult Fruit Fly. *Science* **2022**, *375*, eabk2432, doi:10.1126/science.abk2432.
11. Reim, I.; Frasch, M. Genetic and Genomic Dissection of Cardiogenesis in the Drosophila Model. *Pediatric cardiology* **2010**, *31*, 325–334, doi:10.1007/s00246-009-9612-1.
12. Ahmad, S.M. Conserved Signaling Mechanisms in Drosophila Heart Development. *Developmental Dynamics* **2017**, *246*, 641–656, doi:10.1002/dvdy.24530.
13. Molina, M.R.; Cripps, R.M. Ostia, the Inflow Tracts of the *Drosophila* Heart, Develop from a Genetically Distinct Subset of Cardial Cells. *Mechanisms of Development* **2001**, *109*, 51–59, doi:10.1016/S0925-4773(01)00509-3.
14. Lammers, K.; Abeln, B.; Hüsken, M.; Lehmacher, C.; Psathaki, O.E.; Alcorta, E.; Meyer, H.; Paululat, A. Formation and Function of Intracardiac Valve Cells in the Drosophila Heart. *Journal of Experimental Biology* **2017**, *220*, 1852–1863, doi:10.1242/jeb.156265.
15. Ryan, K.M.; Hoshizaki, D.K.; Cripps, R.M. Homeotic Selector Genes Control the Patterning of *Seven-up* Expressing Cells in the *Drosophila* Dorsal Vessel. *Mechanisms of Development* **2005**, *122*, 1023–1033, doi:10.1016/j.mod.2005.04.007.
16. Bodmer, R.; Frasch, M. Development and Aging of the *Drosophila* Heart. In *Heart Development and Regeneration*; Rosenthal, N., Harvey, R.P., Eds.; Academic Press: Boston, 2010; pp. 47–86 ISBN 978-0-12-381332-9.
17. Mann, T.; Bodmer, R.; Pandur, P. The Drosophila Homolog of Vertebrate Islet1 Is a Key Component in Early Cardiogenesis. *Development* **2009**, *136*, 317–326, doi:10.1242/dev.022533.
18. Meyer, C.; Drechsler, M.; Meyer, H.; Paululat, A. Differentiation and Function of Cardiac Valves in the Adult Drosophila Heart. *Journal of Experimental Biology* **2023**, *226*, jeb245839, doi:10.1242/jeb.245839.
19. Bodmer, R.; Jan, L.Y.; Jan, Y.N. A New Homeobox-Containing Gene, Msh-2, Is Transiently Expressed Early during Mesoderm Formation of Drosophila. *Development* **1990**, *110*, 661–669, doi:10.1242/dev.110.3.661.
20. Hu, Y.; Flockhart, I.; Vinayagam, A.; Bergwitz, C.; Berger, B.; Perrimon, N.; Mohr, S.E. An Integrative Approach to Ortholog Prediction for Disease-Focused and Other Functional Studies. *BMC Bioinformatics* **2011**, *12*, 357, doi:10.1186/1471-2105-12-357.
21. Bodmer, R. The Gene Tinman Is Required for Specification of the Heart and Visceral Muscles in Drosophila. *Development* **1993**, *118*, 719–729, doi:10.1242/dev.118.3.719.
22. Reim, I.; Frasch, M. The Dorsocross T-Box Genes Are Key Components of the Regulatory Network Controlling Early Cardiogenesis in Drosophila. *Development* **2005**, *132*, 4911–4925, doi:10.1242/dev.02077.

23. Lovato, T.L.; Blotz, B.; Bileckyj, C.; Johnston, C.A.; Cripps, R.M. Modeling a Variant of Unknown Significance in the Drosophila Ortholog of the Human Cardiogenic Gene NKX2.5. *DMM Disease Models and Mechanisms* **2023**, *16*, doi:10.1242/dmm.050059.
24. Sloutskin, A.; Itzhak, D.; Vogler, G.; Pozeilov, H.; Ideses, D.; Alter, H.; Adato, O.; Shachar, H.; Doniger, T.; Shohat-Ophir, G.; et al. From Promoter Motif to Cardiac Function: A Single DPE Motif Affects Transcription Regulation and Organ Function in Vivo. *Development* **2024**, *151*, dev202355, doi:10.1242/dev.202355.
25. Lilly, B.; Zhao, B.; Ranganayakulu, G.; Paterson, B.M.; Schulz, R.A.; Olson, E.N. Requirement of MADS Domain Transcription Factor D-MEF2 for Muscle Formation in Drosophila. *Science* **1995**, *267*, 688–693, doi:10.1126/science.7839146.
26. Ryan, K.M.; Hendren, J.D.; Helander, L.A.; Cripps, R.M. The NK Homeodomain Transcription Factor Tinman Is a Direct Activator of *Seven-up* in the *Drosophila* Dorsal Vessel. *Developmental Biology* **2007**, *302*, 694–702, doi:10.1016/j.ydbio.2006.10.025.
27. Qian, L.; Liu, J.; Bodmer, R. *Neuromancer* Tbx20-Related Genes (*H15/Midline*) Promote Cell Fate Specification and Morphogenesis of the *Drosophila* Heart. *Developmental Biology* **2005**, *279*, 509–524, doi:10.1016/j.ydbio.2005.01.013.
28. Qian, L.; Mohapatra, B.; Akasaka, T.; Liu, J.; Ocorr, K.; Towbin, J.A.; Bodmer, R. Transcription Factor Neuromancer/TBX20 Is Required for Cardiac Function in Drosophila with Implications for Human Heart Disease. *Proceedings of the National Academy of Sciences* **2008**, *105*, 19833–19838, doi:10.1073/pnas.0808705105.
29. Han, Z.; Olson, E.N. Hand Is a Direct Target of Tinman and GATA Factors during Drosophila Cardiogenesis and Hematopoiesis. *Development* **2005**, *132*, 3525–3536, doi:10.1242/dev.01899.
30. Han, Z.; Yi, P.; Li, X.; Olson, E.N. Hand, an Evolutionarily Conserved bHLH Transcription Factor Required for Drosophila Cardiogenesis and Hematopoiesis. *Development* **2006**, *133*, 1175–1182, doi:10.1242/dev.02285.
31. Lo, P.C.H.; Zaffran, S.; Sénatore, S.; Frasch, M. The Drosophila Hand Gene Is Required for Remodeling of the Developing Adult Heart and Midgut during Metamorphosis. *Dev Biol* **2007**, *311*, 287–296, doi:10.1016/j.ydbio.2007.08.024.
32. Fujioka, M.; Wessells, R.J.; Han, Z.; Liu, J.; Fitzgerald, K.; Yusibova, G.L.; Zamora, M.; Ruiz-Lozano, P.; Bodmer, R.; Jaynes, J.B. Embryonic Even Skipped–Dependent Muscle and Heart Cell Fates Are Required for Normal Adult Activity, Heart Function, and Lifespan. *Circulation Research* **2005**, *97*, 1108–1114, doi:10.1161/01.RES.0000191546.08532.B2.
33. Tao, Y.; Wang, J.; Tokusumi, T.; Gajewski, K.; Schulz, R.A. Requirement of the LIM Homeodomain Transcription Factor Tailup for Normal Heart and Hematopoietic Organ Formation in Drosophila Melanogaster. *Mol Cell Biol* **2007**, *27*, 3962–3969, doi:10.1128/MCB.00093-07.
34. Meyer, C.; Bataillé, L.; Drechsler, M.; Paululat, A. Tailup Expression in Larval and Adult Cardiac Valve Cells. *genesis* **2023**, *61*, e23506, doi:10.1002/dvg.23506.
35. Junion, G.; Bataillé, L.; Jagla, T.; Ponte, J.P.D.; Tapin, R.; Jagla, K. Genome-Wide View of Cell Fate Specification: Ladybird Acts at Multiple Levels during Diversification of Muscle and Heart Precursors. *Genes Dev.* **2007**, *21*, 3163–3180, doi:10.1101/gad.437307.
36. Yi, P.; Han, Z.; Li, X.; Olson, E.N. The Mevalonate Pathway Controls Heart Formation in Drosophila by Isoprenylation of Gγ1. *Science* **2006**, *313*, 1301–1303, doi:10.1126/science.1127704.
37. Schaub, C.; Nagaso, H.; Jin, H.; Frasch, M. Org-1, the Drosophila Ortholog of Tbx1, Is a Direct Activator of Known Identity Genes during Muscle Specification. *Development* **2012**, *139*, 1001–1012, doi:10.1242/dev.073890.
38. Boukhatmi, H.; Schaub, C.; Bataillé, L.; Reim, I.; Frendo, J.-L.; Frasch, M.; Vincent, A. An Org-1–Tup Transcriptional Cascade Reveals Different Types of Alary Muscles Connecting Internal Organs in Drosophila. *Development* **2014**, *141*, 3761–3771, doi:10.1242/dev.111005.
39. Schaub, C.; März, J.; Reim, I.; Frasch, M. Org-1-Dependent Lineage Reprogramming Generates the Ventral Longitudinal Musculature of the Drosophila Heart. *Current Biology* **2015**, *25*, 488–494, doi:10.1016/j.cub.2014.12.029.
40. Basu, M.; Zhu, J.-Y.; LaHaye, S.; Majumdar, U.; Jiao, K.; Han, Z.; Garg, V. Epigenetic Mechanisms Underlying Maternal Diabetes-Associated Risk of Congenital Heart Disease. *JCI Insight* **2017**, *2*, e95085, doi:10.1172/jci.insight.95085.
41. Liu, N.; Schoch, K.; Luo, X.; Pena, L.D.M.; Bhavana, V.H.; Kukolich, M.K.; Stringer, S.; Powis, Z.; Radtke, K.; Mroske, C.; et al. Functional Variants in TBX2 Are Associated with a Syndromic Cardiovascular and Skeletal Developmental Disorder. *Human Molecular Genetics* **2018**, *27*, 2454–2465, doi:10.1093/hmg/ddy146.
42. Schroeder, A.M.; Allahyari, M.; Vogler, G.; Missinato, M.A.; Nielsen, T.; Yu, M.S.; Theis, J.L.; Larsen, L.A.; Goyal, P.; Rosenfeld, J.A.; et al. Model System Identification of Novel Congenital Heart Disease Gene Candidates: Focus on RPL13. *Human Molecular Genetics* **2019**, *28*, 3954–3969, doi:10.1093/hmg/ddz213.

43. Pareek, G.; Thomas, R.E.; Pallanck, L.J. Loss of the Drosophila M-AAA Mitochondrial Protease Paraplegin Results in Mitochondrial Dysfunction, Shortened Lifespan, and Neuronal and Muscular Degeneration. *Cell Death Dis* **2018**, *9*, 1–14, doi:10.1038/s41419-018-0365-8.
44. Wang, W.; Bouhours, M.; Gracheva, E.O.; Liao, E.H.; Xu, K.; Sengar, A.S.; Xin, X.; Roder, J.; Boone, C.; Richmond, J.E.; et al. ITSN-1 Controls Vesicle Recycling at the Neuromuscular Junction and Functions in Parallel with DAB-1. *Traffic (Copenhagen, Denmark)* **2008**, *9*, 742, doi:10.1111/j.1600-0854.2008.00712.x.
45. He, L.; Wu, B.; Shi, J.; Du, J.; Zhao, Z. Regulation of Feeding and Energy Homeostasis by Clock-Mediated *Gart* in *Drosophila*. *Cell Reports* **2023**, *42*, 112912, doi:10.1016/j.celrep.2023.112912.
46. Tao, Y.; Zhang, Q.; Wang, H.; Yang, X.; Mu, H. Alternative Splicing and Related RNA Binding Proteins in Human Health and Disease. *Sig Transduct Target Ther* **2024**, *9*, 1–33, doi:10.1038/s41392-024-01734-2.
47. Bandura, J.L.; Beall, E.L.; Bell, M.; Silver, H.R.; Botchan, M.R.; Calvi, B.R. Humpty Dumpty Is Required for Developmental DNA Amplification and Cell Proliferation in Drosophila. *Current Biology* **2005**, *15*, 755–759, doi:10.1016/j.cub.2005.02.063.
48. Grant, J.; Saldanha, J.W.; Gould, A.P. A Drosophila Model for Primary Coenzyme Q Deficiency and Dietary Rescue in the Developing Nervous System. *Dis Model Mech* **2010**, *3*, 799–806, doi:10.1242/dmm.005579.
49. Theis, J.L.; Vogler, G.; Missinato, M.A.; Li, X.; Nielsen, T.; Zeng, X.-X.I.; Martinez-Fernandez, A.; Walls, S.M.; Kervadec, A.; Kezos, J.N.; et al. Patient-Specific Genomics and Cross-Species Functional Analysis Implicate LRP2 in Hypoplastic Left Heart Syndrome. *eLife* **2020**, *9*, e59554, doi:10.7554/eLife.59554.
50. Riedel, F.; Vorkel, D.; Eaton, S. Megalin-Dependent Yellow Endocytosis Restricts Melanization in the Drosophila Cuticle. *Development* **2011**, *138*, 149–158, doi:10.1242/dev.056309.
51. Rodríguez-Vázquez, M.; Vaquero, D.; Parra-Peralbo, E.; Mejía-Morales, J.E.; Culi, J. Drosophila Lipophorin Receptors Recruit the Lipoprotein LTP to the Plasma Membrane to Mediate Lipid Uptake. *PLOS Genetics* **2015**, *11*, e1005356, doi:10.1371/journal.pgen.1005356.
52. Ding, M.; Zheng, L.; Li, Q.F.; Wang, W.L.; Peng, W.D.; Zhou, M. Exercise-Training Regulates Apolipoprotein B in Drosophila to Improve HFD-Mediated Cardiac Function Damage and Low Exercise Capacity. *Front. Physiol.* **2021**, *12*, doi:10.3389/fphys.2021.650959.
53. Auxerre-Plantié, E.; Nielsen, T.; Grunert, M.; Olejniczak, O.; Perrot, A.; Özcelik, C.; Harries, D.; Matinmehr, F.; Dos Remedios, C.; Mühlfeld, C.; et al. Identification of MYOM2 as a Candidate Gene in Hypertrophic Cardiomyopathy and Tetralogy of Fallot, and Its Functional Evaluation in the Drosophila Heart. *Dis Model Mech* **2020**, *13*, dmm045377, doi:10.1242/dmm.045377.
54. Akasaka, T.; Ocorr, K.; Lin, L.; Vogler, G.; Bodmer, R.; Grossfeld, P. Overexpression of Kif1A in the Developing Drosophila Heart Causes Valvar and Contractility Defects: Implications for Human Congenital Heart Disease. *Journal of Cardiovascular Development and Disease* **2020**, *7*, 22, doi:10.3390/jcdd7020022.
55. Nim, H.T.; Dang, L.; Thiyagarajah, H.; Bakopoulos, D.; See, M.; Charitakis, N.; Sibbritt, T.; Eichenlaub, M.P.; Archer, S.K.; Fossat, N.; et al. A Cis-Regulatory-Directed Pipeline for the Identification of Genes Involved in Cardiac Development and Disease. *Genome Biol* **2021**, *22*, 335, doi:10.1186/s13059-021-02539-0.
56. Lai, K.; Amsterdam, A.; Farrington, S.; Bronson, R.T.; Hopkins, N.; Lees, J.A. Many Ribosomal Protein Mutations Are Associated with Growth Impairment and Tumor Predisposition in Zebrafish. *Developmental Dynamics* **2009**, *238*, 76–85, doi:10.1002/dvdy.21815.
57. Birker, K.; Ge, S.; Kirkland, N.J.; Theis, J.L.; Marchant, J.; Fogarty, Z.C.; Missinato, M.A.; Kalvakuri, S.; Grossfeld, P.; Engler, A.J.; et al. Mitochondrial MICOS Complex Genes, Implicated in Hypoplastic Left Heart Syndrome, Maintain Cardiac Contractility and Actomyosin Integrity. *eLife* **2023**, *12*, e83385, doi:10.7554/eLife.83385.
58. Stark, K.A.; Yee, G.H.; Roote, C.E.; Williams, E.L.; Zusman, S.; Hynes, R.O. A Novel α Integrin Subunit Associates with β PS and Functions in Tissue Morphogenesis and Movement during Drosophila Development. *Development* **1997**, *124*, 4583–4594, doi:10.1242/dev.124.22.4583.
59. Su, M.-T.; Venkatesh, T.V.; Wu, X.; Golden, K.; Bodmer, R. The Pioneer Gene, *Apontic*, Is Required for Morphogenesis and Function of the *Drosophila* Heart. *Mechanisms of Development* **1999**, *80*, 125–132, doi:10.1016/S0925-4773(98)00197-X.
60. Qian, L.; Liu, J.; Bodmer, R. Slit and Robo Control Cardiac Cell Polarity and Morphogenesis. *Current Biology* **2005**, *15*, 2271–2278, doi:10.1016/j.cub.2005.10.037.
61. MacMullin, A.; Jacobs, J.R. Slit Coordinates Cardiac Morphogenesis in *Drosophila*. *Developmental Biology* **2006**, *293*, 154–164, doi:10.1016/j.ydbio.2006.01.027.
62. Albrecht, S.; Wang, S.; Holz, A.; Bergter, A.; Paululat, A. The ADAM Metalloprotease Kuzbanian Is Crucial for Proper Heart Formation in *Drosophila Melanogaster*. *Mechanisms of Development* **2006**, *123*, 372–387, doi:10.1016/j.mod.2006.03.005.

63. Medioni, C.; Astier, M.; Zmojdzian, M.; Jagla, K.; Sémériva, M. Genetic Control of Cell Morphogenesis during Drosophila Melanogaster Cardiac Tube Formation. *J Cell Biol* **2008**, *182*, 249–261, doi:10.1083/jcb.200801100.

64. Santiago-Martínez, E.; Soplop, N.H.; Patel, R.; Kramer, S.G. Repulsion by Slit and Roundabout Prevents Shotgun/E-Cadherin-Mediated Cell Adhesion during Drosophila Heart Tube Lumen Formation. *J Cell Biol* **2008**, *182*, 241–248, doi:10.1083/jcb.200804120.

65. Zmojdzian, M.; Da Ponte, J.P.; Jagla, K. Cellular Components and Signals Required for the Cardiac Outflow Tract Assembly in Drosophila. *Proceedings of the National Academy of Sciences* **2008**, *105*, 2475–2480, doi:10.1073/pnas.0706402105.

66. Qian, L.; Wythe, J.D.; Liu, J.; Cartry, J.; Vogler, G.; Mohapatra, B.; Otway, R.T.; Huang, Y.; King, I.N.; Maillet, M.; et al. Tinman/Nkx2-5 Acts via miR-1 and Upstream of Cdc42 to Regulate Heart Function across Species. *J Cell Biol* **2011**, *193*, 1181–1196, doi:10.1083/jcb.201006114.

67. Grossman, T.R.; Gamliel, A.; Wessells, R.J.; Taghli-Lamallem, O.; Jepsen, K.; Ocorr, K.; Korenberg, J.R.; Peterson, K.L.; Rosenfeld, M.G.; Bodmer, R.; et al. Over-Expression of DSCAM and COL6A2 Cooperatively Generates Congenital Heart Defects. *PLOS Genetics* **2011**, *7*, e1002344, doi:10.1371/journal.pgen.1002344.

68. Vanderploeg, J.; Vazquez Paz, L.L.; MacMullin, A.; Jacobs, J.R. Integrins Are Required for Cardioblast Polarisation in Drosophila. *BMC Developmental Biology* **2012**, *12*, 8, doi:10.1186/1471-213X-12-8.

69. Vogler, G.; Liu, J.; Iafe, T.W.; Migh, E.; Mihály, J.; Bodmer, R. Cdc42 and Formin Activity Control Non-Muscle Myosin Dynamics during Drosophila Heart Morphogenesis. *Journal of Cell Biology* **2014**, *206*, 909–922, doi:10.1083/jcb.201405075.

70. Haack, T.; Schneider, M.; Schwendele, B.; Renault, A.D. *Drosophila* Heart Cell Movement to the Midline Occurs through Both Cell Autonomous Migration and Dorsal Closure. *Developmental Biology* **2014**, *396*, 169–182, doi:10.1016/j.ydbio.2014.08.033.

71. Patel, M.V.; Zhu, J.; Jiang, Z.; Richman, A.; VanBerkum, M.F.A.; Han, Z. Gia/Mthl5 Is an Aorta Specific GPCR Required for *Drosophila* Heart Tube Morphology and Normal Pericardial Cell Positioning. *Developmental Biology* **2016**, *414*, 100–107, doi:10.1016/j.ydbio.2016.03.009.

72. Raza, Q.S.; Vanderploeg, J.L.; Jacobs, J.R. Matrix Metalloproteinases Are Required for Membrane Motility and Lumenogenesis during Drosophila Heart Development. *PLOS ONE* **2017**, *12*, e0171905, doi:10.1371/journal.pone.0171905.

73. Vanderploeg, J.; Jacobs, J.R. Mapping Heart Development in Flies: Src42A Acts Non-Autonomously to Promote Heart Tube Formation in Drosophila. *Vet Sci* **2017**, *4*, 23, doi:10.3390/vetsci4020023.

74. Hughes, C.J.R.; Turner, S.; Andrews, R.M.; Vitkin, A.; Jacobs, J.R. Matrix Metalloproteinases Regulate ECM Accumulation but Not Larval Heart Growth in *Drosophila Melanogaster*. *Journal of Molecular and Cellular Cardiology* **2020**, *140*, 42–55, doi:10.1016/j.yjmcc.2020.02.008.

75. Wu, X.; Golden, K.; Bodmer, R. Heart Development in *Drosophila* Requires the Segment Polarity Gene *Wingless*. *Developmental Biology* **1995**, *169*, 619–628, doi:10.1006/dbio.1995.1174.

76. Park, M.; Wu, X.; Golden, K.; Axelrod, J.D.; Bodmer, R. The Wingless Signaling Pathway Is Directly Involved in*Drosophila*Heart Development. *Developmental Biology* **1996**, *177*, 104–116, doi:10.1006/dbio.1996.0149.

77. Yin, Z.; Frasch, M. Regulation and Function of Tinman during Dorsal Mesoderm Induction and Heart Specification in Drosophila. *Developmental Genetics* **1998**, *22*, 187–200, doi:10.1002/(SICI)1520-6408(1998)22:3%3C187::AID-DVG2%3E3.0.CO;2-2.

78. Bhanot, P.; Fish, M.; Jemison, J.A.; Nusse, R.; Nathans, J.; Cadigan, K.M. Frizzled and DFrizzled-2 Function as Redundant Receptors for Wingless during Drosophila Embryonic Development. *Development* **1999**, *126*, 4175–4186, doi:10.1242/dev.126.18.4175.

79. Chen, C.; Struhl, G. Wingless Transduction by the Frizzled and Frizzled2 Proteins of Drosophila. *Development* **1999**, *126*, 5441–5452, doi:10.1242/dev.126.23.5441.

80. Gajewski, K.; Choi, C.Y.; Kim, Y.; Schulz, R.A. Genetically Distinct Cardial Cells within the Drosophila Heart. *Genesis* **2000**, *28*, 36–43, doi:10.1002/1526-968x(200009)28:1%3C36::aid-gene50%3E3.0.co;2-4.

81. Lockwood, W.K.; Bodmer, R. The Patterns of *Wingless*, *Decapentaplegic*, and *Tinman* Position the *Drosophila* Heart. *Mechanisms of Development* **2002**, *114*, 13–26, doi:10.1016/S0925-4773(02)00044-8.

82. Liu, J.; Qian, L.; Wessells, R.J.; Bidet, Y.; Jagla, K.; Bodmer, R. Hedgehog and RAS Pathways Cooperate in the Anterior–Posterior Specification and Positioning of Cardiac Progenitor Cells. *Developmental Biology* **2006**, *290*, 373–385, doi:10.1016/j.ydbio.2005.11.033.

83. Johnson, A.N.; Burnett, L.A.; Sellin, J.; Paululat, A.; Newfeld, S.J. Defective Decapentaplegic Signaling Results in Heart Overgrowth and Reduced Cardiac Output in Drosophila. *Genetics* **2007**, *176*, 1609–1624, doi:10.1534/genetics.107.073569.

84. Wu, M.; Sato, T.N. On the Mechanics of Cardiac Function of Drosophila Embryo. *PLOS ONE* **2008**, *3*, e4045, doi:10.1371/journal.pone.0004045.
85. Kadam, S.; McMahon, A.; Tzou, P.; Stathopoulos, A. FGF Ligands in Drosophila Have Distinct Activities Required to Support Cell Migration and Differentiation. *Development* **2009**, *136*, 739–747, doi:10.1242/dev.027904.
86. Dorey, K.; Amaya, E. FGF Signalling: Diverse Roles during Early Vertebrate Embryogenesis. *Development* **2010**, *137*, 3731–3742, doi:10.1242/dev.037689.
87. Johnson, A.N.; Mokalled, M.H.; Haden, T.N.; Olson, E.N. JAK/Stat Signaling Regulates Heart Precursor Diversification in Drosophila. *Development* **2011**, *138*, 4627–4638, doi:10.1242/dev.071464.
88. Graba, Y.; Gieseler, K.; Aragnol, D.; Laurenti, P.; Mariol, M.-C.; Berenger, H.; Sagnier, T.; Pradel, J. DWnt-4, a Novel Drosophila Wnt Gene Acts Downstream of Homeotic Complex Genes in the Visceral Mesoderm. *Development* **1995**, *121*, 209–218, doi:10.1242/dev.121.1.209.
89. Tauc, H.M.; Mann, T.; Werner, K.; Pandur, P. A Role for Drosophila Wnt-4 in Heart Development. *genesis* **2012**, *50*, 466–481, doi:10.1002/dvg.22021.
90. Tang, M.; Yuan, W.; Bodmer, R.; Wu, X.; Ocorr, K. The Role of Pygopus in the Differentiation of Intra-Cardiac Valves in Drosophila. *Genesis* **2014**, *52*, 19–28, doi:10.1002/dvg.22724.
91. Chen, Z.; Zhu, J.; Fu, Y.; Richman, A.; Han, Z. Wnt4 Is Required for Ostia Development in the *Drosophila* Heart. *Developmental Biology* **2016**, *413*, 188–198, doi:10.1016/j.ydbio.2016.03.008.
92. Trujillo, G.V.; Nodal, D.H.; Lovato, C.V.; Hendren, J.D.; Helander, L.A.; Lovato, T.L.; Bodmer, R.; Cripps, R.M. The Canonical Wingless Signaling Pathway Is Required but Not Sufficient for Inflow Tract Formation in the *Drosophila Melanogaster* Heart. *Developmental Biology* **2016**, *413*, 16–25, doi:10.1016/j.ydbio.2016.03.013.
93. Lo, P.C.H.; Skeath, J.B.; Gajewski, K.; Schulz, R.A.; Frasch, M. Homeotic Genes Autonomously Specify the Anteroposterior Subdivision of the *Drosophila* Dorsal Vessel into Aorta and Heart. *Developmental Biology* **2002**, *251*, 307–319, doi:10.1006/dbio.2002.0839.
94. Rosales-Vega, M.; Reséndez-Pérez, D.; Vázquez, M. Antennapedia: The Complexity of a Master Developmental Transcription Factor. *genesis* **2024**, *62*, e23561, doi:10.1002/dvg.23561.
95. Lovato, T.L.; Nguyen, T.P.; Molina, M.R.; Cripps, R.M. The Hox Gene Abdominal-A Specifies Heart Cell Fate in theDrosophila Dorsal Vessel. *Development* **2002**, *129*, 5019–5027, doi:10.1242/dev.129.21.5019.
96. Ponzielli, R.; Astier, M.; Chartier, A.; Gallet, A.; Thérond, P.; Sémériva, M. Heart Tube Patterning in Drosophila Requires Integration of Axial and Segmental Information Provided by the Bithorax Complex Genes and Hedgehog Signaling. *Development* **2002**, *129*, 4509–4521, doi:10.1242/dev.129.19.4509.
97. Monier, B.; Astier, M.; Sémériva, M.; Perrin, L. Steroid-Dependent Modification of Hox Function Drives Myocyte Reprogramming in the Drosophila Heart. *Development* **2005**, *132*, 5283–5293, doi:10.1242/dev.02091.
98. LaBeau, E.M.; Trujillo, D.L.; Cripps, R.M. Bithorax Complex Genes Control Alary Muscle Patterning along the Cardiac Tube of *Drosophila*. *Mechanisms of Development* **2009**, *126*, 478–486, doi:10.1016/j.mod.2009.01.001.
99. Schroeder, A.M.; Nielsen, T.; Lynott, M.; Vogler, G.; Colas, A.R.; Bodmer, R. Nascent Polypeptide-Associated Complex and Signal Recognition Particle Have Cardiac-Specific Roles in Heart Development and Remodeling. *PLOS Genetics* **2022**, *18*, e1010448, doi:10.1371/journal.pgen.1010448.
100. Zhu, J.; Fu, Y.; Nettleton, M.; Richman, A.; Han, Z. High Throughput in Vivo Functional Validation of Candidate Congenital Heart Disease Genes in Drosophila. *eLife* **2017**, *6*, e22617, doi:10.7554/eLife.22617.
101. Zhao, T.; Wang, M.; Li, Z.; Li, H.; Yuan, D.; Zhang, X.; Guo, M.; Qian, W.; Cheng, D. Wds-Mediated H3K4me3 Modification Regulates Lipid Synthesis and Transport in Drosophila. *Int J Mol Sci* **2023**, *24*, 6125, doi:10.3390/ijms24076125.
102. Daubresse, G.; Deuring, R.; Moore, L.; Papoulas, O.; Zakrajsek, I.; Waldrip, W.R.; Scott, M.P.; Kennison, J.A.; Tamkun, J.W. The Drosophila Kismet Gene Is Related to Chromatin-Remodeling Factors and Is Required for Both Segmentation and Segment Identity. *Development* **1999**, *126*, 1175–1187, doi:10.1242/dev.126.6.1175.
103. Huang, W.; Zhu, J.; Fu, Y.; van de Leemput, J.; Han, Z. *Lpt*, *Trr*, and *Hcf* Regulate Histone Mono- and Dimethylation That Are Essential for *Drosophila* Heart Development. *Developmental Biology* **2022**, *490*, 53–65, doi:10.1016/j.ydbio.2022.07.003.
104. Koemans, T.S.; Kleefstra, T.; Chubak, M.C.; Stone, M.H.; Reijnders, M.R.F.; Munnik, S. de; Willemsen, M.H.; Fenckova, M.; Stumpel, C.T.R.M.; Bok, L.A.; et al. Functional Convergence of Histone Methyltransferases EHMT1 and KMT2C Involved in Intellectual Disability and Autism Spectrum Disorder. *PLOS Genetics* **2017**, *13*, e1006864, doi:10.1371/journal.pgen.1006864.
105. Zhu, J.; Liu, C.; Huang, X.; van de Leemput, J.; Lee, H.; Han, Z. H3K36 Di-Methylation Marks, Mediated by Ash1 in Complex with Caf1-55 and MRG15, Are Required during Drosophila Heart Development. *Journal of Cardiovascular Development and Disease* **2023**, *10*, 307, doi:10.3390/jcdd10070307.

106. Zhu, J.-Y.; Lee, H.; Huang, X.; van de Leemput, J.; Han, Z. Distinct Roles for COMPASS Core Subunits Set1, Trx, and Trr in the Epigenetic Regulation of Drosophila Heart Development. *Int J Mol Sci* **2023**, *24*, 17314, doi:10.3390/ijms242417314.
107. Zhu, J.-Y.; van de Leemput, J.; Han, Z. Distinct Roles of COMPASS Subunits to Drosophila Heart Development. *Biol Open* **2024**, *13*, bio061736, doi:10.1242/bio.061736.
