# Supplementary material for: In Vivo Models of Cardiovascular Disease: Drosophila melanogaster as a Genetic Model of Congenital Heart Disease
Source: Biomedicines. 2025 Oct 21;13(10):2569. doi: 10.3390/biomedicines13102569 (PMC12561791; doi:10.3390/biomedicines13102569)
Supplement: Supplementary file 1 [file biomedicines-13-02569-s001.zip › biomedicines-3903996-supplementary_File S2.pdf]

Supplementary Information File S2 Section B

Table S8. *D. melanogaster* and *H. Sapiens* orthologs, summary of function for each gene in the context of cardiovascular development. In cases where a 1-to-many (1:N) or many-to-many (N:N) ortholog relationship is present, the ortholog with the highest DIOPT score, including weighted scores in parentheses, is noted in the corresponding column with additional orthologs, in descending DIOPT score order, included in a separate column. In cases where the highest predicted score is shared between more than one *H. sapiens* orthologs, these are noted in the first ortholog column.

Notes: ABL1/2, ABL Proto-Oncogene 1/2, Non-Receptor Tyrosine Kinase; ACVR1/1B/1C/2A/L1, Activin A Receptor Type 1/1B/1C/2A/L1; ADAMDEC1, ADAM Like Decysin 1; ADGRA3/D1/D2/E1/E2/E3/E5/F3/F4/F5/G2/G3/G4/G6/G7/L1/L2/L3/L4, Adhesion G Protein-Coupled Receptor 3/D1/D2/E1/E2/E3/E5/F3/F4/F5/G2/G3/G4/G6/G7/L1/L2/L3/L4; AFG3L2, AFG3 Like Matrix AAA Peptidase Subunit 2; AHI1, Abelson Helper Integration Site 1; ANKRD11-12, Ankyrin repeat domain containing 11/12; APOB, Apolipoprotein B; ARX, Aristaless Related Homeobox; ASCL1-5, Achaete-Scute Family BHLH Transcription Factor 1-5; ASH1L/2L, ASH1/2 Like Histone Lysine Methyltransferase; ATG16L2, Autophagy Related 16 Like 2; BARHL2, BarH Like Homeobox 2; BARX2, BARX Homeobox 2; BAZ1A, Bromodomain Adjacent To Zinc Finger Domain 1A; BCS1L, BCS1 Homolog, Ubiquinol-Cytochrome C Reductase Complex Chaperone; BHLHA9, Basic Helix-Loop-Helix Family Member A9; BLK, BLK Proto-Oncogene, Src Family Tyrosine Kinase; BMP2-15, Bone morphogenetic protein 2-15; BMPR1A/1B, Bone Morphogenetic Protein Receptor Type 1A/1B; BMX, Bone Marrow Tyrosine Kinase Gene In Chromosome X Protein; BORCS8, BLOC-1 Related Complex Subunit 8; BRCC3, BRCA1/BRCA2-Containing Complex Subunit 3; BSG, Basigin (Ok Blood Group); BTBD18, BTB Domain Containing 18; BTF3/3L4, Basic Transcription Factor 3/ 3 Like 4; BTK, Bruton Tyrosine Kinase; CCDC158/186, Coiled-Coil Domain Containing 158/186; CD320/34/42, CD320/34/42 Molecule; CDH1/20, Cadherin 1/20; CDX1/2/4, Caudal Type Homeobox 1/2/4; CELSR1/2/3, Cadherin EGF LAG Seven-Pass G-Type Receptor 1/2/3; CFAP52, Cilia And Flagella Associated Protein 52; CHADL, Chondroadherin Like; CHD1-9, Chromodomain Helicase DNA Binding Protein 1-9; CNTN4/6, Contactin 4/6; COPS5/6, COP9 Signalosome Subunit 5/6; CORIN, Corin, Serine Peptidase; CRB1/2, Crumbs Cell Polarity Complex Component 1/1; CSF1R, Colony Stimulating Factor 1 Receptor; CSK, C-Terminal Src Kinase; DAB1, DAB Adaptor Protein 1; DAG1, Dystroglycan 1; DAW1, Dynein Assembly Factor With WD Repeats 1; DBX1/2, Developing Brain Homeobox 1/2; DCAF11, DDB1 And CUL4 Associated Factor 11; DHH, Desert Hedgehog Signaling Molecule; DLL1, Delta Like Canonical Notch Ligand 1; DLX1-6, Distal-Less Homeobox 1-6; DNER, Delta/Notch Like EGF Repeat Containing; DOLPP1, Dolichyldiphosphatase 1; DPY30, Dpy-30 Histone Methyltransferase Complex Regulatory Subunit; DSCAM, DS Cell Adhesion Molecule; DSCAML1, DS Cell Adhesion Molecule Like 1; EDIL3, EGF Like Repeats And Discoidin Domains 3; EGF, Epidermal Growth Factor; EGFR, Epidermal Growth Factor Receptor; EHD2/3, EH Domain Containing 2/3; EHF, ETS Homologous Factor; EHMT1/2, Euchromatic Histone Lysine Methyltransferase 1/2; EIF3F, Eukaryotic Translation Initiation Factor 3 Subunit F; ELF5, E74 Like ETS Transcription Factor 5; ELSPBP1, Epididymal Sperm Binding Protein 1; EMX1/2, Empty Spiracles Homeobox 1/2; EOMES, Eomesodermin; EPHA10, EPH Receptor A10; EPS15, Epidermal Growth Factor Receptor Pathway Substrate 15; ERBB2-4, Erb-B2 Receptor Tyrosine Kinase 2-4; ESRRB, Estrogen Related Receptor Beta; EVX1/2, Even-Skipped Homeobox 1/2; EZH1/2, Enhancer Of Zeste 1/2 Polycomb Repressive Complex 2 Subunit; F9, Coagulation Factor IX; FAM43A/B, Family With Sequence Similarity 43 Member A; FAT4, FAT Atypical Cadherin 4; FBLN5/7, Fibulin 5/7; FBN1-3, Fibrillin 1-3; FBXO42, F-Box Protein 42; FBXW2, F-Box And WD Repeat Domain Containing 2; FCHSD1, FCH And Double SH3 Domains 1; FDPS, Farnesyl Diphosphate Synthase; FER, FER Tyrosine Kinase; FERD3L, Fer3 Like BHLH Transcription Factor; FES, FES Proto-Oncogene, Tyrosine Kinase; FGF8, Fibroblast growth factor 8; FGFR1/2/3/4/L1, Fibroblast Growth Factor Receptor 1/2/3/4/L1; FGR, FGR Proto-Oncogene, Src Family Tyrosine Kinase; FIGLA, Folliculogenesis Specific BHLH Transcription Factor; FLT1/3/4; FRK, Fyn Related Src Family Tyrosine Kinase; FRZB, Frizzled Related Protein; FYN, FYN Proto-Oncogene, Src Family Tyrosine Kinase; FZD1/10/2/3/4/5/6/7/8/9, Frizzled Class Receptor 1/10/2/3/4/5/6/7/8/9; G2E3, G2/M-Phase Specific E3 Ubiquitin Protein Ligase; GATA1-6, GATA Binding Protein 1-6; GBX1/2, Gastrulation Brain Homeobox 1/2; GDF1-10, Growth Differentiation Factor 1-10; GGPS1, Geranylgeranyl Diphosphate Synthase 1; GRAP/2/L, GRB2 Related Adaptor Protein; GRB14/2, Growth Factor Receptor Bound Protein 14/2; GSX1/2, GS Homeobox 1/2; GULP1, GULP PTB Domain Containing Engulfment Adaptor 1; HCFC1/2, Host Cell Factor C1/2; HCK, HCK Proto-Oncogene, Src Family Tyrosine Kinase; HMCN1/2, Hemicentin 1/2; HOXA1/10/11/2/3/4/5/6/7/9, Homeobox A1/10/11/2/3/4/5/6/7/9; HOXB1/13/2/3/4/5/6/7/8/9, Homeobox B1/13/2/3/4/5/6/7/8/9; HOXC10/11/12/4/5/6/8/9, Homeobox C10/11/12/4/5/6/8/9; HOXD1/10/11/12/13/3/4/8/9, Homeobox D1/10/11/12/13/3/4/8/9; HPX, Hemopexin; HSPG2, Heparan Sulfate Proteoglycan 2; IGDCC3, Immunoglobulin Superfamily DCC Subclass Member 3; IGF1R, Insulin Like Growth Factor 1 Receptor; IGLON5, IgLON Family Member 5; IGSF9, Immunoglobulin Superfamily Member 9/9B; IHH, Indian Hedgehog Signaling Molecule; INHA, Inhibin Subunit A/BA/B/C/E; ISL1/2, ISL LIM Homeobox 1/2; ITGA1/10/11/2/2B/3/4/5/6/7/8/9/D/E/L/M/V/X, Integrin Subunit Alpha 1/10/11/2/2B/3/4/5/6/7/8/9/D/E/L/M/V/X; ITGB1/2/3/4/5/6/7/8/L1, Integrin Subunit Beta 1/2/3/4/5/6/7/8/L1; ITK, IL2 Inducible T Cell Kinase; ITSN1/2, Intersectin 1/2; JAG1/2, Jagged Canonical Notch Ligand 1/2; JAK2/3, Janus Kinase 2/3; JARID2, Jumonji And AT-Rich Interaction Domain Containing 2; KBTBD4, Kelch Repeat And BTB Domain Containing 4; KDM4A/4B/4D/4E/4F/5A/5B/5C/5D, Lysine Demethylase 4A/4B/4D/4E/4F/5A/5B/5C/5D; KDR, Kinase Insert Domain Receptor; KIF13A/13B/14/16B/1A/1B/1C/2B, Kinesin Family Member 13A/13B/14/16B/1A/1B/1C/2B; KIRREL1-3, Kirre Like Nephrin Family Adhesion Molecule 1-3; KIT, KIT Proto-Oncogene, Receptor Tyrosine Kinase; KLHDC1/C3/C4, Kelch Domain Containing 1/3/4; LAM A1/5/B3/C1-3, Laminin Subunit A1/5/B3/C1-3; LCK, LCK Proto-Oncogene, Src Family Tyrosine Kinase; LDLR, Low Density Lipoprotein Receptor; LDLRAD3, Low Density Lipoprotein Receptor Class A Domain Containing 3; LDLRAP1, Low Density Lipoprotein Receptor Adaptor Protein 1; LEFTY1/2, Left-Right Determination Factor 1/2; LEP, Leptin; LGR6, Leucine Rich Repeat Containing G Protein-Coupled Receptor 6; LRP1/10/12/1B/2/3/4/5/6/8, LDL Receptor Related Protein 1/10/12/1B/2/3/4/5/6/8; LRRC3/3B/3C; LRTM1/2, Leucine Rich Repeat Transmembrane Protein 1/2; LYL1, LYL1 Basic Helix-Loop-Helix Family Member; LYN, LYN Proto-Oncogene, Src Family Tyrosine Kinase; LZTR1, Leucine Zipper Like Post Translational Regulator 1; MAP3K10, Mitogen-Activated Protein Kinase Kinase Kinase 10; MAPK8IP1-2, Mitogen-Activated Protein Kinase 8 Interacting Protein 1-2; MATK, Megakaryocyte-Associated Tyrosine Kinase; MATN2, Matrilin 2; MDC1, Mediator Of DNA Damage Checkpoint 1; MDGA2, MAM Domain Containing Glycosylphosphatidylinositol Anchor 2; MEGF10/11/6/9, Multiple EGF Like Domains 10/11/6/9; MEN1, Menin 1; MEOX1-2, Mesenchyme Homeobox 1-2; MESP1-2, Mesoderm Posterior BHLH Transcription Factor 1-2; MET, MET Proto-Oncogene, Receptor Tyrosine Kinase; MFRP, Membrane Frizzled-Related Protein; MGA, MAX Dimerization Protein MGA; MNX1, Motor Neuron And Pancreas Homeobox 1; MSC, Musculin; MSGN1, Mesogenin 1; MST1R, Macrophage

Stimulating 1 Receptor; MSTN, Myostatin; MSX1-2, Msh Homeobox 1-2; MYBPC1-3, Myosin Binding Protein C1-3; MYOM2, Myomesin 2; MYSM1, Myb Like, SWIRM And MPN Domains 1; NCAM1/2, Neural Cell Adhesion Molecule 1/2; NCK1/2, NCK Adaptor Protein 1/2; NHLH1, Nescient Helix-Loop-Helix 1; NKX2-1-8, NK2 Homeobox 1-8; NKX3-1/2, NK3 Homeobox 1/2; NKX6-1/2/3, NK6 Homeobox 1/2/3; NODAL, Nodal Growth Differentiation Factor; NOS1AP, Nitric Oxide Synthase 1 Adaptor Protein; NOTCH1/2/2NLA/2NLC/2NLR/3/4, Notch Receptor 1/2/2 N-Terminal Like A/2 N-Terminal Like C/2 N-Terminal Like R/3/4; NOTO, Notochord Homeobox; NR1D1-2, Nuclear Receptor Subfamily 1 Group D Member 1-2; NR1H2-4, Nuclear Receptor Subfamily 1 Group H Member 2-4; NR1I2-3, Nuclear Receptor Subfamily 1 Group I Member 2-3; NR2C2/E1/E3/F1/F2/F6, Nuclear Receptor Subfamily 2 Group C Member 2/ E Member 1/3/ F Member 1/2/6; NSD1-3, Nuclear Receptor Binding SET Domain Protein 1-3; NTM, Neurotrimin; NTN1/3/5, Netrin 1/3/5/G1/2; NUMB/L, NUMB/NUMB Like Endocytic Adaptor Protein; NUP37, Nucleoporin 37; NYX, Nyctalopin; OBSL1, Obscurin Like Cytoskeletal Adaptor 1; PAAF1, Proteasomal ATPase Associated Factor 1; PACSIN1-3, Protein Kinase C And Casein Kinase Substrate In Neurons 1-3; PAFAH1B1, Platelet Activating Factor Acetylhydrolase 1b Regulatory Subunit 1; PARL, Presenilin Associated Rhomboid Like; PAXIP1, PAX Interacting Protein 1; PDGFA/B/C/D, Platelet Derived Growth Factor Subunit A/B/C/D; PDGFRA/B, Platelet Derived Growth Factor Receptor A/B; PDSS1/2, Decaprenyl Diphosphate Synthase Subunit 1/2; PDX1, Pancreatic And Duodenal Homeobox 1; PEX10, Peroxisomal Biogenesis Factor 10; PGF, Placental Growth Factor; PHF11/6/7, PHD Finger Protein 11; PLPP1/2/3/4/5, Phospholipid Phosphatase 1/2/3/4/5; PLPPR1/2/3/4/5, Phospholipid Phosphatase Related 1/2/3/4/5; PLRG1, Pleiotropic Regulator 1; POC1A, POC1 Centriolar Protein A; PPARA/D/G, eroxisome Proliferator Activated Receptor A/D/G; PRG4, Proteoglycan 4; PSMD14/7, Proteasome 26S Subunit, Non-ATPase 14/7; PTF1A, Pancreas Associated Transcription Factor 1a; PTK2/6, Protein Tyrosine Kinase 2/6; PTPN6, Protein Tyrosine Phosphatase Non-Receptor Type 6; PTPRQ, Protein Tyrosine Phosphatase Receptor Type Q; PYGO1/2, Pygopus Family PHD Finger 1/2; RABEPK, Rab9 Effector Protein With Kelch Motifs; RAC1/2/3, Rac Family Small GTPase 1/2/3; RARA/B, Retinoic Acid Receptor A/B; RAX/2, Retina And Anterior Neural Fold Homeobox/2; RBBP5, RB Binding Protein 5, Histone Lysine Methyltransferase Complex Subunit; REPS1, RALBP1 Associated Eps Domain Containing 1; RET, Ret Proto-Oncogene; RHBDF1/F2/L1/L2/L3, Rhomboid 5 Homolog 1/2/ Rhomboid Like Homolog 1/2/3; RHEB, Ras Homolog, MTORC1 Binding; RHOA/B/BTB1/BTB2/D/F/G/H/J/Q/U/V, Ras Homolog Family Member A/B/BTB1/BTB2/D/F/G/H/J/Q/U/V; RIPK1, Receptor Interacting Serine/Threonine Kinase 1; RND1-3, Rho Family GTPase 1-3; RNF20/4/40, Ring Finger Protein 20/4/40; ROBO1-4, Roundabout Guidance Receptor 1-4; ROR1/A/B/C, Receptor Tyrosine Kinase Like Orphan Receptor 1/A/B/C; ROS1, ROS Proto-Oncogene 1, Receptor Tyrosine Kinase; RTN4RL1, Reticulon 4 Receptor Like 1; RXRA/B/G, Retinoid X Receptor A/B/G; RYK, Receptor Like Tyrosine Kinase; SCARF1, Scavenger Receptor Class F Member 1; SCX, Scleraxis BHLH Transcription Factor; SDK1/2, Sidekick Cell Adhesion Molecule 1/2; SETBP1, SET Binding Protein 1; SETD1A/1B/2/B1/B2, SET Domain Containing 1A/1B/2/B1/B2, Histone Lysine Methyltransferase; SFRP1-5, Secreted Frizzled Related Protein 1-5; SH3D19, SH3 Domain Containing 19; SHH, Sonic Hedgehog; SHOXA5, Short Stature Homeobox A5; SLA, Src Like Adaptor/2; SLIT1-3, Slit Guidance Ligand 1-3; SLITRK4, SLIT And NTRK Like Family Member 4; SMAD1-9, SMAD Family Member 1-9; SMO, Smoothened, Frizzled Class Receptor; SNED1, Sushi, Nidogen And EGF Like Domains 1; SORL1, Sortilin Related Receptor 1; SPATA5L1, Spermatogenesis associated 5L1; SPG7, SPG7 Matrix AAA Peptidase Subunit, Paraplegin; SRC, SRC Proto-Oncogene, Non-Receptor Tyrosine Kinase; SRF, Serum Response Factor; SRMS, Src-Related Kinase Lacking C-Terminal Regulatory Tyrosine And N-Terminal Myristylation Sites; STAM2, Signal Transducing Adaptor Molecule 2; STAMBP/L1, STAM Binding Protein; STARD9, StAR Related Lipid Transfer Domain Containing 9; STYK1, Serine/Threonine/Tyrosine Kinase 1; SUV39H2, SUV39H2 Histone Lysine Methyltransferase; TAF5, TATA-Box Binding Protein Associated Factor 5; TAL1/2, TAL BHLH Transcription Factor 1/2, Erythroid Differentiation Factor; TBL1X, Transducin Beta Like 1 X-Linked; TBL1XR1, TBL1X/Y Related 1; TBL1Y, Transducin Beta Like 1 Y-Linked; TBL3, Transducin Beta Like 3; TBR1, T-Box Brain Transcription Factor 1; TBRG1, Transforming Growth Factor Beta Regulator 1; TBX1-22, T-Box Transcription Factor 1-22; TBXT, T-Box Transcription Factor T; TCF15-24, Transcription Factor 15-24; TEC, Tec Protein Tyrosine Kinase; TEK, TEK Receptor Tyrosine Kinase; TGFB1/B2/B3, Transforming Growth Factor B1/B2/B3; TGFBR1, Transforming Growth Factor Beta Receptor 1; THRA, yroid Hormone Receptor Alpha; TIE1, Tyrosine Kinase With Immunoglobulin Like And EGF Like Domains 1; TIMP1-4, Tissue inhibitor of metalloproteinase 1-4; TLR7, Toll Like Receptor 7; TLX2, T Cell Leukemia Homeobox 2; TOPBP1, DNA Topoisomerase II Binding Protein 1; TRIP10, Thyroid Hormone Receptor Interactor 10; TRPS1, Transcriptional Repressor GATA Binding 1; TTN, Titin; TWIST1/2, Twist Family BHLH Transcription Factor 1/2; TXK, TXK Tyrosine Kinase; TYK2, Tyrosine Kinase 2; UBE2A/B/O/U, Ubiquitin Conjugating Enzyme E2 A/B/O/U; USH2A, Usherin; USP17L1-30, Ubiquitin Specific Peptidase 17 Like Family Member 1-30; USP27X, Ubiquitin Specific Peptidase 27 X-Linked; USP3-49, Ubiquitin Specific Peptidase 3-49; VAX1/2, Ventral Anterior Homeobox 1/2; VCAN, Versican; VDR, Vitamin D Receptor; VEGFA/B/C/D, Vascular endothelial growth factor A/B/C/D; VLDLR, Very Low Density Lipoprotein Receptor; VTN, Vitronectin; WDR5-88, WD Repeat Domain 5-88; WNT1-9, Wingless-related integration site 1-9; WSB2, WD Repeat And SOCS Box Containing 2; YES1, YES Proto-Oncogene 1, Src Family Tyrosine Kinase; YME1L1, YME1 Like 1 ATPase; ZGLP1, Zinc Finger GATA Like Protein 1; ADAM2-33, A Disintegrin and Metalloproteinase 2-33; ANTP-C, Antennapedia Complex; Abd-A, Abdominal-A; Abd-B, Abdominal-B; Ankrd11, Ankyrin repeat domain containing 11; Antp, Antennapedia; Ash1, Absent, small or homeotic disks 1; Ash2, Absent, small or homeotic disks 2; BARX, BarH-like homeobox; BARHL, BarH-like homeobox; BMP, Bone morphogenetic protein; BTBD18, BTB Domain Containing 18; BX-C, Bithorax Complex; Bre1, B recognition element 1; COMPASS, Complex of proteins associated with Set1; Cdc42, Cell division control protein 42; Col6A2, Collagen Type VI α2 chain; D-mef2, Drosophila Myocyte enhancer factor 2; D. melanogaster, Drosophila melanogaster; DE-Cadherin, Drosophila E-Cadherin; DIOPT, Drosophila RNAi Screening Center Integrative Ortholog Prediction Tool; DPE, Downstream promoter element; DPR, Downstream promoter region; DWnt4, Drosophila Wingless-related integration site 4; Dap160, Dynamin-associated protein 160 kilo Dalton (kD); Dfz2, Dfrizzled-2; Dg, Dystroglycan; Dmef2, Drosophila myocyte enhancer factor 2; Dome, domeless; Donson, Downstream neighbour of Son; Dpp, Decapentaplegic; Dpy-30L1, Dpy-30 like 1; Dscam, Down syndrome Cell adhesion molecule; EGF, Epidermal growth factor; EGFR, Epidermal growth factor receptor; EcR, Ecdysone receptor; Eve, Even-skipped; FGF, Fibroblast growth factor; FGFR, Fibroblast growth factor receptor; Fz, Frizzled; Fz2, Frizzled2; GGPPS/qm, Geranylgeranyl pyrophosphate synthase; GPCR, G-protein coupled receptor; Gart, Phosphoribosylglycinamide Formyltransferase, Phosphoribosylglycinamide synthetase, Phosphoribosylaminoimidazole synthetase; Gata4, GATA binding protein 4; Gia, G-protein coupled receptor in aorta; Gγ1, G protein gamma (γ) subunit 1; HMGCR, Hydroxymethyl-glutaryl (HMG) CoA reductase; Hand, Heart- and Neural crest derivatives-expressed protein; Hcf, Host cell factor; Hd, Humpty dumpy; Hh, Hedgehog; Hox, Homeobox; Isl1, Insulin-related protein 1; Itsn1, Intersectin 1; KMT2A/2B/2C/2D, Lysine methyltransferase 2A/2B/2C/2D; Kif1A, Kinesin family member 1A; Kuz, Kuzbanian; LRP2, Low-density lipoprotein-related receptor 2; Lb, Ladybird; Lbx, Ladybird homeobox; Lid, Drosophila ortholog of KDM5A/5B; Lpt, Lost PHDs of Trithorax-related (Trr); MICOS, Mitochondrial contact site and cristae organization system; MMP1-29, Matrix metalloproteinase 1-29; MYOM2, Myomesin 2; Mef2A/2B/2C/2D, Myocyte enhancer factor 2A/2B/2C/2D; Mhc, Myosin heavy chain; Mnn1, Menin 1; Msh, MutS homolog; Msx, MutS

homolog (Msh) homeobox; Naca, Nascent-associated polypeptide complex, alpha subunit; NetA, netrin A; NetB, Netrin B; Nkx2, Nk2 homeobox; Nkx6, Nk6 homeobox; Nr2f2, Nuclear Receptor subfamily 2 group F member 2; Odd, Odd-skipped; Org-1, Optomotor blind related gene 1; PVR, Platelet-derived growth factor/Vascular endothelial growth factor receptor; Pdss2, Decaprenyl diphosphate synthase, subunit 2; Ptip, PAX Transcription activation domain interacting protein; Rbbp5, Retinoblastoma binding protein 5; Rho, Rhomboid; Robo2, Roundabout2; RpL13, Ribosomal protein L13- L14; RpS24, Ribosomal protein S24; Rpn8, Regulatory particle, Proteasome subunit Rpn8; SCE, sevenup (svp) cardiac enhancer; Scny, Scrawny; Set1, SET Containing domain 1; Set2, SET Containing domain 2; Shh, Sonic hedgehog; Sli, Slit; Slit, Robo, Roundabout; Smox, drosophila ortholog of SMAD2; Son, SON Deoxyribonucleic acid (DNA) and Ribonucleic acid (RNA); Spg7, Spastic paraplegia 7; Src, Sex combs reduced; Src42A, How, Held out wings; Stat, Signal transducer and activator of transcription; Stat92E, Signal transducer and activator of transcription 92E; TF, Transcription factor; TGFβ, Transforming growth factor beta; Tbx1, T-box transcription factor TBX1; Tbx2, T-box transcription factor TBX2; Tbx20, T-box transcription factor TBX20; Tbx5, T-box transcription factor TBX5; Tbx6, T-box transcription factor TBX6; Timp, Tissue inhibitor of metalloproteinase; Tkv, Thickveins; Trr, Trithorax related; Trx, Trithorax; TrxG, Trithorax Group genes; UbcD6, D. melanogaster ortholog of Ubiquitin conjugating enzyme (UBE); Ubx, Ultrabithorax; Upd, Unpaired; Vegf, Vascular endothelial growth factor; Wdr82, WD repeat domain 82; Wds, Will die slowly; Wg, Wingless; Wnt, Wingless-related integration site; Wun, Wunen; Wun2, Wunen2; apoLpp, a homolog of apolipoprotein B; bHLH, basic Helix-Loop-Helix; bab2, bric-a-brac 2; bic, bicaudal; coQ10, Coenzyme Q10; dChchd3/6, drosophila Coiled-coil-helix-coiled-coil-helix-domain containing protein 6; dMnM, Drosophila Myomesin and Myosin protein; doc, dorsocross; lanA, Laminin A; lb, ladybird; mgl, megalin; mid, midline; mmp1, Matrix metalloproteinase 1; mmp2, matrix metalloproteinase 2; mys, myospheroid; nmr1, neuromancer 1; nmr2, neuromancer 2; omb, optomotor blind; pnr, pannier; pyr, pyramus; scb, scab; spdo, sanpodo; srp, serpent; svp, Sevenup; ths, thisbe; tin, tinman; tup, tailup; αPS2, α subunit integrin chain 2; αPS3, α subunit integrin chain; βGGT-I, Geranylgeranyl transferase type I beta (β) subunit; βPS, β subunit integrin chain.

Gene	Information	Highest score Orthologue	*Additional Orthologues*	DIOPT score	Vertebrate Orthologue Information	Study (Reference)
Abd-A	**Homeobox containing (Hox) gene, part of the Bithorax Complex (BX-C) of genes;** Expressed in segments A5-A8, determines the identity of the Posterior Dorsal vessel (**heart chamber**) in the *D. melanogaster* embryo, implicated in AM development in the same location	***HOXB6, HOXC6, HOXA6***	*HOXC5, HOXA7, HOXB7, HOXB5, SHOXA5, HOXD4, HOXA4, HOXB4, HOXC4, HOXC8, HOXD13, HOXA2, HOXD8, GSX2, HOXB8, HOXB2, MNX1, HOXC9, HOXA9, BARX2, HOXB9, HOXA10, HOXD1, MEOX1, HOXB1, GSX1, HOXD3, MEOX2, GBX1, PDX1, HOXA3, GBX2*	**5** (4.87)	In general, anterior *HOX* groups (*HOX1-HOX9*) are homologous to *D. melanogaster ANTP-C* and *Abd*-A [1]<br><br>*HOXA/HOXB* cluster evolution associated with diversification in cardiac form and emergence of complex cardiac shapes including cardiac looping [9]<br><br>***HOXA5, HOXB6, HOXC6*** may correlate with vascular smooth muscle cell de-differentiation in thoracic aortic dissection [10] (**Human**)<br><br>***HOXC6, HOXC8*** involved in vascular smooth muscle cell differentiation [11]<br><br>***HOXA6* has not yet been specifically associated with cardiac development** [11]; ***HOXA6*** amongst the genes regulating differences between upper- and lower-body adipose tissue distribution [12]	**Lo et al., 2002** [2], **Lovato et al., 2002** [3], **Ponzielli et al., 2002** [4], **Perrin et al., 2004** [5], **Ryan et al., 2005** [6], **Monier et al., 2005** [7], **LaBeau et al., 2009** [8]
Abd-B	**Homeobox containing (Hox) gene, part of the Bithorax**	***HOXA10,*** *HOXB13*	*HOXC10, HOXD10, HOXD9, HOXA9, HOXB9, HOXD11,*	**6** (6.01)	In general, posterior *HOX* groups (*HOX9-HOX13*) are homologous to *D. melanogaster Abd-B* [1]	**Lo et al., 2002** [2], **Lovato et al., 2002** [3], **Perrin et al., 2004** [5],

Gene	Information	Highest score Orthologue	*Additional Orthologues*	DIOPT score	Vertebrate Orthologue Information	Study (Reference)
	**Complex (BX-C) of genes;** Expressed in segments A6-A7, determines the identity of the Posterior Dorsal vessel (**heart chamber**) and A8 (**heart terminus**) in the *D. melanogaster* embryo, Involved in Dorsal vessel remodeling and histolysis of segments A6-A7 (**Metamorphosis**) triggered by ecdysone secretion, Function regulated by **NAC chaperone**		*HOXC9, HOXC12, HOXA11, HOXC11, HOXD13, HOXD12, HOXB13, HOXA7, HOXB7, HOXC8, HOXB8, HOXD8, HOXD4, HOXA4, HOXB6, HOXA2, HOXC6, HOXB4, HOXB5, HOXC5, HOXA5, HOXC4, HOXA6*		*HOXA/HOXB* cluster evolution associated with diversification in cardiac form and emergence of complex cardiac shapes including cardiac looping [9]<br><br>***HOXA9, HOXA10*** expressed in the right side of the cardiac crescent during early development; ***HOXA10*** interacts with *NKX2.5* to regulate timing of cardiac mesoderm differentiation [1], participates in a left-to-right temporal wave of cardiac differentiation across *NKX2.5* expressing cells in the early cardiac crescent (**In vitro models**)<br><br>***HOXB13*** involved in cardiomyocyte maturation [11]	**Schroeder et al., 2022** [13]
Antp	**Homeobox containing (Hox) gene, part of the Antennapedia Complex (ANTP-C) of genes;** Expressed in segments T2-A3, determines the identity of the Anterior Dorsal vessel (**aorta**) in the *D. melanogaster* embryo, *Antp* expression in the Posterior Dorsal vessel repressed by *Ubx*	***HOXA7, HOXA1, HOXA3***	*HOXB7, HOXB6, HOXC6, HOXA6, HOXB5, HOXA5, HOXB8, HOXC8, HOXC5, HOXD8, HOXD9, HOXD4, HOXA4, HOXB4, HOXC4, HOXD3, PDX1, HOXD13, HOXB3, HOXC9, HOXA9, NKX6-2, HOXB9, HOXD11, HOXD1, MEOX1, HOXA2, HOXB1, GSX1, GSX2, MEOX2, GBX1, HOXB2, GBX2, MNX1, EMX1, CDX2, BARX2, CDX4, HOXC11, DLX4, RAX, HOXD10, DBX2, CDX1, DBX1, NKX3-1, TLX2, HOXC12, HOXA10, NKX6-1, RAX2, EMX2, NKX6-3, HOXA1, HOXA3*	**9** (8.94)	In general, anterior *HOX* groups (*HOX1-HOX9*) are homologous to *D. melanogaster ANTP-C* and *Abd*-A [1]<br><br>*HOXA/HOXB* cluster evolution associated with diversification in cardiac form and emergence of complex cardiac shapes including cardiac looping [9]<br><br>***HOXA1*** expressed in the Hindbrain, where cardiac Neural crest cell progenitors arise during development, regulates specification of neural crest cells, involved in patterning of pharyngeal arch arteries and outflow tract [14] [15]; ***HOXA1, HOXB1*** interact with each other, expressed in a subpopulation of second heart field (SHF) progenitors that contributes to the outflow tract [16] **(Mouse)**<br><br>***HOXA3*** expressed in the pharyngeal endoderm and cardiac neural crest cells of the 3rd pharyngeal pouch, subpopulation of second heart field progenitors that contributes to the outflow tract, expression regulated by retinoic acid signaling [17] (**Mouse**)<br><br>***HOXA7* has not yet been associated with cardiac development** [11]; ***HOXA7*** downregulated in animal	**Lo et al., 2002** [2], **Perrin et al., 2004** [5]

Gene	Information	Highest score Orthologue	*Additional Orthologues*	DIOPT score	Vertebrate Orthologue Information	Study (Reference)
					models of aortic valve stenosis sclerostin KO, though ***HOXA7*** role in aortic valve stenosis questionable [18] (**Mouse**)	
apoLpp	**Lipophorin (LP ligand) protein component;** Component of Lipophorin (LP ligand), Involved in hemolymph lipid transport, Wnt and Shh signaling regulation	***APOB, LOC400499***	N/A	**3** (2.88, 2.82)	***APOB*** involved in Wnt and Shh signaling regulation, Involved in the regulation of cardiomyocyte proliferation via a downregulation of cell cycle inhibitors and pro-apoptotic factors (TP53, CDKN1A), upregulation of cell-cycle genes (**In vitro models**)	**Theis et al., 2020** [19]
Apt	**bZIP transcription factor;** Involved in cardiac development including late phase cardiac progenitor migration and Dorsal vessel assembly	N/A	N/A	N/A	N/A	**Su et al., 1999** [20], **Liu et al, 2014** [21]
Ash1	**Histone methyltransferase;** Methylation at H3K36, Characterized by steady expression throughout development	***ASH1L***	*NSD2, NSD1, SETBP1, SETD2, NSD3, KMT2D, KMT2C, SETD1B, SETD1A, EHMT1, EZH1, SETDB2, KMT2B, KMT2A, SETDB1*	**14** (13.69)	***ASH1L*** is involved in mouse embryonic stem cell differentiation (**In vitro**), ***HOX*** gene expression [22], including *HOXA6, HOXA10* [23]	**J. Zhu et al., 2023** [24]
Ash2	**Histone methyltransferase, COMPASS Common subunit (Set1/COMPASS, Trx COMPASS-like, Trr COMPASS-like);** Methylation at H3K4	***ASH2L***	N/A	**17** (16.75)	***ASH2L*** is involved in cardiac development via interaction with ***TBX1*** (*ASH2L* is a co-factor for *TBX1*, augments *TBX1* activation), associates with SMYD1 to activate the ***ISL1*** promoter; Exhibits overlapping expression patterns with *TBX1*, upregulates the expression of genes such as ***HOXC8, HOXA9*** via H3K4 methylation [25]	**Zhu et al., 2024** [26]
bab1 bab2	**BTB/POZ domain transcription factor;** regulates dimorphic pigmentation, ovary morphogenesis, Implicated in cardioblast/pericardial cell	***BTBD18***	*KBTBD4*	**4** (3.91)	***BTBD18*** involved in cell proliferation, meiosis and spermiogenesis via regulation of PIWI RNA (piRNA) gene loci [27]	**Junion et al., 2007** [28], **Couderc et al., 2002** [29]

Gene	Information	Highest score Orthologue	*Additional Orthologues*	DIOPT score	Vertebrate Orthologue Information	Study (Reference)
	diversification, somatic muscle cell differentiation					
bic	**NAC β-subunit protein (bicaudal), part of the NAC chaperone complex;** NAC-ribosome complexes direct transport of nascent polypeptide chains to mitochondria, transport of other polypeptide chains to the endoplasmic reticulum, Implicated in Dorsal vessel remodeling (**Metamorphosis**)	***BTF3, BTF3L4***	N/A	**15** (14.87, 14.80)	***BTF3, BTF3L4*** **have not yet been associated with cardiac development**	**Schroeder et al., 2022** [13]
bifid (also known as omb)	**T-Box transcription factor (Brachyury-like);** Involved in the development of the CNS, wings and abdominal segments	***TBX2, TBX3***	*TBX1, TBX18, TBX15, TBX5, TBX22, TBX10, TBX4, TBX6, MGA, TBX19, TBXT, EOMES, TBX21, TBR1, TBX20*	**12** (11.89, 11.85)	***TBX2*** is involved in suppressing the developmental network of chamber formation in the atrioventricular canal; Interacts with *TBX3* and BMP2 to direct endocardial cushion development, regulate chamber development by locally repressing chamber differentiation; Involved in atrioventricular node development and atrioventricular canal myocardial tissue development [30] [31]	**Liu et al., 2018** [32]
Bre1	**Ubiquitin ligase E3;** Ubiquitinates at H2BK120ub, which is required for histone methylation at H3K4me	***RNF40***	*RNF20, PEX10, RNF4*	**16** (15.74)	***RNF40*** **is involved in Chromatin remodeling [Histone 2B monoubiquitylation (H2Bub1)] by associating with E3 ubiquitin RNF20, Ubiquitin ligase UEB2B via formation of the H2Bub1-deposition complex** [33]; Involved in left-right patterning and ciliogenesis [33] (**Mouse**)	**Zhu et al., 2017** [34]
Cdc42	**Small GTPase protein Cdc42;** Involved K+ channel function, Regulated by *tin* via miR-1, Involved in cardiac lumen formation	***CDC42***	*RHOG, RAC1, RAC3, RAC2, RHOQ, RHOJ, RHOU, RHOV, RHOF, RND1, RND2, RND3, RHOH, RHOD, RHEB, RHOBTB1, RHOA, RHOB, RHOBTB2*	**12** (12)	**CDC42** is regulated by Focal adhesion kinase (FAK), involved in cardiomyocyte adherens junction and sarcomere formation, regulates cell polarity, facilitates cardiomyocyte proliferation and expansion of ventricles during cardiac development [35], regulation of cardiac Neural crest cell migration via BMP2 [36] and endothelin signaling [37]	**Qian et al., 2011**[38], **Voglet et a., 2014** [39]

Gene	Information	Highest score Orthologue	*Additional Orthologues*	DIOPT score	Vertebrate Orthologue Information	Study (Reference)
CG10585	**Decaprenyl diphosphate synthase subunit 2;** Involved in coQ10 biosynthesis and Mitochondrial respiration	***PDSS2***	*GGPS1, PDSS1*	**16** (15.8)	***PDSS2*** is involved in the coQ10 biosynthetic pathway, participates in mitochondrial respiration and normal mitochondrial function	**Schroeder et al., 2019** [40]
CG10984	**Chromatin regulator (histone acetylation) protein;** Involved in modulation of histone acetylation and recruitment of Histone deacetylase enzymes	***ANKRD12***	*ANKRD11*	**9** (8.85)	***ANKRD12*** is implicated in cardiac development via upregulation of *NKX2.5, GATA4* early in development, upregulation of genes associated with the cardiomyocyte phenotype (*MLC-2v, ANF, cTnC*) via sequestration of Y-box binding protein 1 [41]	**Schroeder et al., 2019** [40]
CG2658	**Mitochondrial protease;** Involved in physiological mitochondrial function	***SPG7***	*AFG3L2, LOC101930112, YME1L1, SPATA5L1, BCS1L*	**12** (12.06)	***SPG7*** is involved in the mitochondrial unfolded protein response mechanism, regulates mitochondrial proteome and contributes to mitochondrial proteostasis, alongside other proteins; upregulated by an increase in reactive oxygen species within cardiomyocytes [42]	**Schroeder et al., 2019** [40]
D-mef2	**MADS-domain transcription factor;** upregulates genes involved in cardiac development (structural, contractile proteins)	***MEF2C, MEF2A***	*MEF2D, MEF2B, BORCS8-MEF2B, SRF*	**13** (12.96, 12.86)	***MEF2C, MEF2A*** involved in differentiation and development of cardiomyocytes, Cardiac looping, right ventricle development [43]	**Lilly et al., 1995** [44], **Hu et al., 2011** [45]
Dap160	**Adapter protein;** Involved in neuronal synapsis morphology, synaptic vesicle recycling	***ITSN1***	*ITSN2, EHD3, TRIP10, PACSIN1, PACSIN2, SH3D19, GRAP2, PACSIN3, FCHSD1, GRB2, STAM2, REPS1, EHD2, EPS15*	**16** (15.8)	***ITSN1*** regulates intracellular vesicle trafficking; Interacts with actin filaments, contributes to the ubiquitination and degradation of EGFR, thus downregulating EGFR-dependent pathways	**Schroeder et al., 2019** [40]
dChchd3 /6	**Mitochondrial MICOS complex subunit protein;** Mitochondrial MICOS complex subunit, Involved in mitochondrial maintenance and heart function	***CHCHD3 CHCHD6***	N/A	**11 6**	***CHCHD3, CHCHD6*** are involved in mitochondrial maintenance (maintenance of cristae morphology, ETC component assembly, mitochondrial fission/fusion) and heart function	**Birker et al., 2023** [46]

Gene	Information	Highest score Orthologue	*Additional Orthologues*	DIOPT score	Vertebrate Orthologue Information	Study (Reference)
Dg	**Muscle Dystroglycan protein;** Involved in cardiac lumen formation along with Slit, Robo proteins	***DAG1***	N/A	**11** (10.72)	***DAG1* has not yet been associated with cardiac development; *DAG1*** associated with skeletal muscle development [47]	**Medioni et al., 2008** [48]
dMnM	**Myosin-binding protein;** Interacts with *Mhc,* implicated in cardiac function	***TTN,*** *MYOM2*	*MYBPC3, MYBPC1, MYBPC2, MYOM1, MYOM2, MYOM3*	**4** (3.81)	**TTN** is a Myosin-binding protein, associated with the sarcomere<br><br>**MYOM2** is expressed in adult cardiac tissue and fast muscle fibers, associated with the M-band of the sarcomere, expressed during heart development in lower, *MYOM1, MYOM2* interact with the transcription factor MEF2C	**Auxerre-Plantié et al., 2020** [49]
doc1<br>***doc2***<br>***doc3***	**T-box transcription factor;** Differentiation and maintenance of amnioserosa, ectoderm patterning, **part of the core regulatory network directing heart development**	***TBX6***	*TBX1, TBX18, TBX21, TBX22, EOMES, TBR1, TBX5, TBX10, TBX4, MGA, TBX15,* ***TBX2,*** *TBX19, TBXT,* ***TBX3****, TBX20*	**10** (9.88)	***TBX6*** involved in mesoderm and paraxial mesoderm induction, regulation of cardiac mesoderm induction (**In vitro**) [50], regulation of left-right patterning (**Mouse**)<br><br>***TBX2*** is involved in development of the atrioventricular canal, outflow tract [52]<br><br>***TBX3*** is involved in atrial, ventricular cardiomyocyte development associated with the cardiac conduction system [53]	**Han and Olson, 2005** [51]
Dpp	**TGF-β protein;** TGF-β protein morphogen and Tkv ligand, Involved in Dpp (BMP) signaling, mesoderm migration along the overlying ectoderm, dorsoventral patterning, cardiac and visceral mesoderm development	***BMP2***	*BMP4, BMP7, BMP8B, BMP6, BMP5, BMP8A, GDF1, GDF3, BMP10, GDF5, GDF6, GDF2, GDF9, BMP15, GDF7, LEFTY1, GDF15, TGFB2, NODAL, INHBB, TGFB3, INHBA, TGFB1, LEFTY2, GDF10, MSTN, INHA, INHBC, BMP3, INHBE, GDF11*	**12** (11.84)	BMP signaling involved in gastrulation and development of the primitive mesoderm, formation of the first heart field (FHF) and the cardiac crescents, development of the endocardial cushions in the atrioventricular canal and the outflow tract<br><br>***BMP2*** is expressed in atrioventricular canal and outflow tract myocardium [31], involved in cardiac jelly development, induces endothelial-to-mesenchymal transition and atrioventricular canal myocardium patterning [56]	**Lockwood and Bodmer, 2002** [54],<br>**Johnson et al., 2007** [55]

Gene	Information	Highest score Orthologue	*Additional Orthologues*	DIOPT score	Vertebrate Orthologue Information	Study (Reference)
Dpy-30L1	**Histone methyltransferase, COMPASS Common subunit (Set1/COMPASS, Trx COMPASS-like, Trr COMPASS-like);** Methylation at H3K4	***DPY30***	N/A	**10** (9.95)	***DPY30*** is involved in the regulation of embryonic stem cell fate decisions via modulation of H3K4 methylation (**In vitro**)<br>***DPY30*** maintains pluripotency pre-gastrulation [57] (**Mouse**)<br>***DPY30* has not yet been explicitly associated with cardiac development in other animal models** [57]; ***DPY30*** involved in hematopoietic stem cell development [58]	**Zhu et al., 2024** [26]
Dscam	**Cell adhesion protein;** Netrin receptor, Implicated in filopodia, lamellipodia formation in migrating cardioblasts	***DSCAM***	*USH2A, KIRREL3, KIRREL2, CNTN4, ROBO3, CNTN2, BSG, CNTN6, MDGA2, KIRREL1, NCAM2, IGSF9B, NCAM1, SDK1, IGLON5, SDK2, IGSF9, NTM*	**12** (12.01, 11.84)	***DSCAM*** is an immunoglobulin cell adhesion molecule, involved in cardiac development, particularly fusion of the endocardial cushions [59], proliferation of second heart field (SHF) cardiac mesoderm progenitors in the dorsal body wall (Dorsal mesenchymal protrusion) which form the muscular portions of the atrial and atrioventricular canal septae [60]	**Grossman et al., 2011** [61], **Raza and Jacobs, 2016** [62]
EcR	**Ecdysone receptor protein;** Receptor for ecdysone, Ecdysone/EcR binding triggers Dorsal vessel remodelling and histolysis of segments A6-A7 during metamorphosis, Ecdysone also implicated in valve formation	***NR1H2, NR1H3***	*NR1H4, THRA, RARB, RARA, VDR, NR1I2, NR1I3, NR2F1, ESRRB, NR2C2, RORB, RORA, NR1D1, RORC, PPARG, PPARA, PPARD, NR1D2*	**12** (11.88, 11.7)	***NR1H2, NR1H3* have not yet been associated with cardiac development; *NR1H2*** variants associated with Type 2 Diabetes mellitus [63], ***NR1H3*** associated with lipid metabolism [64], vascular pathologies and aortic aneurysm [65]	**Monier et al., 2005** [7]
edl	**Transcription factor;** Transcriptional repressor, Involved in cardioblast/pericardial cell diversification	***EHF, ELF5***	N/A	**2** (1.91)	***EHF, ELF5* have not yet been associated with cardiac development;** *EHF* involved in thymus development [66], ***ELF5*** involved in mammary gland development, angiogenesis [67], early developmental stages [68]	**Schwarz et al., 2018** [69]
Egfr	**Epidermal growth factor receptor protein;** Involved in EGF/EGFR signaling and	***ERBB4,*** *EGFR*	*ERBB2, ERBB3, EPHA10, SRMS, IGF1R, PTK2, ROS1, RYK*	**13** (12.87)	***ERBB4*** involved in cardiac development including trabeculation, endocardial cushion formation, regulation of valve development including regulation of valve mesenchyme proliferation [70] [71]	**Schwarz et al., 2018** [69]

Gene	Information	Highest score Orthologue	*Additional Orthologues*	DIOPT score	Vertebrate Orthologue Information	Study (Reference)
	cardioblast/pericardial cell diversification					
Eve	**Homeodomain transcription factor;** Involved in animal body segmentation, Interacts with/Represses *Ubx, Wg*, Involved in cardiac development and cardioblast/pericardial cell diversification	***EVX2***	*EVX1, BARX2, BARHL2, GSX2, ARX, HOXC12*	**10** (10.04)	***EVX2*** is localized near the *HOX4* locus of chromosome 2 while ***EVX1*** near the *HOX1* locus on chromosome 7 [72]; Both involved in development of limbs and genitalia [73]	**Fujioka et al, 2005** [74]
fz	***Frizzled*** **segment polarity protein;** G-protein coupled receptor, Involved in Wnt signaling, Involved in nervous system development, segmentation, heart morphogenesis	***FZD1, FZD7***	***FZD2****, FZD3, FZD6, MFRP, FZD8, FZD10, FZD9, FZD5, FRZB, FZD4, SFRP2, SFRP5, CORIN, SFRP4, SMO, SFRP1*	**15** (14.87, 14.82)	***FZD1, FZD7*** are membrane receptors Frizzled, Interact with Wnt signaling factors, regulating gene transcription; Involved in induction of mesoderm development and mesoderm patterning [31], regulation of cell polarity and neural patterning	**Bhanot et al., 1999** [75], **Chen and Struhl, 1999** [76]
Gart	**Enzyme protein, part of lipid, purine metabolic biochemical pathways;** Trifunctional enzyme, Involved in lipid metabolism, feeding and lifespan regulation	***GART***	N/A	**17** (16.75)	***GART*** functions as a trifunctional enzyme (Phosphoribosylglycinamide Formyltransferase, Phosphoribosylglycinamide Synthetase, Phosphoribosylaminoimidazole Synthetase), expressed in various tissues throughout development, including the developing heart [77]	**Schroeder et al., 2019** [40]
GGPPS/qm	**Geranylgeranyl Pyrophosphate synthase protein (GGPPS), part of the Mevalonate biochemical pathway;** involved in Gγ1 geranylgeranylation and regulation of adhesion between cardioblast/pericardial cells	***GGPS1***	*PDSS2, FDPS, PDSS1*	**16** (15.72)	**GGPP**, the product of the reaction catalyzed by GGPS1, serves as an end-product for the lipidation and localization of Rho GTPases below the plasma membrane, in turn stimulating pathways that lead to mural cell recruitment alongside assembling endothelial cells [78] (**Zebrafish**)	**Yi et al., 2006** [79]
Gia	**Methuselah-like G-protein coupled receptor protein**	***ADGRF3, ADGRF4,***	N/A	**2** (1.81)	***ADGRG6*** involved in cardiac development, expressed in the endocardium [80] (**Mouse**)	**Patel et al., 2016** [81]

Gene	Information	Highest score Orthologue	*Additional Orthologues*	DIOPT score	Vertebrate Orthologue Information	Study (Reference)
	**(GPCR);** Involved in midline contralateral cardioblast adhesion and cardioblast/pericardial cell adhesion	***ADGRD1, ADGRE2, ADGRG3, ADGRG6, ADGRL1, ADGRG7, ADGRF5, ADGRD2, ADGRG2, ADGRE1, ADGRE5, ADGRG4, ADGRL4, ADGRL2, ADGRE3, ADGRL3***			***ADGRF5, ADGRL4*** both expressed in vascular endothelial cells, implicated in cardiovascular development, though specific mechanism is still unclear [82] (**Mouse**)<br><br>***ADGRL2*** involved in flow-dependent angiogenesis and vascular remodeling [83] (**Mouse**) (**Zebrafish**)<br><br>***ADGRF3, ADGRF4, ADGRD1, ADGRE2, ADGRG3, ADGRL1, ADGRG7, ADGRD2, ADGRG2, ADGRE1, ADGRE5, ADGRG4, ADGRL4, ADGRE3, ADGRL3* have not yet been associated with cardiac development;** ***ADGRF4*** associated with enamel mineralization [84], ***ADGRL1*** implicated in neurodevelopmental disorders [85], ***ADGRG7*** implicated in familial endometriosis [86], ***ADGRG2*** implicated in congenital bilateral absence of the vas deferens [87], ***ADGRL4*** involved in vascular remodeling during development [82], ***ADGRL3*** involved in neurogenesis [88]	
H15 (nmr1)	**T-box transcription factor;** Involved in cardioblast/pericardial cell diversification, **part of the core regulatory network directing heart development**	***TBX20***	*TBX18, MGA, TBX19, TBXT, TBX1, TBX22, EOMES, TBR1, TBX5, TBX10, TBX2, TBX4, TBX6, TBX15, TBX3, TBX21*	**11** (10.73)	***TBX20*** regulates development of the atrioventricular canal, early ventricular differentiation; Interacts with ***NKX2-5, GATA4/5, TBX5*** [89]	**Reim et al., 2005** [90], **Hu et al., 2011** [45]
Hand	**bHLH transcription factor;** Involved in cardiac development, somatic muscle development including wing-heart formation, Dorsal vessel remodeling during metamorphosis	***HAND2***	*HAND1, TCF23, TCF24, BHLHA9, TWIST1, TWIST2, TCF21, FERD3L, TCF15, MSC, SCX, ASCL4, TAL1, ASCL2, MSGN1, MESP1, NHLH1, FIGLA, ASCL5, TAL2, ASCL3, LYL1, ASCL1, MESP2, PTF1A*	**15** (14.74)	***HAND2*** interacts with NOTCH-dependent signaling pathways involved in the development of the endocardium, in turn regulating ventricle trabeculation, septation and maturation of the coronary vasculature and endocardium [91]	**Han et al., 2006** [92], **Lo et al., 2007** [93]
Hcf	**Histone methyltransferase, COMPASS Common subunit (Set1/COMPASS, Trx**	***HCFC1, HCFC2***	*KLHDC1, RABEPK, LZTR1, FBXO42, KLHDC4, KLHDC3*	**8** (8.14, 7.98)	***HCFC1*** is involved in the regulation of processes related to cell cycle progression and regulation, cell proliferation and cell death; *HCFC1b* regulates cardiac neural crest cell differentiation/proliferation in the	**Huang et al., 2022** [95]

Gene	Information	Highest score Orthologue	*Additional Orthologues*	DIOPT score	Vertebrate Orthologue Information	Study (Reference)
	**COMPASS-like);** Methylation at H3K4				posterior pharyngeal arch, chondrocyte progenitor development [94] (**Zebrafish**)<br><br>***HCFC2*** **has not yet been associated with cardiac development**	
Hd	**DNA replication factor;** Involved in DNA amplification in the ovary and DNA proliferation	***DONSON***	N/A	**15** (14.77)	***DONSON*** maintains genomic stability during replication by binding to and stabilizing the DNA replication fork, Activates S-phase and G2/S-phase checkpoints in case of replication stress [96]	**Schroeder et al., 2019** [40]
Hh	**Hedgehog segment polarity protein;** Involved in Hh signaling, Expressed in a striped pattern in each developing segment regulating segment boundaries, involved in limb, wing and eye development	***SHH***	*IHH, DHH*	**15** (14.79)	***SHH*** is involved in heart development particularly in regulation of second heart field (SHF) progenitor development and differentiation, regulates cardiomyocyte differentiation, endocardium development [97]	**Park et al., 1996** [98], **Liu et al., 2006** [99]
HMGCR	**HMG-CoA reductase, part of the Mevalonate biochemical pathway;** involved in Gγ1 geranylgeranylation and regulation of adhesion between cardioblast/pericardial cells	***HMGCR***	N/A	**15** (14.77)	***HMGCR*** implicated in the maintenance of vascular stability during embryonic development via the Rho GTPase pathway and participation in pathways that lead to mural cell recruitment along assembling endothelial cells [78] (**Zebrafish**)	**Yi et al., 2006** [79]
Htl	**Fibroblast growth factor (FGF)-receptor protein;** Involved in FGF/FGFR signaling and mesoderm development	***FGFR3***	*FGFR1, FGFR4, FGFR2, STYK1, MET, ROR1, EPHA10, SRMS, IGF1R, PTK2, ROS1, TEK, KDR, RET, RIPK1, FLT3, PDGFRA, ABL2, MAP3K10, KIT, PDGFRB, FER, TYK2, MST1R, FLT1, ABL1, FLT4, JAK2, FES, TXK, TIE1, MATK, JAK3, FGFRL1, CSF1R*	**15** (14.72)	There are four FGF receptor genes in vertebrates identified so far, *FGFR1, FGFR2, FGFR3, FGFR4,* all involved in non-overlapping functions during development [100]<br><br>**FGFR2B** functions as the main receptor for FGF10, **FGFR3** and **FGFR1** interact with FGF8 in pharyngeal endoderm and ectoderm, mediating migration of cardiac Neural crest cells [103]<br><br>**FGFR23** expressed in higher levels in the cardiac neural crest [103]	**Kadam et al., 2009** [101], **Dorey and Amaya, 2010** [102]

Gene	Information	Highest score Orthologue	*Additional Orthologues*	DIOPT score	Vertebrate Orthologue Information	Study (Reference)
Jarid2	**Jumonji C domain-containing protein;** Transcriptional repressor of Notch, Regulates histone methyltransferase complexes, Involved in the inhibition of *Notch1* expression; *Jarid2* expression increases with reduction in NO levels	***JARID2***	*KDM5B, KDM5A, KDM5C, KDM5D*	**14** (13.79)	***JARID2*** is a transcriptional repressor of Notch, Regulates histone methyltransferase complexes (histone demethylase activity), Interacts with the histone methylase Setdb1, Involved in cardiac development and, in particular, regulation of ventricle trabeculation [104], Expressed in the second heart field (SHF) and implicated in development of the outflow tract; ***JARID2*** expression is regulated by *NKX2.5* [105]	**Basu et al., 2017** [106]
Kif1A	**Microtubule-associated protein**	***KIF1A***	*KIF1B, KIF1C, KIF13A, KIF14, STARD9, KIF13B, KIF16B, KIF2B*	**15** (14.79)	***KIF1A*** is involved in intracellular transport and cardiac development	**Akasaka et al., 2020** [107]
Kismet	**Helicase**; Involved in chromatic remodeling and the establishment of segmentation during development	***CHD7, CHD6***	*CHD9, CHD8, CHD5, CHD4, CHD3, CHD1, CHD2*	**12** (11.85, 11.80)	Involved in chromatin remodeling, **CHD7** interacts with **BGR1**, another chromatin remodeling factor, to regulate gene expression in cardiac Neural crest cells during outflow tract development while both *CHD7* and *TBX1* are involved in aortic arch development [108], *CHD7*, *TBX1* is also involved in fourth pharyngeal arch formation, ear and thymus via expression in the pharyngeal ectoderm expression (**Mouse**)<br><br>***CHD6* has not yet been associated with cardiac development; *CHD6*** involved in skeletal muscle development [109] **(Pig)**	**Zhu et al., 2017** [34]
Kuz	**A Disintegrin and Metalloproteinase (ADAM);** Involved in the proteolytic shedding of transmembrane proteins, Notch lateral inhibition, cardiac and vascular development	***ADAM10***	*ADAMDEC1, ADAM32, ADAM28, ADAM29, ADAM2, ADAM18, ADAM21, ADAM7, ADAM30, ADAM23, ADAM19, ADAM17, ADAM20, ADAM12, ADAM11, ADAM15, ADAM9, ADAM22, ADAM8, ADAM33*	**14** (13.89)	***ADAM10*** regulates NOTCH signaling and function, regulates cardiac development, including processes such as epithelial-to-mesenchymal transition and endocardial cushion development [110], EGFR signaling, coronary vascular development and establishment of arterial cell identity [111]	**Albrecht et al., 2006** [112]
lanA	**Laminin alpha (α) subunit;** Involved in tissue invagination/movement, Morphogenesis of cardiac,	***LAMA5***	*LAMA3, LAMA4, LAMA2, CCDC158, USH2A, LAMA1, HSPG2, MEGF10, CRB1, SCARF1, MEGF6, LAMB3,*	**14** (13.87)	***LAMA5*** derived from endothelial cells regulates the expression of structural cardiomyocyte proteins, stimulates PDGFRβ signaling in nearby vascular cell populations to upregulate deposition of extracellular	**Stark et al., 1997** [114], **Nishiyama et al., 2005** [115]

Gene	Information	Highest score Orthologue	*Additional Orthologues*	DIOPT score	Vertebrate Orthologue Information	Study (Reference)
	salivary gland, trachea, midgut, ventral cord, amnioserosa tissue, Implicated in cardiac leading-edge formation and motility		*MEGF9, CCDC186, LAMC2, LAMC3, LAMC1, NTNG1*		matrix and increase cardiomyocyte contractile force, facilitates maturation of cardiac organoids [113] (**In vitro models**)	
Lid	**Histone demethylase;** Demethylation at H3K4	***KDM5A***	*KDM5B, KDM5C, KDM5D, JARID2, KDM4B, BAZ1A, KDM4A, KDM4F, KDM4D, KDM4E*	**17** (16.75)	***KDM5A*** regulates the metabolic shift characterizing the transition from immature to mature cardiac progenitors, via regulation of genes related to myofibrillar formation, oxidative phosphorylation and fatty acid oxidation, Involved in mitochondrial biogenesis [116] (**In vitro models**)	**Zhu et al., 2017** [34]
Lpt	**Histone methyltransferase;** Methylation at H3K4me	***KMT2D, KMT2C***	N/A	**8 (**7.89**)**	***KMT2D*** involved in cardiac mesoderm and anterior heart field precursor development, activation/regulation of genes associated with cardiomyocyte differentiation including genes associated with ion transport and cell cycle regulation [117] (**Mouse**)<br><br>***KMT2C*** implicated in the hypertrophic cardiac remodelling after Transverse aortic constriction [118] (**Mouse**); ***KMT2C* has not yet been associated with cardiac development; *KMT2C*** involved in neurogenesis [119]	**Huang et al., 2022** [95]
mgl	**Large LDL receptor-related protein;** Mediates receptor endocytosis, involved in dorsoventral wing patterning and cell proliferation	***LRP2***	*LRP1B, LRP1, LRP4, CD320, LDLRAD3, LRP3, LRP12, LRP10, LRP5, LRP6, HMCN1, MEGF6, FBLN7, FBN3, HMCN2, FBLN5, FBN2, FBN1, MATN2, LDLR, EGF, LRP8, SORL1, VLDLR*	**14** (14.01)	***LRP2*** involved in Wnt and Shh signaling regulation, Involved in the regulation of cardiomyocyte proliferation via a downregulation of cell cycle inhibitors and pro-apoptotic factors (TP53, CDKN1A), upregulation of cell-cycle genes (**In vitro models**)	**Theis et al., 2020** [19], **Riedel et al., 2011** [120]
mid (nmr2)	**T-box transcription factor;** Involved in cardioblast/pericardial cell diversification, **part of the core regulatory network directing heart development**	***TBX20***	*TBX18, MGA, TBX19, TBXT, TBX1, TBX22, EOMES, TBR1, TBX5, TBX10, TBX2, TBX4, TBX6, TBX15, TBX3, TBX21*	**13** (12.78)	***TBX20*** regulates development of the atrioventricular canal, early ventricular differentiation; Interacts with ***NKX2-5, GATA4/5, TBX5*** [89]	**Reim et al., 2005** [90], **Hu et al., 2011** [45]

Gene	Information	Highest score Orthologue	*Additional Orthologues*	DIOPT score	Vertebrate Orthologue Information	Study (Reference)
mmp1	**Matrix Metalloproteinase;** Regulates cardiac leading-edge activity, cardiac lumen expansion, Restricts the extent of the luminal domain and distribution of pericardin, viking proteins	***MMP14, MMP2***	*MMP24, MMP16, MMP15, MMP8, MMP1, MMP12, MMP10, MMP20, MMP27, MMP3, MMP26, MMP7, MMP13, MMP25, MMP19, MMP9, MMP11, MMP28, MMP2, MMP17, MMP21, MMP23B, ELSPBP1, VTN, HPX, PRG4*	**12** (11.9)	The interplay of matrix metalloproteinases (MMP) and tissue inhibitor of matrix metalloproteinases (TIMP) contribute to cardiac development via modulation of cell migration/proliferation along with regulation of heart tube formation, endocardial cushion development and myocardial remodeling [121]<br><br>***MMP14*** expressed/implicated in the development of the endocardium and the truncus arteriosus, may contribute to ventricular trabeculation and valve formation, tissue remodeling to promote outflow tract alignment/development, development of the vascular system including endothelial cells that promote vascularization and possibly contributing to coronary vascular development [124] **(Mouse)**<br><br>***MPP2*** regulates heart tube formation during early cardiac development; the pattern of ***MMP2*** expression parallels the expression of ***ISL2*** in anterior heart field progenitors [125] (**Chicken**)	**Raza et al., 2017** [122], **Hughes et al., 2020** [123]
mmp2	**Matrix Metalloproteinase;** Regulates cardiac leading-edge activity, cardiac lumen formation, Restricts the extent of the luminal domain and distribution of pericardin, viking proteins	***MMP15, MMP9***	*MMP14, MMP24, MMP16, MMP25, MMP7, MMP17, MMP1, MMP8, MMP11, MMP9, MMP20, MMP19, MMP2, MMP13, MMP12, MMP10, MMP27, MMP3, MMP28, MMP26, MMP21, MMP23B, ELSPBP1, VTN, PRG4, HPX*	**10** (10)	The interplay of matrix metalloproteinases (MMP) and tissue inhibitor of matrix metalloproteinases (TIMP) contribute to cardiac development via modulation of cell migration/proliferation along with regulation of heart tube formation, endocardial cushion development and myocardial remodeling [121]<br><br>***MMP15*** interacts with *Snail1,* regulates cell migration during epithelial-to-mesenchymal transition in the developing endocardial cushions, contributing to valve development; ***MMP15*** does not initiate the transition from epithelial to mesenchymal states [126] **(Mouse)**<br><br>***MMP9*** involved in cardiac development as overexpression, induced by Phenanthrene compounds (aromatic hydrocarbon), is associated with defects in cardiac looping, cardiac enlargement, thinning of ventricular myocardium [127] (**Zebrafish**)	

**Gene**	**Information**	**Highest score Orthologue**	***Additional Orthologues***	**DIOPT score**	**Vertebrate Orthologue Information**	**Study** (Reference)
Mnn1	**Histone methyltransferase, Trx COMPASS-like Unique subunit;** Methylation at H3K4, Active during Stages 16-17 of *D. melanogaster* embryonic development	***MEN1***	N/A	**14** (13.86)	Involved in regulation of cell proliferation via TGFβ signaling, hematopoiesis via effects on *HOXA*9 [129]; ***MEN1* has not yet been explicitly associated with cardiac development in non-*D. melanogaster* animal models; *MEN1*** interacts with genes involved in skeletal, liver, pancreas and blood development [130]	**Zhu et al., 2024** [26]
msh-2	**Msh-homeobox transcription factor;** many functions, participates in myogenesis, visceral mesoderm development	***MSX2***	*MSX1, NKX6-2, BARX2, BARHL2, GSX2, TLX2, EMX1, VAX1, CDX2, DLX6, DLX2, CDX4, DLX1, DLX4, DLX3, NOTO, NKX6-1, EMX2, NKX6-3, VAX2*	**12** (11.73)	***MSX2*** regulates cardiomyocyte precursor survival, outflow tract development [131]; cardiac blood vessel precursor maturation [132]	**Bodmer et al., 2011**[133], **Hu et al.,2011** [45]
Naca	**NAC α-subunit protein, part of the NAC chaperone complex;** NAC-ribosome complexes direct transport of nascent polypeptide chains to mitochondria, transport of other polypeptide chains to the endoplasmic reticulum, Implicated in Dorsal vessel remodeling (**Metamorphosis**)	***NACA***	N/A	**N/A**	***NACA*** is involved in hematopoiesis with regulation of stromal cell maturation and as a result, hematopoietic niche function [134], regulation of myofibrillar organization in skeletal muscle tissue [135] (**Zebrafish**)<br><br>***NACA*** is involved in the regulation of bone development, skeletal muscle differentiation including incorporation of myosin heavy chain proteins into sarcomeres [136] (**Mouse**)	**Schroeder et al., 2022** [13]
Netrin	**Extracellular protein (secreted);** Dscam ligand, Implicated in filopodia, lamellipodia formation in migrating cardioblasts	***NTN1***	***NTN3, NTN5, NTNG2, LAMA5, LAMC1, LAMC3, NTNG1***	**14** (13.77)	***NTN1*** is involved in axon guidance and neural development, vascular development and guidance owing to differential effects based on Netrin-1 receptor binding (UNC5B receptor repels vascular sprouting/vascularization, NCC receptor attracts vascular sprouting/vascularization); *NTN1* is expressed in the developing neural tube, trabecular myocardium, and its effects are dependent on the different Netrin-1 receptors [137] (**Mouse**)	**Raza and Jacobs, 2016** [63]
Notch	**Transmembrane receptor protein;** Interacts with *Numb*, Involved in Notch signaling, Implicated in cardioblast/pericardial cell diversification	***NOTCH1, NOTCH2, NOTCH3***	*NOTCH4, CRB1, SNED1, NOTCH2NLR, F9, NOTCH2NLC, NOTCH2NLA, CRB2, VCAN, DNER, SLIT1, MEGF10, DLL1, MEGF6, MEGF11, LAMA5, LRP2,*	**12** (11.91, 11.77, 11.67)	Notch signaling, in general, regulates vascular smooth muscle cell development, Interacts with Jagged1 to activate *NKX2.5* expression [138]<br><br>***NOTCH1b*** expression sensitive to blood flow, Implicated in valve tissue maturation (**Mouse**)	**Basu et al., 2017** [107]

Gene	Information	Highest score Orthologue	*Additional Orthologues*	DIOPT score	Vertebrate Orthologue Information	Study (Reference)
			SCARF1, FBN3, EDIL3, FBLN5, FBN2, FAT4, MATN2, JAG1, JAG2		***NOTCH1b*** regulates valve development via Notch-Delta ligand cell-to-cell interactions in response to blood flow (mechanosensitive pathway, Notch-mediated lateral inhibition) [139] (**Zebrafish**)<br>***NOTCH2, NOTCH3*** cooperate and regulate development of vascular smooth muscle tissue [140] (**Mouse**)	
Numb	**Numb protein gene;** Interacts with *Notch* (transcriptional repressor), Involved in Notch signaling	***NUMB***	*NUMBL, LDLRAP1, GULP1, FAM43A, MAPK8IP1, MAPK8IP2, NOS1AP, FAM43B, DAB1[141]*	**12** (11.93)	**NUMB** regulates Notch signaling and left-right asymmetry, Expressed in adult cardiac progenitors and distributed asymmetrically during progenitor division, Other Numb associated proteins regulate myocardial compaction and trabeculation, epicardial entry into the myocardium, septation of the outflow tract and atrioventricular canal [142]	**Basu et al., 2017** [107], **Gajewski et al., 2000** [143]
Org-1	**T-box transcription factor;** Involved in somatic mesoderm and trunk visceral mesoderm development, AM/TARM development, Interacts with *tup, Ubx,* Ecdysone (**metamorphosis**) to promote Ventral longitudinal muscle development	***TBX1***	*TBX10, TBX18, TBX5, TBX4, TBX15, MGA, TBX22, EOMES, TBR1, TBX6, TBX21, TBX2, TBX3, TBX19, TBXT, TBX20*	**12** (11.8)	***TBX1*** expressed in the in the craniopharyngeal mesoderm, directs development of local cell groups towards anterior second heart field (SHF) lineages, Interacts with Baf60a/Smarc-d1 subunit of chromatin remodelling complexes to regulate *WNT5A* expression [144] [31] (**Mouse**)	**Schaub et al., 2012** [145], **Boukhatmi et al., 2014** [146]
pnr	**GATA transcription factor;** essential for dorsal closure (epidermis), involved in the specification of cardiac cells, **part of the core regulatory network directing heart development**	***GATA4***	*GATA5, GATA6, GATA1, GATA2, GATA3, ZGLP1, TRPS1*	**12** (11.8)	***GATA4*** is an early cardiac transcription factor, interacts with *MEF2C (GATA4* cofactor*)*, Involved in cardiac, visceral and skeletal muscle development, Regulates endoderm and mesoderm development [147], development of the proepicardium, endocardial cells [148] and valves [149]; No effect on second heart field (SHF) cardiac progenitors	**Han and Olson, 2005** [52]
Ptip	**Histone methyltransferase, Trr COMPASS-like Unique subunit;** Methylation at H3K4, Active during Stages 13-14 of	***PAXIP1***	***MDC1, TOPBP1***	**11** (10.8)	***PAXIP1*** is involved in the regulation of genomic stability, chromatic condensation, may regulate cell proliferation during cardiac development, Interacts	**Zhu et al., 2024** [26]

Gene	Information	Highest score Orthologue	*Additional Orthologues*	DIOPT score	Vertebrate Orthologue Information	Study (Reference)
	D. melanogaster embryonic development; characterized by a drop in expression during Stage 14E				with gene promoters that are also regulated by *KMT2D* (*KMT2D* is also involved in cardiac development) [150]	
pygo	**Nuclear PHD-finger protein;** Involved in Wnt signaling (canonical signaling), Involved in heart valve development independent of Wnt signaling	***PYGO2***	*PYGO1*	**7** (7.01)	***PYGO2*** is involved in canonical Wnt signaling via the formation of a β-catenin/BCL9/Pygo1 or β-catenin/BCL9/Pygo2 complexes via interaction with the protein HD1, Contributes to embryonic axis formation [151] (**Frog**) (**Zebrafish**)<br><br>***PYGO2*** regulates cardiac progenitors and cardiac Neural crest cells [151] (**Zebrafish**) (**Mouse**)<br><br>***PYGO2*** is involved in signaling pathways independent of β-catenin in mammals, for example during eye lens development, spermatogenesis, enamel formation during tooth development [151] (**Mouse**)	**Tang et al., 2014** [152]
pyr	**Fibroblast growth factor 8 (FGF8)-related protein;** Involved in FGF/FGFR signaling and mesoderm development	***FGF8***	N/A	**--**	***FGF8*** is a paracrine FGF factor, Involved in embryonic mesoderm and endoderm, Involved in cardiac development including development of the anterior heart field, proliferation of second heart field (SHF) progenitors involved in development of the outflow tract and right ventricle [31], survival of migrating cardiac Neural crest cells as they migrate towards the developing outflow tract, regulation of cardiac looping [153]	**Kadam et al., 2009** [102], **Dorey and Amaya, 2010** [103]
Rbbp5	**Histone methyltransferase, COMPASS Common subunit (Set1/COMPASS, Trx COMPASS-like, Trr COMPASS-like);** Methylation at H3K4	***RBBP5***	N/A	**16** (15.8)	***RBBP5*** interacts with c-Jun, involved in DNA transcription during cardiac development, regulates/prevents cardiomyocyte fate lineage direction via regulation of H3K4 [154] **(In vitro models)**	**Zhu et al., 2024** [26]
rho	**Transmembrane protein, part of the ventrolateral group of genes;** Involved in	***RHBDL3***	*RHBDL1, RHBDL2, RHBDF2, RHBDF1, PARL*	**10** (9.76)	**RHBDL3 has not yet been associated with cardiac development;** Vertebrate *rho* ortholog involved in neurogenesis [155]	**Schwarz et al., 2018** [70]

Gene	Information	Highest score Orthologue	*Additional Orthologues*	DIOPT score	Vertebrate Orthologue Information	Study (Reference)
	cardioblast/pericardial cell diversification					
Robo	**Transmembrane protein receptor, main receptor for Slit extracellular protein;** Involved in dorsal closure (ectodermal epithelium migration), cardioblast migration and alignment via accumulation of Slit and Robo proteins in the midline, cardiac lumen formation	***ROBO3, ROBO1, ROBO2***	*ROBO4, OBSL1, HMCN1, NCAM1, HMCN2, IGDCC3*	**10** (9.68, 9.67, 9.62)	**SLIT/ROBO** signaling involved in cell-to-cell adhesion during cardiac cell polarization, migration, lumen development [156] (**Zebrafish**)	**Qian et al., 2005b** [157], **MacMullin and Jacobs, 2006** [158], **Medioni et al., 2008** [48], **Santiago-Martínez et al., 2008** [159], **Zmojdzian et al., 2008** [160], **Zmojdzian et al., 2018** [161], **Raza and Jacobs, 2016** [63]
					**SLIT/ROBO** signaling involved in regulation of chamber development, migration of cardiac Neural crest cells, development of the cardiac outflow tract, atrioventricular endocardial cushions [156], pericardium and venous return systems [162]; functions dependent on the regulation of NOTCH signaling, SLIT/ROBO signaling in turn regulated by ***TBX1*** [156] (**Mouse**)	
					ROBO1, only, can interact with the Neuropilin1 receptor, required for development of the outflow tract endocardial cushions, membranous ventricular septum, expressed in cardiac Neural crest cells [156]	
					ROBO2 expressed in cardiac Neural crest cells [156] and Second heart field (SHF) cardiac progenitors [163] (**Mouse**)	
Robo2	**Transmembrane protein receptor, main receptor for Slit extracellular protein;** Involved in dorsal closure (ectodermal epithelium migration), cardioblast migration and alignment via accumulation of Slit and Robo proteins in the midline, cardiac lumen formation	***ROBO1, ROBO3***	*ROBO2, ROBO4, IGSF9, IGDCC3*	**9** (8.77, 8.68)	**SLIT/ROBO** signaling involved in cell-to-cell adhesion during cardiac cell polarization, migration, lumen development [156] (**Zebrafish**)	
					**SLIT/ROBO** signaling involved in regulation of chamber development, migration of cardiac Neural crest cells, development of the cardiac outflow tract, atrioventricular endocardial cushions [156], pericardium and venous return systems [162]; functions dependent on the regulation of NOTCH signaling, SLIT/ROBO signaling in turn regulated by ***TBX1*** [156] (**Mouse**)	

Gene	Information	Highest score Orthologue	*Additional Orthologues*	DIOPT score	Vertebrate Orthologue Information	Study (Reference)
					***ROBO3* expression mainly detected in the central nervous system, has not yet been associated with cardiac development** [163]	
RpL13	**Large ribosomal subunit protein eL13 gene;** Ribosomal protein (Large ribosomal subunit), Involved in proteostasis	***RPL13***	N/A	**16 (**15.8)	***RPL13*** is involved in the upregulation of genes related to cell-cycle progression (particularly during the S, G2 phases), involved in cardiac progenitor proliferation/differentiation and cardiomyocyte proliferation (**In vitro models**)	**Schroeder et al., 2019** [40]
RpL14	**Large ribosomal subunit L14 protein;** Involved in proteostasis	***RPL14***	N/A	**16 (**15.8**)**	***RPL14*** is involved in proteostasis, Expressed in various tissues including the embryonic heart, brain, liver and limbs	**Nim et al., 2021** [164]
Rpn8	**Regulatory component protein, 26S Ubiquitin-proteasome complex;** Regulatory component of the 26S Ubiquitin-proteasome complex, Involved in proteostasis	***PSMD7***	*MYSM1, BRCC3, EIF3F, STAMBPL1, STAMBP, COPS5, PSMD14, COPS6*	**16** (15.79)	***PSMD7*** is involved in proteostasis, Expressed in various tissues including the embryonic heart, brain, liver and limbs	**Nim et al., 2021** [164]
RpS24	**Small ribosomal subunit S24 protein;** Involved in proteostasis	***RPS24***	N/A	**16** (15.8)	***RPS24*** is involved in proteostasis, Expressed in various tissues including the embryonic heart, brain, liver and limbs	**Nim et al., 2021** [164]
Scny	**Ubiquitin protease;** Deubiquitylation at H2BK120	***USP36***	*USP42, USP17L7, USP17L1, USP17L20, USP17L18, USP17L25, USP17L11, USP17L12, USP17L22, USP17L3, USP17L8, USP17L13, USP17L19, USP17L21, USP17L5, USP17L4, USP17L10, USP17L17, USP17L2, USP17L15, USP17L6P, USP17L28, USP17L29, USP17L27, USP17L30, USP17L26, USP17L24,*	**11** (10.89)	**USP36** is an intracellular signal transduction protein activated in the context of TGFβ signaling, implicated in various cardiovascular processes [165] [166]; ***USP36* has not yet been associated with cardiac development;** *USP36* involved in early embryonic development [167] (**Mouse**)	**Zhu et al., 2017** [34]

Gene	Information	Highest score Orthologue	*Additional Orthologues*	DIOPT score	Vertebrate Orthologue Information	Study (Reference)
			USP17L23, USP49, USP44, USP27X, USP3			
Scr	**Homeobox containing (Hox) gene, part of the Antennapedia Complex (ANTP-C) of genes;** Expressed in segment T1, determines the identity of anterior thoracic structures, Labial structures (proboscis) in the *D. melanogaster* embryo	***HOXB5, HOXA5***	*HOXD4, HOXA4, HOXB4, HOXC4, HOXC5, HOXB6, HOXD8, HOXA7, HOXB8, HOXD9, HOXC6, HOXA6, HOXB7, HOXC8, HOXD3, PDX1, HOXD13, HOXB3, HOXC9, HOXA9, NKX6-2, HOXB9, HOXD11, HOXD1, MEOX1, HOXA2, HOXB1, GSX1, GSX2, MEOX2, GBX1, HOXB2, GBX2, MNX1, EMX1, CDX2, BARX2, HOXA11, CDX4, HOXC11, DLX4, RAX, HOXD10, DBX2, CDX1, DBX1, TLX2, HOXC12, HOXA10, NKX6-1, RAX2, EMX2, NKX6-3, HOXA1, HOXA3*	**9** (8.8)	In general, anterior *HOX* groups (*HOX1-HOX9*) are homologous to *D. melanogaster ANTP-C* and *Abd-A* [1]<br>*HOXA/HOXB* cluster evolution associated with diversification in cardiac form and emergence of complex cardiac shapes including cardiac looping [9]<br>***HOXA5*** involved in aortic arch development [11]<br>***HOXB5*** involved in vascular endothelial cell differentiation [11]	**Perrin et al., 2004** [5]
Set1	**Histone methyltransferase, Set1/COMPASS Unique Core subunit;** Methylation at H3K36me, Characterized by steady expression throughout development	***SETD1A***	*SETD1B, ASH1L, KMT2D, SETD2, NSD2, NSD3, KMT2C, NSD1, KMT2B*	**12** (11.85)	***SETD1A* has not yet been associated with cardiac development** [168]; ***SETD1A*** involved in hematopoiesis [169]	**J. Zhu et al., 2023** [24]
Set2	**Histone methyltransferase;** Methylation at H3K36, Characterized by steady expression throughout development	***SETD2***	*ASH1L, NSD2, NSD3, NSD1, KMT2D, SETD1B, SETD1A, KMT2C, EHMT1, EZH1, EZH2, SUV39H2, EHMT2, SETDB2, KMT2B, KMT2A, SETDB1*	**13** (12.64)	***SETD2*** is involved in early cardiac development, including coronary vascular development, ventricular myocardium development and compaction; also contributes to adult cardiac development/function [170] (**Mouse**)	**J. Zhu et al., 2023** [24]
Shg	**Shotgun protein (DE-Cadherin) gene;** Involved in cardiac lumen formation along with Slit, Robo proteins, adhesive effects between contralateral cardioblasts	*CELSR1, CELSR3, CELSR2*	N/A	**2** (2.01, 2.01, 2.01)	***CELSR1, CELSR2, CELSR3*** stimulate the planar cell polarity pathway (non-canonical WNT signaling), regulate convergence and extension group cell movements during embryonic development, cell migration during cardiac development [171] [172]	**Santiago-Martínez et al., 2008** [159], **Zmojdzian et al., 2008** [160], **Zmojdzian et al., 2018** [161]

Gene	Information	Highest score Orthologue	*Additional Orthologues*	DIOPT score	Vertebrate Orthologue Information	Study (Reference)
Sli	**Extracellular protein (secreted), main ligand for Robo receptor protein;** Involved in dorsal closure (ectodermal epithelium migration), cardioblast migration and alignment via accumulation of Slit and Robo proteins in the midline, cardiac lumen formation	***SLIT1, SLIT2, SLIT3***	*LRTM2, LRTM1, LRRC3B, LRRC3, NYX, LRRC3C, LGR6, MEGF10, CHADL, NOTCH2, TLR7, NOTCH3, MEGF11, RTN4RL1, LAMA5, SLITRK4, NOTCH1, ADGRA3*	**15** (14.87, 14.87, 14.82)	**SLIT/ROBO** signaling involved in cell-to-cell adhesion during cardiac cell polarization, migration, lumen development [156] (**Zebrafish**)<br><br>**SLIT/ROBO** signaling involved in regulation of chamber development, migration of cardiac Neural crest cells, development of the cardiac outflow tract, atrioventricular endocardial cushions [156], pericardium and venous return systems [162]; functions dependent on the regulation of NOTCH signaling, SLIT/ROBO signaling in turn regulated by ***TBX1*** [156] (**Mouse**)<br><br>***SLIT1*** expression detected in atrial myocardium, pharyngeal arch endoderm and ectoderm [163]<br><br>**SLIT2** is the preferred ligand for ROBO2, expressed in the endocardium overlying the atrioventricular canal valves [162], endoderm adjacent the cardiac Neural crest cells [156] (**Mouse**)<br><br>**SLIT3** is the preferred ligand for ROBO1, predominant ligand expressed in the cardiac chambers, expressed only in the myocardium adjacent to the atrioventricular canal valves, expressed in the cardiac Neural crest cells [162] [156]; function mostly relevant to vascular development, early chamber formation requires repression of *SLIT3* by ***NKX2.5*** and ***TBX2*** [163] (**Mouse**)	**Qian et al., 2005b** [157], **MacMullin and Jacobs, 2006** [158], **Medioni et al., 2008** [48], **Santiago-Martínez et al., 2008** [159], **Zmojdzian et al., 2008** [160], **Zmojdzian et al., 2018** [161], **Raza and Jacobs, 2016** [63]
Smox	**Chromatin binding protein;** Binds chromatin at H3K27	***SMAD3***	*SMAD2, SMAD9, SMAD5, SMAD1, SMAD7, SMAD6, SMAD4*	**14** (13.87)	***SMAD3*** is an intracellular signal transduction protein activated in the context of TGFβ signaling, implicated in cardiac fibrosis and remodeling processes [173]; ***SMAD3* has not yet been associated with cardiac development;** *SMAD* involved in multiple processes, including skeletal, testicular development [174]	**Zhu et al., 2017** [34]
Son	**RNA-binding protein;** Protects nascent poplypeptides from	***SON***	N/A	**9** (8.77)	***SON*** involved in the upregulation of genes related to cell-cycle progression (particularly during the S, G2 phases), involved in cardiac progenitor	**Schroeder et al., 2019** [40]

Gene	Information	Highest score Orthologue	*Additional Orthologues*	DIOPT score	Vertebrate Orthologue Information	Study (Reference)
	degradation, Involved in cell cycle/apoptosis regulation, TGFβ and Wnt signaling as well as Integrin-mediated adhesion				proliferation/differentiation and cardiomyocyte proliferation (**In vitro models**)	
Spdo	**Actin/Tropomyosin-associated protein (tropomodulin homolog);** Regulates Notch signaling, involved in cardioblast/pericardial cell diversification	N/A	N/A	N/A	N/A	**Gajewski et al., 2000** [143]
Src42A	**Src kinase protein;** Involved in germband elongation [175], regulation of cell-to-cell adhesion, amnioserosa apoptosis	***FRK***	*YES1, FYN, SRC, FGR, HCK, SRMS, LYN, BLK, LCK, PTK6, SLA, BTK, TEC, ITK, BMX, TXK, GRB2, ABL2, ROR1, GRAP, IGF1R, ROS1, CSK, MATK, FER, PTPN6, EPHA10, GRB14, GRAP2, ABL1, GRAPL, FES, PTK2*	**13** (12.95)	***FRK* has not yet been associated with cardiac development**	**Vanderploeg and Jacobs, 2017** [176]
svp	**COUP-TF transcription factor;** Involved in cardioblast/pericardial cell diversification, **part of the core regulatory network directing heart development**	***NR2F2***	*NR2F1, NR2F6, RXRA, NR2E3, RXRG, RXRB, NR2E1, ESRRB, NR2C2*	**13** (12.76)	***NR2F2*** regulates epithelial-to-mesenchymal transition during cardiac development, vein and lymphatic vessel phenotypes, coronary vessel development [177], atrial tissue identity [178]	**Lo and Frasch, 2001** [179], **Hu et al., 2011** [45]
ths	**Fibroblast growth factor 8 (FGF8)-related protein;** Involved in FGF/FGFR signaling and mesoderm development	***FGF8***	N/A	**1** (0.9)	***FGF8*** is a paracrine FGF factor, Involved in embryonic mesoderm and endoderm, Involved in cardiac development including development of the anterior heart field, proliferation of second heart field (SHF) progenitors involved in development of the outflow tract and right ventricle [31], survival of migrating cardiac Neural crest cells as they migrate towards the developing outflow tract, regulation of cardiac looping [153]	**Kadam et al., 2009** [102], **Dorey and Amaya, 2010** [103]

Gene	Information	Highest score Orthologue	*Additional Orthologues*	DIOPT score	Vertebrate Orthologue Information	Study (Reference)
timp	**Tissue-inhibitor of metalloproteinase;** Involved in regulation of mmp1, mmp2 activity, extracellular matrix turnover, Regulates AM attachment to the Dorsal vessel	***TIMP3, TIMP2***	*TIMP2, TIMP1, TIMP4*	**15** (14.8)	The interplay of matrix metalloproteinases (MMP) and tissue inhibitor of matrix metalloproteinases (TIMP) contribute to cardiac development via modulation of cell migration/proliferation along with regulation of heart tube formation, endocardial cushion development and myocardial remodeling [122]	**Hughes et al., 2020** [124]
					TIMP2, TIMP3 expressed in endocardial tissue before and during endocardial cushion development; *TIMP2* expressed in both outflow tract and atrioventricular canal cushions throughout development, associated with continuous endocardial cushion development [122] (**Chicken**), Expressed during cardiac Neural crest cell migration [126] (**Chicken**)	
					TIMP3 expression enriched in areas where myocardial remodeling occurs, including the atrioventricular canal and ventricular trabeculae, associated with realignment of the atrioventricular canal outflow tract with the developing ventricles [122] (**Chicken**)	
					TIMP3 expression in epicardial cells regulated by EZH2, facilitates epicardial cell migration [180] **(Mouse)**	
tin	**Nk2-homeobox transcription factor;** essential for cardiac mesoderm specification, **part of the core regulatory network directing heart development**	***NKX2-5***	*NKX2-3, NKX2-2, HOXD13, NKX2-6, MSX2, TLX2, HOXC12, MSX1, NKX2-1, NKX2-4, NKX2-8, NKX3-2, NKX3-1*	**5** (4.87)	***NKX2-5*** regulates cardiac development, acts synergistically with other early cardiac transcription factors; not essential for early cardiac mesoderm specification (as opposed to *tin* in *D. melanogaster) [181]*	**Bodmer et al., 1992** [182], **Hu et al., 2011** [45], **Yin and Frasch, 1998** [183]
Tkv	**Receptor protein serine/threonine kinase;** Type I receptor for Dpp morphogen, Involved in Dpp (BMP) signaling, mesoderm migration along the overlying ectoderm, dorsoventral patterning, cardiac and	***BMPR1B,*** *BMPR1A*	*BMPR1A, ACVR1C, ACVR1B, TGFBR1, ACVR1, ACVRL1, ACVR2A*	**14** (13.74)	BMP signaling is involved in gastrulation and development of the primitive mesoderm, formation of the first heart field (FHF) and the cardiac crescents via the receptor **BMPR1A**, development of the endocardial cushions in the atrioventricular canal and the outflow tract [31]; ***BMPR1B* has not yet been associated with cardiac development; *BMPR1B*** involved in ovarian/follicular development [184]	**Yin and Frasch, 1998** [183]

Gene	Information	Highest score Orthologue	*Additional Orthologues*	DIOPT score	Vertebrate Orthologue Information	Study (Reference)
	visceral mesoderm development					
Trr	**Histone methyltransferase, Trr COMPASS-like Unique Core subunit;** Methylation at H3K4, Characterized by a drop in expression towards later developmental stages (Stage 16)	***KMT2C***	*KMT2D, KMT2B, NSD2, NSD1, KMT2A, ASH1L, SETD1A, NSD3, SETD1B, SETD2, EZH1, SETDB1, TBRG1*	**12** (11.73)	Implicated in cardiac development, interacts with *TBX1* [185] (**Mouse**)	**J. Zhu et al., 2023** [24], **Huang et al., 2022** [96]
Trx	**Histone methyltransferase, Trx COMPASS-like Unique Core subunit;** Methylation at H3K36, Characterized by steady expression throughout *D. melanogaster* development	***KMT2A***	*KMT2B, TCF20, PHF11, G2E3, PHF6, PHF7*	**13** (12.84)	***KMT2A*** is involved in development of the axial mesoderm, hematopoietic system (**Mouse**)	**Zhu et al., 2017** [34], **J. Zhu et al., 2023** [24]
tup	**LIM homeodomain transcription factor;** Involved in early cardiac development, cardioblast, pericardial cell diversification, Interacts with/Activates *Hand, srp, Odd*, Regulates *tin, doc, pnr* expression, Involved in AM/TARM development, Expressed in valve cells, **part of the core regulatory network directing heart development**	***ISL1, ISL2***	N/A	**16** (15.8, 15.75)	***ISL1*** is expressed transiently during early development as second heart field (SHF) progenitors appear and expand, involved in development of the distal outflow tract, atrial septum, sinoatrial node, atrioventricular node [186], endothelial and vascular smooth muscle cell groups (coronary vasculature)<br><br>***ISL2*** implicated in development of the anterior second heart field (SHF), with *ISL2a* involved in cardiac looping and *ISL2b* involved in the development of the arterial pole [188]	**Tao et al., 2007** [187]
UbcD6	**Ubiquitin ligase E2;** Ubiquitinates at H2BK120ub	***UBE2B***	*UBE2A, UBE2U, UBE2O, CDC34*	**15** (14.8)	***UBE2B* is involved in Chromatin remodeling [Histone 2B monoubiquitylation (H2Bub1)] by associating with E3 ubiquitin RNF20, RNF40 via formation of the H2Bub1-deposition complex**; The complex facilitates differentiation of cardiomyocytes from induced Pluripotent stem cell (iPSC) populations [33] (**In vitro models**)	**Zhu et al., 2017** [34]

Gene	Information	Highest score Orthologue	*Additional Orthologues*	DIOPT score	Vertebrate Orthologue Information	Study (Reference)
					***UBE2B* is involved in Chromatin remodeling [Histone 2B monoubiquitylation (H2Bub1)] by associating with E3 ubiquitin RNF20, RNF40 via formation of the H2Bub1-deposition complex**; The complex regulates sarcomere structure and development of the ventricular myocardium [33], Left-right patterning and ciliogenesis (**Mouse**)	
Ubx	**Homeobox containing (Hox) gene, part of the Bithorax Complex (BX-C) of genes;** Expressed in segments T3, A1-A5, determines the identity of the Anterior Dorsal vessel (**aorta**) in the *D. melanogaster* embryo, Implicated in AM development in the same location	***HOXB6, HOXC6, HOXC5, HOXA7, HOXB7, HOXB5, HOXA5, HOXD4, HOXA4, HOXB4, HOXC4***	*HOXD13, HOXA6, HOXA2, HOXD8, HOXC8, HOXB8, HOXB2, MNX1, HOXC9, HOXA9, NKX6-2, HOXB9, HOXC12, HOXA10, HOXD9, GSX2, HOXD1, MEOX1, HOXB1, GSX1, HOXD3, GBX1, PDX1, HOXA3, GBX2*	**4** (3.91, 3.91, 3.91, 3.91, 3.91, 3.91, 3.91, 3.81, 3.81, 3.81, 3.81)	In general, posterior *HOX* groups (*HOX9-HOX13*) are homologous to *D. melanogaster Abd-B* [1]	**Lo et al., 2002** [2], **Lovato et al., 2002** [3], **Ponzielli et al., 2002** [4], **Perrin et al., 2004** [5], **Monier et al., 2005** [7], **Ryan et al., 2005** [6], **LaBeau et al., 2009** [8]
					HOXA/HOXB cluster evolution associated with diversification in cardiac form and emergence of complex cardiac shapes including cardiac looping [9]	
					HOXA4 expression upregulated in cardiogenic tissues when treated with retinoic acid, leading to cardiomyocyte fate lineage restriction [189] (**In vitro models**)	
					HOXA5 involved in aortic arch development [11]	
					HOXB4, HOXC4 expressed in cardiac progenitor cell lines derived from the sinus venosus; *HOXB4* expression increases in response to miR-23b [190] (**In vitro models**)	
					HOXB5 involved in vascular endothelial cell differentiation [11]	
					HOXB7, HOXC6, HOXC8 involved in vascular smooth muscle cell differentiation and proliferation [11]	
					HOXD4 associated with early cardiac development [189] (**Chicken**)	

Gene	Information	Highest score Orthologue	*Additional Orthologues*	DIOPT score	Vertebrate Orthologue Information	Study (Reference)
					HOXA5, HOXB6, HOXC6 may correlate with vascular smooth muscle cell de-differentiation in thoracic aortic dissection [10] (**Human**)	
					HOXA7 **has not yet been associated with cardiac development** [11]; ***HOXA7*** downregulated in animal models of aortic valve stenosis sclerostin KO, though ***HOXA7*** role in aortic valve stenosis questionable [18] (**Mouse**)	
					HOXC5 **have not yet been associated with cardiac development;** *HOXC5* involved in lung mesenchyme and regulate myofibroblast adhesion [191] (**Mouse**), regulation of fibroblast assembly during dermal papilla development [192] (**Human**)	
Vegf	**Vascular endothelial growth factor;** Involved in VEGF/VEFGR/PVR signaling, Involved in hemocyte migration and survival as these accompany migrating cardiac progenitors to the midline	***PDGFA***	*PDGFB, VEGFA, PGF, VEGFD, VEGFB, PDGFC, PDGFD, VEGFC*	**9** (8.83)	***PDGFA*** interacts with PDGFRα homodimers, regulates cell proliferation in epicardial mesothelial cells, embryonic epicardium [193] (**Rat**), Involved in atrioventricular valve development including mitral valve development [194] (**Mouse**)	**Wu and Sato, 2008** [195]
Wdr82	**Histone methyltransferase, Set1/COMPASS Unique subunit;** Methylation at H3K4, Active during Stages 13-14 of *D. melanogaster* embryonic development	***WDR82***	N/A	**16** (15.8)	***WDR82*** **has not yet been explicitly associated with cardiac development in other animal models;** ***WDR82*** associated with early embryonic development [196] (**Mouse**)	**Zhu et al., 2024** [26]
Wds	**Histone acetyltransferase, COMPASS Common subunit (Set1/COMPASS, Trx COMPASS-like, Trr COMPASS-like);** Histone acetyltransferase at H3K4,	***WDR5***	*WDR5B, WDR86, WDSUB1, WDR13, WSB2, WDR38, WDR27, PAAF1, AHI1, WDR88, TBL3, PLRG1, DAW1, TBL1Y, TBL1X, PAFAH1B1, POC1A, TBL1XR1, FBXW2,*	**16** (15.75)	***WDR5*** is involved in Chromatin regulation, Regulation of factors that modulate WNT signaling, Left-right patterning and ciliogenesis [197] (**Frog**)	**Zhu et al., 2017** [34], **Zhao et al., 2023** [198]

Gene	Information	Highest score Orthologue	*Additional Orthologues*	DIOPT score	Vertebrate Orthologue Information	Study (Reference)
	Involved in Lipid homeostasis during development		*ATG16L2, TAF5, DCAF11, CFAP52, WDR49, NUP37*			
Wg	**Wingless segment polarity protein;** Involved in Wnt signaling, Expressed in the overlying ectoderm in a segmental pattern, Involved in nervous system development, segmentation and heart morphogenesis	***WNT1***	*WNT4, WNT5A, WNT3, WNT8A, WNT6, WNT5B, WNT2, WNT11, WNT3A, WNT9A, WNT10B, WNT7A, WNT7B, WNT2B, WNT10A, WNT8B, WNT16, WNT9B*	**15** (14.7)	There are 19 Wnt proteins in higher vertebrates, many of which are implicated in cardiac development [199]<br><br>***WNT1*** encodes for Wnt1 that mainly functions via the canonical Wnt signaling pathway, by binding at the membrane receptor Frizzled2 (*FZD2*) or Frizzled (*FZD*) and LRP5/6 and regulating gene transcription; Involved in induction of mesoderm development and mesoderm patterning [31], regulation of cell polarity, neural patterning	**Wu et al., 1995** [200], **Lockwood and Bodmer, 2002** [55]
DWnt4, Wnt4	**Drosophila Wnt4 homologue segment polarity protein;** Involved in heart development including ostia development via the planar cell polarity pathway (non-canonical Wnt signaling)	***WNT9B***	*WNT9A, WNT11, WNT1, WNT6, WNT2, WNT2B, WNT16, WNT10B, WNT7A, WNT7B, WNT8A, WNT5A, WNT4, WNT5B, WNT10A, WNT8B, WNT3, WNT3A*	**8** (7.76)	***WNT9B*** protein involved in both canonical and non-canonical Wnt signaling, implicated in the final steps of valve tissue maturation, Interacts with KLF2a/b in response to blow flow changes to regulate valve development via a mechanosensitive pathway [139] [201] (**Mouse**) (**Zebrafish**)<br><br>***WNT9A/WNT9B*** direct heart tube development [205] (**Zebrafish**)	**Tauc et al., 2012** [202], **Graba et al., 1995** [203], **Chen et al., 2016** [204]
wun	**Lipid phosphate phosphatase (LPP) protein;** Involved in cardiac leading-edge motility maintaining ectoderm leading edge migration alongside the cardiac leading-edge	***PLPP1***	*PLPP3, PLPP2, PLPP4, PLPP5, DOLPP1, PLPPR2, PLPPR5, PLPPR4, PLPPR1, PLPPR3*	**17** (16.75)	***PLPP1 has* not yet been associated with cardiac development**<br><br>***PLPP3*** implicated in vascular development, aortic stenosis (calcific) [207]	**Haack et al., 2014** [206]
wun2	**Lipid phosphate phosphatase (LPP) protein;** Involved in cardiac leading-edge motility maintaining ectoderm leading edge migration alongside the cardiac leading-edge	***PLPP3, PLPP1***	***PLPP2, PLPP5, PLPP4, PLPPR2, PLPPR5, PLPPR4, PLPPR1, PLPPR3***	**15** (14.8)	*PLPP3* may be involved in cardiac and vascular development; *PLPP3 is* expressed in the dorsal region of the developing heart, endocardial cushions of the atrioventricular canal and contributes to formation of developing valves, as well as truncus arteriosus [208] **(Mouse), *PLPP3*** also implicated in vascular development, aortic stenosis (calcific) [207]	

Gene	Information	Highest score Orthologue	*Additional Orthologues*	DIOPT score	Vertebrate Orthologue Information	Study (Reference)
					PLPP1 has **not yet been associated with cardiac development**	
αPS3 (scb)	***αPS3*** **integrin subunit, corresponding gene also known as scab;** *αPS3* associates with *βPS*, Involved in tissue invagination/movement, Morphogenesis of cardiac, salivary gland, trachea, midgut, ventral cord, amnioserosa tissue, Implicated in cardiac leading-edge formation and motility	***ITGA4,*** *ITGA5*	*ITGA9, ITGAL, ITGAM, ITGAE, ITGA2, ITGAD, ITGA11, ITGA1, ITGA10, ITGAX, ITGA8, ITGA6, ITGA2B, ITGAV, ITGA3, ITGA5, ITGA7*	**7** (6.76)	***ITGA4*** is a fibronectin receptor involved in the focal adhesion pathway [209]; Functions redundantly with ***ITGA5*** in zebrafish cardiac development, involved in endocardial development and differentiation, expressed in the endoderm and both developing endocardium/myocardium contributing to endocardial, myocardial migration towards the midline; *ITGA4* mostly functions in cases where *ITGA5* function is compromised [210] (**Zebrafish**)<br><br>***ITGA4*** expressed in pericytes and presumptive vascular smooth muscle cells during development facilitating their distribution/migration surrounding developing blood vessels, Involved in coronary vascular development, epicardial development [213] (**Mouse**)	**Stark et al., 1997** [115], **Moreira et al., 2013** [211], **Vanderploeg et al., 2012** [212]
βPS (mys)	***βPS*** **integrin subunit, corresponding gene also known as myospheroid;** *αPS3* associates with *βPS,* Involved in tissue invagination/movement, Morphogenesis of cardiac, salivary gland, trachea, midgut, ventral cord, amnioserosa tissue, Implicated in cardiac leading-edge formation and motility	***ITGB1***	*ITGB7, ITGB2, ITGB3, ITGB6, ITGB5, ITGB8, ITGB4, ITGBL1*	**16** (15.82)	***ITGB1*** involved in the regulation of cardiac development and cardiac maturation during late embryonic development [214]; Interacts with actin, γ-SAG, FLNC and associated factors in cardiomyocytes to form a bridge between actin filaments and the extracellular matrix, contributing/facilitating physiological tissue structure during cardiac development [215]	**Stark et al., 1997** [115], **Moreira et al., 2013** [211], **Vanderploeg et al., 2012** [212]

References

1. Behrens, A.N.; Iacovino, M.; Lohr, J.L.; Ren, Y.; Zierold, C.; Harvey, R.P.; Kyba, M.; Garry, D.J.; Martin, C.M. Nkx2-5 Mediates Differential Cardiac Differentiation through Interaction with Hoxa10. *Stem Cells Dev* **2013**, *22*, 2211–2220, doi:10.1089/scd.2012.0611.
2. Lo, P.C.H.; Skeath, J.B.; Gajewski, K.; Schulz, R.A.; Frasch, M. Homeotic Genes Autonomously Specify the Anteroposterior Subdivision of the *Drosophila* Dorsal Vessel into Aorta and Heart. *Developmental Biology* **2002**, *251*, 307–319, doi:10.1006/dbio.2002.0839.
3. Lovato, T.L.; Nguyen, T.P.; Molina, M.R.; Cripps, R.M. The Hox Gene Abdominal-A Specifies Heart Cell Fate in theDrosophila Dorsal Vessel. *Development* **2002**, *129*, 5019–5027, doi:10.1242/dev.129.21.5019.
4. Ponzielli, R.; Astier, M.; Chartier, A.; Gallet, A.; Thérond, P.; Sémériva, M. Heart Tube Patterning in Drosophila Requires Integration of Axial and Segmental Information Provided by the Bithorax Complex Genes and Hedgehog Signaling. *Development* **2002**, *129*, 4509–4521, doi:10.1242/dev.129.19.4509.
5. Perrin, L.; Monier, B.; Ponzielli, R.; Astier, M.; Semeriva, M. *Drosophila* Cardiac Tube Organogenesis Requires Multiple Phases of Hox Activity. *Developmental Biology* **2004**, *272*, 419–431, doi:10.1016/j.ydbio.2004.04.036.
6. Ryan, K.M.; Hoshizaki, D.K.; Cripps, R.M. Homeotic Selector Genes Control the Patterning of *Seven-up* Expressing Cells in the *Drosophila* Dorsal Vessel. *Mechanisms of Development* **2005**, *122*, 1023–1033, doi:10.1016/j.mod.2005.04.007.
7. Monier, B.; Astier, M.; Sémériva, M.; Perrin, L. Steroid-Dependent Modification of Hox Function Drives Myocyte Reprogramming in the Drosophila Heart. *Development* **2005**, *132*, 5283–5293, doi:10.1242/dev.02091.
8. LaBeau, E.M.; Trujillo, D.L.; Cripps, R.M. Bithorax Complex Genes Control Alary Muscle Patterning along the Cardiac Tube of *Drosophila*. *Mechanisms of Development* **2009**, *126*, 478–486, doi:10.1016/j.mod.2009.01.001.
9. Soshnikova, N.; Dewaele, R.; Janvier, P.; Krumlauf, R.; Duboule, D. Duplications of Hox Gene Clusters and the Emergence of Vertebrates. *Developmental Biology* **2013**, *378*, 194–199, doi:10.1016/j.ydbio.2013.03.004.
10. Liu, P.; Zhang, J.; Du, D.; Zhang, D.; Jin, Z.; Qiu, W.; Zhou, X.; Dong, S.; Zhou, M.; Zhao, H.; et al. Altered DNA Methylation Pattern Reveals Epigenetic Regulation of Hox Genes in Thoracic Aortic Dissection and Serves as a Biomarker in Disease Diagnosis. *Clinical Epigenetics* **2021**, *13*, 124, doi:10.1186/s13148-021-01110-9.
11. Zhou, Y.; Wu, Q.; Guo, Y. Deciphering the Emerging Landscape of HOX Genes in Cardiovascular Biology, Atherosclerosis and beyond (Review). *International Journal of Molecular Medicine* **2024**, *53*, 1–12, doi:10.3892/ijmm.2023.5341.
12. Karpe, F.; Pinnick, K.E. Biology of Upper-Body and Lower-Body Adipose Tissue--Link to Whole-Body Phenotypes. *Nat Rev Endocrinol* **2015**, *11*, 90–100, doi:10.1038/nrendo.2014.185.
13. Schroeder, A.M.; Nielsen, T.; Lynott, M.; Vogler, G.; Colas, A.R.; Bodmer, R. Nascent Polypeptide-Associated Complex and Signal Recognition Particle Have Cardiac-Specific Roles in Heart Development and Remodeling. *PLOS Genetics* **2022**, *18*, e1010448, doi:10.1371/journal.pgen.1010448.
14. Tischfield, M.A.; Bosley, T.M.; Salih, M.A.M.; Alorainy, I.A.; Sener, E.C.; Nester, M.J.; Oystreck, D.T.; Chan, W.-M.; Andrews, C.; Erickson, R.P.; et al. Homozygous HOXA1 Mutations Disrupt Human Brainstem, Inner Ear, Cardiovascular and Cognitive Development. *Nat Genet* **2005**, *37*, 1035–1037, doi:10.1038/ng1636.
15. Makki, N.; Capecchi, M.R. Cardiovascular Defects in a Mouse Model of HOXA1 Syndrome. *Human Molecular Genetics* **2012**, *21*, 26–31, doi:10.1093/hmg/ddr434.
16. Roux, M.; Laforest, B.; Capecchi, M.; Bertrand, N.; Zaffran, S. *Hoxb1* Regulates Proliferation and Differentiation of Second Heart Field Progenitors in Pharyngeal Mesoderm and Genetically Interacts with *Hoxa1* during Cardiac Outflow Tract Development. *Developmental Biology* **2015**, *406*, 247–258, doi:10.1016/j.ydbio.2015.08.015.
17. Diman, N.Y.S.-G.; Remacle, S.; Bertrand, N.; Picard, J.J.; Zaffran, S.; Rezsohazy, R. A Retinoic Acid Responsive Hoxa3 Transgene Expressed in Embryonic Pharyngeal Endoderm, Cardiac Neural Crest and a Subdomain of the Second Heart Field. *PLoS One* **2011**, *6*, e27624, doi:10.1371/journal.pone.0027624.
18. Joll, J.E.; Riley, L.A.; Bersi, M.R.; Nyman, J.S.; Merryman, W.D. Sclerostin Ablation Prevents Aortic Valve Stenosis in Mice. *Am J Physiol Heart Circ Physiol* **2022**, *323*, H1037–H1047, doi:10.1152/ajpheart.00355.2022.
19. Theis, J.L.; Vogler, G.; Missinato, M.A.; Li, X.; Nielsen, T.; Zeng, X.-X.I.; Martinez-Fernandez, A.; Walls, S.M.; Kervadec, A.; Kezos, J.N.; et al. Patient-Specific Genomics and Cross-Species Functional Analysis Implicate LRP2 in Hypoplastic Left Heart Syndrome. *eLife* **2020**, *9*, e59554, doi:10.7554/eLife.59554.
20. Su, M.-T.; Venkatesh, T.V.; Wu, X.; Golden, K.; Bodmer, R. The Pioneer Gene, *Apontic*, Is Required for Morphogenesis and Function of the *Drosophila* Heart. *Mechanisms of Development* **1999**, *80*, 125–132, doi:10.1016/S0925-4773(98)00197-X.

21. Liu, Q.-X.; Wang, X.-F.; Ikeo, K.; Hirose, S.; Gehring, W.J.; Gojobori, T. Evolutionarily Conserved Transcription Factor Apontic Controls the G1/S Progression by Inducing Cyclin E during Eye Development. *Proceedings of the National Academy of Sciences* **2014**, *111*, 9497–9502, doi:10.1073/pnas.1407145111.
22. Vann, K.R.; Sharma, R.; Hsu, C.-C.; Devoucoux, M.; Tencer, A.H.; Zeng, L.; Lin, K.; Zhu, L.; Li, Q.; Lachance, C.; et al. Structure-Function Relationship of ASH1L and Histone H3K36 and H3K4 Methylation. *Nat Commun* **2025**, *16*, 2235, doi:10.1038/s41467-025-57556-5.
23. Gregory, G.D.; Vakoc, C.R.; Rozovskaia, T.; Zheng, X.; Patel, S.; Nakamura, T.; Canaani, E.; Blobel, G.A. Mammalian ASH1L Is a Histone Methyltransferase That Occupies the Transcribed Region of Active Genes. *Mol Cell Biol* **2007**, *27*, 8466–8479, doi:10.1128/MCB.00993-07.
24. Zhu, J.; Liu, C.; Huang, X.; van de Leemput, J.; Lee, H.; Han, Z. H3K36 Di-Methylation Marks, Mediated by Ash1 in Complex with Caf1-55 and MRG15, Are Required during Drosophila Heart Development. *Journal of Cardiovascular Development and Disease* **2023**, *10*, 307, doi:10.3390/jcdd10070307.
25. Stoller, J.Z.; Huang, L.; Tan, C.C.; Huang, F.; Zhou, D.D.; Yang, J.; Gelb, B.D.; Epstein, J.A. Ash2l Interacts with Tbx1 and Is Required during Early Embryogenesis. *Exp Biol Med (Maywood)* **2010**, *235*, 569–576, doi:10.1258/ebm.2010.009318.
26. Zhu, J.-Y.; van de Leemput, J.; Han, Z. Distinct Roles of COMPASS Subunits to Drosophila Heart Development. *Biol Open* **2024**, *13*, bio061736, doi:10.1242/bio.061736.
27. Zhou, L.; Canagarajah, B.; Zhao, Y.; Baibakov, B.; Tokuhiro, K.; Maric, D.; Dean, J. BTBD18 Regulates a Subset of piRNA-Generating Loci through Transcription Elongation in Mice. *Developmental Cell* **2017**, *40*, 453-466.e5, doi:10.1016/j.devcel.2017.02.007.
28. Junion, G.; Bataillé, L.; Jagla, T.; Ponte, J.P.D.; Tapin, R.; Jagla, K. Genome-Wide View of Cell Fate Specification: Ladybird Acts at Multiple Levels during Diversification of Muscle and Heart Precursors. *Genes Dev.* **2007**, *21*, 3163–3180, doi:10.1101/gad.437307.
29. Couderc, J.-L.; Godt, D.; Zollman, S.; Chen, J.; Li, M.; Tiong, S.; Cramton, S.E.; Sahut-Barnola, I.; Laski, F.A. The Bric à Brac Locus Consists of Two Paralogous Genes Encoding BTB/POZ Domain Proteins and Acts as a Homeotic and Morphogenetic Regulator of Imaginal Development in Drosophila. *Development* **2002**, *129*, 2419–2433, doi:10.1242/dev.129.10.2419.
30. Singh, R.; Hoogaars, W.M.; Barnett, P.; Grieskamp, T.; Rana, M.S.; Buermans, H.; Farin, H.F.; Petry, M.; Heallen, T.; Martin, J.F.; et al. Tbx2 and Tbx3 Induce Atrioventricular Myocardial Development and Endocardial Cushion Formation. *Cell. Mol. Life Sci.* **2012**, *69*, 1377–1389, doi:10.1007/s00018-011-0884-2.
31. Li, Y.; Du, J.; Deng, S.; Liu, B.; Jing, X.; Yan, Y.; Liu, Y.; Wang, J.; Zhou, X.; She, Q. The Molecular Mechanisms of Cardiac Development and Related Diseases. *Sig Transduct Target Ther* **2024**, *9*, 1–60, doi:10.1038/s41392-024-02069-8.
32. Liu, N.; Schoch, K.; Luo, X.; Pena, L.D.M.; Bhavana, V.H.; Kukolich, M.K.; Stringer, S.; Powis, Z.; Radtke, K.; Mroske, C.; et al. Functional Variants in TBX2 Are Associated with a Syndromic Cardiovascular and Skeletal Developmental Disorder. *Human Molecular Genetics* **2018**, *27*, 2454–2465, doi:10.1093/hmg/ddy146.
33. Barish, S.; Berg, K.; Drozd, J.; Berglund-Brown, I.; Khizir, L.; Wasson, L.K.; Seidman, C.E.; Seidman, J.G.; Chen, S.; Brueckner, M. The H2Bub1-Deposition Complex Is Required for Human and Mouse Cardiogenesis. *Development* **2023**, *150*, dev201899, doi:10.1242/dev.201899.
34. Zhu, J.; Fu, Y.; Nettleton, M.; Richman, A.; Han, Z. High Throughput in Vivo Functional Validation of Candidate Congenital Heart Disease Genes in Drosophila. *eLife* **2017**, *6*, e22617, doi:10.7554/eLife.22617.
35. Li, J.; Liu, Y.; Jin, Y.; Wang, R.; Wang, J.; Lu, S.; VanBuren, V.; Dostal, D.E.; Zhang, S.L.; Peng, X. Essential Role of Cdc42 in Cardiomyocyte Proliferation and Cell-Cell Adhesion during Heart Development. *Developmental Biology* **2017**, *421*, 271–283, doi:10.1016/j.ydbio.2016.12.012.
36. Liu, Y.; Jin, Y.; Li, J.; Seto, E.; Kuo, E.; Yu, W.; Schwartz, R.J.; Blazo, M.; Zhang, S.L.; Peng, X. Inactivation of Cdc42 in Neural Crest Cells Causes Craniofacial and Cardiovascular Morphogenesis Defects. *Dev Biol* **2013**, *383*, 239–252, doi:10.1016/j.ydbio.2013.09.013.
37. Fritz, K.R.; Zhang, Y.; Ruest, L.B. Cdc42 Activation by Endothelin Regulates Neural Crest Cell Migration in the Cardiac Outflow Tract. *Dev Dyn* **2019**, *248*, 795–812, doi:10.1002/dvdy.75.
38. Qian, L.; Wythe, J.D.; Liu, J.; Cartry, J.; Vogler, G.; Mohapatra, B.; Otway, R.T.; Huang, Y.; King, I.N.; Maillet, M.; et al. Tinman/Nkx2-5 Acts via miR-1 and Upstream of Cdc42 to Regulate Heart Function across Species. *J Cell Biol* **2011**, *193*, 1181–1196, doi:10.1083/jcb.201006114.
39. Vogler, G.; Liu, J.; Iafe, T.W.; Migh, E.; Mihály, J.; Bodmer, R. Cdc42 and Formin Activity Control Non-Muscle Myosin Dynamics during Drosophila Heart Morphogenesis. *Journal of Cell Biology* **2014**, *206*, 909–922, doi:10.1083/jcb.201405075.

40. Schroeder, A.M.; Allahyari, M.; Vogler, G.; Missinato, M.A.; Nielsen, T.; Yu, M.S.; Theis, J.L.; Larsen, L.A.; Goyal, P.; Rosenfeld, J.A.; et al. Model System Identification of Novel Congenital Heart Disease Gene Candidates: Focus on RPL13. *Human Molecular Genetics* **2019**, *28*, 3954–3969, doi:10.1093/hmg/ddz213.
41. Murphy, N.P.; Lubbers, E.R.; Mohler, P.J. Advancing Our Understanding of AnkRD1 in Cardiac Development and Disease. *Cardiovasc Res* **2020**, *116*, 1402–1404, doi:10.1093/cvr/cvaa063.
42. Bakovic, P.; Mirosevic, V.; Svagusa, T.; Sepac, A.; Kulic, A.; Milicic, D.; Gasparovic, H.; Rudez, I.; Urlic, M.; Sikiric, S.; et al. Reduced Expression of UPRmt Proteins HSP10, HSP60, HTRA2, OMA1, SPG7, and YME1L Is Associated with Accelerated Heart Failure in Humans. *Biomedicines* **2025**, *13*, 1142, doi:10.3390/biomedicines13051142.
43. Clapham, K.R.; Singh, I.; Capuano, I.S.; Rajagopal, S.; Chun, H.J. MEF2 and the Right Ventricle: From Development to Disease. *Front. Cardiovasc. Med.* **2019**, *6*, doi:10.3389/fcvm.2019.00029.
44. Lilly, B.; Zhao, B.; Ranganayakulu, G.; Paterson, B.M.; Schulz, R.A.; Olson, E.N. Requirement of MADS Domain Transcription Factor D-MEF2 for Muscle Formation in Drosophila. *Science* **1995**, *267*, 688–693, doi:10.1126/science.7839146.
45. Hu, Y.; Flockhart, I.; Vinayagam, A.; Bergwitz, C.; Berger, B.; Perrimon, N.; Mohr, S.E. An Integrative Approach to Ortholog Prediction for Disease-Focused and Other Functional Studies. *BMC Bioinformatics* **2011**, *12*, 357, doi:10.1186/1471-2105-12-357.
46. Birker, K.; Ge, S.; Kirkland, N.J.; Theis, J.L.; Marchant, J.; Fogarty, Z.C.; Missinato, M.A.; Kalvakuri, S.; Grossfeld, P.; Engler, A.J.; et al. Mitochondrial MICOS Complex Genes, Implicated in Hypoplastic Left Heart Syndrome, Maintain Cardiac Contractility and Actomyosin Integrity. *eLife* **2023**, *12*, e83385, doi:10.7554/eLife.83385.
47. Jahncke, J.N.; Wright, K.M. The Many Roles of Dystroglycan in Nervous System Development and Function: Dystroglycan and Neural Circuit Development: Dystroglycan and Neural Circuit Development. *Dev Dyn* **2023**, *252*, 61–80, doi:10.1002/dvdy.516.
48. Medioni, C.; Astier, M.; Zmojdzian, M.; Jagla, K.; Sémériva, M. Genetic Control of Cell Morphogenesis during Drosophila Melanogaster Cardiac Tube Formation. *J Cell Biol* **2008**, *182*, 249–261, doi:10.1083/jcb.200801100.
49. Auxerre-Plantié, E.; Nielsen, T.; Grunert, M.; Olejniczak, O.; Perrot, A.; Özcelik, C.; Harries, D.; Matinmehr, F.; Dos Remedios, C.; Mühlfeld, C.; et al. Identification of MYOM2 as a Candidate Gene in Hypertrophic Cardiomyopathy and Tetralogy of Fallot, and Its Functional Evaluation in the Drosophila Heart. *Dis Model Mech* **2020**, *13*, dmm045377, doi:10.1242/dmm.045377.
50. Sadahiro, T.; Isomi, M.; Muraoka, N.; Kojima, H.; Haginiwa, S.; Kurotsu, S.; Tamura, F.; Tani, H.; Tohyama, S.; Fujita, J.; et al. Tbx6 Induces Nascent Mesoderm from Pluripotent Stem Cells and Temporally Controls Cardiac versus Somite Lineage Diversification. *Cell Stem Cell* **2018**, *23*, 382-395.e5, doi:10.1016/j.stem.2018.07.001.
51. Hadjantonakis, A.-K.; Pisano, E.; Papaioannou, V.E. Tbx6 Regulates Left/Right Patterning in Mouse Embryos through Effects on Nodal Cilia and Perinodal Signaling. *PLoS One* **2008**, *3*, e2511, doi:10.1371/journal.pone.0002511.
52. Han, Z.; Olson, E.N. Hand Is a Direct Target of Tinman and GATA Factors during Drosophila Cardiogenesis and Hematopoiesis. *Development* **2005**, *132*, 3525–3536, doi:10.1242/dev.01899.
53. Harrelson, Z.; Kelly, R.G.; Goldin, S.N.; Gibson-Brown, J.J.; Bollag, R.J.; Silver, L.M.; Papaioannou, V.E. Tbx2 Is Essential for Patterning the Atrioventricular Canal and for Morphogenesis of the Outflow Tract during Heart Development. *Development* **2004**, *131*, 5041–5052, doi:10.1242/dev.01378.
54. Oh, Y.; Abid, R.; Dababneh, S.; Bakr, M.; Aslani, T.; Cook, D.P.; Vanderhyden, B.C.; Park, J.G.; Munshi, N.V.; Hui, C.-C.; et al. Transcriptional Regulation of the Postnatal Cardiac Conduction System Heterogeneity. *Nat Commun* **2024**, *15*, 6550, doi:10.1038/s41467-024-50849-1.
55. Lockwood, W.K.; Bodmer, R. The Patterns of *Wingless*, *Decapentaplegic*, and *Tinman* Position the *Drosophila* Heart. *Mechanisms of Development* **2002**, *114*, 13–26, doi:10.1016/S0925-4773(02)00044-8.
56. Johnson, A.N.; Burnett, L.A.; Sellin, J.; Paululat, A.; Newfeld, S.J. Defective Decapentaplegic Signaling Results in Heart Overgrowth and Reduced Cardiac Output in Drosophila. *Genetics* **2007**, *176*, 1609–1624, doi:10.1534/genetics.107.073569.
57. Ma, L.; Lu, M.-F.; Schwartz, R.J.; Martin, J.F. Bmp2 Is Essential for Cardiac Cushion Epithelial-Mesenchymal Transition and Myocardial Patterning. *Development* **2005**, *132*, 5601–5611, doi:10.1242/dev.02156.
58. Bertero, A.; Madrigal, P.; Galli, A.; Hubner, N.C.; Moreno, I.; Burks, D.; Brown, S.; Pedersen, R.A.; Gaffney, D.; Mendjan, S.; et al. Activin/Nodal Signaling and NANOG Orchestrate Human Embryonic Stem Cell Fate Decisions by Controlling the H3K4me3 Chromatin Mark. *Genes Dev.* **2015**, *29*, 702–717, doi:10.1101/gad.255984.114.
59. Yang, Z.; Shah, K.; Khodadadi-Jamayran, A.; Jiang, H. Dpy30 Is Critical for Maintaining the Identity and Function of Adult Hematopoietic Stem Cells. *J Exp Med* **2016**, *213*, 2349–2364, doi:10.1084/jem.20160185.

60. Mollo, N.; Scognamiglio, R.; Conti, A.; Paladino, S.; Nitsch, L.; Izzo, A. Genetics and Molecular Basis of Congenital Heart Defects in Down Syndrome: Role of Extracellular Matrix Regulation. *International Journal of Molecular Sciences* **2023**, *24*, 2918, doi:10.3390/ijms24032918.
61. Dunlevy, L.; Bennett, M.; Slender, A.; Lana-Elola, E.; Tybulewicz, V.L.; Fisher, E.M.C.; Mohun, T. Down's Syndrome-like Cardiac Developmental Defects in Embryos of the Transchromosomic Tc1 Mouse. *Cardiovasc Res* **2010**, *88*, 287–295, doi:10.1093/cvr/cvq193.
62. Grossman, T.R.; Gamliel, A.; Wessells, R.J.; Taghli-Lamallem, O.; Jepsen, K.; Ocorr, K.; Korenberg, J.R.; Peterson, K.L.; Rosenfeld, M.G.; Bodmer, R.; et al. Over-Expression of DSCAM and COL6A2 Cooperatively Generates Congenital Heart Defects. *PLOS Genetics* **2011**, *7*, e1002344, doi:10.1371/journal.pgen.1002344.
63. Raza, Q.; Jacobs, J.R. Guidance Signalling Regulates Leading Edge Behaviour during Collective Cell Migration of Cardiac Cells in *Drosophila*. *Developmental Biology* **2016**, *419*, 285–297, doi:10.1016/j.ydbio.2016.09.005.
64. Sadeghi, M.B.; Nakhaee, A.; Saravani, R.; Sargazi, S. Significant Association of LXRβ (NR1H2) Polymorphisms (Rs28514894, Rs2303044) with Type 2 Diabetes Mellitus and Laboratory Characteristics. *J Diabetes Metab Disord* **2021**, *20*, 261–270, doi:10.1007/s40200-021-00740-3.
65. Zheng, Z.-G.; Zhu, S.-T.; Cheng, H.-M.; Zhang, X.; Cheng, G.; Thu, P.M.; Wang, S.P.; Li, H.-J.; Ding, M.; Qiang, L.; et al. Discovery of a Potent SCAP Degrader That Ameliorates HFD-Induced Obesity, Hyperlipidemia and Insulin Resistance via an Autophagy-Independent Lysosomal Pathway. *Autophagy* **2021**, *17*, 1592–1613, doi:10.1080/15548627.2020.1757955.
66. Guo, X.; Zhong, J.; Zhao, Y.; Fu, Y.; Sun, L.-Y.; Yuan, A.; Liu, J.; Chen, A.F.; Pu, J. LXRα Promotes Abdominal Aortic Aneurysm Formation Through UHRF1 Epigenetic Modification of miR-26b-3p. *Circulation* **2024**, *150*, 30–46, doi:10.1161/CIRCULATIONAHA.123.065202.
67. Lammers, S.; Barrera, V.; Brennecke, P.; Miller, C.; Yoon, J.; Balolong, J.; Anderson, M.S.; Ho Sui, S.; Steinmetz, L.M.; von Andrian, U.H.; et al. Ehf and Fezf2 Regulate Late Medullary Thymic Epithelial Cell and Thymic Tuft Cell Development. *Front Immunol* **2023**, *14*, 1277365, doi:10.3389/fimmu.2023.1277365.
68. Zhou, J.; Chehab, R.; Tkalcevic, J.; Naylor, M.J.; Harris, J.; Wilson, T.J.; Tsao, S.; Tellis, I.; Zavarsek, S.; Xu, D.; et al. Elf5 Is Essential for Early Embryogenesis and Mammary Gland Development during Pregnancy and Lactation. *EMBO J* **2005**, *24*, 635–644, doi:10.1038/sj.emboj.7600538.
69. Alotaibi, H. The Transcription Factor ELF5 Is Essential for Early Preimplantation Development. *Mol Biol Rep* **2023**, *50*, 2119–2125, doi:10.1007/s11033-022-08217-z.
70. Schwarz, B.; Hollfelder, D.; Scharf, K.; Hartmann, L.; Reim, I. Diversification of Heart Progenitor Cells by EGF Signaling and Differential Modulation of ETS Protein Activity. *eLife* **2018**, *7*, e32847, doi:10.7554/eLife.32847.
71. Brown, G.S.; Jang, J.; Li, D. Growth Factors and Their Roles in Cardiac Development and Regeneration: A Narrative Review. *Pediatric Medicine* **2023**, *6*, doi:10.21037/pm-22-17.
72. Iwamoto, R.; Mine, N.; Mizushima, H.; Mekada, E. ErbB1 and ErbB4 Generate Opposing Signals Regulating Mesenchymal Cell Proliferation during Valvulogenesis. *J Cell Sci* **2017**, *130*, 1321–1332, doi:10.1242/jcs.196618.
73. Faiella, A.; D'Esposito, M.; Rambaldi, M.; Acampora, D.; Balsfiore, S.; Stornaiuolo, A.; Mallamaci, A.; Migliaccio, E.; Gulisano, M.; Simeone, A.; et al. Isolation and Mapping of EVx1, a Human Homeobox Gene Homologus to Even-Skipped , Localized at the 5′ End of Hox1 Locus on Chromosome 7. *Nucleic Acids Res* **1991**, *19*, 6541–6545, doi:10.1093/nar/19.23.6541.
74. Goodman, F.R.; Majewski, F.; Collins, A.L.; Scambler, P.J. A 117-Kb Microdeletion Removing HOXD9–HOXD13 and EVX2 Causes Synpolydactyly. *Am J Hum Genet* **2002**, *70*, 547–555, doi:10.1086/338921.
75. Fujioka, M.; Wessells, R.J.; Han, Z.; Liu, J.; Fitzgerald, K.; Yusibova, G.L.; Zamora, M.; Ruiz-Lozano, P.; Bodmer, R.; Jaynes, J.B. Embryonic Even Skipped–Dependent Muscle and Heart Cell Fates Are Required for Normal Adult Activity, Heart Function, and Lifespan. *Circulation Research* **2005**, *97*, 1108–1114, doi:10.1161/01.RES.0000191546.08532.B2.
76. Bhanot, P.; Fish, M.; Jemison, J.A.; Nusse, R.; Nathans, J.; Cadigan, K.M. Frizzled and DFrizzled-2 Function as Redundant Receptors for Wingless during Drosophila Embryonic Development. *Development* **1999**, *126*, 4175–4186, doi:10.1242/dev.126.18.4175.
77. Chen, C.; Struhl, G. Wingless Transduction by the Frizzled and Frizzled2 Proteins of Drosophila. *Development* **1999**, *126*, 5441–5452, doi:10.1242/dev.126.23.5441.
78. Szabo, L.; Morey, R.; Palpant, N.J.; Wang, P.L.; Afari, N.; Jiang, C.; Parast, M.M.; Murry, C.E.; Laurent, L.C.; Salzman, J. Statistically Based Splicing Detection Reveals Neural Enrichment and Tissue-Specific Induction of Circular RNA during Human Fetal Development. *Genome Biology* **2015**, *16*, 126, doi:10.1186/s13059-015-0690-5.
79. Eisa-Beygi, S.; Hatch, G.; Noble, S.; Ekker, M.; Moon, T.W. The 3-Hydroxy-3-Methylglutaryl-CoA Reductase (HMGCR) Pathway Regulates Developmental Cerebral-Vascular Stability via Prenylation-Dependent Signalling Pathway. *Developmental Biology* **2013**, *373*, 258–266, doi:10.1016/j.ydbio.2012.11.024.

80. Yi, P.; Han, Z.; Li, X.; Olson, E.N. The Mevalonate Pathway Controls Heart Formation in Drosophila by Isoprenylation of Gγ1. *Science* **2006**, *313*, 1301–1303, doi:10.1126/science.1127704.
81. Torregrosa-Carrión, R.; Piñeiro-Sabarís, R.; Siguero-Álvarez, M.; Grego-Bessa, J.; Luna-Zurita, L.; Fernandes, V.S.; MacGrogan, D.; Stainier, D.Y.R.; de la Pompa, J.L. Adhesion G Protein-Coupled Receptor Gpr126/Adgrg6 Is Essential for Placental Development. *Sci Adv* **2021**, *7*, eabj5445, doi:10.1126/sciadv.abj5445.
82. Patel, M.V.; Zhu, J.; Jiang, Z.; Richman, A.; VanBerkum, M.F.A.; Han, Z. Gia/Mthl5 Is an Aorta Specific GPCR Required for *Drosophila* Heart Tube Morphology and Normal Pericardial Cell Positioning. *Developmental Biology* **2016**, *414*, 100–107, doi:10.1016/j.ydbio.2016.03.009.
83. Lu, S.; Liu, S.; Wietelmann, A.; Kojonazarov, B.; Atzberger, A.; Tang, C.; Schermuly, R.T.; Gröne, H.-J.; Offermanns, S. Developmental Vascular Remodeling Defects and Postnatal Kidney Failure in Mice Lacking Gpr116 (Adgrf5) and Eltd1 (Adgrl4). *PLoS One* **2017**, *12*, e0183166, doi:10.1371/journal.pone.0183166.
84. Tanaka, K.; Chen, M.; Prendergast, A.; Zhuang, Z.; Nasiri, A.; Joshi, D.; Hintzen, J.; Chung, M.; Kumar, A.; Mani, A.; et al. Latrophilin-2 Mediates Fluid Shear Stress Mechanotransduction at Endothelial Junctions. *The EMBO Journal* **2024**, *43*, 3175–3191, doi:10.1038/s44318-024-00142-0.
85. Chiba, Y.; Yoshizaki, K.; Saito, K.; Ikeuchi, T.; Iwamoto, T.; Rhodes, C.; Nakamura, T.; de Vega, S.; Morell, R.J.; Boger, E.T.; et al. G Protein-Coupled Receptor Gpr115 (Adgrf4) Is Required for Enamel Mineralization Mediated by Ameloblasts. *J Biol Chem* **2020**, *295*, 15328–15341, doi:10.1074/jbc.RA120.014281.
86. Vitobello, A.; Mazel, B.; Lelianova, V.G.; Zangrandi, A.; Petitto, E.; Suckling, J.; Salpietro, V.; Meyer, R.; Elbracht, M.; Kurth, I.; et al. ADGRL1 Haploinsufficiency Causes a Variable Spectrum of Neurodevelopmental Disorders in Humans and Alters Synaptic Activity and Behavior in a Mouse Model. *Am J Hum Genet* **2022**, *109*, 1436–1457, doi:10.1016/j.ajhg.2022.06.011.
87. Oliveira, F.G.; Rosa-E-Silva, J.C.; Gomes, A.G.; Grzesiuk, J.D.; Vidotto, T.; Squire, J.A.; Panepucci, R.A.; Meola, J.; Martelli, L. Identification of a Rare Copy Number Polymorphic Gain at 3q12.2 with Candidate Genes for Familial Endometriosis. *Rev Bras Ginecol Obstet* **2024**, *46*, e-rbgo12, doi:10.61622/rbgo/2024CR12.
88. Tan, M.-Q.; Tang, Y. [Gene mutations in congenital bilateral absence of the vas deferens: An update]. *Zhonghua Nan Ke Xue* **2021**, *27*, 450–455.
89. Vidal, O.M.; Vélez, J.I.; Arcos-Burgos, M. ADGRL3 Genomic Variation Implicated in Neurogenesis and ADHD Links Functional Effects to the Incretin Polypeptide GIP. *Sci Rep* **2022**, *12*, 15922, doi:10.1038/s41598-022-20343-z.
90. Gao, X.; Yan, B. The Mechanism and Diagnostic Value of Tbx20 in Cardiovascular Diseases. *Gene Reports* **2023**, *30*, 101723, doi:10.1016/j.genrep.2022.101723.
91. Reim, I.; Mohler, J.P.; Frasch, M. *Tbx20*-Related Genes, *Mid* and *H15*, Are Required for *Tinman* Expression, Proper Patterning, and Normal Differentiation of Cardioblasts in *Drosophila*. *Mechanisms of Development* **2005**, *122*, 1056–1069, doi:10.1016/j.mod.2005.04.006.
92. George, R.M.; Firulli, B.A.; Podicheti, R.; Rusch, D.B.; Mannion, B.J.; Pennacchio, L.A.; Osterwalder, M.; Firulli, A.B. Single Cell Evaluation of Endocardial Hand2 Gene Regulatory Networks Reveals HAND2-Dependent Pathways That Impact Cardiac Morphogenesis. *Development* **2023**, *150*, dev201341, doi:10.1242/dev.201341.
93. Han, Z.; Yi, P.; Li, X.; Olson, E.N. Hand, an Evolutionarily Conserved bHLH Transcription Factor Required for Drosophila Cardiogenesis and Hematopoiesis. *Development* **2006**, *133*, 1175–1182, doi:10.1242/dev.02285.
94. Lo, P.C.H.; Zaffran, S.; Sénatore, S.; Frasch, M. The Drosophila Hand Gene Is Required for Remodeling of the Developing Adult Heart and Midgut during Metamorphosis. *Dev Biol* **2007**, *311*, 287–296, doi:10.1016/j.ydbio.2007.08.024.
95. Quintana, A.M.; Geiger, E.A.; Achilly, N.; Rosenblatt, D.S.; Maclean, K.N.; Stabler, S.P.; Artinger, K.B.; Appel, B.; Shaikh, T.H. *Hcfc1b*, a Zebrafish Ortholog of *HCFC1*, Regulates Craniofacial Development by Modulating *Mmachc* Expression. *Developmental Biology* **2014**, *396*, 94–106, doi:10.1016/j.ydbio.2014.09.026.
96. Huang, W.; Zhu, J.; Fu, Y.; van de Leemput, J.; Han, Z. *Lpt*, *Trr*, and *Hcf* Regulate Histone Mono- and Dimethylation That Are Essential for *Drosophila* Heart Development. *Developmental Biology* **2022**, *490*, 53–65, doi:10.1016/j.ydbio.2022.07.003.
97. Reynolds, J.J.; Bicknell, L.S.; Carroll, P.; Higgs, M.R.; Shaheen, R.; Murray, J.E.; Papadopoulos, D.K.; Leitch, A.; Murina, O.; Tarnauskaitė, Ž.; et al. Mutations in DONSON Disrupt Replication Fork Stability and Cause Microcephalic Dwarfism. *Nat Genet* **2017**, *49*, 537–549, doi:10.1038/ng.3790.
98. Inoue, S.; Nosetani, M.; Nakajima, Y.; Sakaki, S.; Kato, H.; Saba, R.; Takeshita, N.; Nishikawa, K.; Ueyama, A.; Matsuo, K.; et al. Sonic Hedgehog Signaling Regulates the Optimal Differentiation Pace from Early-Stage Mesoderm to Cardiogenic Mesoderm in Mice. *Dev Growth Differ* **2025**, *67*, 75–84, doi:10.1111/dgd.12955.
99. Park, M.; Wu, X.; Golden, K.; Axelrod, J.D.; Bodmer, R. The Wingless Signaling Pathway Is Directly Involved in*Drosophila*Heart Development. *Developmental Biology* **1996**, *177*, 104–116, doi:10.1006/dbio.1996.0149.

100. Liu, J.; Qian, L.; Wessells, R.J.; Bidet, Y.; Jagla, K.; Bodmer, R. Hedgehog and RAS Pathways Cooperate in the Anterior–Posterior Specification and Positioning of Cardiac Progenitor Cells. *Developmental Biology* **2006**, *290*, 373–385, doi:10.1016/j.ydbio.2005.11.033.
101. Dell'Era, P.; Ronca, R.; Coco, L.; Nicoli, S.; Metra, M.; Presta, M. Fibroblast Growth Factor Receptor-1 Is Essential for In Vitro Cardiomyocyte Development. *Circulation Research* **2003**, *93*, 414–420, doi:10.1161/01.RES.0000089460.12061.E1.
102. Kadam, S.; McMahon, A.; Tzou, P.; Stathopoulos, A. FGF Ligands in Drosophila Have Distinct Activities Required to Support Cell Migration and Differentiation. *Development* **2009**, *136*, 739–747, doi:10.1242/dev.027904.
103. Dorey, K.; Amaya, E. FGF Signalling: Diverse Roles during Early Vertebrate Embryogenesis. *Development* **2010**, *137*, 3731–3742, doi:10.1242/dev.037689.
104. Sato, A.; Scholl, A.M.; Kuhn, E.B.; Stadt, H.A.; Decker, J.R.; Pegram, K.; Hutson, M.R.; Kirby, M.L. FGF8 Signaling Is Chemotactic for Cardiac Neural Crest Cells. *Developmental Biology* **2011**, *354*, 18–30, doi:10.1016/j.ydbio.2011.03.010.
105. Mysliwiec, M.R.; Bresnick, E.; Lee, Y. Abstract 21584: Jarid2/Jumonji Dependent Epigenetic Control of Notch1 Expression Is Required for Normal Cardiac Development. *Circulation* **2010**, *122*, A21584–A21584, doi:10.1161/circ.122.suppl_21.A21584.
106. Barth, J.L.; Clark, C.D.; Fresco, V.M.; Knoll, E.P.; Lee, B.; Argraves, W.S.; Lee, K.-H. Jarid2 Is among a Set of Genes Differentially Regulated by Nkx2.5 during Outflow Tract Morphogenesis. *Developmental Dynamics* **2010**, *239*, 2024–2033, doi:10.1002/dvdy.22341.
107. Basu, M.; Zhu, J.-Y.; LaHaye, S.; Majumdar, U.; Jiao, K.; Han, Z.; Garg, V. Epigenetic Mechanisms Underlying Maternal Diabetes-Associated Risk of Congenital Heart Disease. *JCI Insight* **2017**, *2*, e95085, doi:10.1172/jci.insight.95085.
108. Akasaka, T.; Ocorr, K.; Lin, L.; Vogler, G.; Bodmer, R.; Grossfeld, P. Overexpression of Kif1A in the Developing Drosophila Heart Causes Valvar and Contractility Defects: Implications for Human Congenital Heart Disease. *Journal of Cardiovascular Development and Disease* **2020**, *7*, 22, doi:10.3390/jcdd7020022.
109. Han, P.; Hang, C.T.; Yang, J.; Chang, C.-P. Chromatin Remodeling in Cardiovascular Development and Physiology. *Circulation Research* **2011**, *108*, 378–396, doi:10.1161/CIRCRESAHA.110.224287.
110. Zhang, X.; Cai, S.; Chen, L.; Yuan, R.; Nie, Y.; Ding, S.; Fang, Y.; Zhu, Q.; Chen, K.; Wei, H.; et al. Integrated miRNA-mRNA Transcriptomic Analysis Reveals Epigenetic-Mediated Embryonic Muscle Growth Differences between Wuzhishan and Landrace Pigs1. *J Anim Sci* **2019**, *97*, 1967–1978, doi:10.1093/jas/skz091.
111. Zhang, C.; Tian, L.; Chi, C.; Wu, X.; Yang, X.; Han, M.; Xu, T.; Zhuang, Y.; Deng, K. Adam10 Is Essential for Early Embryonic Cardiovascular Development. *Developmental Dynamics* **2010**, *239*, 2594–2602, doi:10.1002/dvdy.22391.
112. Farber, G.; Parks, M.M.; Lustgarten Guahmich, N.; Zhang, Y.; Monette, S.; Blanchard, S.C.; Di Lorenzo, A.; Blobel, C.P. ADAM10 Controls the Differentiation of the Coronary Arterial Endothelium. *Angiogenesis* **2019**, *22*, 237–250, doi:10.1007/s10456-018-9653-2.
113. Albrecht, S.; Wang, S.; Holz, A.; Bergter, A.; Paululat, A. The ADAM Metalloprotease Kuzbanian Is Crucial for Proper Heart Formation in *Drosophila Melanogaster*. *Mechanisms of Development* **2006**, *123*, 372–387, doi:10.1016/j.mod.2006.03.005.
114. Voges, H.K.; Foster, S.R.; Reynolds, L.; Parker, B.L.; Devilée, L.; Quaife-Ryan, G.A.; Fortuna, P.R.J.; Mathieson, E.; Fitzsimmons, R.; Lor, M.; et al. Vascular Cells Improve Functionality of Human Cardiac Organoids. *Cell Reports* **2023**, *42*, doi:10.1016/j.celrep.2023.112322.
115. Stark, K.A.; Yee, G.H.; Roote, C.E.; Williams, E.L.; Zusman, S.; Hynes, R.O. A Novel α Integrin Subunit Associates with β PS and Functions in Tissue Morphogenesis and Movement during Drosophila Development. *Development* **1997**, *124*, 4583–4594, doi:10.1242/dev.124.22.4583.
116. Nishiyama, M.; Takase, M.; Tanaka, Y.; Gamo, S. Ether-Resistant Mutant of Laminin Alpha Subunit (*LanA*) in *Drosophila Melanogaster*. *International Congress Series* **2005**, *1283*, 260–262, doi:10.1016/j.ics.2005.07.082.
117. Deogharia, M.; Venegas-Zamora, L.; Agrawal, A.; Shi, M.; Jain, A.K.; McHugh, K.J.; Altamirano, F.; Marian, A.J.; Gurha, P. Histone Demethylase KDM5 Regulates Cardiomyocyte Maturation by Promoting Fatty Acid Oxidation, Oxidative Phosphorylation, and Myofibrillar Organization. *Cardiovasc Res* **2024**, *120*, 630–643, doi:10.1093/cvr/cvae014.
118. Ang, S.-Y.; Uebersohn, A.; Spencer, C.I.; Huang, Y.; Lee, J.-E.; Ge, K.; Bruneau, B.G. KMT2D Regulates Specific Programs in Heart Development via Histone H3 Lysine 4 Di-Methylation. *Development* **2016**, *143*, 810–821, doi:10.1242/dev.132688.
119. Jiang, D.-S.; Yi, X.; Li, R.; Su, Y.-S.; Wang, J.; Chen, M.-L.; Liu, L.-G.; Hu, M.; Cheng, C.; Zheng, P.; et al. The Histone Methyltransferase Mixed Lineage Leukemia (MLL) 3 May Play a Potential Role in Clinical Dilated Cardiomyopathy. *Mol Med* **2017**, *23*, 196–203, doi:10.2119/molmed.2017.00012.

120. Rots, D.; Choufani, S.; Faundes, V.; Dingemans, A.J.M.; Joss, S.; Foulds, N.; Jones, E.A.; Stewart, S.; Vasudevan, P.; Dabir, T.; et al. Pathogenic Variants in KMT2C Result in a Neurodevelopmental Disorder Distinct from Kleefstra and Kabuki Syndromes. *The American Journal of Human Genetics* **2024**, *111*, 1626–1642, doi:10.1016/j.ajhg.2024.06.009.
121. Riedel, F.; Vorkel, D.; Eaton, S. Megalin-Dependent Yellow Endocytosis Restricts Melanization in the Drosophila Cuticle. *Development* **2011**, *138*, 149–158, doi:10.1242/dev.056309.
122. Brauer, P.R.; Cai, D.H. Expression of Tissue Inhibitor of Metalloproteinases (TIMPs) during Early Cardiac Development. *Mechanisms of Development* **2002**, *113*, 175–179, doi:10.1016/S0925-4773(02)00016-3.
123. Raza, Q.S.; Vanderploeg, J.L.; Jacobs, J.R. Matrix Metalloproteinases Are Required for Membrane Motility and Lumenogenesis during Drosophila Heart Development. *PLOS ONE* **2017**, *12*, e0171905, doi:10.1371/journal.pone.0171905.
124. Hughes, C.J.R.; Turner, S.; Andrews, R.M.; Vitkin, A.; Jacobs, J.R. Matrix Metalloproteinases Regulate ECM Accumulation but Not Larval Heart Growth in *Drosophila Melanogaster*. *Journal of Molecular and Cellular Cardiology* **2020**, *140*, 42–55, doi:10.1016/j.yjmcc.2020.02.008.
125. Muñoz-Sáez, E.; Moracho, N.; Learte, A.I.R.; Arroyo, A.G.; Sánchez-Camacho, C. Dynamic Expression of Membrane Type 1-Matrix Metalloproteinase (Mt1-Mmp/Mmp14) in the Mouse Embryo. *Cells* **2021**, *10*, 2448, doi:10.3390/cells10092448.
126. Cantemir, V.; Cai, D. h.; Reedy, M. v.; Brauer, P. r. Tissue Inhibitor of Metalloproteinase-2 (TIMP-2) Expression during Cardiac Neural Crest Cell Migration and Its Role in proMMP-2 Activation. *Developmental Dynamics* **2004**, *231*, 709–719, doi:10.1002/dvdy.20171.
127. Tao, G.; Levay, A.K.; Gridley, T.; Lincoln, J. *Mmp15* Is a Direct Target of Snai1 during Endothelial to Mesenchymal Transformation and Endocardial Cushion Development. *Developmental Biology* **2011**, *359*, 209–221, doi:10.1016/j.ydbio.2011.08.022.
128. Zhang, Y.; Huang, L.; Wang, C.; Gao, D.; Zuo, Z. Phenanthrene Exposure Produces Cardiac Defects during Embryo Development of Zebrafish (Danio Rerio) through Activation of MMP-9. *Chemosphere* **2013**, *93*, 1168–1175, doi:10.1016/j.chemosphere.2013.06.056.
129. Novotny, E.; Compton, S.; Liu, P.P.; Collins, F.S.; Chandrasekharappa, S.C. In Vitro Hematopoietic Differentiation of Mouse Embryonic Stem Cells Requires the Tumor Suppressor Menin and Is Mediated by Hoxa9. *Mech Dev* **2009**, *126*, 517–522, doi:10.1016/j.mod.2009.04.001.
130. Zhang, H.-L.; Luo, T.-H.; Feng, L.; Zhao, Y.; Li, W.-Y.; Xu, J.; Zhang, Q.; Xu, L.-H.; Zheng, S.; Li, G.; et al. Microarray Analysis of Gene Expression in Men1 Knockout Embryoid Body Reveals Genetic Events Involved in Early Mouse Embryonic Development. *Biochem Biophys Res Commun* **2007**, *352*, 456–462, doi:10.1016/j.bbrc.2006.11.031.
131. Chen, Y.-H.; Ishii, M.; Sun, J.; Sucov, H.M.; Maxson, R.E. *Msx1* and *Msx2* Regulate Survival of Secondary Heart Field Precursors and Post-Migratory Proliferation of Cardiac Neural Crest in the Outflow Tract. *Developmental Biology* **2007**, *308*, 421–437, doi:10.1016/j.ydbio.2007.05.037.
132. Lopes, M.; Goupille, O.; Cloment, C.S.; Lallemand, Y.; Cumano, A.; Robert, B. Msx Genes Define a Population of Mural Cell Precursors Required for Head Blood Vessel Maturation. *Development* **2011**, *138*, 3055–3066, doi:10.1242/dev.063214.
133. Bodmer, R.; Jan, L.Y.; Jan, Y.N. A New Homeobox-Containing Gene, Msh-2, Is Transiently Expressed Early during Mesoderm Formation of Drosophila. *Development* **1990**, *110*, 661–669, doi:10.1242/dev.110.3.661.
134. Murayama, E.; Sarris, M.; Redd, M.; Le Guyader, D.; Vivier, C.; Horsley, W.; Trede, N.; Herbomel, P. NACA Deficiency Reveals the Crucial Role of Somite-Derived Stromal Cells in Haematopoietic Niche Formation. *Nat Commun* **2015**, *6*, 8375, doi:10.1038/ncomms9375.
135. Li, H.; Randall, W.R.; Du, S.-J. skNAC (Skeletal Naca), a Muscle-Specific Isoform of Naca (Nascent Polypeptide-Associated Complex Alpha), Is Required for Myofibril Organization. *FASEB J* **2009**, *23*, 1988–2000, doi:10.1096/fj.08-125542.
136. Berger, F.; Berkholz, J.; Breustedt, T.; Ploen, D.; Munz, B. Skeletal Muscle-Specific Variant of Nascent Polypeptide Associated Complex Alpha (skNAC): Implications for a Specific Role in Mammalian Myoblast Differentiation. *Eur J Cell Biol* **2012**, *91*, 150–155, doi:10.1016/j.ejcb.2011.10.004.
137. Matos-Nieves, A.; Greskovich, S.C.; Choudhury, T.Z.; Manivannan, S.; Ueyama, Y.; Rao, A.S.; Cameron, E.M.; Garg, V. Expression of Netrin-1 in the Developing Mouse Heart. *Gene Expression Patterns* **2025**, *56*, 119398, doi:10.1016/j.gep.2025.119398.
138. Azhdari, M.; zur Hausen, A. Wnt/β-Catenin and Notch Signaling Pathways in Cardiovascular Disease: Mechanisms and Therapeutics Approaches. *Pharmacological Research* **2025**, *211*, 107565, doi:10.1016/j.phrs.2024.107565.

139. Paolini, A.; Fontana, F.; Pham, V.-C.; Rödel, C.J.; Abdelilah-Seyfried, S. Mechanosensitive Notch-Dll4 and Klf2-Wnt9 Signaling Pathways Intersect in Guiding Valvulogenesis in Zebrafish. *Cell Rep* **2021**, *37*, 109782, doi:10.1016/j.celrep.2021.109782.
140. Wang, Q.; Zhao, N.; Kennard, S.; Lilly, B. Notch2 and Notch3 Function Together to Regulate Vascular Smooth Muscle Development. *PLoS One* **2012**, *7*, e37365, doi:10.1371/journal.pone.0037365.
141. Niikura, Y.; Tabata, Y.; Tajima, A.; Inoue, I.; Arai, K.; Watanabe, S. Zebrafish Numb Homologue: Phylogenetic Evolution and Involvement in Regulation of Left–Right Asymmetry. *Mechanisms of Development* **2006**, *123*, 407–414, doi:10.1016/j.mod.2006.03.008.
142. Zhao, C.; Guo, H.; Li, J.; Myint, T.; Pittman, W.; Yang, L.; Zhong, W.; Schwartz, R.J.; Schwarz, J.J.; Singer, H.A.; et al. Numb Family Proteins Are Essential for Cardiac Morphogenesis and Progenitor Differentiation. *Development* **2014**, *141*, 281–295, doi:10.1242/dev.093690.
143. Gajewski, K.; Choi, C.Y.; Kim, Y.; Schulz, R.A. Genetically Distinct Cardial Cells within the Drosophila Heart. *Genesis* **2000**, *28*, 36–43, doi:10.1002/1526-968x(200009)28:1%3C36::aid-gene50%3E3.0.co;2-4.
144. Nomaru, H.; Liu, Y.; De Bono, C.; Righelli, D.; Cirino, A.; Wang, W.; Song, H.; Racedo, S.E.; Dantas, A.G.; Zhang, L.; et al. Single Cell Multi-Omic Analysis Identifies a Tbx1-Dependent Multilineage Primed Population in Murine Cardiopharyngeal Mesoderm. *Nat Commun* **2021**, *12*, 6645, doi:10.1038/s41467-021-26966-6.
145. Schaub, C.; Nagaso, H.; Jin, H.; Frasch, M. Org-1, the Drosophila Ortholog of Tbx1, Is a Direct Activator of Known Identity Genes during Muscle Specification. *Development* **2012**, *139*, 1001–1012, doi:10.1242/dev.073890.
146. Boukhatmi, H.; Schaub, C.; Bataillé, L.; Reim, I.; Frendo, J.-L.; Frasch, M.; Vincent, A. An Org-1–Tup Transcriptional Cascade Reveals Different Types of Alary Muscles Connecting Internal Organs in Drosophila. *Development* **2014**, *141*, 3761–3771, doi:10.1242/dev.111005.
147. Yilbas, A.; Hamilton, A.; Wang, Y.; Mach, H.; Lacroix, N.; Davis, D.R.; Chen, J.; Li, Q. Activation of GATA4 Gene Expression at the Early Stage of Cardiac Specification. *Front. Chem.* **2014**, *2*, doi:10.3389/fchem.2014.00012.
148. Afouda, B.A. Towards Understanding the Gene-Specific Roles of GATA Factors in Heart Development: Does GATA4 Lead the Way? *Int J Mol Sci* **2022**, *23*, 5255, doi:10.3390/ijms23095255.
149. Rivera-Feliciano, J.; Lee, K.-H.; Kong, S.W.; Rajagopal, S.; Ma, Q.; Springer, Z.; Izumo, S.; Tabin, C.J.; Pu, W.T. Development of Heart Valves Requires Gata4 Expression in Endothelial-Derived Cells. *Development* **2006**, *133*, 3607–3618, doi:10.1242/dev.02519.
150. Theis, J.L.; Niaz, T.; Sundsbak, R.S.; Fogarty, Z.C.; Bamlet, W.R.; Hagler, D.J.; Olson, T.M. CELSR1 Risk Alleles in Familial Bicuspid Aortic Valve and Hypoplastic Left Heart Syndrome. *Circulation: Genomic and Precision Medicine* **2022**, *15*, e003523, doi:10.1161/CIRCGEN.121.003523.
151. Cantù, C.; Felker, A.; Zimmerli, D.; Prummel, K.D.; Cabello, E.M.; Chiavacci, E.; Méndez-Acevedo, K.M.; Kirchgeorg, L.; Burger, S.; Ripoll, J.; et al. Mutations in Bcl9 and Pygo Genes Cause Congenital Heart Defects by Tissue-Specific Perturbation of Wnt/β-Catenin Signaling. *Genes Dev.* **2018**, *32*, 1443–1458, doi:10.1101/gad.315531.118.
152. Tang, M.; Yuan, W.; Bodmer, R.; Wu, X.; Ocorr, K. The Role of Pygopus in the Differentiation of Intra-Cardiac Valves in Drosophila. *Genesis* **2014**, *52*, 19–28, doi:10.1002/dvg.22724.
153. Itoh, N.; Ohta, H.; Nakayama, Y.; Konishi, M. Roles of FGF Signals in Heart Development, Health, and Disease. *Front Cell Dev Biol* **2016**, *4*, 110, doi:10.3389/fcell.2016.00110.
154. Zhong, H.; Zhang, R.; Li, G.; Huang, P.; Zhang, Y.; Zhu, J.; Kuang, J.; Hutchins, A.P.; Qin, D.; Zhu, P.; et al. C-JUN Is a Barrier in hESC to Cardiomyocyte Transition. *Life Science Alliance* **2023**, *6*, doi:10.26508/lsa.202302121.
155. Jászai, J.; Brand, M. Cloning and Expression of *Ventrhoid*, a Novel Vertebrate Homologue of the *Drosophila* EGF Pathway Gene *Rhomboid*. *Mechanisms of Development* **2002**, *113*, 73–77, doi:10.1016/S0925-4773(01)00655-4.
156. Mommersteeg, M.T.M.; Yeh, M.L.; Parnavelas, J.G.; Andrews, W.D. Disrupted Slit-Robo Signalling Results in Membranous Ventricular Septum Defects and Bicuspid Aortic Valves. *Cardiovasc Res* **2015**, *106*, 55–66, doi:10.1093/cvr/cvv040.
157. Qian, L.; Liu, J.; Bodmer, R. Slit and Robo Control Cardiac Cell Polarity and Morphogenesis. *Current Biology* **2005**, *15*, 2271–2278, doi:10.1016/j.cub.2005.10.037.
158. MacMullin, A.; Jacobs, J.R. Slit Coordinates Cardiac Morphogenesis in *Drosophila*. *Developmental Biology* **2006**, *293*, 154–164, doi:10.1016/j.ydbio.2006.01.027.
159. Santiago-Martínez, E.; Soplop, N.H.; Patel, R.; Kramer, S.G. Repulsion by Slit and Roundabout Prevents Shotgun/E-Cadherin-Mediated Cell Adhesion during Drosophila Heart Tube Lumen Formation. *J Cell Biol* **2008**, *182*, 241–248, doi:10.1083/jcb.200804120.
160. Zmojdzian, M.; Da Ponte, J.P.; Jagla, K. Cellular Components and Signals Required for the Cardiac Outflow Tract Assembly in Drosophila. *Proceedings of the National Academy of Sciences* **2008**, *105*, 2475–2480, doi:10.1073/pnas.0706402105.

161. Zmojdzian, M.; Joussineau, S. de; Ponte, J.P.D.; Jagla, K. Distinct Subsets of Eve-Positive Pericardial Cells Stabilise Cardiac Outflow and Contribute to Hox Gene-Triggered Heart Morphogenesis in Drosophila. *Development (Cambridge, England)* **2018**, *145*, dev158717, doi:10.1242/dev.158717.
162. Mommersteeg, M.T.M.; Andrews, W.D.; Ypsilanti, A.R.; Zelina, P.; Yeh, M.L.; Norden, J.; Kispert, A.; Chédotal, A.; Christoffels, V.M.; Parnavelas, J.G. Slit–Roundabout Signaling Regulates the Development of the Cardiac Systemic Venous Return and Pericardium. *Circulation Research* **2013**, *112*, 465–475, doi:10.1161/CIRCRESAHA.112.277426.
163. Zhao, J.; Mommersteeg, M.T.M. Slit–Robo Signalling in Heart Development. *Cardiovascular Research* **2018**, *114*, 794–804, doi:10.1093/cvr/cvy061.
164. Nim, H.T.; Dang, L.; Thiyagarajah, H.; Bakopoulos, D.; See, M.; Charitakis, N.; Sibbritt, T.; Eichenlaub, M.P.; Archer, S.K.; Fossat, N.; et al. A Cis-Regulatory-Directed Pipeline for the Identification of Genes Involved in Cardiac Development and Disease. *Genome Biol* **2021**, *22*, 335, doi:10.1186/s13059-021-02539-0.
165. Li, D.; Ma, Q. Ubiquitin-Specific Protease: An Emerging Key Player in Cardiomyopathy. *Cell Communication and Signaling* **2025**, *23*, 143, doi:10.1186/s12964-025-02123-0.
166. Fei, X.; Song, C.; Cui, J.; Li, Y.; Lei, X.; Tang, H. The Role of Deubiquitinases in Cardiovascular Diseases: Mechanisms and Therapeutic Implications. *Front. Cardiovasc. Med.* **2025**, *12*, doi:10.3389/fcvm.2025.1582049.
167. Fraile, J.M.; Campos-Iglesias, D.; Rodríguez, F.; Astudillo, A.; Vilarrasa-Blasi, R.; Verdaguer-Dot, N.; Prado, M.A.; Paulo, J.A.; Gygi, S.P.; Martín-Subero, J.I.; et al. Loss of the Deubiquitinase USP36 Destabilizes the RNA Helicase DHX33 and Causes Preimplantation Lethality in Mice. *J Biol Chem* **2018**, *293*, 2183–2194, doi:10.1074/jbc.M117.788430.
168. Zhu, J.; van de Leemput, J.; Han, Z. The Roles of Histone Lysine Methyltransferases in Heart Development and Disease. *J Cardiovasc Dev Dis* **2023**, *10*, 305, doi:10.3390/jcdd10070305.
169. Kranz, A.; Anastassiadis, K. The Role of SETD1A and SETD1B in Development and Disease. *Biochim Biophys Acta Gene Regul Mech* **2020**, *1863*, 194578, doi:10.1016/j.bbagrm.2020.194578.
170. Chen, F.; Chen, J.; Wang, H.; Tang, H.; Huang, L.; Wang, S.; Wang, X.; Fang, X.; Liu, J.; Li, L.; et al. Histone Lysine Methyltransferase SETD2 Regulates Coronary Vascular Development in Embryonic Mouse Hearts. *Front Cell Dev Biol* **2021**, *9*, 651655, doi:10.3389/fcell.2021.651655.
171. Qiao, X.; Liu, Y.; Li, P.; Chen, Z.; Li, H.; Yang, X.; Finnell, R.H.; Yang, Z.; Zhang, T.; Qiao, B.; et al. Genetic Analysis of Rare Coding Mutations of CELSR1–3 in Congenital Heart and Neural Tube Defects in Chinese People. *Clin Sci (Lond)* **2016**, *130*, 2329–2340, doi:10.1042/CS20160686.
172. Wansleeben, C.; Meijlink, F. The Planar Cell Polarity Pathway in Vertebrate Development. *Developmental Dynamics* **2011**, *240*, 616–626, doi:10.1002/dvdy.22564.
173. Humeres, C.; Venugopal, H.; Frangogiannis, N.G. Smad-Dependent Pathways in the Infarcted and Failing Heart. *Curr Opin Pharmacol* **2022**, *64*, 102207, doi:10.1016/j.coph.2022.102207.
174. Wang, W.; Song, B.; Anbarchian, T.; Shirazyan, A.; Sadik, J.E.; Lyons, K.M. Smad2 and Smad3 Regulate Chondrocyte Proliferation and Differentiation in the Growth Plate. *PLoS Genet* **2016**, *12*, e1006352, doi:10.1371/journal.pgen.1006352.
175. Chandran, L.; Backer, W.; Schleutker, R.; Kong, D.; Beati, S.A.H.; Luschnig, S.; Müller, H.-A.J. Src42A Is Required for E-Cadherin Dynamics at Cell Junctions during Drosophila Axis Elongation. *Development* **2023**, *150*, dev201119, doi:10.1242/dev.201119.
176. Vanderploeg, J.; Jacobs, J.R. Mapping Heart Development in Flies: Src42A Acts Non-Autonomously to Promote Heart Tube Formation in Drosophila. *Vet Sci* **2017**, *4*, 23, doi:10.3390/vetsci4020023.
177. Mansoor, W.; Heidari, M.M.; Khatami, M.; Hadadzadeh, M.; Tabrizi, F.; Darvand Araghi, M.H. Rare Pathogenic *NR2F2* (COUP-TFII) Variants as Potential Etiological Causes in Pediatric Patients with Congenital Heart Diseases (CHDs). *Hellenic Journal of Cardiology* **2025**, doi:10.1016/j.hjc.2025.02.005.
178. Dohn, T.E.; Ravisankar, P.; Tirera, F.T.; Martin, K.E.; Gafranek, J.T.; Duong, T.B.; VanDyke, T.L.; Touvron, M.; Barske, L.A.; Crump, J.G.; et al. Nr2f-Dependent Allocation of Ventricular Cardiomyocyte and Pharyngeal Muscle Progenitors. *PLoS Genet* **2019**, *15*, e1007962, doi:10.1371/journal.pgen.1007962.
179. Lo, P.C.H.; Frasch, M. A Role for the COUP-TF-Related Gene *Seven-up* in the Diversification of Cardioblast Identities in the Dorsal Vessel of *Drosophila*. *Mechanisms of Development* **2001**, *104*, 49–60, doi:10.1016/S0925-4773(01)00361-6.
180. Jiang, H.; Bai, L.; Song, S.; Yin, Q.; Shi, A.; Zhou, B.; Lian, H.; Chen, H.; Xu, C.-R.; Wang, Y.; et al. EZH2 Controls Epicardial Cell Migration during Heart Development. *Life Sci Alliance* **2023**, *6*, e202201765, doi:10.26508/lsa.202201765.
181. Scott, I.C. Life Before *Nkx2.5*. In *Current Topics in Developmental Biology*; Bruneau, B.G., Ed.; Heart Development; Academic Press, 2012; Vol. 100, pp. 1–31.

182. Bodmer, R. The Gene Tinman Is Required for Specification of the Heart and Visceral Muscles in Drosophila. *Development* **1993**, *118*, 719–729, doi:10.1242/dev.118.3.719.
183. Yin, Z.; Frasch, M. Regulation and Function of Tinman during Dorsal Mesoderm Induction and Heart Specification in Drosophila. *Developmental Genetics* **1998**, *22*, 187–200, doi:10.1002/(SICI)1520-6408(1998)22:3%3C187::AID-DVG2%3E3.0.CO;2-2.
184. França, M.M.; Mendonca, B.B. Genetics of Ovarian Insufficiency and Defects of Folliculogenesis. *Best Pract Res Clin Endocrinol Metab* **2022**, *36*, 101594, doi:10.1016/j.beem.2021.101594.
185. Zhao, Y.; Wang, Y.; Shi, L.; McDonald-McGinn, D.M.; Crowley, T.B.; McGinn, D.E.; Tran, O.T.; Miller, D.; Lin, J.-R.; Zackai, E.; et al. Chromatin Regulators in the TBX1 Network Confer Risk for Conotruncal Heart Defects in 22q11.2DS. *npj Genom. Med.* **2023**, *8*, 1–15, doi:10.1038/s41525-023-00363-y.
186. Sun, Y.; Liang, X.; Najafi, N.; Cass, M.; Lin, L.; Cai, C.-L.; Chen, J.; Evans, S.M. Islet 1 Is Expressed in Distinct Cardiovascular Lineages, Including Pacemaker and Coronary Vascular Cells. *Developmental Biology* **2007**, *304*, 286–296, doi:10.1016/j.ydbio.2006.12.048.
187. Tao, Y.; Wang, J.; Tokusumi, T.; Gajewski, K.; Schulz, R.A. Requirement of the LIM Homeodomain Transcription Factor Tailup for Normal Heart and Hematopoietic Organ Formation in Drosophila Melanogaster. *Mol Cell Biol* **2007**, *27*, 3962–3969, doi:10.1128/MCB.00093-07.
188. Witzel, H.R.; Cheedipudi, S.; Gao, R.; Stainier, D.Y.R.; Dobreva, G.D. Isl2b Regulates Anterior Second Heart Field Development in Zebrafish. *Sci Rep* **2017**, *7*, 41043, doi:10.1038/srep41043.
189. Searcy, R.D.; Yutzey, K.E. Analysis of Hox gene expression during early avian heart development. *Developmental Dynamics* **1998**, *213*, 82–91, doi:10.1002/(SICI)1097-0177(199809)213:1%3C82::AID-AJA8%3E3.0.CO;2-U.
190. Garcia-Padilla, C.; Dueñas, A.; Franco, D.; Garcia-Lopez, V.; Aranega, A.; Garcia-Martinez, V.; Lopez-Sanchez, C. Dynamic MicroRNA Expression Profiles During Embryonic Development Provide Novel Insights Into Cardiac Sinus Venosus/Inflow Tract Differentiation. *Front Cell Dev Biol* **2022**, *9*, 767954, doi:10.3389/fcell.2021.767954.
191. Hrycaj, S.M.; Marty-Santos, L.; Cebrian, C.; Rasky, A.J.; Ptaschinski, C.; Lukacs, N.W.; Wellik, D.M. Hox5 Genes Direct Elastin Network Formation during Alveologenesis by Regulating Myofibroblast Adhesion. *Proc Natl Acad Sci U S A* **2018**, *115*, E10605–E10614, doi:10.1073/pnas.1807067115.
192. Morioka, N.; Ganier, C.; Watt, F.M. Fetal Fibroblast Heterogeneity Defines Dermal Architecture during Human Embryonic Skin Development. *J Invest Dermatol* **2025**, *145*, 1081-1091.e7, doi:10.1016/j.jid.2024.12.027.
193. Kang, J.; Gu, Y.; Li, P.; Johnson, B.L.; Sucov, H.M.; Thomas, P.S. PDGF-A as an Epicardial Mitogen during Heart Development. *Developmental Dynamics* **2008**, *237*, 692–701, doi:10.1002/dvdy.21469.
194. Moore, K.; Fulmer, D.; Guo, L.; Koren, N.; Glover, J.; Moore, R.; Gensemer, C.; Beck, T.; Morningstar, J.; Stairley, R.; et al. PDGFRα: Expression and Function during Mitral Valve Morphogenesis. *Journal of Cardiovascular Development and Disease* **2021**, *8*, 28, doi:10.3390/jcdd8030028.
195. Wu, M.; Sato, T.N. On the Mechanics of Cardiac Function of Drosophila Embryo. *PLOS ONE* **2008**, *3*, e4045, doi:10.1371/journal.pone.0004045.
196. Bi, Y.; Lv, Z.; Wang, Y.; Hai, T.; Huo, R.; Zhou, Z.; Zhou, Q.; Sha, J. WDR82, a Key Epigenetics-Related Factor, Plays a Crucial Role in Normal Early Embryonic Development in Mice. *Biol Reprod* **2011**, *84*, 756–764, doi:10.1095/biolreprod.110.084343.
197. Kulkarni, S.S.; Khokha, M.K. WDR5 Regulates Left-Right Patterning via Chromatin-Dependent and -Independent Functions. *Development* **2018**, *145*, dev159889, doi:10.1242/dev.159889.
198. Zhao, T.; Wang, M.; Li, Z.; Li, H.; Yuan, D.; Zhang, X.; Guo, M.; Qian, W.; Cheng, D. Wds-Mediated H3K4me3 Modification Regulates Lipid Synthesis and Transport in Drosophila. *Int J Mol Sci* **2023**, *24*, 6125, doi:10.3390/ijms24076125.
199. Cohen, E.D.; Tian, Y.; Morrisey, E.E. Wnt Signaling: An Essential Regulator of Cardiovascular Differentiation,Morphogenesis and Progenitor Self-Renewal. *Development* **2008**, *135*, 789–798, doi:10.1242/dev.016865.
200. Wu, X.; Golden, K.; Bodmer, R. Heart Development in *Drosophila* Requires the Segment Polarity Gene *Wingless*. *Developmental Biology* **1995**, *169*, 619–628, doi:10.1006/dbio.1995.1174.
201. Goddard, L.M.; Duchemin, A.-L.; Ramalingan, H.; Wu, B.; Chen, M.; Bamezai, S.; Yang, J.; Li, L.; Morley, M.; Wang, T.; et al. Hemodynamic Forces Sculpt Developing Heart Valves through a KLF2-WNT9B Paracrine Signaling Axis. *Dev Cell* **2017**, *43*, 274-289.e5, doi:10.1016/j.devcel.2017.09.023.
202. Tauc, H.M.; Mann, T.; Werner, K.; Pandur, P. A Role for Drosophila Wnt-4 in Heart Development. *genesis* **2012**, *50*, 466–481, doi:10.1002/dvg.22021.
203. Graba, Y.; Gieseler, K.; Aragnol, D.; Laurenti, P.; Mariol, M.-C.; Berenger, H.; Sagnier, T.; Pradel, J. DWnt-4, a Novel Drosophila Wnt Gene Acts Downstream of Homeotic Complex Genes in the Visceral Mesoderm. *Development* **1995**, *121*, 209–218, doi:10.1242/dev.121.1.209.

204. Chen, Z.; Zhu, J.; Fu, Y.; Richman, A.; Han, Z. Wnt4 Is Required for Ostia Development in the *Drosophila* Heart. *Developmental Biology* **2016**, *413*, 188–198, doi:10.1016/j.ydbio.2016.03.008.
205. Paolini, A.; Sharipova, D.; Lange, T.; Abdelilah-Seyfried, S. Wnt9 Directs Zebrafish Heart Tube Assembly via a Combination of Canonical and Non-Canonical Pathway Signaling. *Development* **2023**, *150*, dev201707, doi:10.1242/dev.201707.
206. Haack, T.; Schneider, M.; Schwendele, B.; Renault, A.D. *Drosophila* Heart Cell Movement to the Midline Occurs through Both Cell Autonomous Migration and Dorsal Closure. *Developmental Biology* **2014**, *396*, 169–182, doi:10.1016/j.ydbio.2014.08.033.
207. Smyth, S.S.; Kraemer, M.; Yang, L.; Van Hoose, P.; Morris, A.J. Roles for Lysophosphatidic Acid Signaling in Vascular Development and Disease. *Biochim Biophys Acta Mol Cell Biol Lipids* **2020**, *1865*, 158734, doi:10.1016/j.bbalip.2020.158734.
208. Escalante-Alcalde, D.; Morales, S.L.; Stewart, C.L. Generation of a Reporter-Null Allele of Ppap2b/Lpp3and Its Expression during Embryogenesis. *Int J Dev Biol* **2009**, *53*, 139–147, doi:10.1387/ijdb.082745de.
209. Lu, C.; Wu, X.; Meng, X.; Liu, Y.; Yang, T.; Zeng, Y.; Chen, Y.; Huang, Y.; Fang, Z.; Yang, X.; et al. Silver Nanoparticles Exposure Impairs Cardiac Development by Suppressing the Focal Adhesion Pathway in Zebrafish. *Int J Nanomedicine* **2024**, *19*, 9291–9304, doi:10.2147/IJN.S476168.
210. Schumacher, J.A.; Wright, Z.A.; Owen, M.L.; Bredemeier, N.O.; Sumanas, S. Integrin A5 and Integrin A4 Cooperate to Promote Endocardial Differentiation and Heart Morphogenesis. *Developmental Biology* **2020**, *465*, 46–57, doi:10.1016/j.ydbio.2020.06.006.
211. Moreira, C.G.A.; Jacinto, A.; Prag, S. Drosophila Integrin Adhesion Complexes Are Essential for Hemocyte Migration in Vivo. *Biology Open* **2013**, *2*, 795–801, doi:10.1242/bio.20134564.
212. Vanderploeg, J.; Vazquez Paz, L.L.; MacMullin, A.; Jacobs, J.R. Integrins Are Required for Cardioblast Polarisation in Drosophila. *BMC Developmental Biology* **2012**, *12*, 8, doi:10.1186/1471-213X-12-8.
213. Palmquist-Gomes, P.; Ruiz-Villalba, A.; Guadix, J.A.; Romero, J.P.; Bessiéres, B.; MacGrogan, D.; Conejo, L.; Ortiz, A.; Picazo, B.; Houyel, L.; et al. Origin of Congenital Coronary Arterio-Ventricular Fistulae from Anomalous Epicardial and Myocardial Development. *Exp Mol Med* **2023**, *55*, 228–239, doi:10.1038/s12276-022-00913-x.
214. Geng, Z.; Wang, J.; Pan, L.; Li, M.; Zhang, J.; Cai, X.; Chu, M. Microarray Analysis of Differential Gene Expression Profile Between Human Fetal and Adult Heart. *Pediatr Cardiol* **2017**, *38*, 700–706, doi:10.1007/s00246-017-1569-x.
215. Zhou, X.; Fang, X.; Ithychanda, S.S.; Wu, T.; Gu, Y.; Chen, C.; Wang, L.; Bogomolovas, J.; Qin, J.; Chen, J. Interaction of Filamin C With Actin Is Essential for Cardiac Development and Function. *Circ Res* **2023**, *133*, 400–411, doi:10.1161/CIRCRESAHA.123.322750.
