# Supplementary material for: In Vivo Models of Cardiovascular Disease: Drosophila melanogaster as a Genetic Model of Congenital Heart Disease
Source: Biomedicines. 2025 Oct 21;13(10):2569. doi: 10.3390/biomedicines13102569 (PMC12561791; doi:10.3390/biomedicines13102569)
Supplement: Supplementary file 1 [file biomedicines-13-02569-s001.zip › biomedicines-3903996-supplementary_File S3.pdf]

Review

In Vivo Models of Cardiovascular Disease: *Drosophila melanogaster* as a Genetic Model of Congenital Heart Disease

Supplementary Information

Theodora M Stougiannou [1,*], Maria Koutini [1], Fotios Mitropoulos [2] and Dimos Karangelis [1]

1 Department of Cardiothoracic Surgery, University General Hospital, Democritus University of Thrace, 68100 Alexandroupolis, Greece

2 Department of Paediatric, Congenital and Adult Cardiac Surgery, Mitera Hospital, Marousi, 15123 Athens, Greece

* Correspondence: theodorastougiannos@gmail.com

Supplementary Information File S3 Section C

Table S9. Complete list of gene names used throughout the manuscript and supplemental sections.

Gene abbreviation	Gene name
ABL1/2	Non-Receptor Tyrosine Kinase
ACVR1/1B/1C/2A/L1	Activin A Receptor Type 1/1B/1C/2A/L1
ADAMDEC1	ADAM Like Decysin 1
ADGRA3/D1/D2/E1/E2/E3/E5/F3/F4/F5/G2/G3/G4/G6/G7/L1/L2/L3/L4	Adhesion G Protein-Coupled Receptor 3/D1/D2/E1/E2/E3/E5/F3/F4/F5/G2/G3/G4/G6/G7/L1/L2/L3/L4
AFG3L2	AFG3 Like Matrix AAA Peptidase Subunit 2
AHI1	Abelson Helper Integration Site 1
ANKRD11-12	Ankyrin repeat domain containing 11/12
APOB	Apolipoprotein B
ARX	Aristaless Related Homeobox
ASCL1-5	Achaete-Scute Family BHLH Transcription Factor 1-5
ASH1L/2L	ASH1/2 Like Histone Lysine Methyltransferase
ATG16L2	Autophagy Related 16 Like 2
BARHL2	BarH Like Homeobox 2
BARX2	BARX Homeobox 2
BAZ1A	Bromodomain Adjacent To Zinc Finger Domain 1A
BCS1L	BCS1 Homolog, Ubiquinol-Cytochrome C Reductase Complex Chaperone
BHLHA9	Basic Helix-Loop-Helix Family Member A9
BLK	BLK Proto-Oncogene, Src Family Tyrosine Kinase
BMP2-15	Bone morphogenetic protein 2-15
BMPR1A/1B	Bone Morphogenetic Protein Receptor Type 1A/1B
BMX	Bone Marrow Tyrosine Kinase Gene In Chromosome X Protein
BORCS8	BLOC-1 Related Complex Subunit 8
BRCC3	BRCA1/BRCA2-Containing Complex Subunit 3
BSG	Basigin (Ok Blood Group)

Gene abbreviation	Gene name
BTBD18	BTB Domain Containing 18
BTF3/3L4	Basic Transcription Factor 3/ 3 Like 4
BTK	Bruton Tyrosine Kinase
CCDC158/186	Coiled-Coil Domain Containing 158/186
CD320/34/42	CD320/34/42 Molecule
CDH1/20	Cadherin 1/20
CDX1/2/4	Caudal Type Homeobox 1/2/4
CELSR1/2/3	Cadherin EGF LAG Seven-Pass G-Type Receptor 1/2/3
CFAP52	Cilia And Flagella Associated Protein 52
CHADL	Chondroadherin Like;
CHD1-9	Chromodomain Helicase DNA Binding Protein 1-9
CNTN4/6	Contactin 4/6
COPS5/6	COP9 Signalosome Subunit 5/6
CORIN	Corin, Serine Peptidase
CRB1/2	Crumbs Cell Polarity Complex Component 1/1
CSF1R	Colony Stimulating Factor 1 Receptor
CSK	C-Terminal Src Kinase
DAB1	DAB Adaptor Protein 1
DAG1	Dystroglycan 1
DAW1	Dynein Assembly Factor With WD Repeats 1
DBX1/2	Developing Brain Homeobox 1/2
DCAF11	DDB1 And CUL4 Associated Factor 11
DHH	Desert Hedgehog Signaling Molecule
DLL1	Delta Like Canonical Notch Ligand 1
DLX1-6	Distal-Less Homeobox 1-6
DNER	Delta/Notch Like EGF Repeat Containing
DOLPP1	Dolichyldiphosphatase 1

Gene abbreviation	Gene name
DPY30	Dpy-30 Histone Methyltransferase Complex Regulatory Subunit
DSCAM	DS Cell Adhesion Molecule
DSCAML1	DS Cell Adhesion Molecule Like 1
EDIL3	EGF Like Repeats And Discoidin Domains 3
EGF	Epidermal Growth Factor
EGFR	Epidermal Growth Factor Receptor
EHD2/3	EH Domain Containing 2/3
EHF	ETS Homologous Factor
EHMT1/2	Euchromatic Histone Lysine Methyltransferase 1/2
EIF3F	Eukaryotic Translation Initiation Factor 3 Subunit F
ELF5	E74 Like ETS Transcription Factor 5
ELSPBP1	Epididymal Sperm Binding Protein 1
EMX1/2	Empty Spiracles Homeobox 1/2
EOMES	Eomesodermin
EPHA10	EPH Receptor A10;
EPS15	Epidermal Growth Factor Receptor Pathway Substrate 15;
ERBB2-4	Erb-B2 Receptor Tyrosine Kinase 2-4
ESRRB	Estrogen Related Receptor Beta
EVX1/2	Even-Skipped Homeobox 1/2
EZH1/2	Enhancer Of Zeste 1/2 Polycomb Repressive Complex 2 Subunit
F9	Coagulation Factor IX
FAM43A/B	Family With Sequence Similarity 43 Member A
FAT4	FAT Atypical Cadherin 4
FBLN5/7	Fibulin 5/7
FBN1-3	Fibrillin 1-3
FBXO42	F-Box Protein 42
FBXW2	F-Box And WD Repeat Domain Containing 2

Gene abbreviation	Gene name
FCHSD1	FCH And Double SH3 Domains 1
FDPS	Farnesyl Diphosphate Synthase
FER	FER Tyrosine Kinase
FERD3L	Fer3 Like BHLH Transcription Factor
FES	FES Proto-Oncogene, Tyrosine Kinase
FGF8	Fibroblast growth factor 8
FGFR1/2/3/4/L1	Fibroblast Growth Factor Receptor 1/2/3/4/L1
FGR	FGR Proto-Oncogene, Src Family Tyrosine Kinase
FIGLA	Folliculogenesis Specific BHLH Transcription Factor
FRZB	Frizzled Related Protein
FYN	FYN Proto-Oncogene, Src Family Tyrosine Kinase
FZD1/10/2/3/4/5/6/7/8/9	Frizzled Class Receptor 1/10/2/3/4/5/6/7/8/9
G2E3	G2/M-Phase Specific E3 Ubiquitin Protein Ligase
GATA1-6	GATA Binding Protein 1-6
GBX1/2	Gastrulation Brain Homeobox 1/2
GDF1-10	Growth Differentiation Factor 1-10
GGPS1	Geranylgeranyl Diphosphate Synthase 1
GRAP/2/L	GRB2 Related Adaptor Protein
GRB14/2	Growth Factor Receptor Bound Protein 14/2
GSX1/2	GS Homeobox 1/2
GULP1	GULP PTB Domain Containing Engulfment Adaptor 1
HCFC1/2	Host Cell Factor C1/2
HCK	HCK Proto-Oncogene, Src Family Tyrosine Kinase
HMCN1/2	Hemicentin 1/2
HOXA1/10/11/2/3/4/5/6/7/9	Homeobox A1/10/11/2/3/4/5/6/7/9
HOXB1/13/2/3/4/5/6/7/8/9	Homeobox B1/13/2/3/4/5/6/7/8/9
HOXC10/11/12/4/5/6/8/9	Homeobox C10/11/12/4/5/6/8/9

Gene abbreviation	Gene name
HOXD1/10/11/12/13/3/4/8/9	Homeobox D1/10/11/12/13/3/4/8/9
HPX	Hemopexin
HSPG2	Heparan Sulfate Proteoglycan 2
IGDCC3	Immunoglobulin Superfamily DCC Subclass Member 3
IGF1R	Insulin Like Growth Factor 1 Receptor
IGLON5	IgLON Family Member 5
IGSF9	Immunoglobulin Superfamily Member 9/9B
IHH	Indian Hedgehog Signaling Molecule
INHA	Inhibin Subunit A/BA/B/C/E
ISL1/2	ISL LIM Homeobox 1/2
ITGA1/10/11/2/2B/3/4/5/6/7/8/9/D/E/L/M/V/X	Integrin Subunit Alpha 1/10/11/2/2B/3/4/5/6/7/8/9/D/E/L/M/V/X
ITGB1/2/3/4/5/6/7/8/L1	Integrin Subunit Beta 1/2/3/4/5/6/7/8/L1
ITK	IL2 Inducible T Cell Kinase
ITSN1/2	Intersectin 1/2
JAG1/2	Jagged Canonical Notch Ligand 1/2
JAK2/3	Janus Kinase 2/3
JARID2	Jumonji And AT-Rich Interaction Domain Containing 2
KBTBD4	Kelch Repeat And BTB Domain Containing 4
KDM4A/4B/4D/4E/4F/5A/5B/5C/5D	Lysine Demethylase 4A/4B/4D/4E/4F/5A/5B/5C/5D
KDR	Kinase Insert Domain Receptor
KIF13A/13B/14/16B/1A/1B/1C/2B	Kinesin Family Member 13A/13B/14/16B/1A/1B/1C/2B
KIRREL1-3	Kirre Like Nephrin Family Adhesion Molecule 1-3
KIT	KIT Proto-Oncogene, Receptor Tyrosine Kinase
KLHDC1/C3/C4	Kelch Domain Containing 1/3/4
LAM A1/5/B3/C1-3	Laminin Subunit A1/5/B3/C1-3

Gene abbreviation	Gene name
LCK	LCK Proto-Oncogene, Src Family Tyrosine Kinase
LDLR	Low Density Lipoprotein Receptor
LDLRAD3	Low Density Lipoprotein Receptor Class A Domain Containing 3
LDLRAP1	Low Density Lipoprotein Receptor Adaptor Protein 1
LEFTY1/2	Left-Right Determination Factor 1/2
LEP	Leptin
LGR6	Leucine Rich Repeat Containing G Protein-Coupled Receptor 6
LRP1/10/12/1B/2/3/4/5/6/8	LDL Receptor Related Protein 1/10/12/1B/2/3/4/5/6/8; LRRC3/3B/3C
LRTM1/2	Leucine Rich Repeat Transmembrane Protein 1/2
LYL1	LYL1 Basic Helix-Loop-Helix Family Member
LYN	LYN Proto-Oncogene, Src Family Tyrosine Kinase
LZTR1	Leucine Zipper Like Post Translational Regulator 1
MAP3K10	Mitogen-Activated Protein Kinase Kinase Kinase 10
MAPK8IP1-2	Mitogen-Activated Protein Kinase 8 Interacting Protein 1-2
MATK	Megakaryocyte-Associated Tyrosine Kinase
MATN2	Matrilin 2
MDC1	Mediator Of DNA Damage Checkpoint 1
MDGA2	MAM Domain Containing Glycosylphosphatidylinositol Anchor 2
MEGF10/11/6/9	Multiple EGF Like Domains 10/11/6/9
MEN1	Menin 1
MEOX1-2	Mesenchyme Homeobox 1-2
MESP1-2	Mesoderm Posterior BHLH Transcription Factor 1-2
MET	MET Proto-Oncogene, Receptor Tyrosine Kinase
MFRP	Membrane Frizzled-Related Protein
MGA	MAX Dimerization Protein MGA
MNX1	Motor Neuron And Pancreas Homeobox 1
MSC	Musculin

Gene abbreviation	Gene name
MSGN1	Mesogenin 1
MST1R	Macrophage Stimulating 1 Receptor
MSTN	Myostatin
MSX1-2	Msh Homeobox 1-2
MYBPC1-3	Myosin Binding Protein C1-3
MYOM2	Myomesin 2
MYSM1	Myb Like, SWIRM And MPN Domains 1
NCAM1/2	Neural Cell Adhesion Molecule 1/2
NCK1/2	NCK Adaptor Protein 1/2
NHLH1	Nescient Helix-Loop-Helix 1
NKX2-1-8	NK2 Homeobox 1-8
NKX3-1/2	NK3 Homeobox 1/2
NKX6-1/2/3	NK6 Homeobox 1/2/3
NODAL	Nodal Growth Differentiation Factor
NOS1AP	Nitric Oxide Synthase 1 Adaptor Protein
NOTCH1/2/2NLA/2NLC/2NLR/3/4	Notch Receptor 1/2/2 N-Terminal Like A/2 N-Terminal Like C/2 N-Terminal Like R/3/4
NOTO	Notochord Homeobox
NR1D1-2	Nuclear Receptor Subfamily 1 Group D Member 1-2
NR1H2-4	Nuclear Receptor Subfamily 1 Group H Member 2-4
NR1I2-3	Nuclear Receptor Subfamily 1 Group I Member 2-3
NR2C2/E1/E3/F1/F2/F6	Nuclear Receptor Subfamily 2 Group C Member 2/ E Member 1/3/ F Member 1/2/6
NSD1-3	Nuclear Receptor Binding SET Domain Protein 1-3
NTM	Neurotrimin
NTN1/3/5	Netrin 1/3/5
NUMB/L	NUMB/NUMB Like Endocytic Adaptor Protein
NUP37	Nucleoporin 37

Gene abbreviation	Gene name
NYX	Nyctalopin
OBSL1	Obscurin Like Cytoskeletal Adaptor 1
PAAF1	Proteasomal ATPase Associated Factor 1
PACSIN1-3	Protein Kinase C And Casein Kinase Substrate In Neurons 1-3
PAFAH1B1	Platelet Activating Factor Acetylhydrolase 1b Regulatory Subunit 1
PARL	Presenilin Associated Rhomboid Like
PAXIP1	PAX Interacting Protein 1
PDGFA/B/C/D	Platelet Derived Growth Factor Subunit A/B/C/D
PDGFRA/B	Platelet Derived Growth Factor Receptor A/B
PDSS1/2	Decaprenyl Diphosphate Synthase Subunit 1/2
PDX1	Pancreatic And Duodenal Homeobox 1
PEX10	Peroxisomal Biogenesis Factor 10
PGF	Placental Growth Factor
PHF11/6/7	PHD Finger Protein 11/6/7
PLPP1/2/3/4/5	Phospholipid Phosphatase 1/2/3/4/5
PLPPR1/2/3/4/5	Phospholipid Phosphatase Related 1/2/3/4/5
PLRG1	Pleiotropic Regulator 1
POC1A	POC1 Centriolar Protein A
PPARA/D/G	Peroxisome Proliferator Activated Receptor A/D/G
PRG4	Proteoglycan 4
PSMD14/7	Proteasome 26S Subunit, Non-ATPase 14/7
PTF1A	Pancreas Associated Transcription Factor 1a
PTK2/6	Protein Tyrosine Kinase 2/6
PTPN6	Protein Tyrosine Phosphatase Non-Receptor Type 6
PTPRQ	Protein Tyrosine Phosphatase Receptor Type Q
PYGO1/2	Pygopus Family PHD Finger 1/2
RABEPK	Rab9 Effector Protein With Kelch Motifs

Gene abbreviation	Gene name
RAC1/2/3	Rac Family Small GTPase 1/2/3
RARA/B	Retinoic Acid Receptor A/B
RAX/2	Retina And Anterior Neural Fold Homeobox/2
RBBP5	RB Binding Protein 5, Histone Lysine Methyltransferase Complex Subunit
REPS1	RALBP1 Associated Eps Domain Containing 1
RET	Ret Proto-Oncogene
RHBDF1/F2/L1/L2/L3	Rhomboid 5 Homolog 1/2/ Rhomboid Like Homolog 1/2/3
RHEB	Ras Homolog, MTORC1 Binding
RHOA/B/BTB1/BTB2/D/F/G/H/J/Q/U/V	Ras Homolog Family Member A/B/BTB1/BTB2/D/F/G/H/J/Q/U/V
RIPK1	Receptor Interacting Serine/Threonine Kinase 1
RND1-3	Rho Family GTPase 1-3
RNF20/4/40	Ring Finger Protein 20/4/40
ROBO1-4	Roundabout Guidance Receptor 1-4
ROR1/A/B/C	Receptor Tyrosine Kinase Like Orphan Receptor 1/A/B/C
ROS1	ROS Proto-Oncogene 1, Receptor Tyrosine Kinase
RTN4RL1	Reticulon 4 Receptor Like 1
RXRA/B/G	Retinoid X Receptor A/B/G
RYK	Receptor Like Tyrosine Kinase
SCARF1	Scavenger Receptor Class F Member 1
SCX	Scleraxis BHLH Transcription Factor
SDK1/2	Sidekick Cell Adhesion Molecule 1/2
SETBP1	SET Binding Protein 1
SETD1A/1B/2/B1/B2	SET Domain Containing 1A/1B/2/B1/B2, Histone Lysine Methyltransferase
SFRP1-5	Secreted Frizzled Related Protein 1-5
SH3D19	SH3 Domain Containing 19
SHH	Sonic Hedgehog

Gene abbreviation	Gene name
SHOXA5	Short Stature Homeobox A5
SLA	Src Like Adaptor/2
SLIT1-3	Slit Guidance Ligand 1-3
SLITRK4	SLIT And NTRK Like Family Member 4
SMAD1-9	SMAD Family Member 1-9
SMO	Smoothened, Frizzled Class Receptor
SNED1	Sushi, Nidogen And EGF Like Domains 1
SORL1	Sortilin Related Receptor 1
SPATA5L1	Spermatogenesis associated 5L1
SPG7	SPG7 Matrix AAA Peptidase Subunit, Paraplegin
SRC	SRC Proto-Oncogene, Non-Receptor Tyrosine Kinase
SRMS	Src-Related Kinase Lacking C-Terminal Regulatory Tyrosine And N-Terminal Myristylation Sites
STAM2	Signal Transducing Adaptor Molecule 2
STAMBP/L1	STAM Binding Protein
STARD9	StAR Related Lipid Transfer Domain Containing 9
STYK1	Serine/Threonine/Tyrosine Kinase 1
SUV39H2	SUV39H2 Histone Lysine Methyltransferase
TAF5	TATA-Box Binding Protein Associated Factor 5
TAL1/2	TAL BHLH Transcription Factor 1/2, Erythroid Differentiation Factor
TBL1X	Transducin Beta Like 1 X-Linked
TBL1XR1	TBL1X/Y Related 1
TBL1Y	Transducin Beta Like 1 Y-Linked
TBL3	Transducin Beta Like 3
TBR1	T-Box Brain Transcription Factor 1
TBRG1	Transforming Growth Factor Beta Regulator 1
TBX1-22	T-Box Transcription Factor 1-22

Gene abbreviation	Gene name
TBXT	T-Box Transcription Factor T
TCF15-24	Transcription Factor 15-24
TEC	Tec Protein Tyrosine Kinase
TEK	TEK Receptor Tyrosine Kinase
TGFB1/B2/B3	Transforming Growth Factor B1/B2/B3
TGFBR1	Transforming Growth Factor Beta Receptor 1
THRA	Thyroid Hormone Receptor Alpha
TIE1	Tyrosine Kinase With Immunoglobulin Like And EGF Like Domains 1
TIMP1-4	Tissue inhibitor of metalloproteinase 1-4
TLR7	Toll Like Receptor 7
TLX2	T Cell Leukemia Homeobox 2
TOPBP1	DNA Topoisomerase II Binding Protein 1
TRIP10	Thyroid Hormone Receptor Interactor 10
TRPS1	Transcriptional Repressor GATA Binding 1
TTN	Titin
TWIST1/2	Twist Family BHLH Transcription Factor 1/2
TXK	TXK Tyrosine Kinase
TYK2	Tyrosine Kinase 2
UBE2A/B/O/U	Ubiquitin Conjugating Enzyme E2 A/B/O/U
USH2A	Usherin
USP17L1-30	Ubiquitin Specific Peptidase 17 Like Family Member 1-30
USP27X	Ubiquitin Specific Peptidase 27 X-Linked
USP3-49	Ubiquitin Specific Peptidase 3-49
VAX1/2	Ventral Anterior Homeobox 1/2
VCAN	Versican
VDR	Vitamin D Receptor
VEGFA/B/C/D	Vascular endothelial growth factor A/B/C/D

Gene abbreviation	Gene name
VLDLR	Very Low Density Lipoprotein Receptor
VTN	Vitronectin
WDR5-88	WD Repeat Domain 5-88
WNT1-9	Wingless-related integration site 1-9
WSB2	WD Repeat And SOCS Box Containing 2
YES1	YES Proto-Oncogene 1
YME1L1	YME1 Like 1 ATPase
ZGLP1	Zinc Finger GATA Like Protein 1
ADAM2-33	A Disintegrin and Metalloproteinase 2-33
ANTP-C	Antennapedia Complex
Abd-A	Abdominal-A
Abd-B	Abdominal-B
Ankrd11	Ankyrin repeat domain containing 11
Antp	Antennapedia
Ash1/2	Absent, small or homeotic disks 1/2
BARX	BarH-like homeobox
BARHL	BarH-like homeobox
BMP	Bone morphogenetic protein
BTBD18	BTB Domain Containing 18
BX-C	Bithorax Complex
Bre1	B recognition element 1
Cdc42	Cell division control protein 42
Col6A2	Collagen Type VI α2 chain
D-mef2	Drosophila Myocyte enhancer factor 2
DE-Cadherin	Drosophila E-Cadherin
DWnt4	Drosophila Wingless-related integration site 4
Dap160	Dynamin-associated protein 160 kilo Dalton (kD)

Gene abbreviation	Gene name
Dfz2	Dfrizzled-2
Dg	Dystroglycan
Dmef2	Drosophila myocyte enhancer factor 2
Dome	domeless
Donson	Downstream neighbour of Son
Dpp	Decapentaplegic
Dpy-30L1	Dpy-30 like 1
Dscam	Down syndrome Cell adhesion molecule
EGF	Epidermal growth factor
EGFR	Epidermal growth factor receptor
EcR	Ecdysone receptor
Eve	Even-skipped
FGF	Fibroblast growth factor
FGFR	Fibroblast growth factor receptor
Fz/2	Frizzled/2
GGPPS/qm	Geranylgeranyl pyrophosphate synthase
GPCR	G-protein coupled receptor
Gart	Phosphoribosylglycinamide Formyltransferase, Phosphoribosylglycinamide synthetase, Phosphoribosylaminoimidazole synthetase
Gata4	GATA binding protein 4
Gia	G-protein coupled receptor in aorta
Gγ1	G protein gamma (γ) subunit 1
HMGCR	Hydroxymethyl-glutaryl (HMG) CoA reductase
Hand	Heart- and Neural crest derivatives-expressed protein
Hd	Humpty dumpy
Hh	Hedgehog
Hox	Homeobox

Gene abbreviation	Gene name
Isl1	Insulin-related protein 1
Itsn1	Intersectin 1
KMT2A/2B/2C/2D	Lysine methyltransferase 2A/2B/2C/2D
Kif1A	Kinesin family member 1A
Kuz	Kuzbanian
LRP2	Low-density lipoprotein-related receptor 2
Lb	Ladybird
Lbx	Ladybird homeobox
Lid	Drosophila ortholog of KDM5A/5B
Lpt	Lost PHDs of Trithorax-related (Trr)
MICOS	Mitochondrial contact site and cristae organization system
MMP1-29	Matrix metalloproteinase 1-29
MYOM2	Myomesin 2
Mef2A/2B/2C/2D	Myocyte enhancer factor 2A/2B/2C/2D
Mnn1	Menin 1
Msh	MutS homolog
Msx	MutS homolog (Msh) homeobox
Naca	Nascent-associated polypeptide complex, alpha subunit
NetA/B	netrin A/B
Nkx2/6	Nk2/6 homeobox
Nr2f2	Nuclear Receptor subfamily 2 group F member 2
Odd	Odd-skipped
Org-1	Optomotor blind related gene 1
PVR	Platelet-derived growth factor/Vascular endothelial growth factor receptor
Pdss2	Decaprenyl diphosphate synthase, subunit 2
Ptip	PAX Transcription activation domain interacting protein
Rbbp5	Retinoblastoma binding protein 5

Gene abbreviation	Gene name
Rho	Rhomboid
Robo2	Roundabout2
RpL13	Ribosomal protein L13- L14
RpS24	Ribosomal protein S24
Rpn8	Regulatory particle, Proteasome subunit Rpn8
SCE	sevenup (svp) cardiac enhancer
Scny	Scrawny
Set1	SET Containing domain 1
Set2	SET Containing domain 2
Shh	Sonic hedgehog
Sli	Slit
Smox	drosophila ortholog of SMAD2
Son	SON Deoxyribonucleic acid (DNA) and Ribonucleic acid (RNA)
Spg7	Spastic paraplegia 7
Scr	Sex combs reduced
How	Held out wings
Stat	Signal transducer and activator of transcription
Stat92E	Signal transducer and activator of transcription 92E
Tbx1/2/20/5/6	T-box transcription factor TBX1/2/20/5/6
Tkv	Thickveins
TrxG	Trithorax Group genes
UbcD6	D. melanogaster ortholog of Ubiquitin conjugating enzyme (UBE)
Ubx	Ultrabithorax
Upd	Unpaired
Vegf	Vascular endothelial growth factor
Wdr82	WD repeat domain 82
Wds	Will die slowly

Gene abbreviation	Gene name
Wg	Wingless
Wnt	Wingless-related integration site
Wun	Wunen
Wun2	Wunen2
apoLpp	a homolog of apolipoprotein B
bHLH	basic Helix-Loop-Helix
bab2	bric-a-brac 2
bic	bicaudal
dChchd3/6	drosophila Coiled-coil-helix-coiled-coil-helix-domain containing protein 6
dMnM	, Drosophila Myomesin and Myosin protein
doc	dorsocross
lanA	Laminin A
lb	ladybird
mgl	megalin
mid	midline
mmp1	Matrix metalloproteinase 1
mmp2	matrix metalloproteinase 2
mys	myospheroid
nmr1	neuromancer 1
nmr2	neuromancer 2
omb	optomotor blind
pnr	pannier
pyr	pyramus
scb	scab
spdo	sanpodo
srp	serpent
svp	Sevenup

Gene abbreviation	Gene name
ths	thisbe
tin	tinman
tup	tailup
αPS2	α subunit integrin chain 2
αPS3	α subunit integrin chain 3
βGGT-I	Geranylgeranyl transferase type I beta (β) subunit
βPS	β subunit integrin chain
